# Taxonomic revision of the genus *Cranichis* (Orchidaceae, Cranichideae) in Colombia

**DOI:** 10.7717/peerj.7385

**Published:** 2019-08-09

**Authors:** Dariusz L. Szlachetko, Marta Kolanowska

**Affiliations:** 1 Department of Plant Taxonomy and Nature Conservation, Faculty of Biology, University of Gdańsk, Gdańsk, Poland; 2 Department of Geobotany and Plant Ecology, Faculty of Biology and Environmental Protection, University of Lodz, Lodz, Poland; 3 Department of Biodiversity Research, Global Change Research Institute AS CR, Brno, Czech Republic

**Keywords:** Biodiversity, Cranichideae, Neotropics, New species

## Abstract

The geographical range of the orchid genus *Cranichis* extends from USA, Central America, and the Caribbean to Bolivia and Argentina—with the greatest diversity observed in the Andean region. This taxon embraces herbs with the scape and basal leaves developing from a single, terminal bud and having the scape enclosed in several sheaths. The small, non-resupinate, flowers are arranged in a racemose inflorescence. The petals and lip are free from the column part and their gynostemium is short and massive. In this paper, the synopsis of *Cranichis* in Colombia is presented. Each species occurring in the country is characterized and illustrated. The information about habitat and distribution of national genus representatives are provided. Several groups of species that are very similar in their morphology are delineated to facilitate process of species identification. A total of 10 species are described here as new. Lectotypes for the six species *Cranichis ciliata*, *C. fendleri*, *C. mandonii*, *C. tenuis*, *C. viereckii*, and *C. wageneri* have been selected. Additionally, two new nomenclatorial combinations within *Ocampoa* are proposed.

## Introduction

The orchid genus *Cranichis* (Orchidaceae, Cranichideae) was described in late 18th century by Swartz (1788). The generic name derived from the Greek *kranos* means “helmet” and it was given in reference to the lip form which is uppermost and often deeply concave. Swartz included in his newly created genus five species: *Cranichis aphylla* Sw., *C. diphylla* Sw., *C. oligantha* Sw., *C. stachyodes* Sw., and *C. muscosa* Sw. About 150 years later the latter taxon was designated as the genus type (Acuña, 1939).

Until today about 120 specific epithets have been applied to *Cranichis*; however, many of them were transferred to other genera, e.g., *Exalaria* Garay & G.A. Romero, *Ocampoa* A. Rich. & Galeotti, and *Ponthieva* R. Br. (Dodson, 1996; Garay & Romero González, 1999; Williams, 1972; Szlachetko & Kolanowska, 2017b). Numerous new *Cranichis* species were discovered by Schlechter in the early 20th century (Schlechter, 1917, 1920, 1921, 1923). Within the past 20 years, only 10 members of the genus have been described (Christenson, 2004; Kolanowska & Szlachetko, 2014; Szlachetko & Kolanowska, 2013a, 2013b). According to Chase et al. (2015), 53 species are currently accepted within *Cranichis*.

*Cranichis* was placed in the subtribe Cranichidinae based on morphological studies (Pfitzer, 1887; Dressler, 1993; Szlachetko, 1995), later confirmed by molecular analyses (Álvarez-Molina & Cameron, 2009; Chase et al., 2015). Álvarez-Molina & Cameron (2009) demonstrated that its closest relatives are *Baskervilla* Lindl., *Ponthieva*, *Exalaria*, and *Pterichis* Lindl. which are distinguished by their scape and leaves, the shape of the gynostemium, adnation of the petals to the columnar part, and fusion of the lateral sepals.

As currently recognized, *Cranichis* are herbs with scape and leaves developing from a single, terminal bud, the basal leaves and the scape being enclosed in several sheaths. The small, nonresupinate flowers are arranged in a racemose inflorescence, petals and lip are free from the columnar part and the gynostemium is short and massive. Most species are terrestrial or lithophytic, though epiphytes are also found. The altitudinal range extends from lowland areas to high mountains (above 3,000 m). Plants usually are found growing in the litter layer in shady places of various forest types (rainforest, premontane forest, montane forest, cloud forest).

The geographical range of *Cranichis* extends from the USA (Florida) through Central America and the Caribbean to Bolivia and Argentina with the greatest diversity observed in the Andean region from Venezuela to Peru (Cribb in Pridgeon et al., 2003). Schneider (1953) lists 19 species of the genus from Colombia considering *C. guatemalensis* Schltr., *C. ovatilabia* Schltr., *C. alfredii* Schltr. as synonyms of *C. diphyla* Sw. and *C. pilosa* Fawc. & Rendle, *C. subcordata* Schltr., *C. costaricensis* Schltr., *C. vierecki* Ames as synonyms of *C. wageneri* Rchb. f. Several new species were found in the country in 2013 (Kolanowska & Szlachetko, 2014; Szlachetko & Kolanowska, 2013a, 2013b). In the most recent catalogue of Colombian plants and lichens, Bernal, Gradstein & Celis (2016) listed a total of 17 *Cranichis* species, three of which actually belonging to *Ocampoa* (*C. crumenifera*, *C. gibbosa*, *C. lehmanniana*; Szlachetko & Kolanowska, 2017b).

The aim of the present study has been to provide a taxonomic revision of the orchid genus *Cranichis* in Colombia. While comprehensive orchid floras have already been published for all areas that border Colombia (Dunsterville & Garay, 1966; Foldats, 1969; Garay, 1978; Williams, Allen & Dressler, 1980), the species composition of Orchidaceae in this region remains poorly recognized and new species are continuously being discovered in the country (Bonilla Morales, Mosquera Hernández & Petini-Benelli, 2017; Moreno, Vieira-Uribe & Karremans, 2017). The most recent compilation of Colombian plants and lichens (Bernal, Gradstein & Celis, 2016) lists all species, but so far the only study also providing characteristics of national species merely covers the basal-most orchids (Szlachetko & Kolanowska, 2017a).

Here, we present the morphological characteristics of each Colombian species of *Cranichis* together along with an illustration of details of the perianth segments. A key to species identification and notes on the taxonomic affinities of the studied taxa are provided.

## Materials and Methods

We examined by standard procedures a total of about 400 specimens and illustrations deposited or borrowed from the following herbaria AAU, AMES, BM, C, CAY, CUVC, COL, FMB, K, MO, NY, P, PSO, RENZ, RPSC, UGDA, US, VALLE, and W. Additionally, we screened the electronic database of the digitalized herbarium specimens from RENZ. Herbaria acronyms are cited according to “*Index Herbariorum*” (Thiers, 2018). Each studied specimen was photographed and the data from the labels were recorded. The arrangement of leaves, petiole length, as well as blade shape and size were examined first along with the length and surface of scape and rachis. The flowers of each specimen were studied after softening in boiling water. The form and size of the floral bracts and ovaries as well as the perianth segment were studied by stereomicroscopy.

Due to the close similarity of the vegetative parts among many species of *Cranichis* we focussed on the floral traits which we consider as taxonomically informative. The infrapopulational stability of the diagnostic features used in species delimitation in previous treatments of this genus (Schlechter, 1919, 1920, 1921, 1923; Schneider, 1953; Schweinfurth, 1970; Foldats, 1969; Dueñas Gómez & Fernández Alonso, 2009) was verified by examining several specimens placed on the same herbarium sheet. Moreover, the morphology of flowers preserved in alcohol and glued to herbarium sheets was compared to evaluate the possible alterations caused by the drying of specimens.

Distribution maps were prepared using ArcGIS 9.3 (Esri, Redlands, CA, USA) along with a digital elevation model. Each locality was precisely georeferenced based on the information from herbarium labels and assigned to one of terrestrial ecoregions as described by Olson et al. (2001). The countries in the “distribution” section of characteristics of each species are listed in geographical order (from north to south, west to east).

### Nomenclature

The electronic version of this article in portable document format will represent a published work according to the International Code of Nomenclature for algae, fungi, and plants (Turland et al., 2018), and hence the new names contained in the electronic version are effectively published under that Code from the electronic edition alone. In addition, new names contained in this work that have been issued with identifiers by IPNI will eventually be made available to the Global Names Index. The IPNI Life Science Identifiers (LSIDs) can be resolved and the associated information viewed through any standard web browser by appending the LSID contained in this publication to the prefix “http://ipni.org/.” The online version of this work is archived and available from the following digital repositories: PeerJ, PubMed Central, and CLOCKSS.

## Results

In our study, we consider lip morphology (shape and ornamentation) as the most taxonomically informative character. Other essential traits used for distinguishing particular taxa are tepal shape and ornamentation as well as form of ovary surface. We did not find any differences in tepal shape or surface ornamentation in dried material and flowers preserved in alcohol.

The compiled list of Colombian *Cranichis* includes a total of 42 species, of which 10 are new species described in this paper. We compared our findings with earlier papers by Schneider (1953), Dueñas Gómez & Fernández-Alonso (2009) and lists of Colombian Orchidaceae by Ortiz Valdivieso & Uribe Vélez (2007) and more recently by Bernal, Gradstein & Celis (2016). We could not confirm the occurrence in Colombia of *C. picta* Rchb.f., *C. tenuiflora* Griseb. (regarded as synonym of *C. diphylla* by several authors), and *C. tenuis* Rchb. f. 17 species of *Cranichis* are Colombian endemics. That group includes numerous recently discovered species (*C. pennellii* Szlach. & Kolan., *C. roldanii* Szlach. & Kolan., *C. callejasii* Szlach. & Kolan., *C. juajibioyi* Szlach. & Kolan., *C. neglecta* Szlach. & Kolan., *C. popayanensis* Szlach. & Kolan., *C. schultesii* Szlach. & Kolan.*, C. queremalensis* Szlach. & Kolan., *C. rotundifolia* Szlach. & Kolan., *C. schlechteri* Szlach. & Kolan., *C. cristalinensis* Szlach. & Kolan. and *C. zarucchii* Szlach. & Kolan.), 10 species described as new in this paper, and five species described in the early 20th century (*C. atrata* Schltr., *C. brachyblephara* Schltr., *C. ovatilabia* Schltr., *C. pleioneura* Schltr., *C. polyblephara* Schltr.). Lectotypes for six species were selected: *C. ciliata*, *C. fendleri*, *C. mandonii*, *C. tenuis*, *C. viereckii*, and *C. wageneri*.

**Figure 1:**
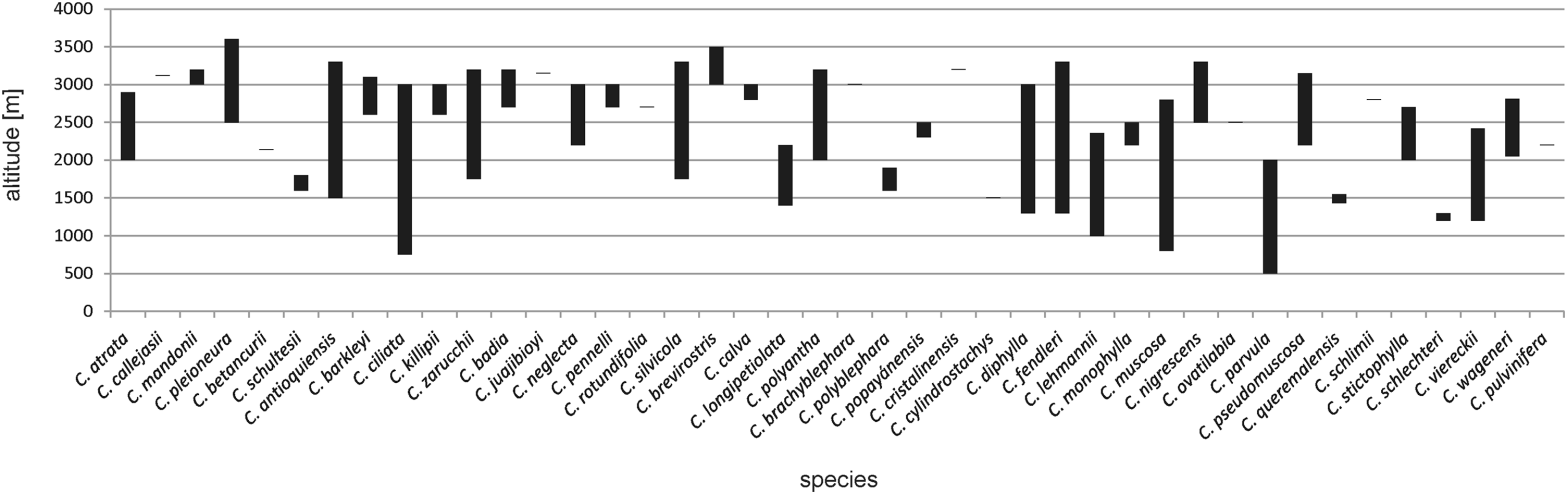
Altitudinal range of Colombian *Cranichis*.

Numerous Colombian *Cranichis* representatives are restricted in their distribution to the Andean region. However, five species have also been reported from Central America (*C. ciliata* (Kunth) Kunth, *C. diphylla* Sw., *C. muscosa* Sw., *C. nigrescens* Schltr., and *C. wageneri* Rchb.f.). In Colombia, species of *Cranichis* are found growing at altitudes of 500–3,200 m (Fig. 1). The greatest altitudinal amplitude is observed in *C. ciliata* (2,250 m range), and *C. fendleri* Schltr. and *C. muscosa* (each at the 2,000 m range). The plants grow in disturbed forests, along roadsides, as well as in wet premontane, montane, and cloud forests as well as in paramo. *C. mandonii* was found in a *Cupressus* plantation.

**Figure 2:**
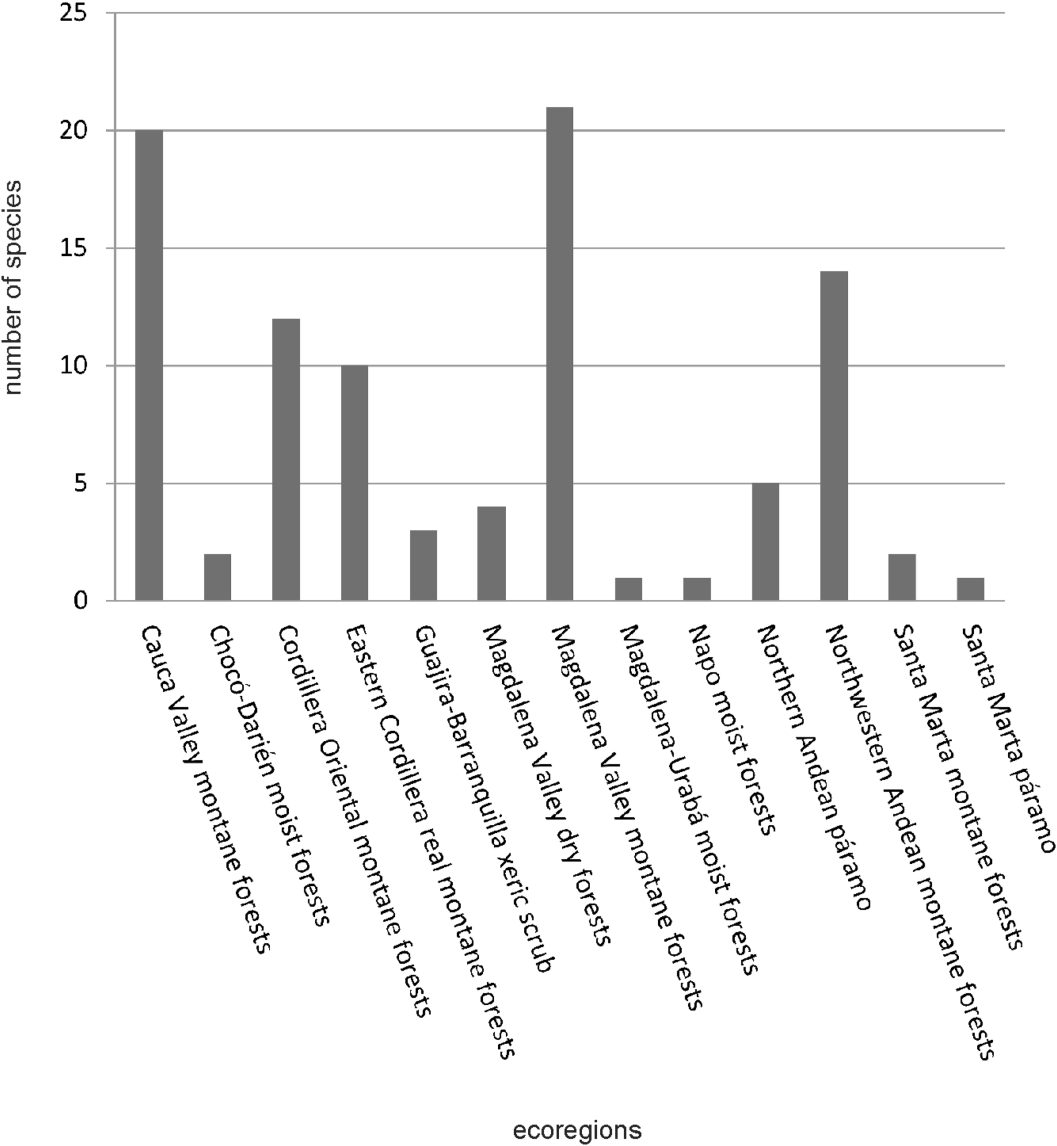
Ecoregional species richness of Colombian *Cranichis*.

The largest number of *Cranichis* species occur in the Cauca and Magdalena river valleys—21 in Magdalena Valley montane forests, four in Magdalena Valley dry forests, and 20 in Cauca Valley montane forests (Fig. 2). Other species-rich ecoregions are Northwestern Andean montane forests (14 species), Cordillera Oriental montane forests (12 species), and Eastern Cordillera Real montane forests (10 species).

## Taxonomic Treatment

***Cranichis*** Sw.
Nov. Gen. Sp. Pl. Prodr. 8: 120. 1788. TYPE: *Cranichis muscosa* Sw.

Plants terrestrial, lithophytic or rarely epiphytic. Roots fasciculated, villous-hairy. Leaves one to many, basal, fleshy, commonly with a distinct petiole, suberect or arcuately spreading. Scape erect with a few adpressed sheaths. Inflorescence racemose. Flowers rather small, non-resupinate. Sepals free, subequal, more or less spreading. Petals much narrower, often ciliolate, pubescent or glandular on margin. Lip uppermost, usually rather fleshy, cochleate, entire or lobed, often with conspicuously marked or colored reticulate veins, sometimes with various numbers of nodules (knob-like projections or irregularly subglobose thickenings). Gynostemium usually short, erect, relatively massive, often swollen at the apex. Column foot absent. Anther erect, oblong to ovate, motile. Pollinia 4, ovoid to oblong-ovoid, compact. Caudiculae inconspicuous. Staminodes producing a prominent dorsal clinandrium; clinandrium usually thick, massive, spacious. Stigma horizontal to subventral, obscurely 3-lobed, confluent, oval to elliptic, flat, usually surrounded by a high, thick rim. Rostellum erect, finger-like to triangular, acute to blunt, rather thick and massive. Viscidium single, detachable, cellular, thick, relatively small. Hamulus usually well developed, elongate, finger-like, thick, directed toward the anther. Rostellum remnant truncate at the apex in most species.

The genus *Cranichis* embraces species with predominantly inconspicuous, small flowers, usually white or greenish. Their proper determination is not possible without studying flower segments under stereomicroscope or lenses, as many characters are very subtle. Based on our experience we consider that the taxonomically most informative, relatively fixed and invariable features are: the numbers of veins on sepals and petals, the character of petal margins, the texture of the lip, the number and character of lip veins, the presence of nodules or glandular outgrowths on the inner lip surface, and the general lip shape.

In order to facilitate the process of species identification, several groups of species that are morphologically similar in appearance are delineated in this paper.

## Key to the Groups

1. Lip with nodules on the inner surface2
1* Lip without any nodules on the inner surface3
2. Lip suborbicular in outline, basally cochleate, gibbose, more or less 3-lobed at the apex, laterally much thickened11. *Pulvinifera* group
2* Lip suborbicular to elliptic when spread, evenly thin, or somewhat thickened in the lower half, almost flat, apex unlobed9. *Diphylla group*
3. Petals glabrous4
3* Petals ciliate, papillate, or glandular along margin/s6
4. Lip subglobose, very thick in the center, margins thinner, erose-dentate8. *Cristalinensis* group
4* Lip not as above5
5. Lip obovate, suborbicular to elliptic in general outline, rather flat, occasionally somewhat gibbose at base, veins thickened5. *Badia* group
5* Lip deeply cochleate, helmet-like, suborbicular to elliptic when spread, veins usually not thickened6. *Polyantha* group
6. Lip more or less oblong, attenuate and elongate apically10. *Wageneri* group
6* Lip usually elliptic, obovate to suborbicular7
7. Lip deeply cochleate, gibbose at base, suborbicular to elliptic when spread, veins usually not thickened7. *Polyblephara* group
7* Lip more or less obovate or suborbicular in general outline, rather flat above, veins thickened, dendritic branching8
8. Petals papillate or ciliate along margins9
8* Petals glandular, pubescent or villous along margins10
9. Petals papillate to ciliate evenly along margin/s1. *Pleioneura* group
9* Petals papillate along inner margin and ciliate to pubescent along the outer one2. *Schultesii* group
10. Petals pubescent, villous or pilose along both margins3. *Ciliata* group
10* Petals more or less glandular along both margins4. *Engelii* group

### *Pleioneura* group

Lip more or less obovate in general outline, widest near or above the middle, attenuate toward somewhat gibbose base, rather flat above, veins thickened, dendritic branching, without any nodules. Petals evenly papillate to ciliate along margin/s.

Four Colombian species belong to this group. Most probably the *Lehmann 944* collection represents an undescribed taxon.

## Key to the Species

1. Lateral sepals 1-veined2
1* Lateral sepals 3-veined3
2. Lip elliptic-ovate, gibbose*C. atrata*
2* Lip oblong elliptic, almost flat*C. sp. 1*
3. Sepals sparsely pubescent on the outside*C. mandonii*
3* Sepals glabrous4
4. Lip fleshy*C. callejasii*
4* Lip thin*C. pleioneura*

***Cranichis atrata*** Schltr., Repert. Spec. Nov. Regni Veg., Beih. **7**: 58. 1920. TYPE: Colombia. *Madero s.n*. (B†).

**Figure 3:**
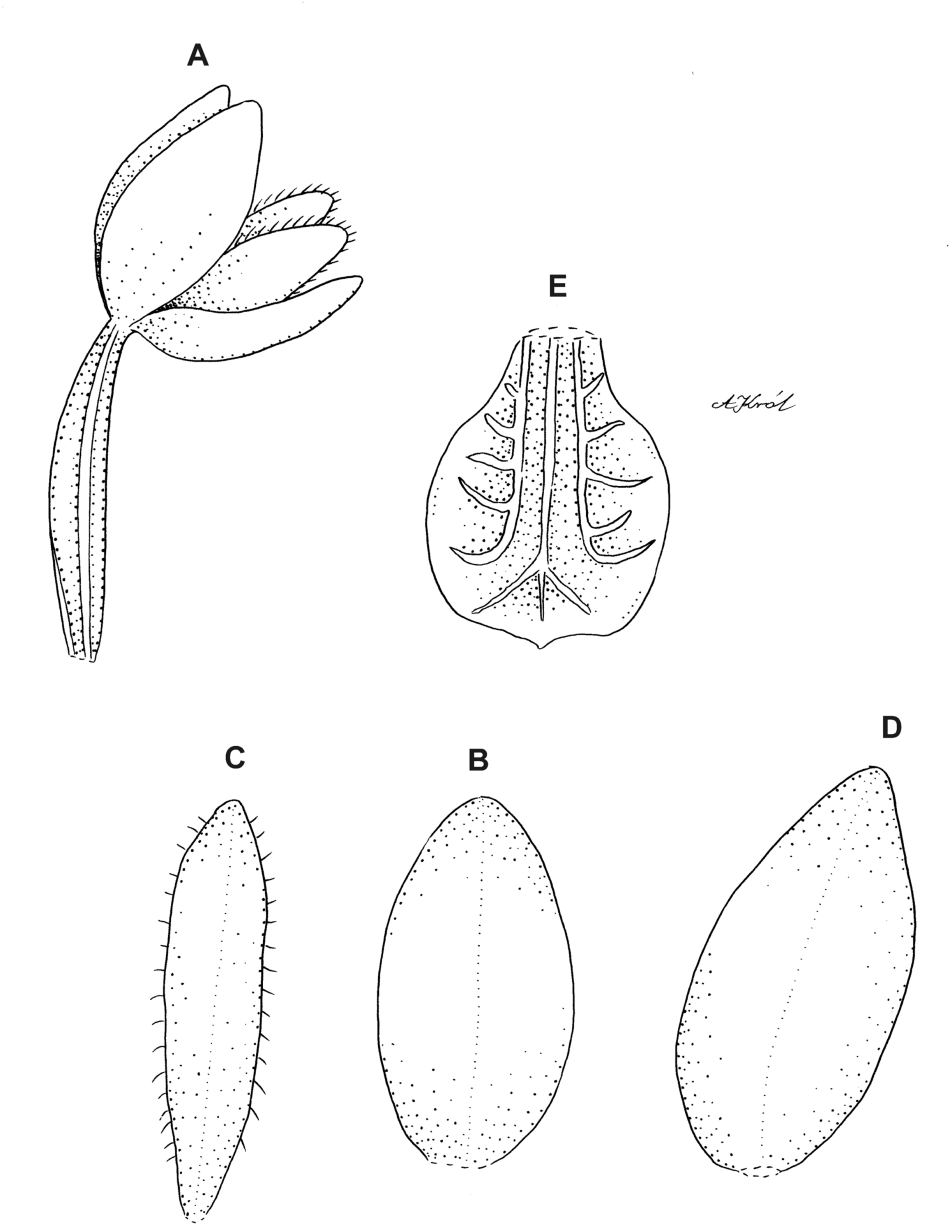
*Cranichis atrata* Schltr. (A) Flower; (B) dorsal sepal; (C) petal; (D) lateral sepal; (E) lip. Redrawn by A. Król according to the original illustration from Schlechter.

**Figure 4:**
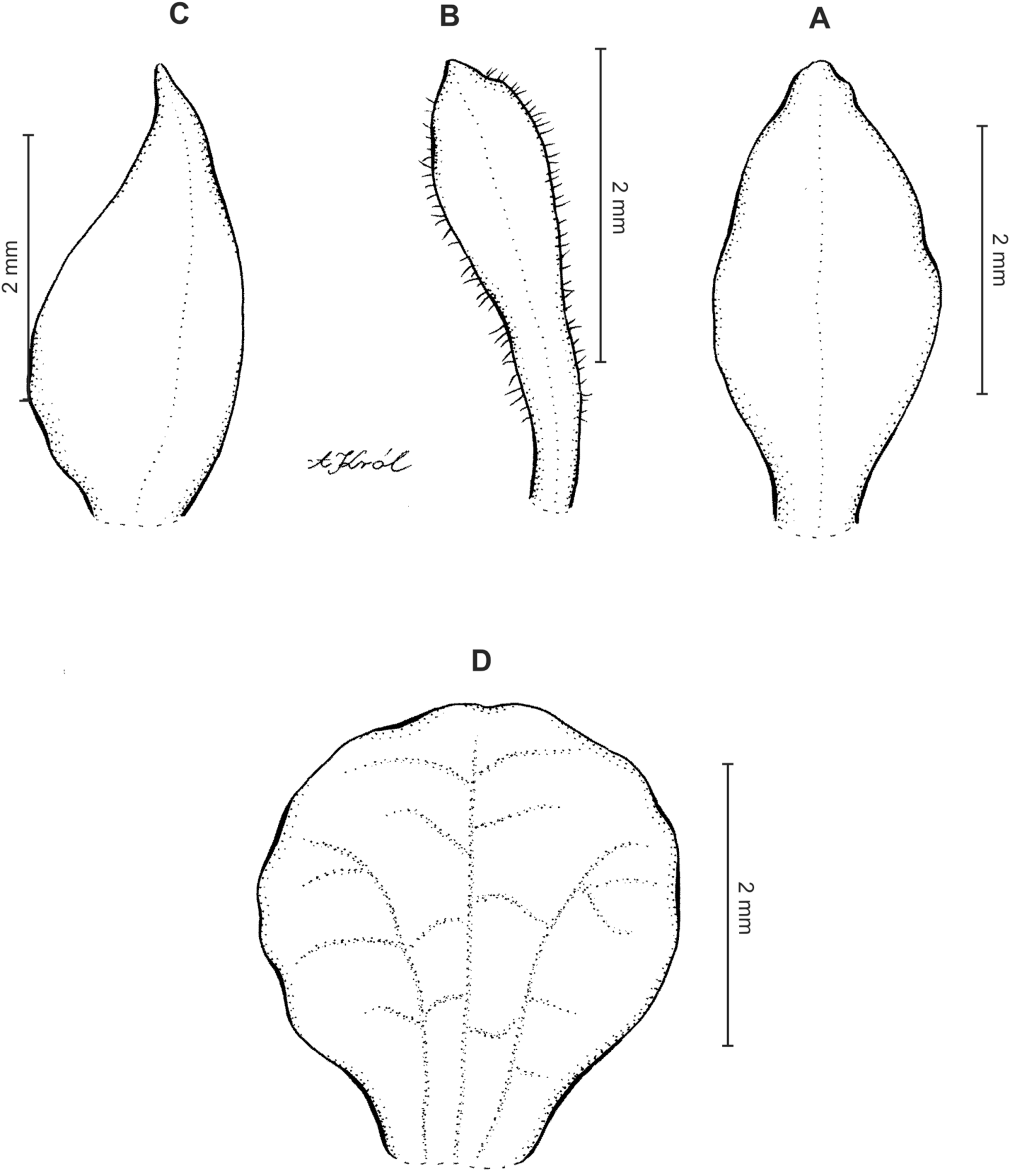
*Cranichis atrata* Schltr. (A) Dorsal sepal; (B) petal; (C) lateral sepal; (D) lip. Drawn by A. Król from *Rimann s.n*. (W-R).

Plants 30–50 cm tall, erect. Leaves 1–2, basal, petiolate; petiole 5–15 cm long, narrow, canaliculate; blade 5–7.5 cm long, 1.5–4.5 cm wide, obliquely elliptic, acuminate, base cordate. Scape delicate, enclosed in four to five sheaths, glabrous. Inflorescence two to nine cm long, subdensely few (about 7) -flowered. Flowers small, glabrous. Floral bracts seven mm long, slightly shorter than ovary, elliptic-lanceolate, acuminate. Pedicellate ovary five to eight mm long, glabrous. Sepals glabrous. Dorsal sepal 3–4.6 mm long, 1.1–2 mm wide, elliptic to elliptic-ovate, obtuse, 1-veined. Petals 2.7–4 mm long, 0.6–0.8 mm wide, obliquely oblanceolate to ligulate-oblanceolate, subacute, more or less long-ciliate along both margins, 1-veined. Lateral sepals 3.5–4.2 mm long, 1.5–2 mm wide, obliquely elliptic to elliptic-ovate, slightly concave at the base, subacuminate or subobtuse, 1-veined. Lip 2.6–3.5 mm long, 2.1–2.8 mm wide, concave, subsessile, elliptic-ovate, minutely apiculate to rounded; disc with three thickened, dendritic branching veins, without any nodules. Gynostemium 1.8 mm long (Figs. 3 and 4).

*Ecology:* Terrestrial in dense forest, along stream and on the roadside at the altitude of 2,000–2,900 m. Flowering occurs in January, March, September, and November.

*Distribution:* Colombia.

**Figure 5:**
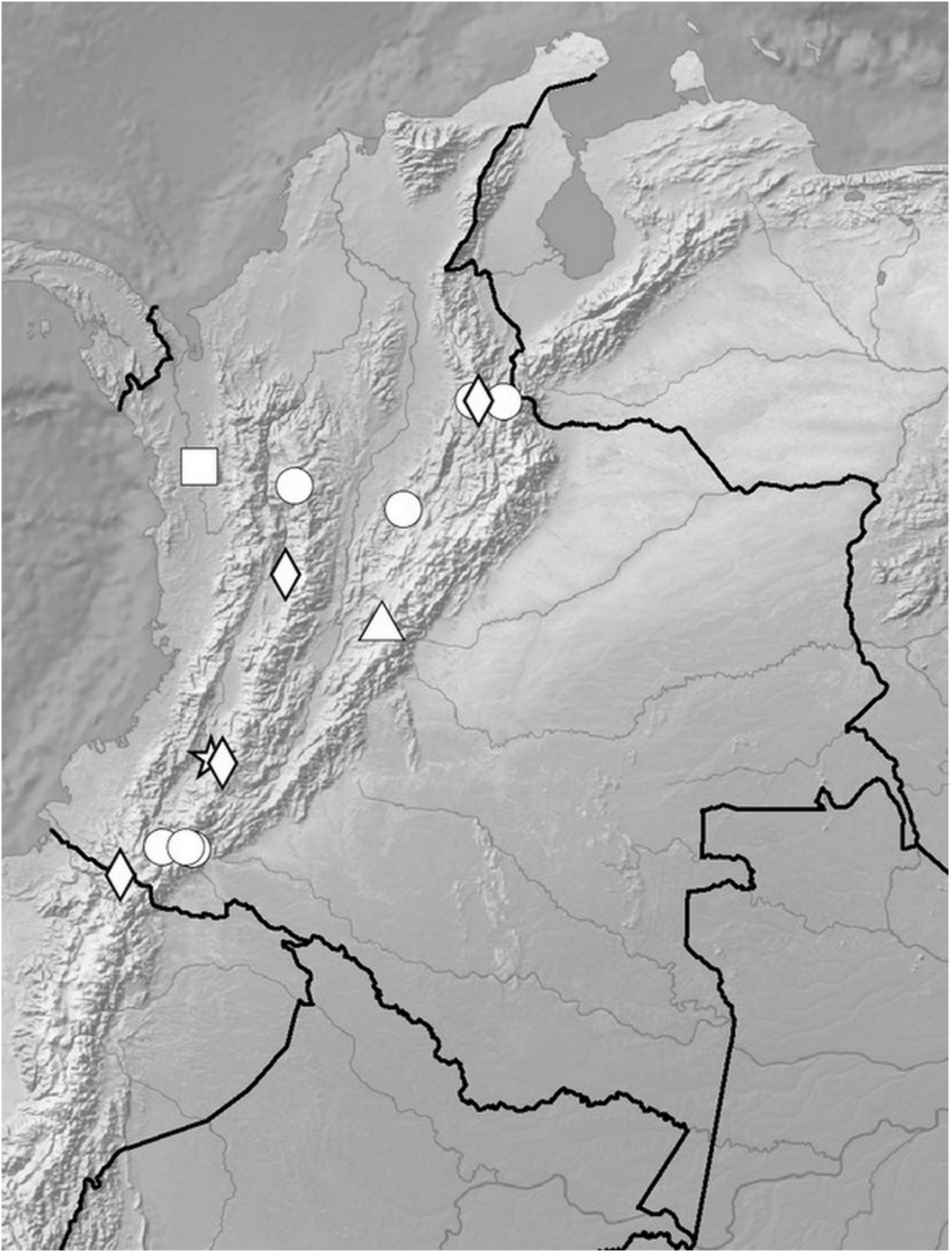
Distribution of members of the *Cranichis Pleioneura* group. *C. atrata* (circle), *C. callejasii* (square), *C. mandonii* (triangle), *C. pleioneura* (diamond), *Cranichis sp. 1* (star).

*Notes*: *Cranichis atrata*, Colombian endemic species, differs from all other taxa of this group (except *C. sp. 1*) by having 1-veined sepals, 1-veined petals with both margins being more or less long-ciliate. This species is relatively variable in lip form, although it is always gibbose, what clearly distinguishes it from *C. sp. 1*.

*Representative specimens:* COLOMBIA. **Antioquia**: Along road from Medellín to Rionegro. Dense forest along stream, 2,500 m, November 17, 1948, *E.P. Killip et al. 39868* (US!, UGDA-DLSz!—drawing). **Cauca**: *Sine loc*., *M. Madero s.n*. (B†); Pasto, *H. Rimann s.n*. (W-R!, UGDA-DLSz!—drawing). **Norte de Santander**: Parámo del Hatico, en route from Toledo to Pamplona, 2,900 m, March 12–13, 1927, *E. Killip & A.C. Smith 20643* (AMES!). **Putumayo**: Bosque alto al N del Valle de Sibundoy, hacia la mina de San Francisco, 2,800 m, January 9, 1957, *M. Ospina H. & J. Idrobo 146* (AMES!); Km 63 Pasto to Mocoa, 15 km beyond San Francisco, 2,500 m, January 22, 1987, *C.H. Dodson et al. 17035* (RPSC!, UGDA-DLSz!—drawing). **Santander**: Valley of Río Minero, near Florian. About six km E of La Belleza, 2,400 m, September 26, 1944, *N. Fassett 25804* (AMES!, UGDA-DLSz!—drawing); Río Suratá valley, above Suratá, 2,000–2,300 m, January 5–6, 1927, *E.P. Killip & A.C. Smith 16730* (AMES!, US!, UGDA-DLSz!—drawing) (Fig. 5).

Garay (1978) synonymized this species under *C. ciliata*. Both species differs in petals ornamentation (long-ciliate vs. pilose), lateral sepals venation (1-veined vs. 2-veined) and lip form (subsessile, elliptic-ovate vs. shortly unguiculate, obovate).

Like *C. nigrescens* Schltr. this species is black when dried.

Garay (1978) designated as lectotype of *C. atrata* drawing of *Madero 3* collection made under Schlechter’s supervision. Schlechter (1920), however, mentioned in the protologue *Madero s.n*.

***Cranichis callejasii*** Szlach. & Kolan., ***sp. nov***. TYPE: Colombia. *Callejas et al. 7757* (holotype, MO!; isotype, NY!, UGDA-DLSz!—drawing).
*Species similar to C. werffii, distinguished by petals being ciliolate in the upper half and glabrous ovary. It can be easily distinguished from C. pleioneura by having fleshy lip (vs. thin lip)*.

**Figure 6:**
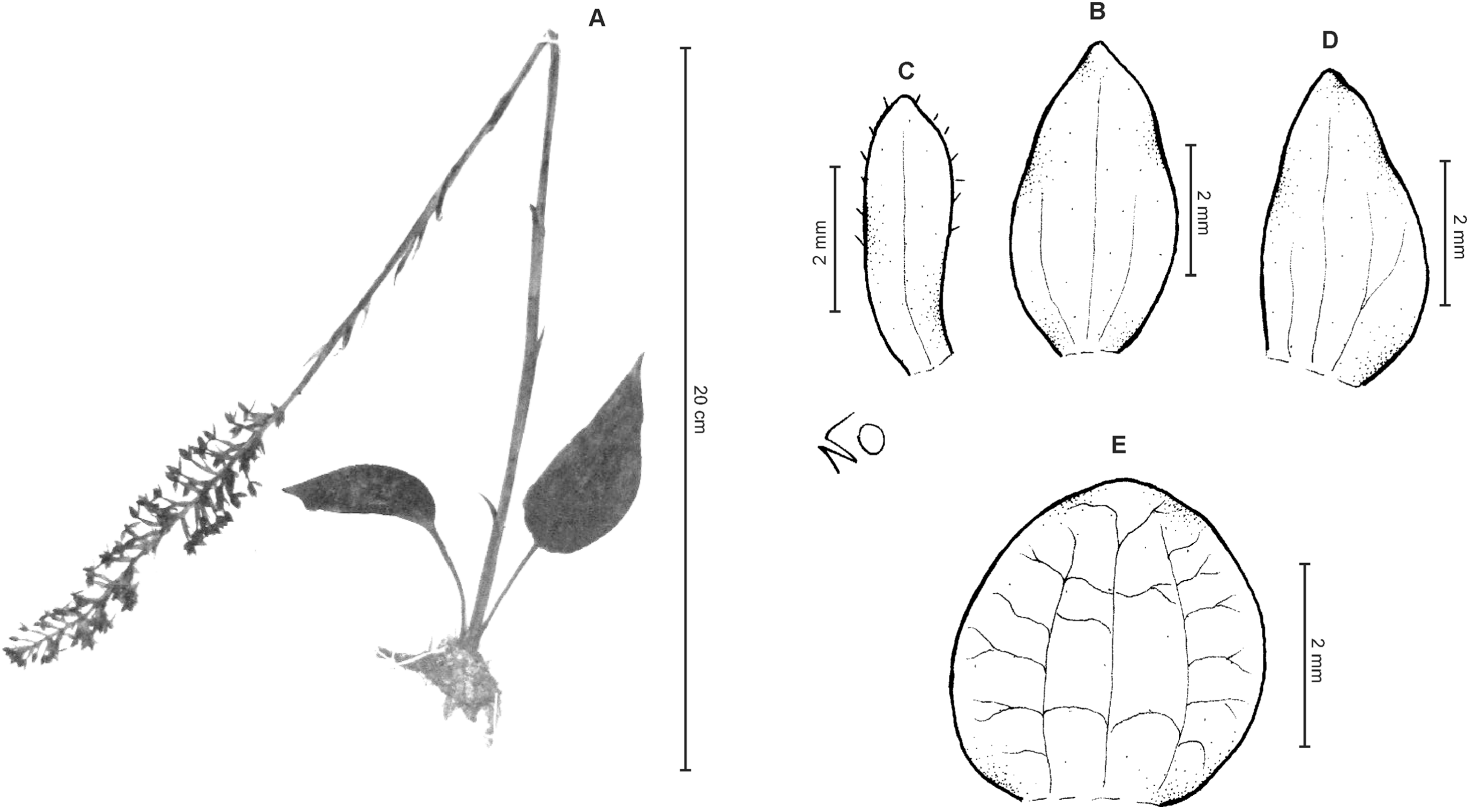
*Cranichis callejasii* Szlach. & Kolan. (A) Habit; (B) dorsal sepal; (C) petal; (D) lateral sepal; (E) lip. Drawn by N. Olędrzyńska from *Callejas et al. 7757* (MO).

Plants about 41 cm tall. Leaves 2, basal, petiolate; petiole 3.5 cm long, narrow, canaliculate; blade up to six cm long, 2.6 cm wide, ovate, acute, greenish-purple. Scape erect, glandular in the upper part, enclosed in 11 sheaths. Inflorescence 11 cm long, cylindrical, rather densely many-flowered. Flowers white, glabrous. Floral bracts up to 6.5 mm long. Pedicellate ovary up to seven mm long. Dorsal sepal 4.8 mm long, 1.9 mm wide, ovate, obtuse, obscurely 3-veined. Petals 3.8 mm long, one mm wide, ligulate-falcate, obtuse, ciliolate in the upper half, 1-veined. Lateral sepals 4.1 mm long, 2.6 mm wide, obliquely ovate, obtuse, obscurely 3-veined, lateral vein dichotomous. Lip 3.6 mm long, three mm wide, fleshy, sessile, suborbicular, obtuse; disc with three anastomozing, thickened veins. Gynostemium 1.5 mm long (Fig. 6).

*Etymology*: Named in honor of the senior collector of the type specimen.

*Ecology*: Terrestrial plant growing at an altitude of 3,120 m. Flowering occurs in April.

*Distribution*: Colombia.

*Representative specimens*: COLOMBIA. **Antioquia**: Mpio. Urrao. Corregimiento Encarnacion. Sitio El Rio, 1 hora de camino del Páramo de Frontino. 6°27′N 76°46′W, 3,120 m, April 7, 1989, *R. Callejas et al. 7757* (MO!, NY!, UGDA-DLSz!—drawing) (Fig. 5).

*Notes*: *C. callejasii* is similar to its Ecuadorean congener, *C. werffii*, but can be easily distinguished from the latter by having petals ciliolate in the upper half. Petals of *C. werffii* are sparsely ciliolate along the anterior margin, and glabrous along posterior one. The ovary of *C. werffii* is sparsely glandular-pubescent, whereas in *C. callejasii*—glabrous. From *C. pleioneura* the new species differs in petals being ciliolate in the upper half (vs. ciliate along both margins) and fleshy (vs. thin), suborbicular (vs. suborbicular-obovate) lip.

***Cranichis mandonii*** Schltr., Repert. Spec. Nov. Regni Veg. Beih. 10: 38. 1922. TYPE (here designated): Bolivia. *Mandon 1163* (lectotype, AMES!; isolectotypes, BM, G, GH, NY, P, S).

**Figure 7:**
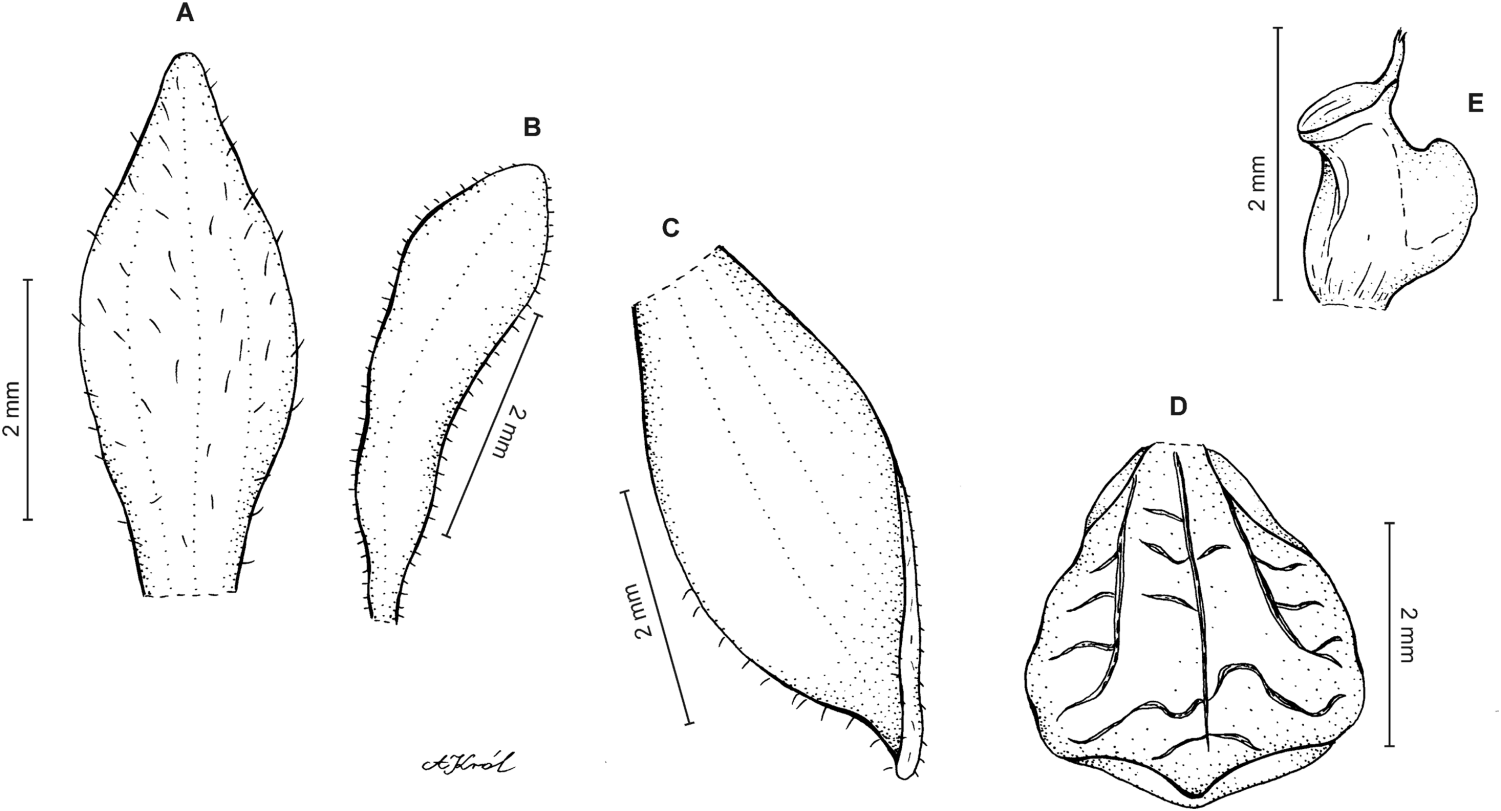
*Cranichis mandonii* Schltr. (A) Dorsal sepal; (B) petal; (C) lateral sepal; (D) lip; (E) gynostemium. Redrawn by A. Król from Garay’s illustration of specimen collected by *Mandon 1163* (AMES).

Plants 40–60 cm tall. Leaves 1–2, basal, petiolate; petiole 13–16 cm long, narrow, canaliculate; blade 6–8.5 cm long, 3.7–4.4 cm wide, oblong to ovate, slightly oblique, base cordate. Scape delicate, terete, enclosed in five to six acuminate sheaths, apically glandular-pilose. Inflorescence 12 cm long, cylindrical, subdensely many-flowered. Flowers with tepals maroon at the base, white at the apex. Floral bracts about 5–5.5 mm long, narrowly lanceolate, acuminate. Pedicellate ovary about 5–5.5 mm long, fusiform-cylindrical, sparsely glandular. Sepals sparsely pubescent on the outer surface. Dorsal sepal 4.5 mm long, 1.5 mm wide, lanceolate-ovate to oblong ovate, subacute to subobtuse, 3-veined. Petals four mm long, one mm wide, oblong ligulate to oblong oblanceolate, obtuse to rounded at the apex, 1-veined, margins minutely ciliate-papillate. Lateral sepals 4.5 mm long, two mm wide, elliptic to elliptic-ovate, subacute, 3-veined. Lip three mm long and wide, basally gibbose, subsessile, suborbicular-obovate to suborbicular, apically rounded; disc 3-veined, veins somewhat thickened, dendritic branching, without any nodules. Gynostemium two mm long (Fig. 7).

*Ecology*: In Colombia this species was found growing terrestrially in a *Cupressus* plantation at an altitude of 3,000–3,200 m. Flowering occurs in November.

*Distribution*: Colombia, Bolivia.

*Representative specimens:* COLOMBIA. **Cundinamarca**: Bogotá. Usme, sector sel Embalse de Chisacá. Vereda Las Margaritas, 3,000–3,200 m., 4°20′N 74°15′W, November 21, 2005, *Y. Figueroa et al. 880* (COL!, UGDA!—drawing) (Fig. 5).

*Other materials examined*: BOLIVIA. **Cochabamba**: Carrasco. La Siberia, January 1983, *R. Vásquez et al. 792* (Herbarium Vasquezianum—Dodson & Vásquez, 1989). **Larecaja**: Sorata, 2,650–3,100 m, Apr–May 1860, *G. Mandon 1163* (AMES!, BM, G, GH, NY, P, S).

*Notes*: *C. mandonii* can be easily misidentified as *C. pleioneura* from which it can be distinguished by having sparsely glandular (vs. glabrous) ovary, sparsely pubescent (vs. glabrous) sepals, and minutely ciliate-papillate (vs. ciliate) margins of petals.

*Cranichis mandonii* can be also confused with *C. ciliata* but the former has sparsely glandular ovary (vs. glabrous), 3-veined dorsal and lateral sepals (vs. 1-veined and 2-veined, respectively), and rounded lip apex (vs. rounded and slightly notched).

According to the original description of *C. mandonii* by Schlechter the sepals are glabrous. We confirm, however, Garay’s observation (cf. his illustration kept at AMES) of the presence of sparse pubescence on sepals.

Until now this species was reported exclusively from Bolivia; however, in our opinion, the collection of *Figueros et al. 880* (COL) also represents this species.

***Cranichis pleioneura*** Schltr., Repert. Spec. Nov. Regni Veg., Beih. 7: 60. 1920; TYPE: Colombia. *Madero s.n*. (B†;).

**Figure 8:**
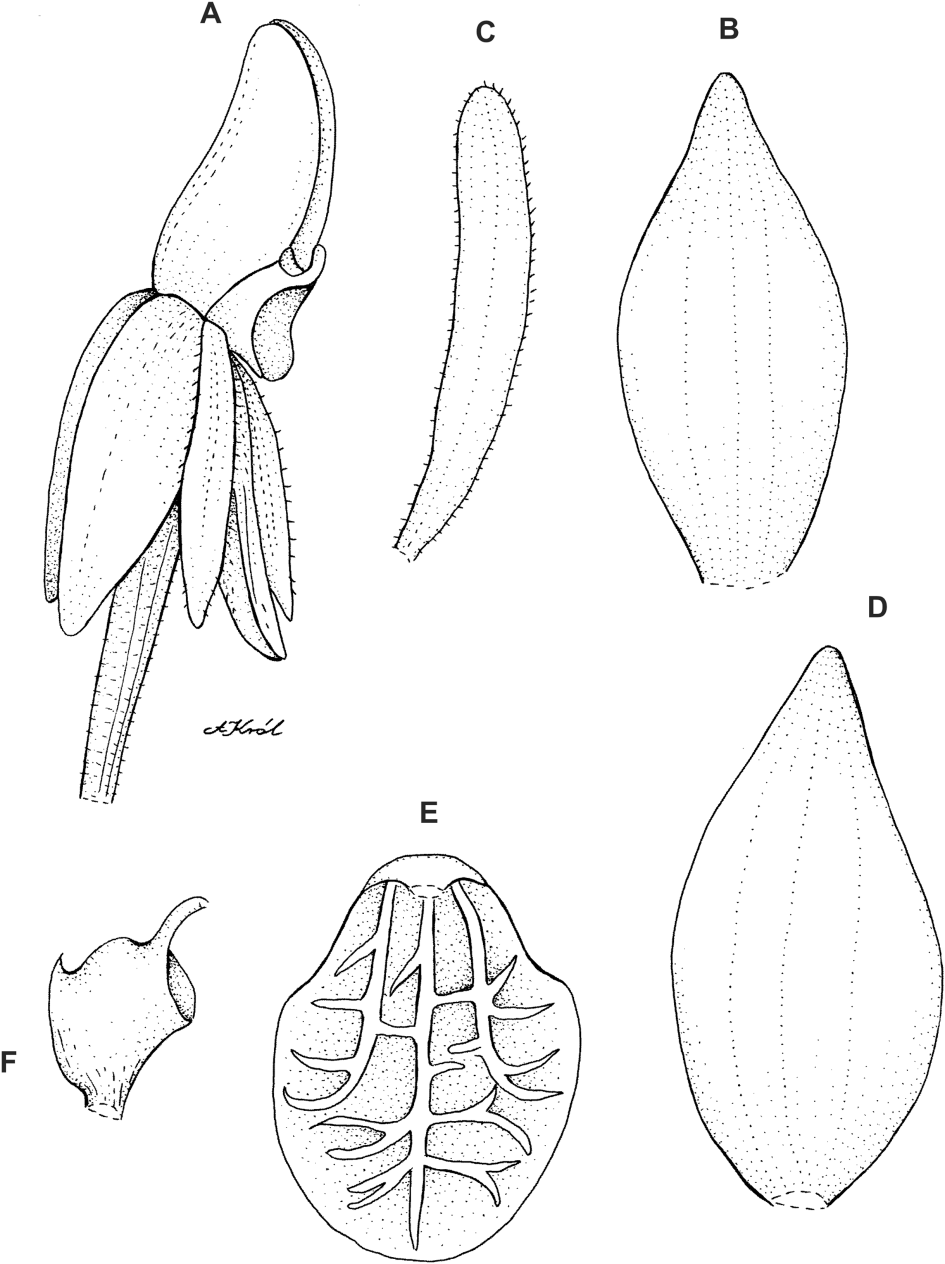
*Cranichis pleioneura* Schltr. (A) Flower; (B) dorsal sepal; (C) petal; (D) lateral sepal; (E) lip; (F) gynostemium. Redrawn by A. Król according to the original illustration from Schlechter.

**Figure 9:**
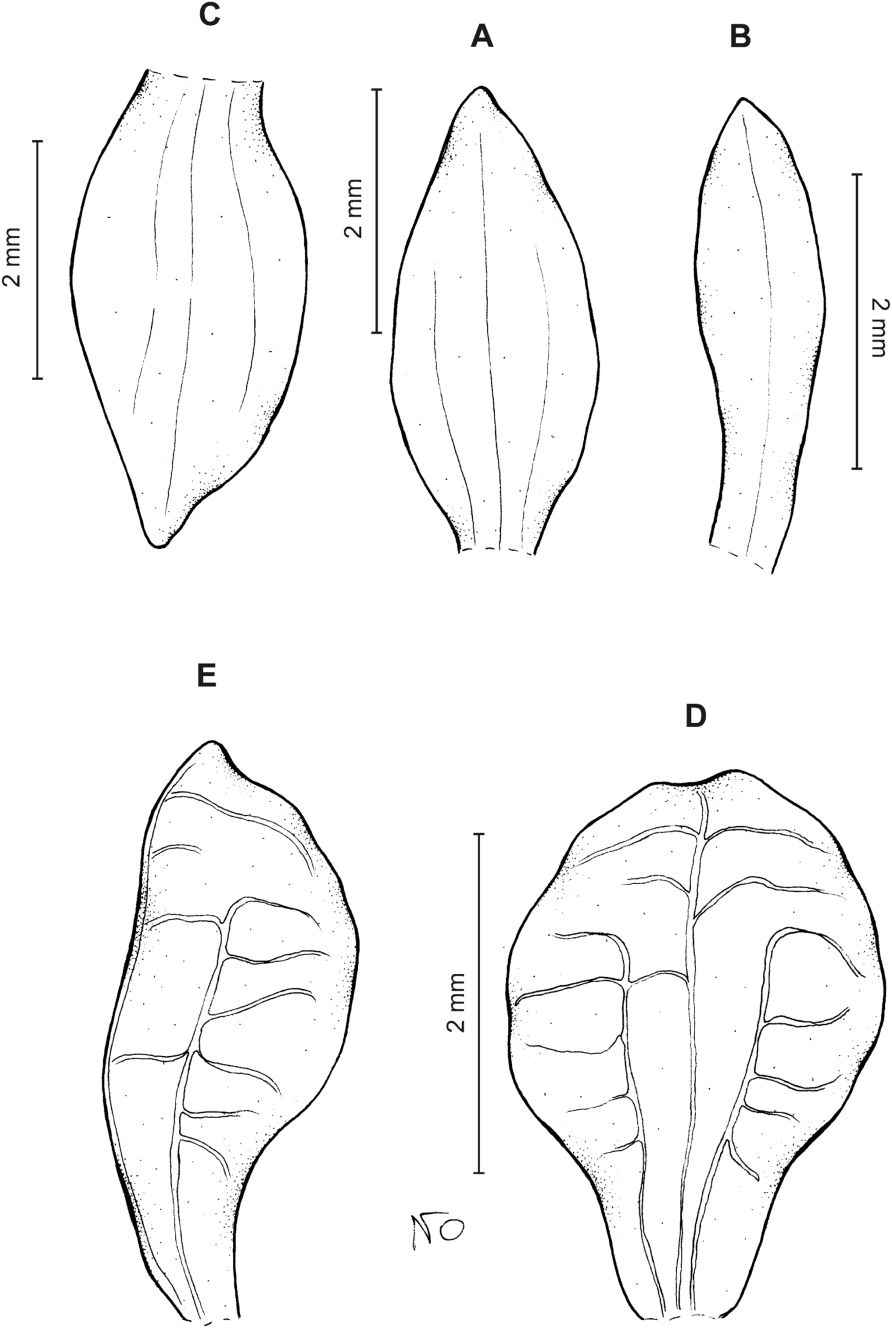
*Cranichis pleioneura* Schltr. (A) Dorsal sepal; (B) petal; (C) lateral sepal; (D) lip (front view); (E) lip (side view). Drawn by N. Olędrzyńska from *Pennell 9400* (NY).

Plants 30–60 cm tall, erect. Leaf 1, basal, petiolate; petiole 8–15 cm long, narrow, canaliculate; blade 7.5–15 cm long, 3.5–7.5 cm wide, oblong-ovate, acute to acuminate. Scape delicate, terete, enclosed in three to five sheaths, glandular toward the apex. Inflorescence 5–12 cm long, cylindric, densely many-flowered. Flowers small, glabrous. Floral bracts 4.5–5 mm long, slightly longer than ovary, lanceolate, acuminate, glabrous. Pedicellate ovary five to nine mm long, glabrous. Dorsal sepal 3–3.8 mm long, 1.1–1.5 mm wide, oblong-elliptic to elliptic-ovate, subobtuse, 3-veined. Petals 2.8–3.2 mm long, 0.4–0.8 mm wide, obliquely linear-oblanceolate to linear-ligulate, obtuse to truncate at apex, ciliate along both margins, 1-veined. Lateral sepals three to four mm long, 1.3–2 mm wide, obliquely ovate to elliptic-ovate, slightly concave at the base, subacuminate, subobtuse, 3-veined. Lip 2.5–3.2 mm long, 2–2.6 mm wide, concave in the lower part, subsessile, suborbicular-obovate, apex rounded; disc thin, with three thickened, dendritic branching veins, without any nodules. Gynostemium 1.3–1.8 mm long (Figs. 8 and 9).

*Ecology*: Terrestrial plant growing on the edge of dense forest and in paramo at an altitude of 2,500–3,600 m. Flowering occurs in January, July, and August.

*Distribution*: Colombia.

*Representative specimens:* COLOMBIA. **Caldas**: Pinares. Above Salento. Cordillera Central. Edge of forest, 2,700–2,900 m, August 2–10, 1922, *F.W. Pennell 9400* (AMES!, NY!, US!, UGDA-DLSz!—drawing). **Cauca**: *Sine loc*., 2,500 m, *M. Madero s.n*. (B†); Volcán de Puracé, páramo de San Rafael, 3,300 m, July 23, 1960, *L.A. Garay 15* (AMES!, UGDA-DLSz!—drawing). **Nariño**: Cordillera Central. Río Chiles, across river from Tufiño, 3,600 m, August 17, 1944, *W. Drew E-482* (AMES!, UGDA-DLSz!—drawing). **Santander**: Quebrada de Pais, N of La Baja. Dense forest, 3,200 m, January 31, 1927, *E.P. Killip & A.C. Smith 18790* (AMES!, NY!, US!) (Fig. 5).

*Notes*: This species shares with *C. mandonii* and *C. werffii* the same number of veins on sepals and petals, i.e., three and one, respectively. Unlike the former, however, *C. pleioneura* has glabrous sepals and ovary (vs. sparsely glandular); the petals of the latter are ciliate along both margins (vs. sparsely ciliolate along anterior margin, glabrous along posterior one). *Garay 15* (AMES) collection is characterized by having sparsely ciliate petal margins. Otherwise, the plant fits well the description of *C. pleioneura*. This species can be distinguised from *C. callejasii* described above by the lip texture (thin vs. fleshy).

*Cranichis pleioneura* can be easily misidentified as the widely distributed *C. ciliata* from which it can be distinguished by its 3-veined sepals (vs. 1-veined dorsal sepal, 2-veined lateral sepals), and petals are ciliate along both margins (vs. margins of petals unevenly pilose). Additionally, the lip of *C. pleioneura* is subsessile, suborbicular-obovate in outline with rounded apex (vs. shortly unguiculate, obovate in outline, rounded and slightly notched at apex) and the scape is glandular (vs. glandular-pubescent). Further studies should be able to reveal the taxonomic status of both taxa.

The drawing of *Cranichis pleioneura* made under Schlechter’s supervision and stored at AMES bears *Madero 92* and information that the plant was collected in Antioquia, 2,500 m a.s.l. Schlechter (1920), however, mentioned as *Madero s.n*. in the protologue.

### *Cranichis* sp. 1

**Figure 10:**
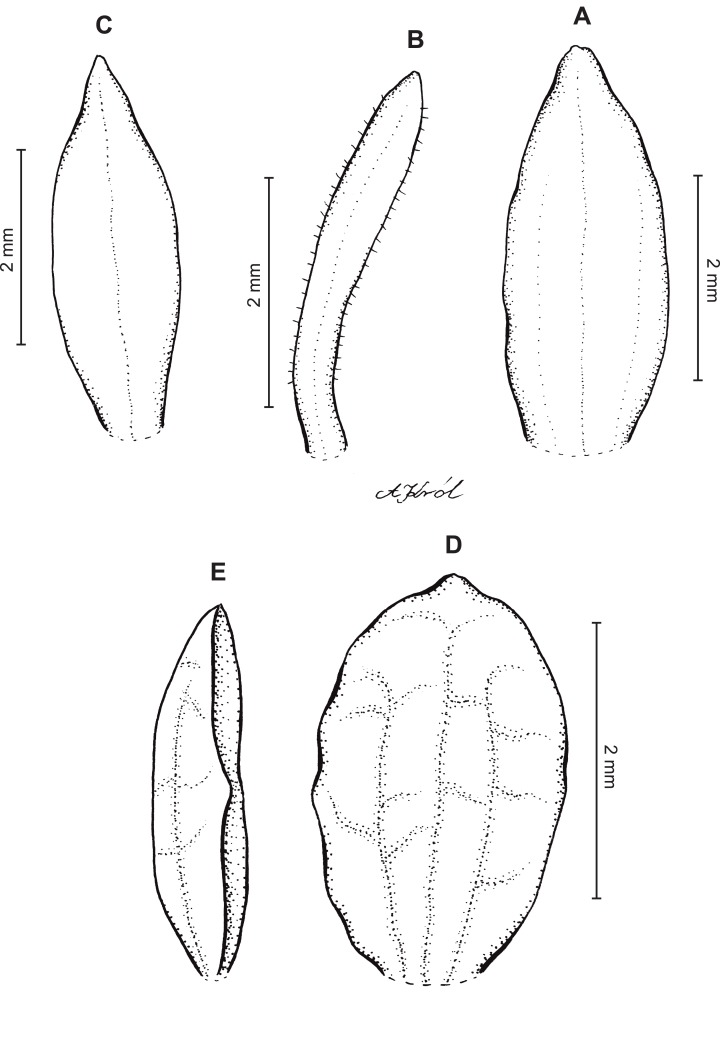
*Cranichis sp. 1*. (A) Dorsal sepal; (B) petal; (C) lateral sepal; (D) lip (front view); (E) lip (side view). Drawn by A. Król from *Lehmann 944* (W-R).

Floral bract five mm long, glabrous. Pedicellate ovary eight mm long, glabrous. Dorsal sepal four mm long, 1.5 mm wide, oblong elliptic, subobtuse, 3-veined. Petals 3.5 mm long, 0.4 mm wide, linear-oblanceolate, 1-veined, ciliolate along both margins. Lateral sepals four mm long, one mm wide, obliquely elliptic, obtuse, 1-veined. Lip three mm long, 1.7 mm wide, almost flat, sessile, oblong-elliptic, apiculate, obtuse; disc with three thickened, branching veins. Gynostemium 1.5 mm long (Fig. 10).

*Representative specimen:* COLOMBIA. **Cauca**: Cordillera Central uber Popayán, September 2, 1881, *F.C. Lehmann 944* (W-R!, UGDA-DLSz!—drawing) (Fig. 5).

*Notes*: This specimen is characterized by almost flat, oblong elliptic lip, linear petals, sparsely ciliolate along both margins. Both these characters distinguish it from all other species of this group.

### *Schultesii* group

Lip more or less suborbicular to obovate in general outline, widest near or above the middle, somewhat gibbose at the base, then rather flat, veins thickened, dendritic branching, without any nodules. Petals papillate along inner margin and ciliate to pubescent along the outer one.

## Key to the Species

1. Lateral sepals 3-veined*C. schultesii*
1* Lateral sepals 1- or 2-veined*C. roldanii*

***Cranichis roldanii*** Szlach. & Kolan., ***sp. nov***. TYPE: Colombia. *Betancur et al. 1042* (holotype, MO!; UGDA-DLSz!—drawing).
*Species distinguished from C. pleioneura by petals being papillate along the inner margin, long ciliate along the outer margin, 1-veined sepals and fleshy, suborbicular lip. It can be distinguished from C. schultesii by its 1- or 2-veined lateral sepals and obtuse lip*.

**Figure 11:**
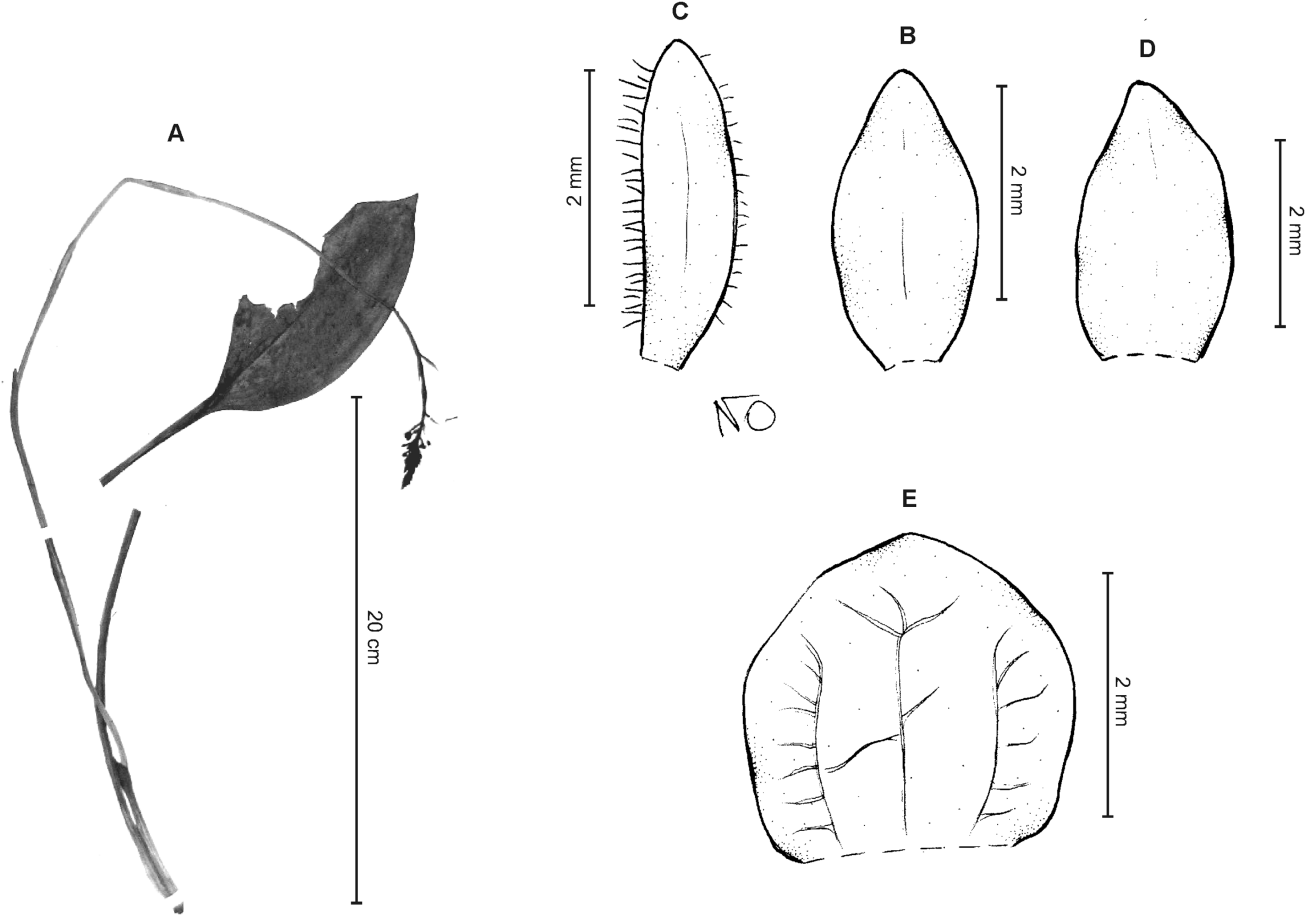
*Cranichis roldanii* Szlach. & Kolan. (A) Habit; (B) dorsal sepal; (C) petal; (D) lateral sepal; (E) lip (front view). Drawn by N. Olędrzyńska from *Betancur et al. 1042* (MO).

Plant about 48 cm tall. Leaves 2, basal, petiolate; petiole 21 cm long; blade up to 11 cm long, five cm wide, elliptic, shortly acuminate. Scape delicate, densely glandular, enclosed in about seven sheaths. Inflorescence four cm long, conical, subdensely several-flowered. Flowers greenish, glabrous. Floral bracts up to 5.5 mm long. Pedicellate ovary up to 5.5 mm long. Dorsal sepal 2.8 mm long, 1.4 mm wide, elliptic, rounded at the apex, 1-veined. Petals 2.8 mm long, 0.8 mm wide, ligulate, obtuse, papillate along the inner margin, long ciliate along the outer margin, 1-veined. Lateral sepals three mm long, 1.3 mm wide, ovate, apex rounded, 1-veined. Lip fleshy, somewhat gibbose at the base, sessile, 2.6 mm long, 2.9 mm wide, suborbicular, obtuse; disc with three thickened, dendritic branching veins. Gynostemium 1.8 mm long (Fig. 11).

*Etymology*: Dedicated to F. Roldán, co-collector of type specimen.

*Ecology*: Terrestrial plant growing in cloud forest at an altitude of about 2,140 m. Flowering occurs in October.

*Distribution*: Colombia.

**Figure 12:**
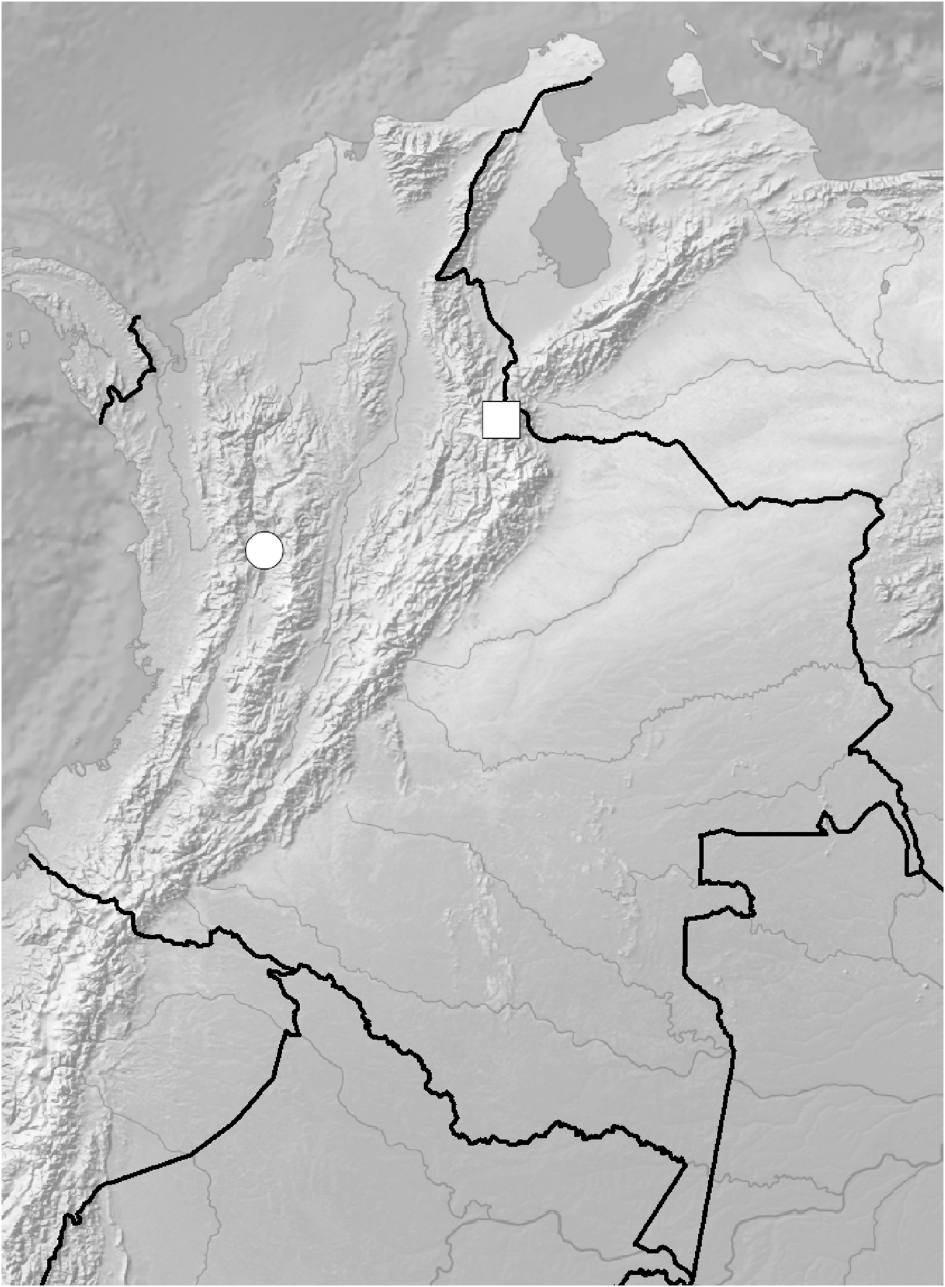
Distribution of members of the *Cranichis Schultesii* group. *C. roldanii* (circle), *C. schultesii* (square).

*Notes*: This species is characterized by somewhat fleshy, orbicular lip, petals papillate along the inner margin, and long ciliate along the outer one. From *C. pleioneura* the new species differs in having two basal leaves (vs. single leaf), petals papillate along the inner margin, long ciliate along the outer margin (vs. ciliate along both margins), 1-veined sepals (vs. 3-veined) and fleshy (vs. thin), suborbicular (vs. suborbicular-obovate) lip. *C. roldanii* resembles *C. schultesii* from which it differs by having 1- or 2-veined lateral sepals, petals papillate along the inner margin, long ciliate along the outer one and obtuse lip.

*Representative specimens*: COLOMBIA. **Antioquia**: Mpio. Caramanta. Vereda Hojas Anchas. Cerro Viringo, 9.8 km de Caramanta hacia Supia, Cordillera Occidental, bosque primario poco intervenido y nublado, 5°31.8′N 75°40.69′W, 2,140 m, October 15, 1988, *J. Betancur et al. 1042* (MO!, UGDA-DLSz!—drawing) (Fig. 12).

***Cranichis schultesii*** Szlach. & Kolan., ***sp. nov***. TYPE: Colombia. *Cuatrecasas et al. 12169a* (holotype, AMES!; isotype, AMES!; UGDA-DLSz!—drawing).
*Species distinguished from C. roldanii based on 3-veined sepals, petals ciliate along the upper part of the outer margin, and apiculate lip*.

**Figure 13:**
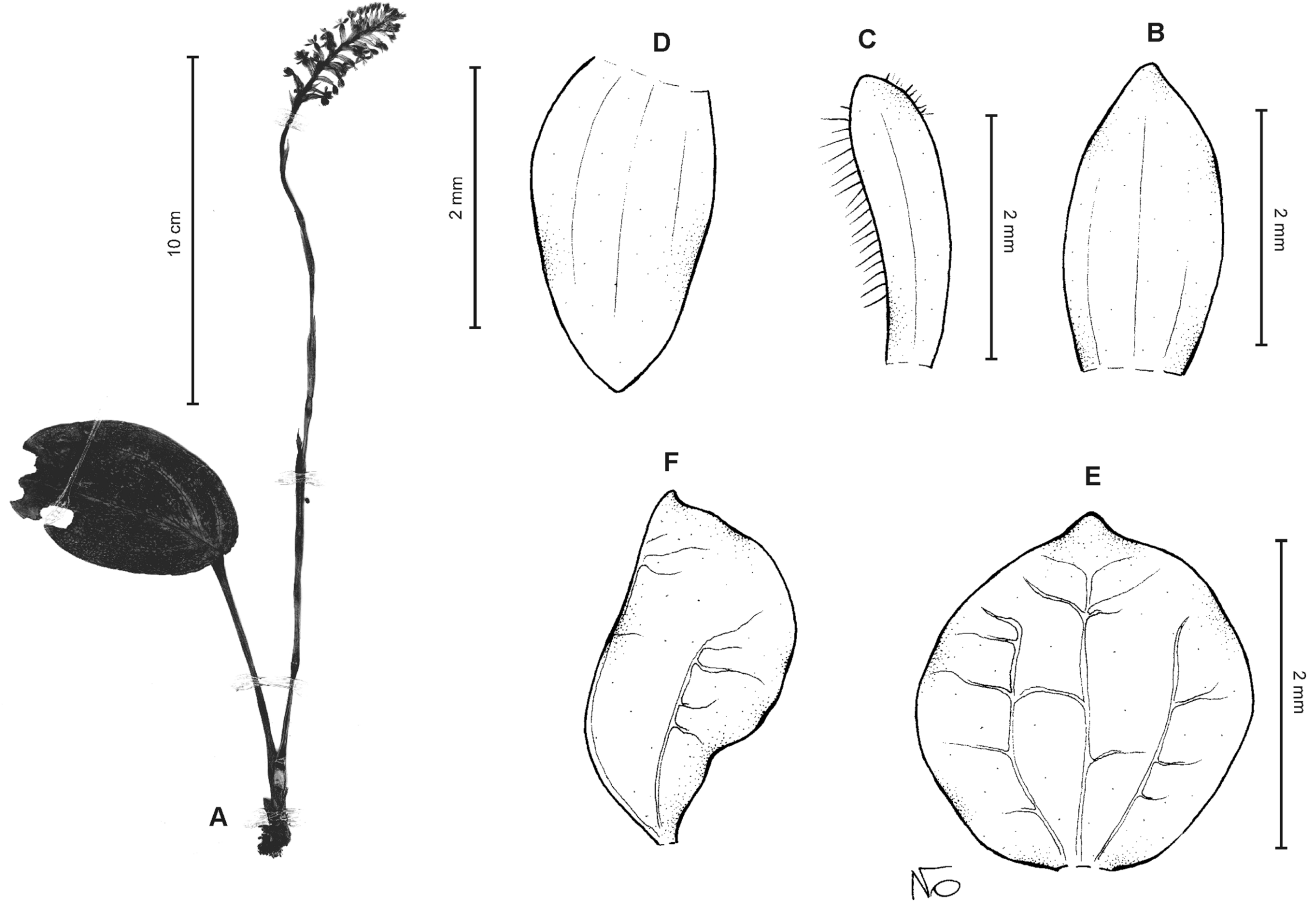
*Cranichis schultesii* Szlach. & Kolan. (A) Habit; (B) dorsal sepal; (C) petal; (D) lateral sepal; (E) lip (front view); (F) lip (side view). Scale bar for (E–F) = 2 mm. Drawn by N. Olędrzyńska from *Cuatrecasas et al. 12169a* (AMES).

Plant up to about 25 cm long. Leaves 1–2, basal, petiolate; petiole up to 7.5 cm long; blade up to seven cm long, 4.2 cm wide, ovate, acute, the inner leaf smaller, ovate. Scape erect, sparsely glandular below the rachis, enclosed in five to six sheaths. Inflorescence 4.5 cm long, cylindric, subdensely many-flowered. Flowers white, glabrous. Floral bracts up to 5.5 mm long. Pedicellate ovary up to 6.5 mm long. Dorsal sepal 2.6 mm long, 1.2 mm wide, elliptic, obtuse, 3-veined. Petals 2.4 mm long, 0.8 mm wide, ligulate, obtuse, papillate along the inner margin, ciliate along the upper part of the outer margin, 1-veined. Lateral sepals 2.6 mm long, 1.3 mm wide, obliquely ovate, obtuse, 3-veined. Lip somewhat fleshy and gibbose at the base, subsessile, 2.2 mm long and wide, suborbicular, apiculate, obtuse; disc with three thickened, dendritic branching veins. Gynostemium 1.3 mm long (Fig. 13).

*Etymology*: Named in honor of the co-collector of the type specimen.

*Ecology*: Probably terrestrial plant growing at an altutide of 1,600–1,800 m. Flowering occurs in October.

*Distribution*: Colombia.

*Representative specimens*: COLOMBIA. **Norte de Santander**: Cordillera Oriental. Región de Sarare. Hoya del Río Chitaga, sobre La Cabuya, 1,600–1,800 m, October 13, 1941, *J. Cuatrecasas et al. 12169a* (AMES!, UGDA-DLSz!—drawing) (Fig. 12).

*Notes*: The new entity can be distinguished from *C. roldanii* by its 3-veined sepals (vs. 1-veined), petals ciliate along the upper part of the outer margin (vs. entire outer margin long-ciliate), and apiculate (vs. non-apiculate) lip.

Because four plants are attached to a single herbarium sheet, we have selected the one from the upper-left corner to serve as a holotype.

### *Ciliata* group

Lip more or less suborbicular-obovate or elliptic in general outline, widest near or just above the middle, rather flat, occasionally gibbose at base, veins thickened, dendritic branching, without any nodules. Petals pubescent, villous, or pilose along both margins.

Five Colombian species belong to this group.

## Key to the Species

1. Petals with glabrous base and apex2
1* Petals not as above3
2. Petals densely and softly pubescent, lip elliptic-ovate in outline, widest just below the middle, apex truncate with short, triangular, acute apiculus, margins somewhat wavy at base*C. zarucchii*
2* Petals densely and softly pubescent-papillate, lip elliptic-obovate in outline, widest near the middle, apex truncate to rounded*C. antioquiensis*
3. Lateral sepals 1-veined*C. barkleyi*
3* Lateral sepals 2- or 3-veined4
4. Flowers white, petals distinctly narrower in the basal fourth, lip rounded to obtuse at apex*C. killipii*
4* Flowers white, marked with green or purple-brown, petals ligulate, lip rounded and slightly notched at apex*C. ciliata*

***Cranichis antioquiensis*** Schltr., Repert. Spec. Nov. Regni Veg., Beih. 7: 57. 1920. TYPE: Colombia. *Madero 168* (B†; lectotype, designated by Garay (1978: 189) AMES!—drawing).

**Figure 14:**
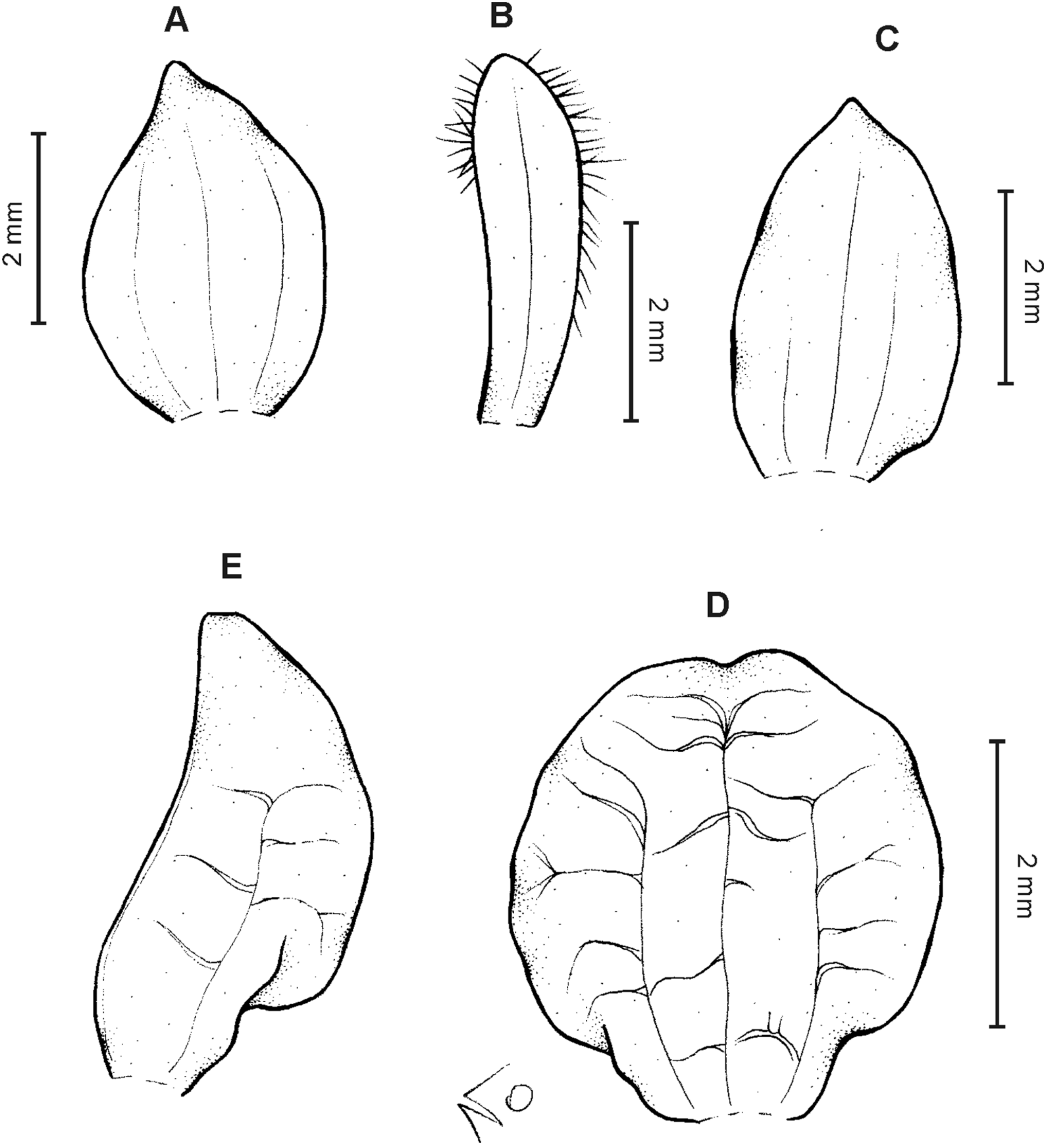
*Cranichis antioquiensis* Schltr. (A) Dorsal sepal; (B) petal; (C) lateral sepal; (D) lip (front view); (E) lip (side view). Drawn by N. Olędrzyńska from *Dodson et al. 18774* (RPSC).

**Figure 15:**
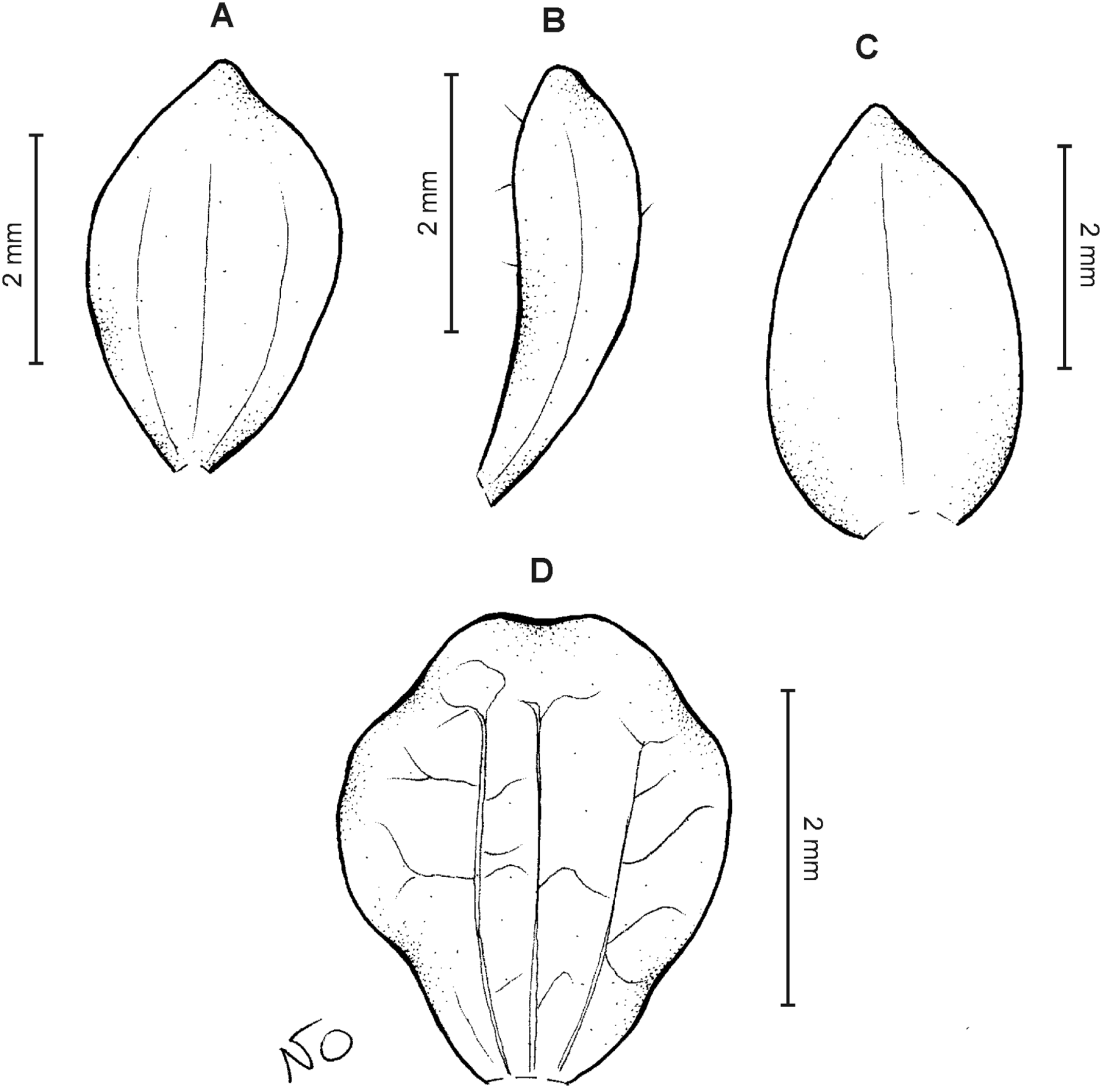
*Cranichis antioquiensis* Schltr. (A) Dorsal sepal; (B) petal; (C) lateral sepal; (D) lip (front view). Drawn by N. Olędrzyńska from *Schneider 172* (AMES).

**Figure 16:**
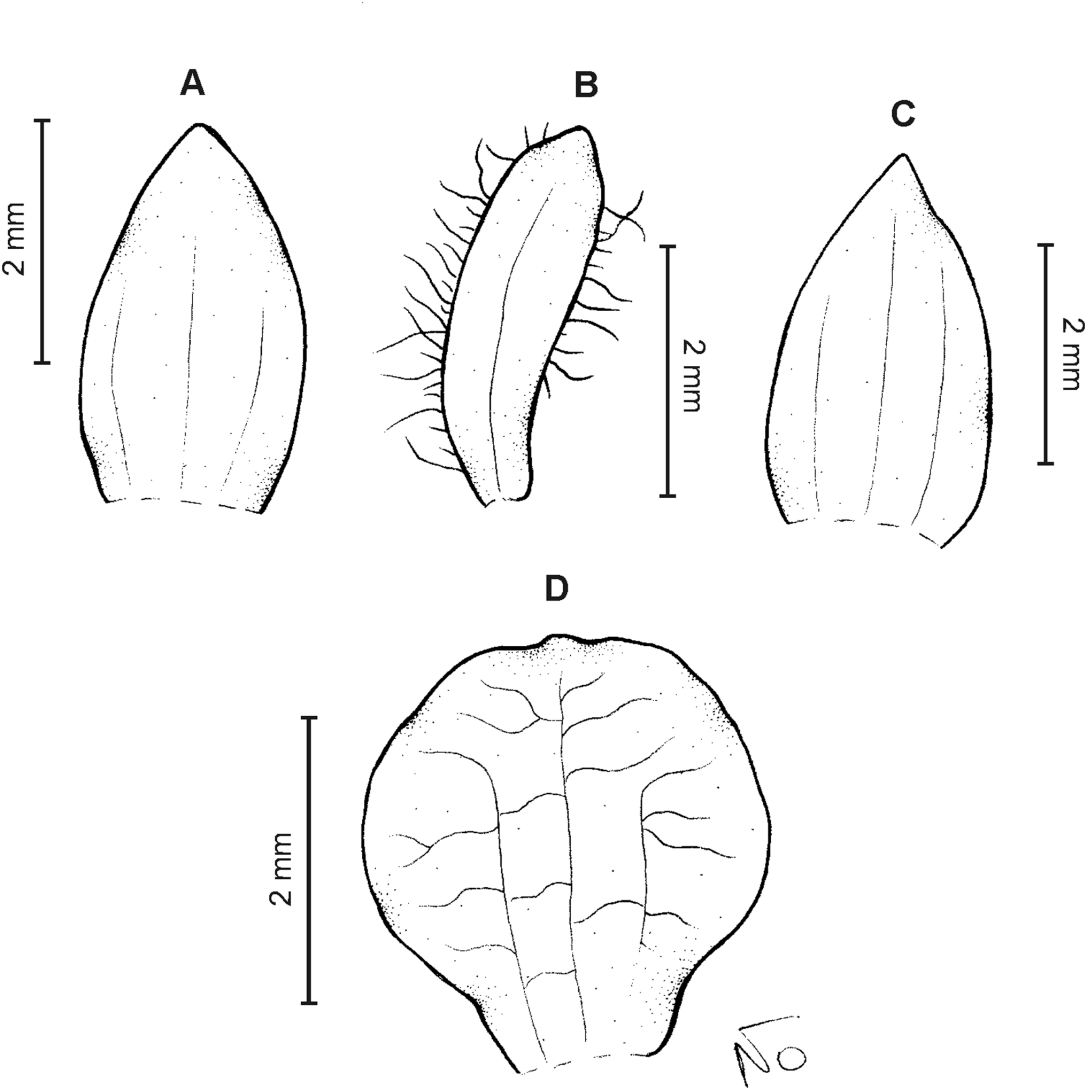
*Cranichis antioquiensis* Schltr. (A) Dorsal sepal; (B) petal; (C) lateral sepal; (D) lip (front view). Drawn by N. Olędrzyńska from *Ospina H. 1577* (COL).

Plants up to 65 cm tall. Leaves 1–2, basal, petiolate; petiole 10–17 cm long, narrow, canaliculate; blade 6–13 cm long, 3–6.5 cm wide, elliptic-ovate, somewhat oblique, acute, base cordate. Scape erect, delicate, glabrous in the lower half, densely glandular below and within inflorescence, enclosed distantly in three to seven sheaths. Inflorescence up to 14 cm long, cylindrical, laxly many-flowered. Flowers small, inconspicuous. Floral bracts six to eight mm long, lanceolate, acuminate, glabrous. Subsessile ovary 7–10 mm long, glabrous. Sepals glabrous. Dorsal sepal three to five mm long, 1.2–2.1 mm wide, oblong-ovate to ovate-lanceolate, subacute, somewhat cochleate in the center, 3-veined. Petals 2.2–4.8 mm long, 0.7–1 mm wide, oblong- or linear-lanceolate, subacute to subobtuse, falcate, densely and softly pubescent-papillate along both margins except base and apex, 1-veined. Lateral sepals 3–5.7 mm long, 1.3–2.2 mm wide, obliquely ovate to elliptic-ovate, acute to subacute, slightly concave, 3(1)-veined. Lip 2–3.3 mm long, 2–3.3 mm wide, shallowly cochleate below the center, sessile, elliptic-obovate in outline, widest near the middle, apex truncate to rounded; disc 3-veined, veins protruding, branching and anastomosing, sometimes secondary branches can be observed, without any nodules. Gynostemium 1.2–2.5 mm long (Figs. 14–16).

*Ecology*: Terrestrial plant growing on embankments, in well-drained soil, in moist soil on wooded slopes, and in shade of very humid forest at an altitude of 1,500–3,300 m. Flowering throughout the year.

*Distribution*: Colombia, Ecuador.

**Figure 17:**
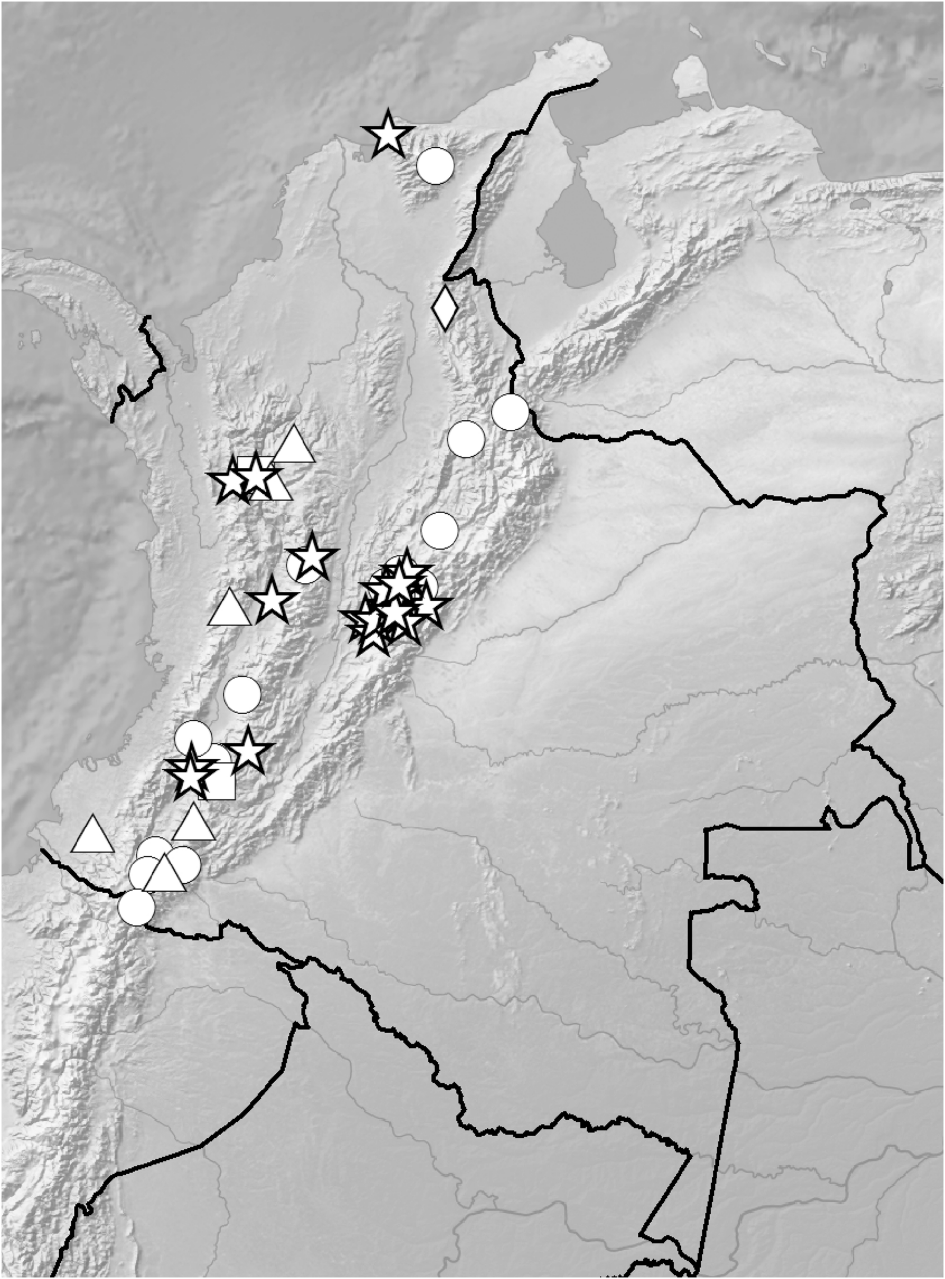
Distribution of members of the *Cranichis Ciliata* group. *C. antioquiensis* (circle), *C. barkleyi* (square), *C. ciliata* (triangle), *C. killipii* (diamond), *C. zarucchii* (star).

*Representative specimens*: COLOMBIA. **Antioquia**: *Sine loc.*, 2,000 m, *M. Madero s.n*. (B†); *Sine loc., M. Madero 168* (B†; AMES—drawing). **Boyacá**: Mpio. de Arcabuco. En los alrededores de la poblacion Arcabuco, 2,739–2,850 m, October 20, 1965, *G. Huertas & L. Camargo 6266 & 6283* (COL!). **Caldas**: Salento to Laguneta, old Quindío trail. Cordillera Central, 2,500–2,800 m, August 1, 1922, *E. Killip & T. Hazen 11892* (AMES!). **Cauca**: *F.C. Lehmann 6327* (NY!, UGDA-DLSz!—drawing); About 15 km from Popayán, 2,200 m, April 10, 1958, *D. Correll Co512* (AMES!, UGDA-DLSz!—drawing); Cannan, Mt. Puracé. Cordillera Central, 3,100–3,300 m, June 11–13, 1922, *F. Pennell & E. Killip 6644* (AMES!); Wet glan in forest, San José. San Antonio. Cordillera Occidental, 2,400–2,700 m, June 28, 1922, *F. Pennell & E. Killip 7329* (AMES!); El Tambo. Parque Nacional Natural Munchique. Km 45–47 vía La Romelia a La Gallera. Talud a orilla de la carretera, 2,440 m, April 11, 1994, *J. lvarez et al. 37* (COL!). **Cundinamarca**: Mpio. de Sesquilé. Sendero inferior de La Laguna (Antigua) de Guatavita, 3,000 m, October 3, 1995, *J.L. Fernandez Alonso et al. 12698 & 12700* (COL!); Mpio. de Sesquilé. Vereda San José, quebrada Granadillo, 2,560 m, November 14, 1998, *M. Acosta et al. 140* (COL!); Mpio. de Subachoque. Vereda Tobal, finca El Cerro, 2,950 m, September 7, 2002, *M. Hernandez Schmidt 880* (COL!); Sopó, 2,600–2,700 m, September 20, 1953, *M. Schneider 172/2* (COL!); Entre Zipaquira y Cogua, La Juratena, 2,670 m, October 8, 1942, *L. Camargo & G. Huertes 1120* (COL!); Represa del Sisga, November 8, 1953, *A. Richter s.n*. (COL!); Sisga, 2,850 m, October 3, 2003, *M. Ospina H. 1577* (COL!); Quebrada del Chicó, 2,800 m, October 24, 1944, *M. Schneider 172/1* (COL!). **Magdalena**: Sierra Nevada de Santa Marta. Valle del Río Donachuy, camino Sacaracungue-Canguruaca, 1,975 m, October 14, 1958, *T. van der Hammen 1099* (COL!). **Nariño**: Mpio. de Pasto. Bosque de Daza, km 8 de la carretera al Aeropuerto de Cano, 2,790 m, May 22, 1967, *G. Lopez C. 96* (COL!); Tangua. Vereda de Cubijan. Cerca a Pasto. Terricola, 3,100 m, July 28, 1965, *L. Uribe U. 5331* (COL!); Cordillera Oriental. Camino between La Victoria and Pun (Prov. Carchi, Ecuador) in Río Chingual drainage, 2,740 m, September 24, 1944, *J.A. Ewan 16196* (AMES!, US!, UGDA-DLSz!—drawing); Mpio. Túquerres. En el camino de Túquerres al Volcán Azufral, July 1963, *L. Mora 2733* (AMES!, UGDA-DLSz!—drawing); **Norte de Santander**: Culaga Valley, near Tapata (N of Toledo), 1,500–2,100 m, March 3–8, 1927, *E. Killip & A. Smith 20172* (AMES!). **Putumayo**: Valle de Sibundoy. Two kilometer NE Sibundoy, 2,150 m, November 14, 1962, *M. Bristol 357* (AMES!, UGDA-DLSz!—drawing). **Santander**: Between Piedecuesta and Las Vegas, 2,000–2,500 m, December 19–21, 1926, *E. Killip & A. Smith 15571* (AMES!). **Valle del Cauca**: La Nevera, January 26, 1980, *I. Guarin O. 114* (COL!) (Fig. 17).

*Other materials examined:* ECUADOR. **Azuay**: Quebradas leading into the Río Collay, three to eight km N of Sevilla de Oro, 2,300–2,800 m, August 27, 1945, *W. Camp E-4999* (AMES!, UGDA-DLSz!—drawing). **Bolivar**: Guaranda-Caluma-Catarama, km 9.4, 2,875 m, July 5, 1991, *C. Dodson et al. 18774* (RPSC!, UGDA-DLSz!—drawing); Cañar. Under thorn scrub at Topicocho on RR from Sibambi to Azogues, 3,000 m, May 8, 1958, *C. Dodson 353* (RPSC!, UGDA-DLSz!—drawing). **Tungurahua**: Road Patate to Leito to Llanganates Range, 3,000 m, April 12, 1985, *A. Hirtz et al. 2495* (RPSC!).

*Notes*: This species appears to be quite common in Ecuador and Colombia and it is characterized by having 3-veined dorsal and lateral sepals. The petals of this species are densely and softly pubescent-papillate along both margins except base and apex. This character is shared with *C. zarucchii*, but unlike in the latter species, petals of *C. antioquiensis* are densely and softly pubescent-papillate, its lip is elliptic-obovate in outline, widest near the middle, with truncate to rounded apex.

This species appears to be related to Ecuadorian *C. sororia* Schltr., but is stronger and fleshier in all parts, with smaller flowers and different petals, lip and thicker gynostemium (Schlechter, 1920).

Schlechter in the protologue of this species cited as the type *Madero* collection with no number.

***Cranichis barkleyi*** Szlach. & Kolan., ***sp. nov***. TYPE: Colombia. *Valderrama & Barkley 18A215* (holotype, AMES!; isotype, US, UGDA-DLSz!—drawing).
*Species distinguished from C. zarucchii by having 1-veined dorsal sepal, sparsely pilose petal margins and shortly unguiculate, elliptic-ovate lip*.

**Figure 18:**
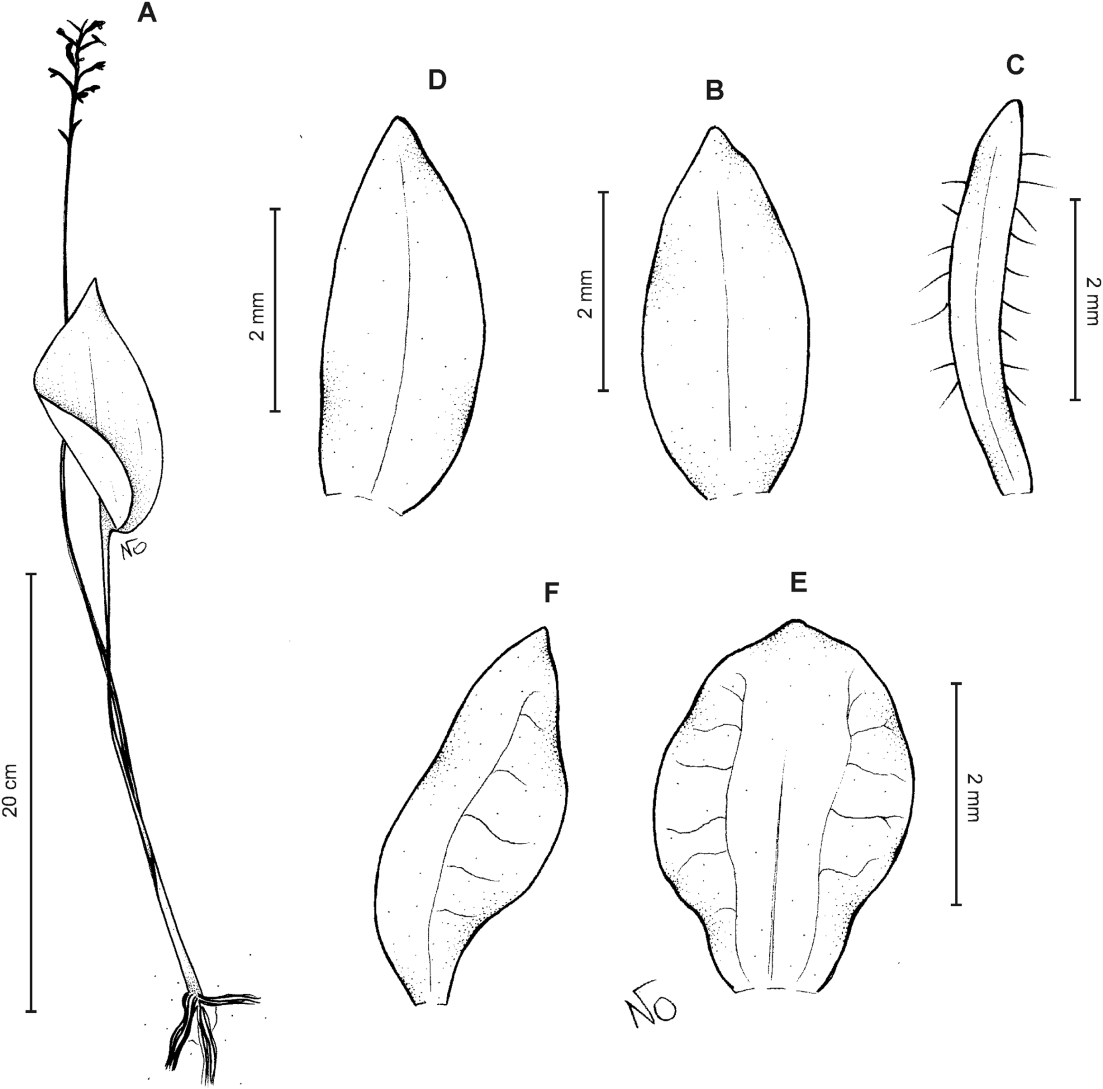
*Cranichis barkleyi* Szlach. & Kolan. (A) Habit; (B) dorsal sepal; (C) petal; (D) lateral sepal; (E) lip (front view); (F) lip (side view). Drawn by N. Olędrzyńska from *Valderrama & Barkley 18A215* (AMES).

**Figure 19:**
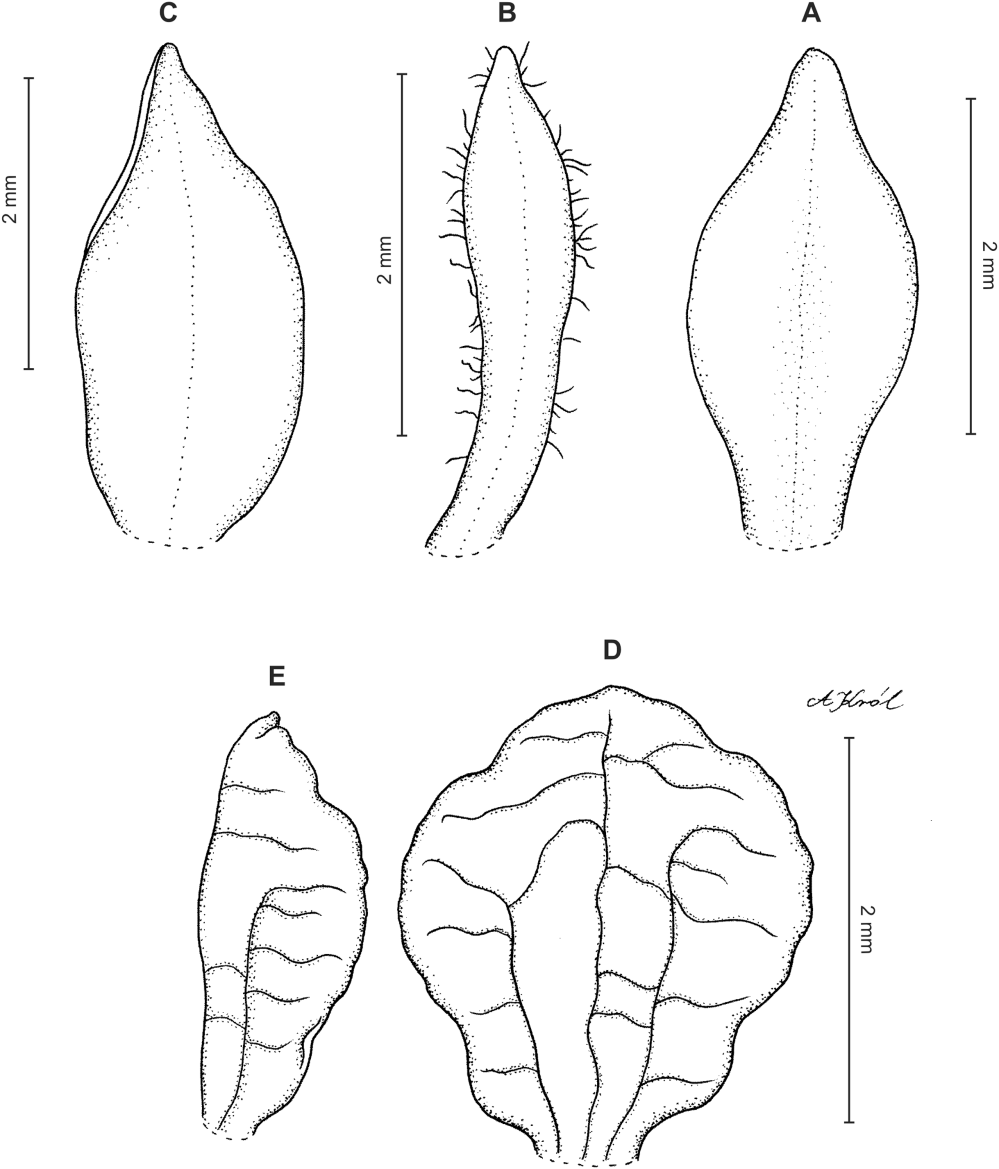
*Cranichis barkleyi* Szlach. & Kolan. (A) Dorsal sepal; (B) petal; (C) lateral sepal; (D) lip (front view); (E) lip (side view). Drawn by A. Król from *Andre 4508* (K).

Plants up to 45 cm tall. Leaf l, basal, petiolate; petiole 9–17 cm long, narrow, canaliculate; blade 9–10 cm long, four to seven cm wide, ovate, ovate-elliptic, acute or acuminate, cordate at the base, oblique. Scape slender, glabrous in lower part, glandular above, enclosed in four sheaths. Inflorescence three to four cm long, cylindrical, rather loosely many-flowered. Flowers white. Floral bracts five to eight mm long, lanceolate, acuminate, glabrous. Pedicellate ovary eight to nine mm long, glabrous. Sepals glabrous. Dorsal sepal 3–4.5 mm long, 1.1–2 mm wide, elliptic-ovate to oblong ovate, subacute, 1-veined. Petals 2.8–4 mm long, 0.4–0.8 mm wide, narrowly linear to narrowly ligulate, somewhat falcate, subobtuse, margins sparsely pilose, 1-veined. Lateral sepals 3.5–4 mm long, 1.6–2 mm wide, oblong ovate, oblique at base, subacute, 1-veined. Lip 2.5–3.5 mm long, 2–2.8 mm wide, gibbose at the base, cochleate above, shortly unguiculate, obovate in outline, rounded at apex; disc 3-veined, veins thickened, laterals with dendritic branching, without any nodules. Gynostemium two mm long (Figs. 18 and 19).

*Etymology*: Named in honor of the co-collector of the type specimen.

*Ecology*: Terrestrial plant growing in subparamo and montane forest at an altitude of 2,600–3,100 m. Flowering occurs in June and September.

*Distribution*: Colombia, Ecuador.

*Representative specimens*: COLOMBIA. **Antioquia**: One km al S de Hoya Rico, 2,600 m, September 26, 1948, *L. Valderrama & F. Barkley 18A215* (AMES!, US, UGDA-DLSz!—drawing). **Cauca**: Cannan. Mt Puracé. Cordillera Central, 2,900–3,100 m, June 11–16, 1922, *E.P. Killip 6791* (AMES!, NY!, US!, UGDA-DLSz!—drawing). **Cundinamarca**: Bogotá. Localidad de Usaquén, Torca, Finca Tibabitá. 04°46′45.7″N 74°01′13.9″W, December 18, 2012, *J. Valencia et al. 1619* (COL!, UGDA-DLSz!—drawing) (Fig. 17).

*Other material examined*: ECUADOR. *Andre 4508* (K!, UGDA-DLSz!—drawing).

*Notes*: This species, known from Colombia and Ecuador, is distinguished by having 1-veined sepals, sparsely pilose petal margins, not found in any other species of this group. From similar *C. zarucchii* the new species differs in venation of dorsal sepal (1- vs. 3-veined). Moreover, petals of *C. zarucchii* are densely and softly pubescent along both margins except base and apex and the lip is sessile (vs. shortly unguiculate), elliptic-ovate in outline (vs. obovate).

***Cranichis ciliata*** (Kunth) Kunth, Syn. Pl. Aeq. 1: 324. 1822.
*Ophrys ciliata* Kunth, Nov. Gen. Sp. (quarto ed.) 1: 334, t. 74. 1816. TYPE (here designated): Venezuela. *Humboldt s.n*. (lectotype, W!).

**Figure 20:**
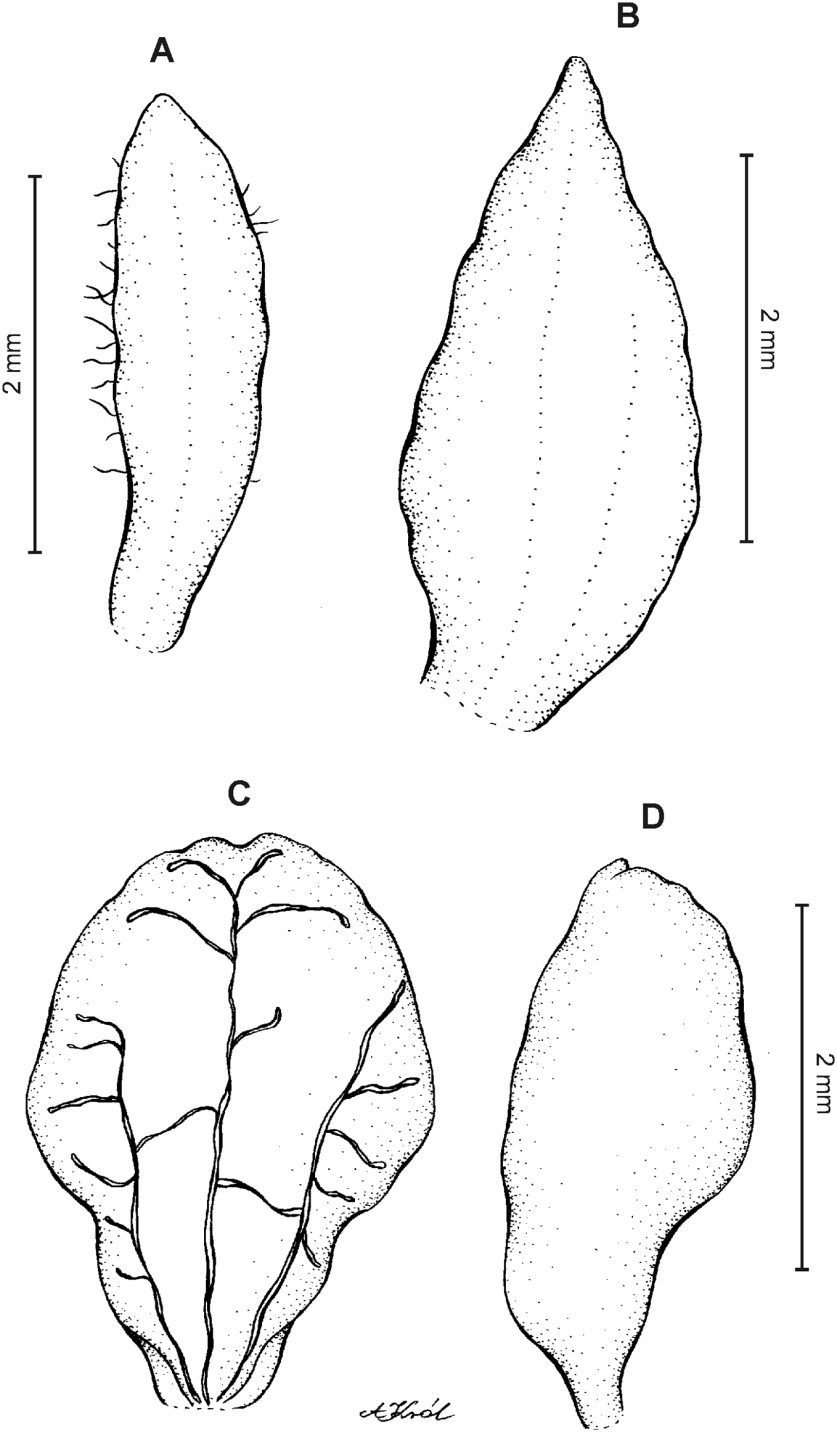
*Cranichis ciliata* (Kunth) Kunth. (A) Petal; (B) lateral sepal; (C) lip (front view); (D) lip (side view). Drawn by A. Król from *Humboldt s.n*. (W-R).

**Figure 21:**
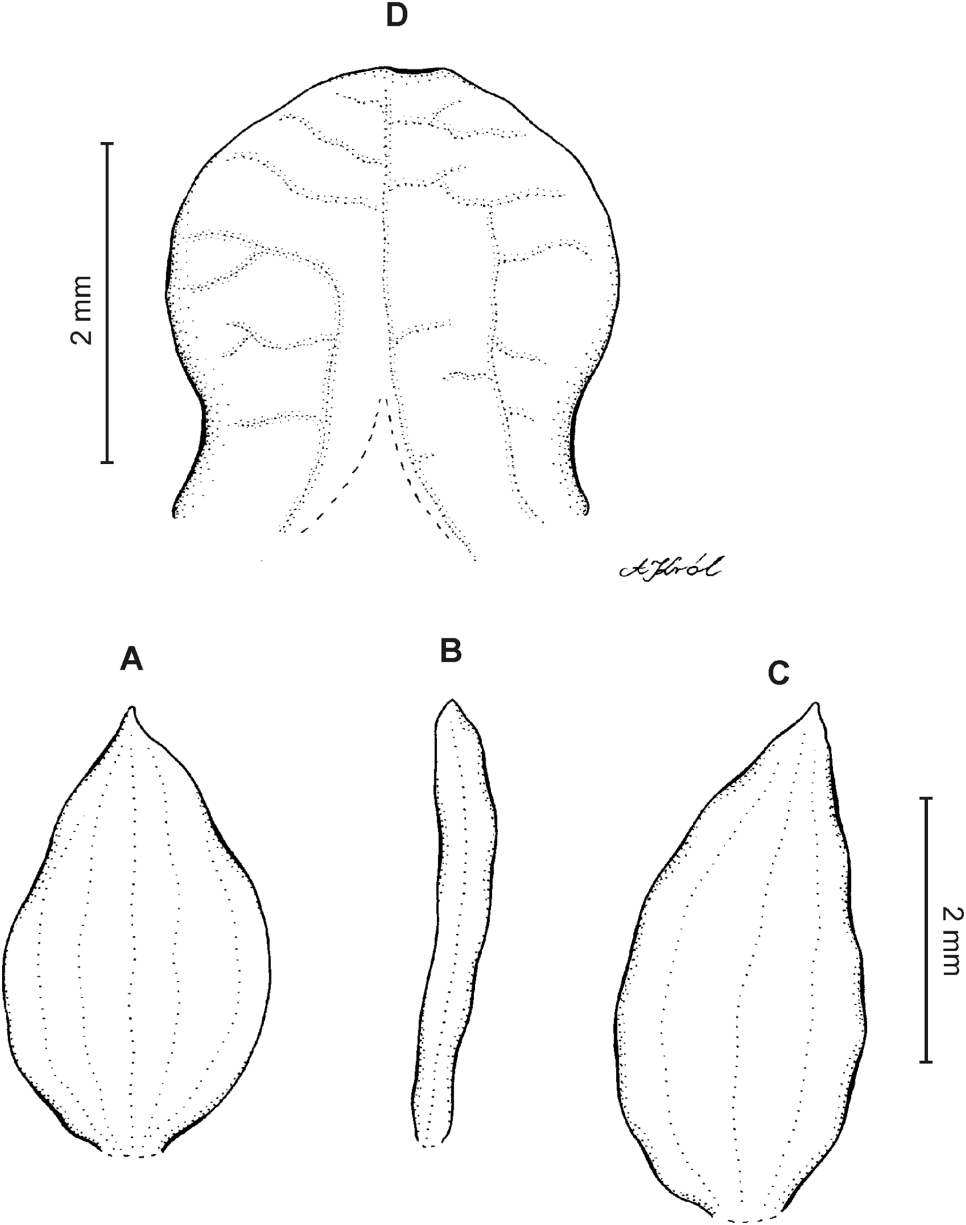
*Cranichis ciliata* (Kunth) Kunth. (A) Dorsal sepal; (B) petal; (C) lateral sepal; (D) lip (front view). Scale bar for (A–C) = 2 mm. Drawn by A. Król from *Lehmann 1* (W-R).

Plants up to 60 cm tall. Leaves l, rarely 2, basal, petiolate; petiole 7.5–15 cm long, narrowly winged; blade 5–12 cm long, 2.3–7 cm wide, ovate, ovate-lanceolate or elliptic, abruptly acute or acuminate, broadly rounded to subcordate at the base, oblique, somewhat variegated. Scape slender, glabrous in lower part, glandular-pubescent above, enclosed in four to six sheaths. Inflorescence 4–15 cm long, cylindrical, rather densely few- to many-flowered. Flowers white, marked with green or purple-brown. Floral bracts six to eight mm long, lanceolate, acuminate, glabrous. Pedicellate ovary 6–11 mm long, glabrous. Dorsal sepal 3.3–4.2 mm long, 1.5–2 mm wide, elliptic to elliptic-oblong, obtuse, the margins involute, 1-, 3-, or occasionally 5-veined. Petals three to four mm long, 0.7–1 mm wide, oblong-lanceolate to narrowly-ligulate, somewhat falcate, subobtuse, margins unevenly pilose, 1-veined. Lateral sepals 3–4.2 mm long, 1.6–2.2 mm wide, lanceolate-ovate, oblique at base, subacute, 2- or 3-veined. Lip 2.5–4 mm long, 2–3.5 mm wide, gibbose at the base, cochleate above, shortly unguiculate, obovate in outline, rounded and slightly notched at apex; disc 3-veined, veins thickened, dendritic branching, without any nodules. Gynostemium 1.3–2.5 mm long (Figs. 20 and 21).

*Ecology*: Terrestrial plant growing in shade of cloud forest, along roadsides at an altitude of 750–3,000 m. Flowering occurs throughout the year.

*Distribution*: Mexico (Breedlove, 1986), Guatemala (Ames & Correll, 1985), El Salvador (Hamer, 1974), Honduras (Hamer, 1988), Nicaragua (Hamer, 1985), Costa Rica (Dressler, 2003), Panama (Christenson, 1991), Argentina (Correa, 1972), Bolivia, Peru, Ecuador, Colombia, Venezuela.

*Representative specimens*: COLOMBIA. **Antioquia**: *Sine loc*. (Schlechter, 1920); El Yarumal. Highlands of Santa Rosa, 2,000–2,400 m, November 19, 1891, *F.C. Lehmann 7258* (AMES!, NY!, UGDA-DLSz!—drawing); San Heronimo, 1,600–1,800 m, October 10, 1884, *F.C. Lehmann 8155* (AMES!, NY!). **Cauca**: Bei Casabamba an der Cocha bei Pasto, 3,000 m, October 25, 1878, *F.C. Lehmann 1* (W!); Along camino between Valencia and San Sebastián, E slope of mountain, 2,700–3,000 m, July 21, 1944, *E.L. Core 1020* (US!, UGDA-DLSz!—drawing). **Cundinamarca**: *Sine loc*. (Schlechter, 1920). [**Nariño**?]. Barbacoas, 750 m, 1851–1857*, J.J. Triana 621* (P!, US!). **Valle del Cauca**: Mpio. El Cairo. Las Amarillas, frontera Valle-Choco, Cordillera Occidental, Serrania de los Paraguas, carratera destapada El Cairo-Rio Blanco a 1 hora en jeep de El Cairo. Zona abierta al lado de carratera, 2,100 m, March 30, 1988, *F. Silverstone-Sopkin et al. 3817* (CUVC!) (Fig. 17).

*Other materials examined*: BOLIVIA. **Larecaja**: Sorata, 2,650–3,100 m, Apr–May 1860, *G. Mandon 1163* (W!). PERU. Muña, 2,400 m, May–Jun 1923, *J. Macbride 4045* (W!). ECUADOR. *Sine loc*. April 1986, *A. Hirtz s.n*. (RPSC!). **Napo**: Acerca de Papallacta, Quito-Baeza, 3,000 m, October 1983, *A. Hirtz 1335* (RPSC!). **Pichincha**: La Iberia, W del Volcán Pululagua, 2,600 m, March 1984, *A. Hirtz 1593* (RPSC!, UGDA-DLSz!—drawing); Volcán Pululagua, 35 km NW of Quito, beyond the Monument to the Center of the Earth, 2,300 m, May 1985, *A. Hirtz 2583* (RPSC!, UGDA-DLSz!—drawing); *In Andibus*. 1857–1859, *R. Spruce 5216* (W!); *W. Jameson s.n*. (W!); Bei Baeza am Wege von Quito nach Archidona, January 16, 1880, *F.C. Lehmann 470* (W!). VENEZUELA. Colonia Tovar bei Caracas, *H. Karsten s.n*. (W!); *Sine loc., A. Humboldt s.n*. (W!).

*Notes: C. ciliata* is one of the most widespread species in the genus known from Florida to Argentina and Bolivia. It is not unusual for a plant with such a large geographical range to be quite variable. It is distinguishable by its nonfoliaceous scape, green leaves, petals unevenly pilose along both margins, and glabrous ovary. The dorsal sepal is usually 1-veined, but we found specimens with either three or five veins, and the lateral sepals can be 2- or 3-veined.

According to Ames & Correll (1952) *C. ciliata* is distinguishable from closely allied species, particularly *C. silvatica*, by its orbicular lip and the mid-stripe of the disc, which extends beyond the lateral stripes and branches near the apex.

Garay (1978) synonymized under this name *C. atrata* and *C. sororia. C. ciliata* differs from the latter species in having pilose petal margins (vs. long-ciliate) and shortly unguiculate (vs. subsessile), obovate (vs. suborbicular) lip.

***Cranichis killipii*** Szlach. & Kolan., ***sp. nov***. TYPE: Colombia. *Killip & Smith 16120* (holotype, AMES!, UGDA-DLSz!—drawing).
*Species distinguished from C. ciliata by having white flowers, petals being distinctly narrowed in the basal fourth and lip apiculate, rounded to obtuse at the apex*.

**Figure 22:**
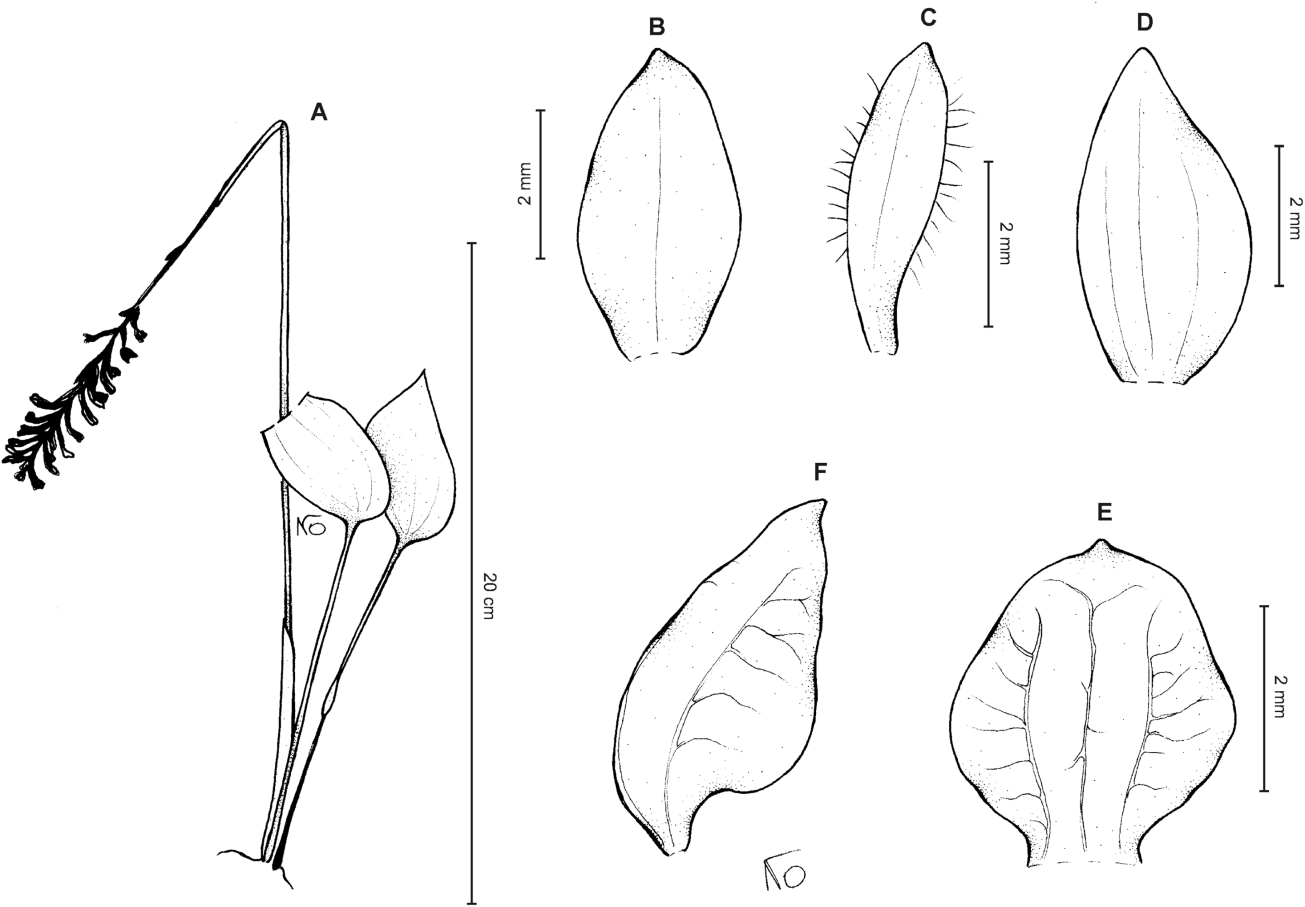
*Cranichis killipii* Szlach. & Kolan. (A) Habit; (B) dorsal sepal; (C) petal; (D) lateral sepal; (E) lip (front view); (F) lip (side view). Drawn by N. Olędrzyńska from *Killip & Smith 16120* (AMES). Scale bar (E–F) = 2 mm.

**Figure 23:**
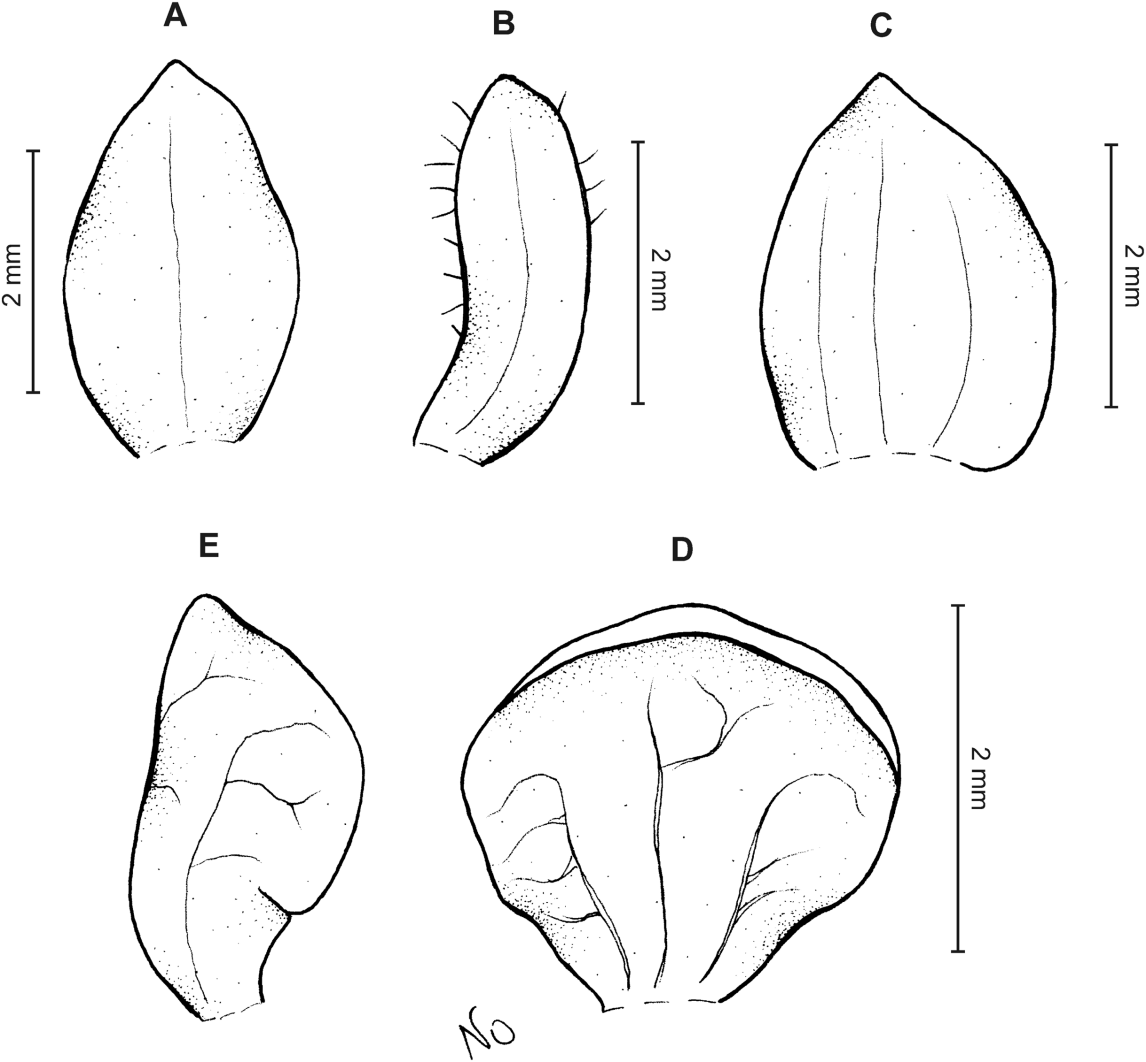
*Cranichis killipii* Szlach. & Kolan. (A) Dorsal sepal; (B) petal; (C) lateral sepal; (D) lip (front view); (E) lip (side view). Drawn by N. Olędrzyńska from *Dalessandro 620* (RPSC).

Plants 27–40 cm tall. Leaves 1 or 2, basal, petiolate; petiole 8–16 cm long, narrowly winged; blade six to eight cm long, three to four cm wide, oblong-elliptic to elliptic-ovate, acute, cuneate to subcordate at base, oblique. Scape slender, glabrous in lower part, glandular-pubescent above, enclosed in four to seven sheaths. Inflorescence 4–7.5 cm long, cylindrical, subdensely many-flowered. Flowers white. Floral bracts four to six mm long, lanceolate, acuminate, glabrous. Pedicellate ovary 6–10 mm long, glabrous. Dorsal sepal 3.2–4.2 mm long, 1.3–1.6 mm wide, elliptic, obtuse to subacute, 1-veined. Petals 3–3.8 mm long, 0.6–1 mm wide, oblong-oblanceolate to narrowly ligulate, distinctly narrower in the basal fourth, falcate, subobtuse, margins sparsely and unevenly pilose, 1-veined. Lateral sepals 3–4.8 mm long, 1.9–2.2 mm wide, lanceolate-ovate to broadly ovate, oblique at base, subacute, glabrous, 3-veined. Lip 2.3–3.5 mm long, 2.5–3.3 mm wide, gibbose at the base, cochleate above, shortly unguiculate, obovate to broadly obovate in outline, rounded to obtuse at apiculate apex; disc 3-veined, veins thickened, dendritic branching, without any nodules. Gynostemium 1.2–2.2 mm long (Figs. 22 and 23).

*Etymology*: Named in honor of the senior collector of the type specimen.

*Ecology*: Terrestrial plant growing on roadsides at an altitude of 2,600–3,000 m. Flowering occurs in April and December.

*Distribution*: Ecuador, Colombia.

*Representative specimens*: COLOMBIA. **Cundinamarca**: Macizo Bogotá. El Chicó, December 27, 1943, *M. Schneider 172* (COL!). **Santander**: Vicinity of Las Vegas, 2,600–3,000 m, December 21–23, 1926, *E. Killip & A. Smith 16120* (AMES!, UGDA-DLSz!—drawing) (Fig. 17).

*Other material examined*: ECUADOR. **Loja**: San Pedro de Vilcabamba entering from along the road to Loja and walking two km up the path, 2,400 m, April 23, 1986, *D. Dalessandro 620* (RPSC!, UGDA-DLSz!—drawing).

*Notes*: The species is similar to *C. ciliata* by having 2- or 3-veined lateral sepals. It can be distinguished from the latter based on white flowers (vs. flowers white marked with green or purple-brown), petals being distinctly narrower in the basal fourth (vs. petals ligulate), and lip rounded to obtuse at the apiculate apex (vs. lip rounded and slightly notched at apex).

***Cranichis zarucchii*** Szlach. & Kolan., Pl. Syst. Evol. 299(5): 981. 2013. TYPE: Colombia. *Zarucchi et al. 5694* (holotype, COL!; isotypes, AMES!, K!, MO!, NY!).

**Figure 24:**
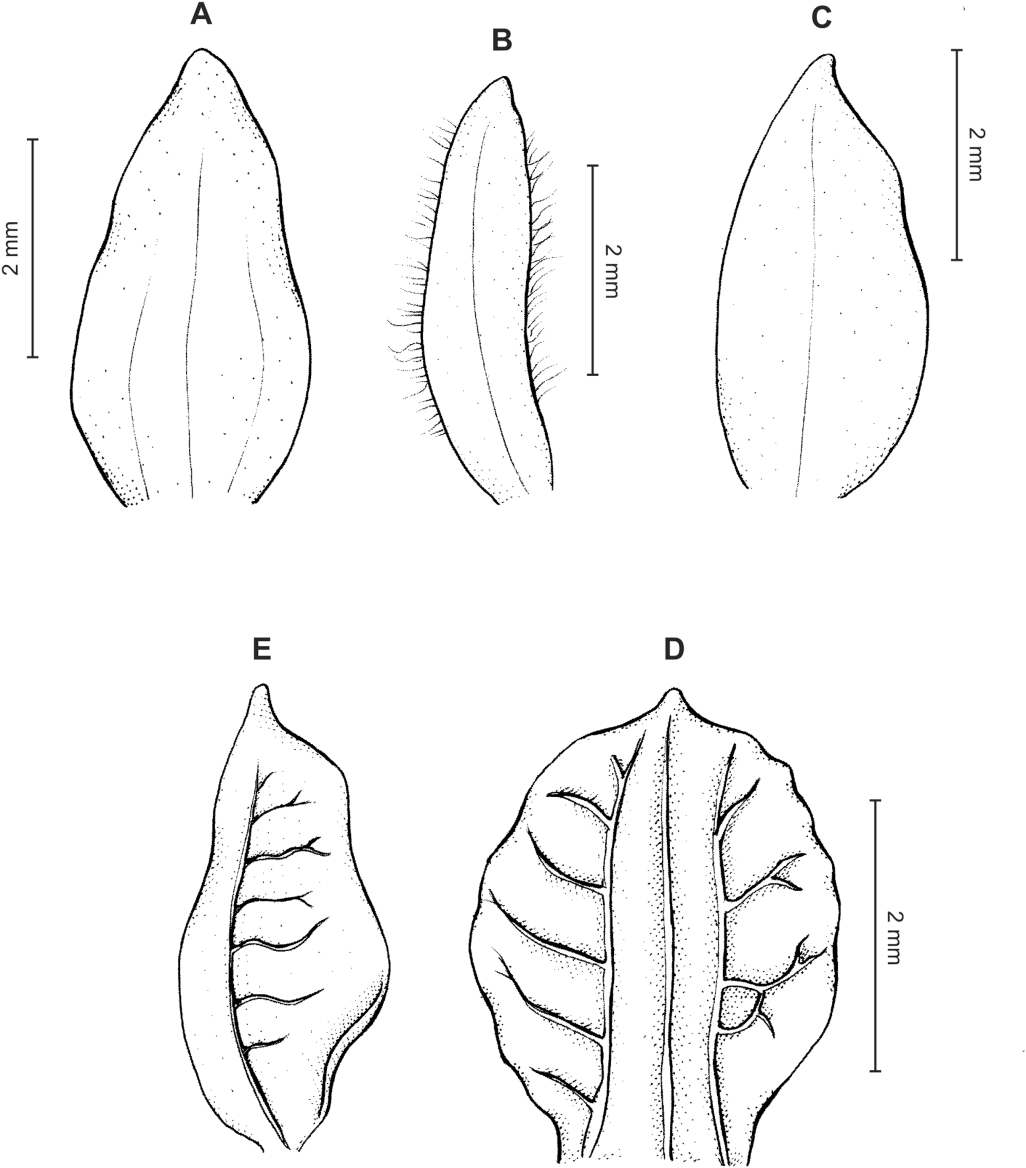
*Cranichis zarucchii* Szlach. & Kolan. (A) Dorsal sepal; (B) petal; (C) lateral sepal; (D) lip (front view); (E) lip (side view). Drawn by P. Baranow from *Zarucchi et al. 5694* (COL).

**Figure 25:**
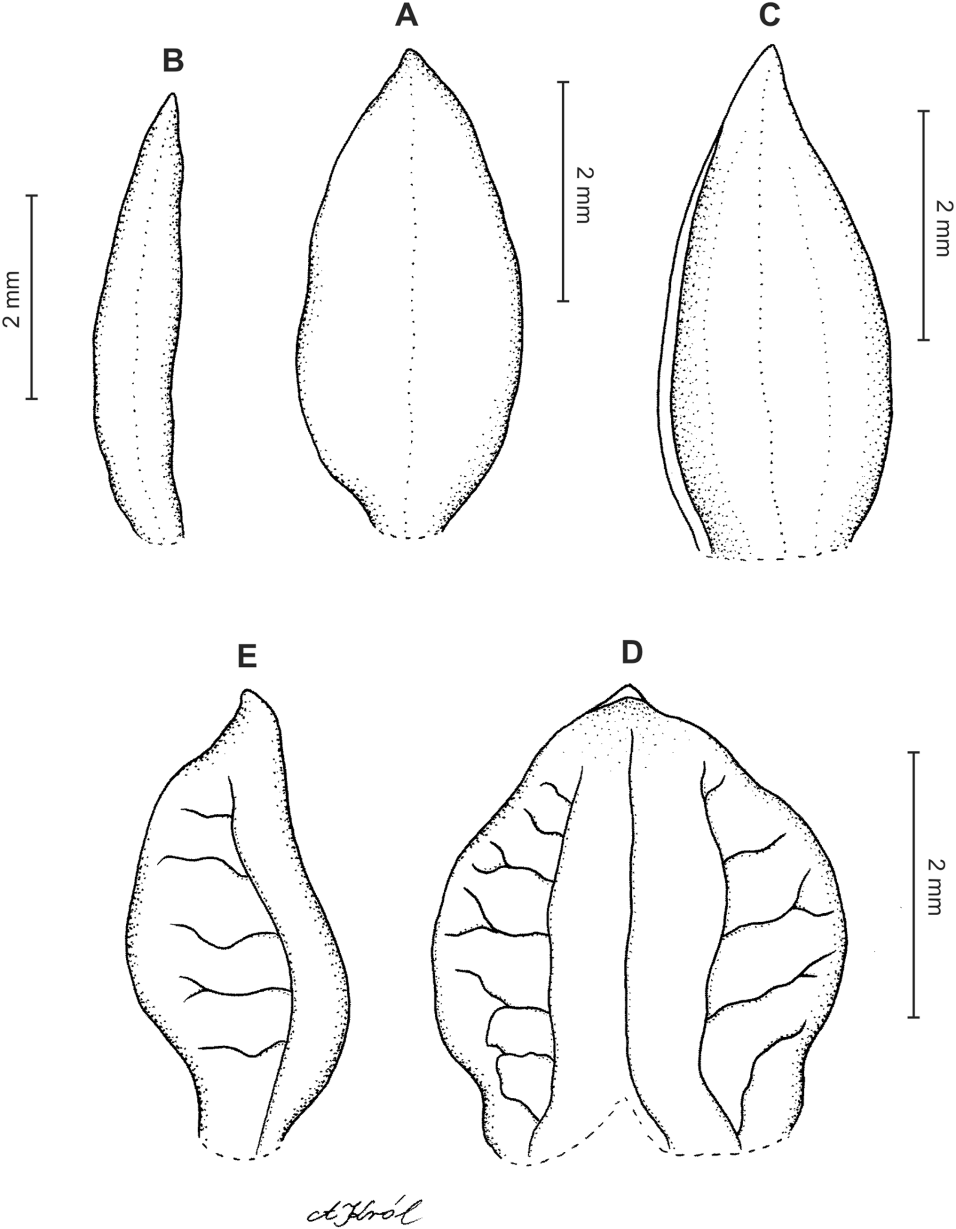
*Cranichis zarucchii* Szlach. & Kolan. (A) Dorsal sepal; (B) petal; (C) lateral sepal; (D) lip (front view); (E) lip (side view). Drawn by A. Król from *Zarucchi et al. 5694* (K).

Plants up to 55 cm tall. Leaves 1–2, basal, petiolate; petiole 10–18 cm long, narrow, canaliculate; blade 6.5–12.5 cm long, 2.5–7.5 cm wide, triangular-ovate to oblong ovate, somewhat oblique, acute, base cordate. Scape erect, delicate, glabrous in the lower half, densely glandular below and within inflorescence, enclosed distantly in three to seven sheaths. Inflorescence up to 12 cm long, cylindrical, laxly to subdensely many-flowered. Flowers small, inconspicuous. Floral bracts five to six mm long, lanceolate, acuminate, glabrous. Subsessile ovary up to nine mm long, densely and very minutely papillate or glabrous. Sepals glabrous. Dorsal sepal 3–4.2 mm long, 1.6–2.1 mm wide, oblong-ovate, subobtuse, somewhat cochleate in the center, 1- or obscurely 3-veined. Petals 2.8–4 mm long, 0.8–0.9 mm wide, oblong- or linear-lanceolate, subacute to subobtuse, falcate, densely and softly pubescent along both margins except base and apex, 1-veined. Lateral sepals 3–4.2 mm long, 1.6–2.1 mm wide, obliquely ovate to elliptic-ovate, somewhat acuminate, acute to subacute, slightly concave, 1- or 3-veined. Lip 2.6–4 mm long, 2.7–3.7 mm wide, shallowly cochleate below the center, sessile, elliptic-ovate in outline, widest just below the middle, apex truncate with short, triangular, acute apiculus, margins somewhat wavy at base; disc 3-veined, veins protruding, profusely branching, sometimes secondary branches can be observed, without any nodules. Gynostemium 1.6–2.5 mm long (Figs. 24 and 25).

*Ecology*: Terrestrial plants growing in pluvial subparamo as well as in the wet and very wet montane areas at altitudes of 1,750–3,200 m. Flowering occurs throughout the year.

*Distribution*: Colombia.

*Representative specimens*: COLOMBIA. **Antioquia**: Mpio. Sonsón. Km 11 of road Sonsón-Nariño 25 km from Narino, near km post 151 marking distance from Bogotá. Pluvial subparamo vegetation, 5.42′N, 75.15′W, 2,780 m, April 1, 1987, *J.L. Zarucchi et al. 5177* (COL!, MO!, NY!); Sobre la carretera hacia Nariño, 2,740 m, May 5, 1972, *G. Lozano C. & J. Rivera 2211* (COL!); Mpio. Frontino. Km 12 of road Nutibara-Murri. Disturbed wet/very wet montane vegetation, roadside, 6.45′N, 76.22′W, 2,010 m, September 23, 1987, *J.L. Zarucchi et al. 5694* (COL!, K!, MO!); one km al S de Hoya Rico, 2,600 m, September 26, 1948, *J. Correa & F. Barkley 18A198* (AMES!, US, UGDA-DLSz!—drawing). **Cauca**: Mpio. de Inza. Vereda Río Sucio. Jardín Botánico las Delicias, km 62, 2,700–2,800 m, September 2, 2003, *E.L. Munoz E. et al. 1602* (COL!, UGDA-DLSz!—drawing); Mpio. de Almaguer. La Cuchilla, 3,100 m, April 15, 1999, *R. Javier 13* (COL!). **Cundinamarca**: Mpio. de Alban. Granjas del Padre Luna. Carretera Alban-Sasaima, bosque secundario, 4°53′N, 74°2′W, 2,200 m, May 15, 2001, *J. Vera et al. 40* (COL!); Mpio. de Fusagasuga. La Aguadita, October 29, 1988, *F. Sarmiento 2152* (COL!); Mpio. de Gama. Vía Gacheta. Vda. Pauto, 1,750 m, October 9, 1992, *A. Chaparro de Barrera & E. Barrera Torres 224* & *317* (COL!); Mpio. de Mesitas. Abajo del salto de Taquendama. Montes arriba de Santibar, 2,100 m, October 31, 1965, *L. Uribe U. 5451* (COL!); Mpio. de Nemocon. Quebrada Checua, 2,600 m, 1996, *T. van der Hammen 7153* (COL!); Mpio. de Suba. Hacienda Las Mercedes, October 5, 1964, *E. Forero & R. Jaramillo 49* (COL!); Mpio. de Suesca. Vereda de Hato Grande. Hacia el ferrocarril, parte NW del caserio, December 19, 1963, *C. Saravia T. & H. Torres R. 3188* (COL!); Mpio. de Suesca. Hacia el caserio de Hato Grande. El Crucero, 3,100–3,200 m, December 18, 1963, *C. Saravia T. & R. Restrepo M. 3144* (COL!); Mpio. de Sesquilé. Vereda San José. Quebrada Granadillo, 2,560 m, November 14, 1998, *M. Acosta et al. 160* (COL!); Carretera entre Subachoque y La Pradera. Vertiente oriental, 3,000 m, November 24, 1956, *M. Ospina H. & J.M. Idrobo 44* (AMES!, COL!); Alrededores de Bogotá. Quebrada El Chicó, 2,700 m, June 15, 1944, *M. Schneider 11/2* (COL!); Alrededores de Bogotá. Quebrada de El Chicó, 2,700 m, October 28, 1950, *M. Schneider 11/3* (COL!); Alrededores de Bogotá. Quebrada de El Chicó, 2,700–2,800 m, May 18, 1944, *M. Schneider 11/1 p.p*. (COL!); Maciza de Bogotá. Quebrada Chicó, 2,750–2,890 m, June 8, 1939, *J. Cuatrecasas 5425* (COL!); Paramo de Guasca, July 1919, *Bro Ariste-Joseph A404* (US!, UGDA-DLSz!—drawing). **Huila**: Mpio. de Toez. Vía al nevado del Huila, 2°47′0″N, 76°01′26″, December 9, 1993, *B. Cesar 9652* (COL!). **Magdalena**: Mpio. de Santa Marta. Corregimiento de Minca. Sierra Nevada de Santa Marta. Cerro San Lorenzo, Cabana Inderena, 2,100–2,500 m, November 23, 1985, *J.H. Torres & P. Pinto 2950* (COL!). **Risaralda**: Mpio. de Pereira. Parque Regional Ucumari. Entre El Cedral y La Pastoral. Bosque y potrero cerca del camino, 2,300 m, June 14, 1989, *R. Bernal et al. 1648* (COL!) (Fig. 17).

*Notes*: This Colombian endemic species can be characterized by the presence of 1-veined petals, which are densely and softly pubescent along both margins except base and apex. In this respect *C. zarucchii* is similar to *C. antioquiensis*. Both can be distinguished by petals and lip shape. Petals of *C. zarucchii* are densely and softly pubescent (vs. densely and softly pubescent-papillate), and lip is elliptic-ovate in outline, widest just below the middle, apex truncate with short, triangular, acute apiculus, margins somewhat wavy at base (vs. elliptic-obovate in outline, widest near the middle, apex truncate to rounded). The lateral sepals of *C. zarucchii* can be either 1- or rarely 3-veined.

*Cranichis zarucchii* appears to be similar to both *C. engelii* and *C. cylindrostachys*. This species is easily distinguishable from the former by having 3-veined dorsal sepal (vs. 1-veined), petals with marginal cilia shorter than half width of petals (vs. cilia almost as long as width of petals) and lip margins somewhat wavy at the base only (vs. lip margins prominently wavy). *C. cylindrostachys* has 1-veined dorsal sepal, obscurely 2-veined lateral sepals, petals linear-lanceolate with glabrous margins and oblong-elliptic lip.

### *Engelii* group

Lip more or less obovate in general outline, widest above the middle, rather flat, occasionally gibbose at base, veins thickened, dendritic branching, without any nodules. Petals more or less glandular along both margins.

Only one representative of this group occurs in Colombia.

***Cranichis engelii*** Rchb.f., Linnaea 41: 19. 1876. TYPE: Venezuela. *Engel s.n*. (lectotype, designated by Garay (1978: 196) W!; AMES!—drawing).

**Figure 26:**
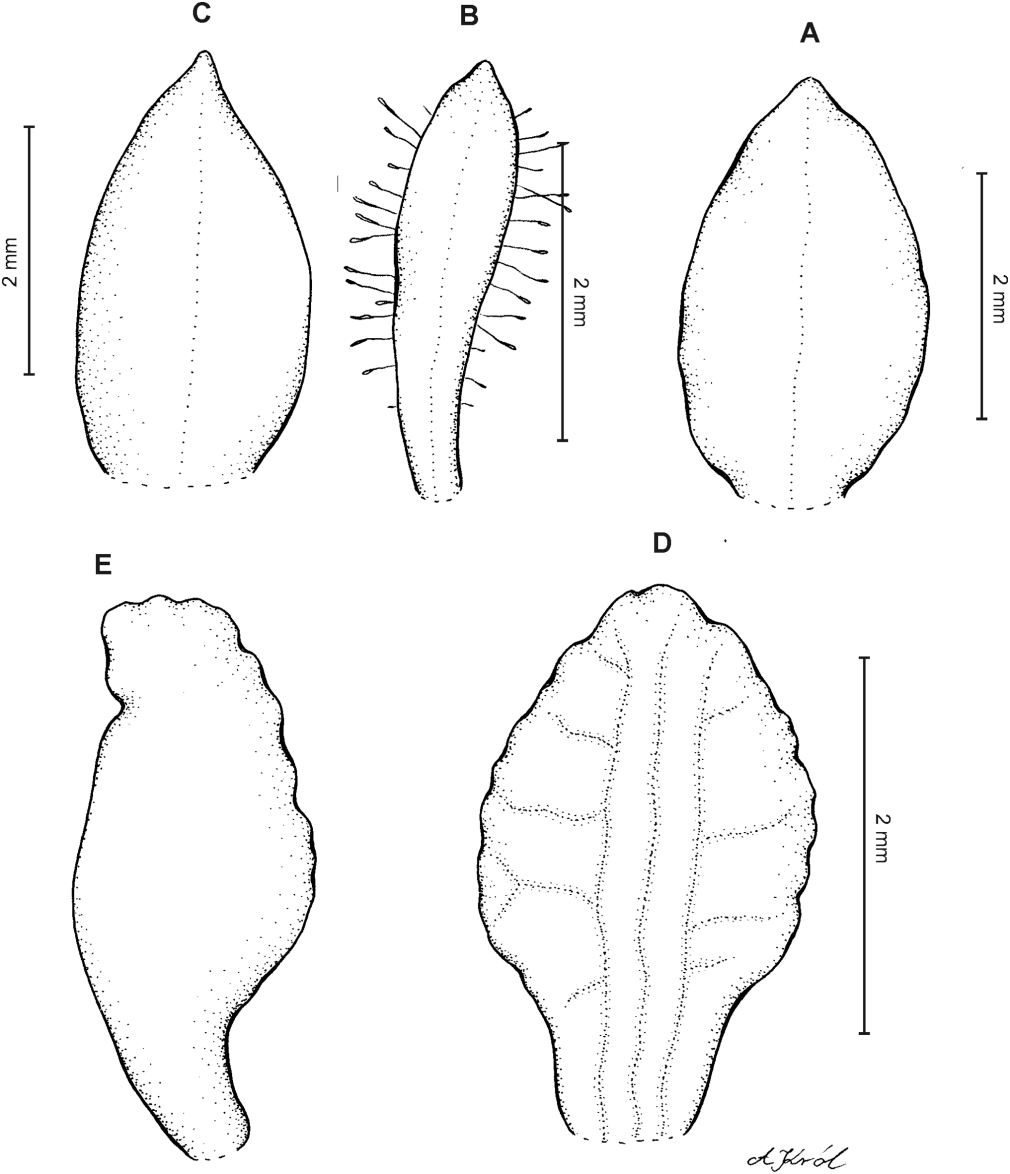
*Cranichis engelii* Rchb.f. (A) Dorsal sepal; (B) petal; (C) lateral sepal; (D) lip (front view); (E) lip (side view). Drawn by A. Król from *Bruchmuller s.n*. (W-R).

**Figure 27:**
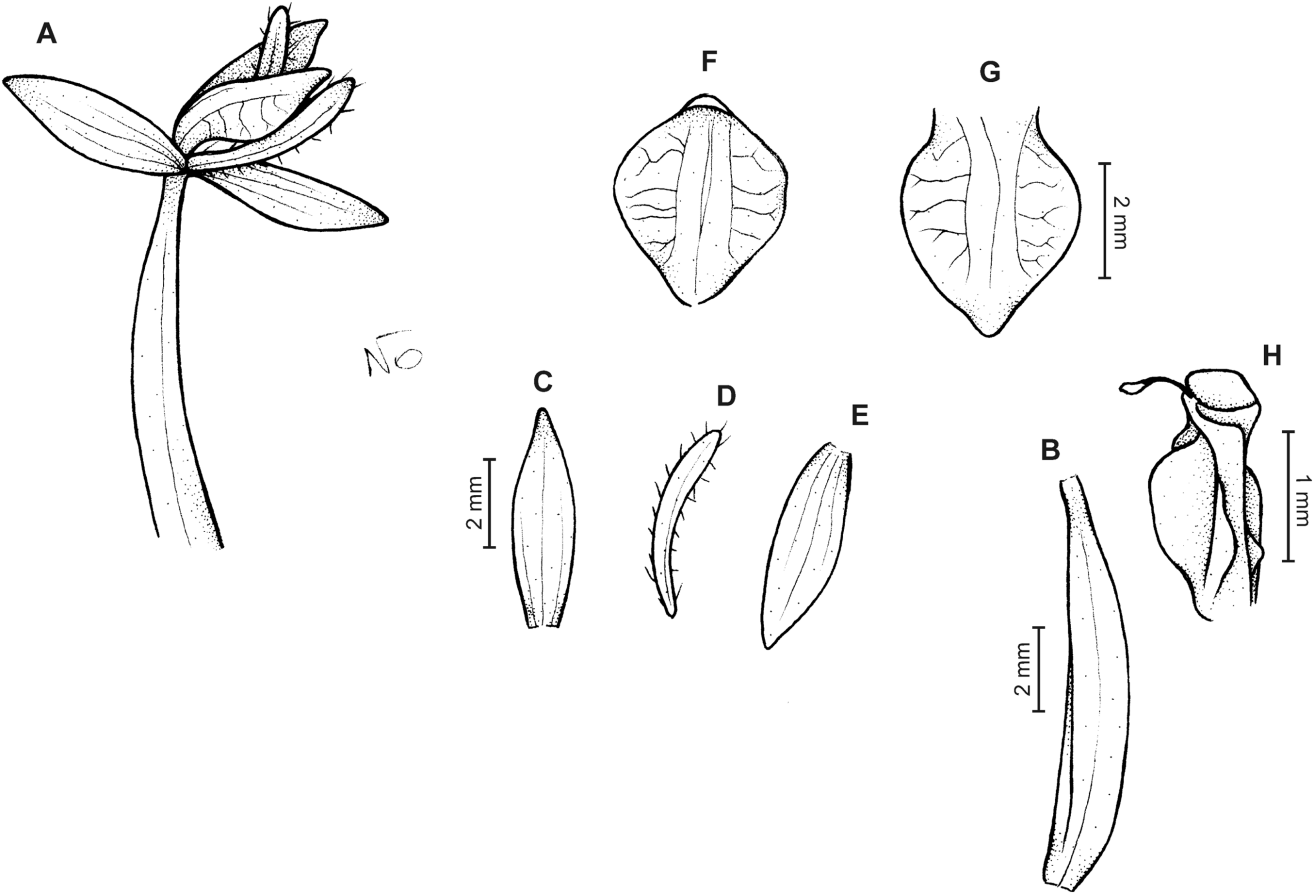
*Cranichis engelii* Rchb.f. (A) Flower; (B) ovary; (C) dorsal sepal; (D) petal; (E) lateral sepal; (F–G) lip (front view); (H) gynostemium. Redrawn by N. Olędrzyńska from Garay’s illustration of a specimen collected by *Engel s.n*. (W).

Plants to 35 cm tall. Leaves 2–3, gathered in a basal rosette, petiolate; petiole 4–14 cm long, narrow, canaliculate; blade 7–11.5 cm long and four to seven cm wide, obliquely ovate-cordate, base cuneate, acute to shortly acuminate. Scape erect, delicate, enclosed in about five to seven sheaths, glabrous below, sparsely pubescent above. Inflorescence up to seven cm long, cylindrical, rather laxly many-flowered. Flowers small, greenish-white, lip with dark green venation. Floral bracts five to six mm long, lanceolate, acuminate. Pedicellate ovary 8–10 mm long, glabrous. Dorsal sepal 2.5–5 mm long, 1.2–1.5 mm wide, oblong-lanceolate to oblong-elliptic, acute to subacute, 3-, occasionally 1-veined. Petals 2.5–4 mm long, 0.5–0.7 mm wide, obliquely oblong-oblanceolate to linear-oblanceolate, obtuse to acute, glandular along margins. Lateral sepals three to five mm long, 1.5–2.3 mm wide, obliquely oblong-elliptic to elliptic, subobtuse to subacute, 3-, occasionally 1-veined. Lip 2.5–4 mm long and wide, cochleate, subsessile, suborbicular to broadly ovate, rounded or truncate at the apex; disc 3-veined, with the midvein unbranched, lateral veins branching, without any nodules. Gynostemium two mm long (Figs. 26 and 27).

*Ecology*: Terrestrial plant. No data on habitat or flowering time in Colombia.

*Distribution*: Ecuador (Dodson & Luer, 2005), Colombia, Venezuela.

**Figure 28:**
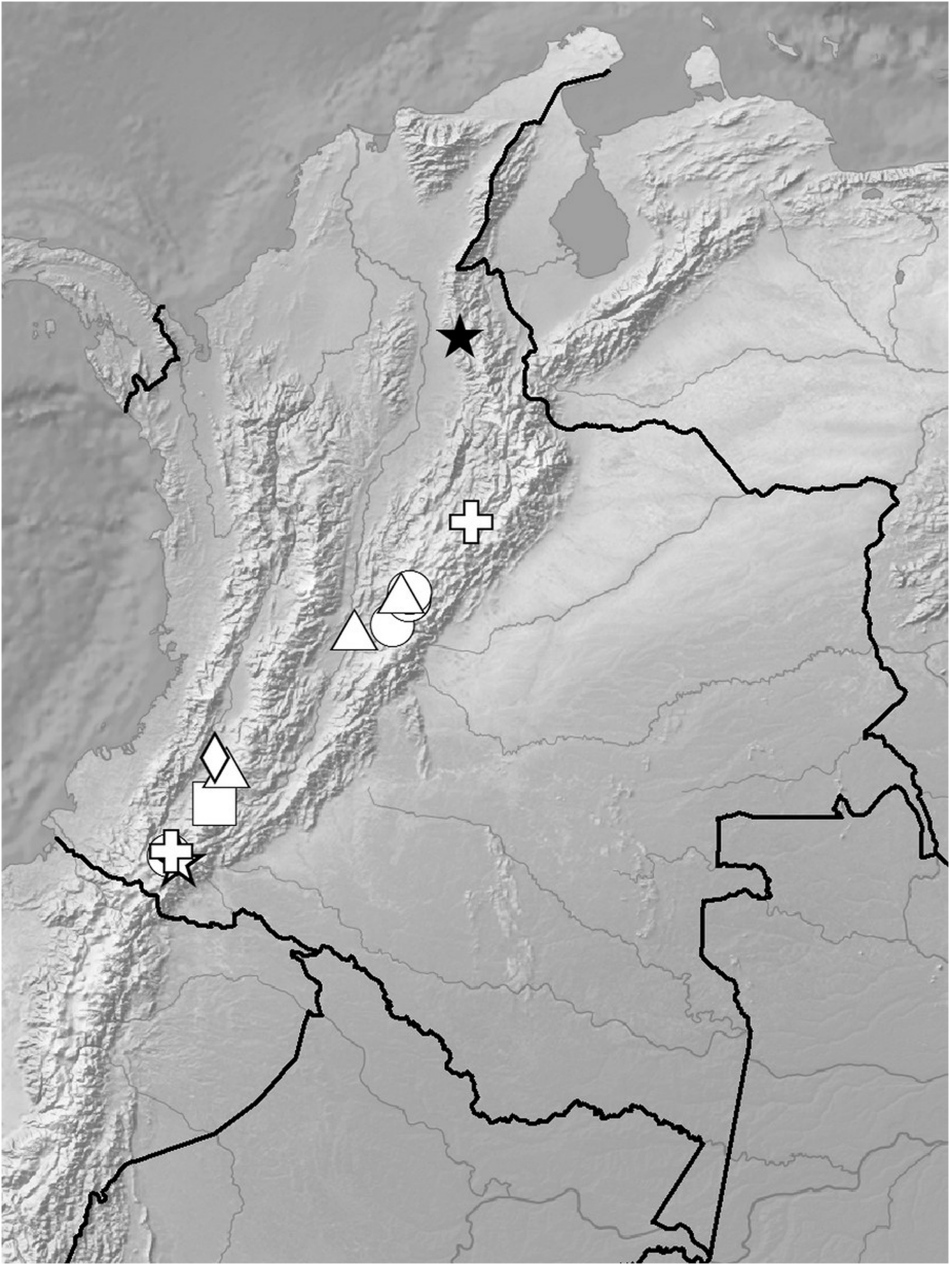
Distribution of members of the *Cranichis Engelii* group and *Badia* group. *C. engelii* (black star), *C. badia* (circle), *C. juajibioyi* (square), *C. neglecta* (triangle), *C. pennellii* (diamond), *C. rotundifolia* (white star), *C. silvicola* (cross).

*Representative specimens*: COLOMBIA. **Norte de Santander**: *Sine loc*. (Schlechter, 1920); Ocaña, *A. Bruchmuller s.n*. (W-R!, UGDA-DLSz!—drawing). *Sine loc., G. Giraldo 66C-20* (COL!, UGDA-DLSz!—drawing) (Fig. 28).

*Other materials examined*: VENEZUELA. **Mérida**: *F. Engel s.n*. (AMES!—drawing, W!).

*Notes*: This species has been reported from Ecuador, Colombia, and Venezuela and it resembles *C. elliptica* Rchb.f. but has glabrous ovary and glandular petal margins without papillae between glandulae.

### *Badia* group

Lip obovate, suborbicular to elliptic in general outline, rather flat, occasionally somewhat gibbose at base, veins thickened, dendritic branching. Petals glabrous along both margins.

Six Colombian species belong to this group.

## Key to the Species

1. Lip widest near the middle or above2
1* Lip widest below the middle, attenuate toward apex4
2. Dorsal sepal 3(5)-veined3
2* Dorsal sepal 1-veined*C. neglecta*
3. Floral bracts equal in length to pedicellate ovary, lateral sepals 2- or 3-veined, lip suborbicular to obovate in outline from the cuneate base, margins somewhat undulate*C. silvicola*
3* Floral bracts distinctly shorter than pedicellate ovary, lateral sepals 3-veined, lip unguiculate, elliptic-subrhombic above*C. badia*
4. Lip with 2, dendritic veins*C. rotundifolia*
4* Lip with 3 veins, both laterals branching5
5. Leaf variegated, lip with flat margins*C. juajibioyi*
5* Leaf plain green, lip with undulate margins*C. pennellii*

***Cranichis badia*** Renz *ex* Kolan. & Szlach., Nordic J. Bot. 32(3): 289. 2014. TYPE: Venezuela. *O. Renz 6065* (holotype, RENZ!; isotypes, RENZ!).

**Figure 29:**
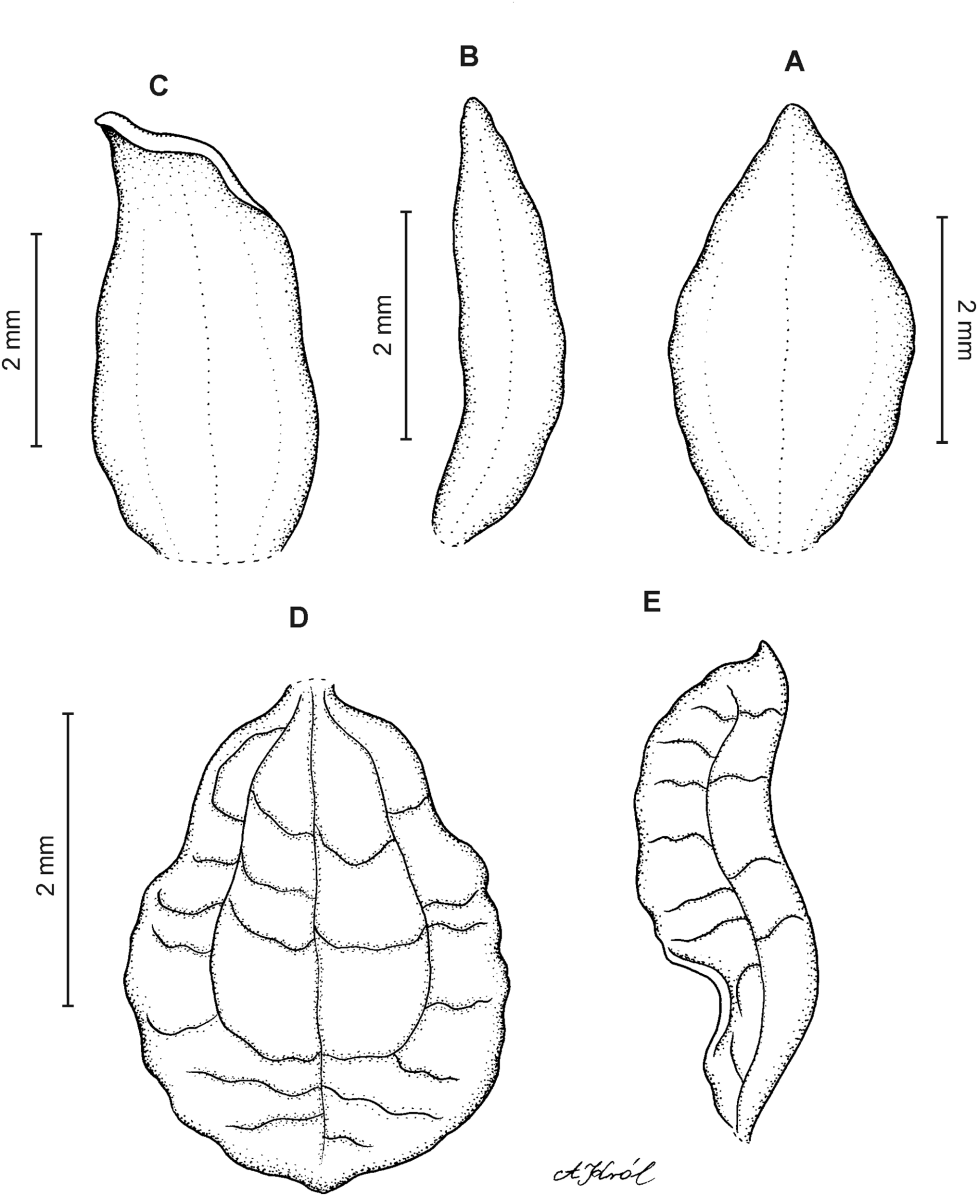
*Cranichis badia* Renz *ex* Kolan. & Szlach. (A) Dorsal sepal; (B) petal; (C) lateral sepal; (D) lip (front view); (E) lip (side view). Drawn by A. Król from *Lehmann 8173* (K).

**Figure 30:**
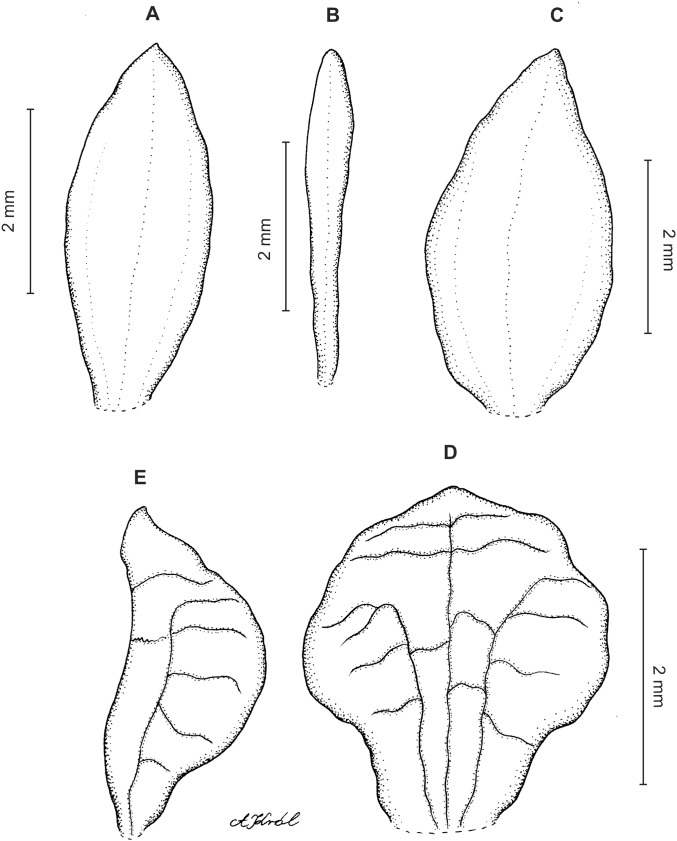
*Cranichis badia* Renz *ex* Kolan. & Szlach. (A) Dorsal sepal; (B) petal; (C) lateral sepal; (D) lip (front view); (E) lip (side view). Drawn by A. Król from *Andre 2222* (K).

Plants up to 50 cm tall, erect. Leaves usually 1, but two to three sometimes present, basal, petiolate; petiole 5–15 cm long, narrow, canaliculate; blade 4–12 cm long and two to six cm wide, elliptic to ovate-elliptic, acuminate, cuneate at the base. Scape up to 32 cm long, enclosed in four to five sheaths. Inflorescence up to 15 cm long, cylindrical-conical, subdensely many-flowered. Flowers small, brown or whitish, lip white with brown venation. Floral bracts five to eight mm long, lanceolate, acuminate, glabrous or almost glabrous. Pedicellate ovary 7–10 mm long, glabrous to rarely glandular. Dorsal sepal 3.5–4.5 mm long, 1.8–2.3 mm wide, elliptic to oblong-ovate, subacute to subobtuse, 3-, occasionally 5-veined. Petals 3.2–4 mm long, 0.4–0.8(1.2) mm wide, linear, almost filiform, apex rounded, margins glabrous, 1-veined. Lateral sepals 3.5–4.5 mm long, 2–2.8 mm wide, obliquely oblong-elliptic to elliptic, subobtuse to subacute, 3-veined. Lip up to about four mm long, 2.5–4 mm wide, somewhat concave, almost flat, unguiculate, elliptic-subrhombic above, lateral margins recurved, subacute to obtuse at the apex; disc 3-veined, midvein often anastomozing, lateral veins strongly branching, without any nodules. Gynostemium 1.6–2 mm long (Figs. 29 and 30).

*Ecology*: In Colombia it was found growing terrestrially at altitudes from 2,700 to 3,200 m. Flowering occurs in May, October, November, and December.

*Distribution*: Ecuador, Colombia, Venezuela.

*Representative specimens*: COLOMBIA. **Cundinamarca**: Bogotá. Quebrada del Chicó, 2,700–2,750 m, December 27, 1943, *M. Schneider 172/5* (RENZ!); Bogotá, Usme, sector del Embalse de Chisacá, vereda Las Margaritas, 4°20N 74°15W, 3,000–3,200 m, November 20, 2005, *Y. Figueroa-C., J. Mora & T. Vivas 867* (COL); Alrededores de Bogotá. Quebrada de El Chicó, 2,700–2,800 m, 18 May 1944, *M. Schneider 11/1* (COL!, UGDA-DLSz!—drawing); Usaquén, 3,000 m, October 30, 1949, *M. Schneider 253* (COL!); *Sine loc. J. Ordonez et al. 1566* (COL!, UGDA-DLSz!—drawing). *Sine loc., F.C. Lehmann 8173* (K!, UGDA-DLSz!—drawing). **Nariño**: Pasto, 3,000 m, October 25, 1878, *F.C. Lehmann 1 (or s.n.)* (W-R!, UGDA-DLSz!—drawing) (Fig. 28).

*Other materials examined*: ECUADOR. **Bolivar**: Seven km E of Balsapamba on road to San José de Chimbo, region of El Torneado, 1,400 m, June 16, 1960, *C. Dodson 98* (RPSC!, UGDA-DLSz!—drawing). **Cotopaxi**: Road Quevedo-Latacunga. Macuchi, 1,600 m, May 1, 1968, *G. Harling et al. 8831* (AMES!, UGDA-DLSz!—drawing). **Pichincha**: Mt Pasochoa, cerca de Aloag, carretera Quito-Machachi, 2,900–3,200 m, May 1, 1985, *C. Dodosn & A. Hirtz 15816* (RPSC!, UGDA-DLSz!—drawing). **Tungurahua**: Road Patate to Leito to Llanganates Range, 3,000 m, April 12, 1985, *A. Hirtz et al. 2490* (RPSC!). **Loja** Beyond the pass, Yangana to Valladolid, 2,500–3,000 m, April 1985, *D. Dallesandro 0390* (RPSC!). *Sine loc*., *M.E. André 2222* (K!, UGDA-DLSz!—drawing). VENEZUELA. **Mérida**: Sierra Nevada. Alrededores de La Laguna Coromoto, December 2, 1959, *H.G. Barclay & P. Juajibioy 9933* (COL!). **Tachira**: 2,880 m, November 23, 1949, *O. Renz 6174* (RENZ!), 2,900 m, November 23, 1949, *O. Renz 6175* (RENZ!).

*Notes*: This species is similar to its Colombian-Venezuelan congener, *C. silvicola*, unlike the latter, however, its floral bracts are equal in length to the pedicellate ovary (vs. floral bracts shorter than pedicellate ovary), and its lip is unguiculate, elliptic-subrhombic above (vs. lip suborbicular to obovate in outline from the cuneate base, margins somewhat undulate). Lip lateral veins are much stronger branched in *C. badia* than in *C. silvicola*.

***Cranichis juajibioyi*** Szlach. & Kolan., ***sp. nov***. TYPE: Colombia. *Barclay & Juajibioy 5878* (holotype, AMES!; v MO!, UGDA-DLSz!—drawing).
*Species distinguished from C. rotundifolia by spathulate petals and 3-veined lip that is attenuate toward the apex*.

**Figure 31:**
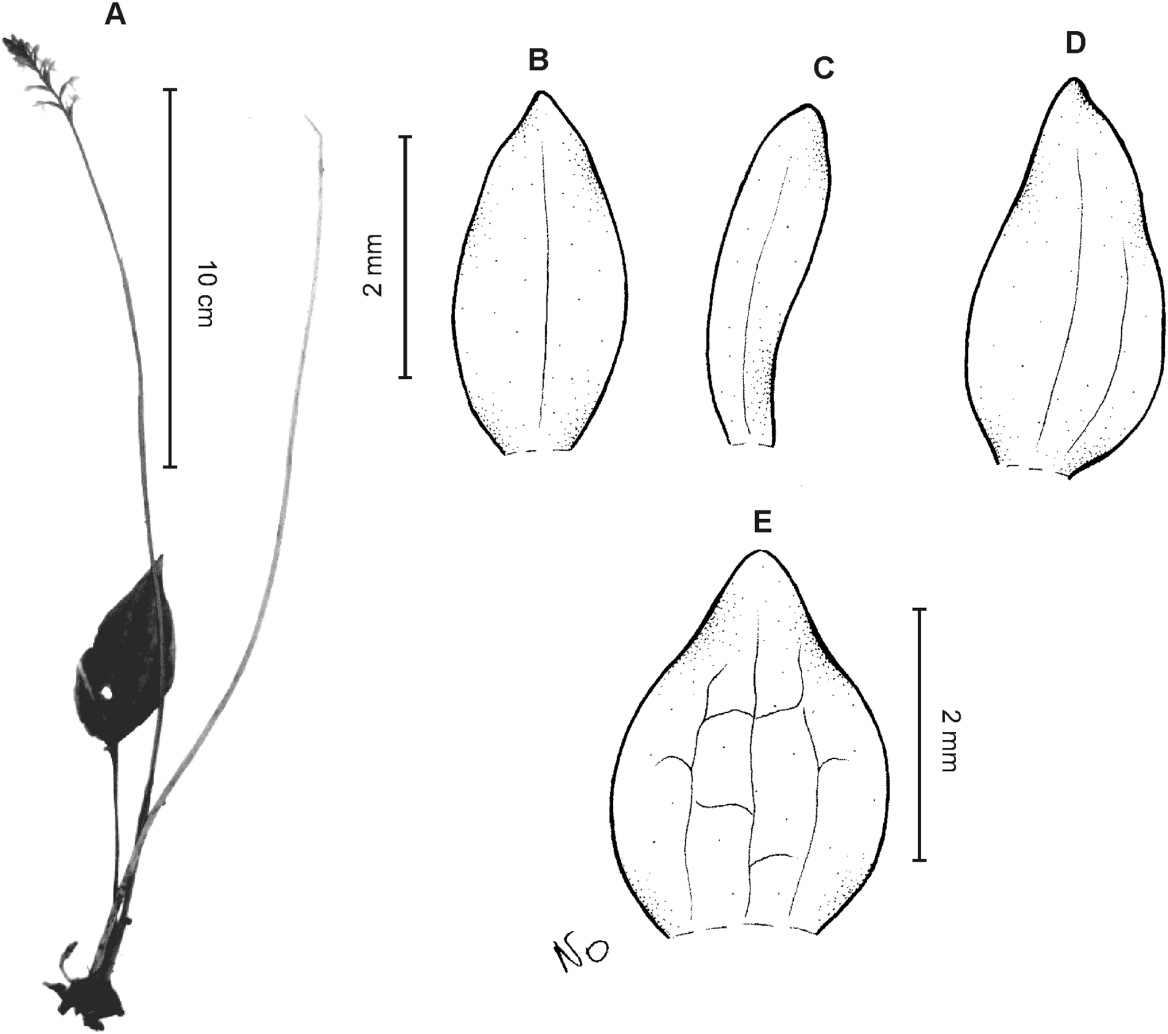
*Cranichis juajibioyi* Szlach. & Kolan. (A) Habit; (B) dorsal sepal; (C) petal; (D) lateral sepal; (E) lip (front view). Drawn by N. Olędrzyńska from *Barclay & Juajibioy 5878* (MO). Scale bar two mm for (A–C).

Plants 26 cm tall. Leaf single, variegated, petiolate; petiole 6.5 cm long, narrow; blade 5.5 cm long, 2.5 cm wide, ovate, acute, slightly undulate on margin. Scape delicate, enclosed in four sheaths, glandular below rachis. Inflorescence three cm long, conical, rather laxly many-flowered. Flowers almost transparent, lip spotted, glabrous. Floral bracts four mm long, glabrous. Pedicellate ovary four mm long, sparsely glandular. Dorsal sepal three mm long, 1.2 mm wide, elliptic, obtuse, 1-veined. Petals three mm long, 0.8 mm wide, ligulate, obtuse, margins glabrous, 1-veined. Lateral sepals 3.5 mm long, 1.5 mm wide, obliquely ovate, obtuse, 2-veined. Lip flat, subsessile, three mm long, 1.8 mm wide, broadly ovate, attenuate toward the apex; disc with three anastomozing veins. Gynostemium 1.2 mm long (Fig. 31).

*Etymology*: Named in honor of the co-collector of the type specimen.

*Ecology*: Terrestrial plant growing in wet shrubs within *Espeletia* at an altitude of 3,150 m. Flowering occurs in September and October.

*Distribution*: Colombia.

*Representative specimens*: COLOMBIA. **Cauca**: Macizo Colombiano. Valle de Las Papas. Alrededores de Valencia, Los Andes. Second shrubby patch occurring as an island in *Espeletia*-grass cienaga (wet area), somewhat S of first, at adge of quebrada, 3,150 m, September 25–October 19, 1958. *H.G. Barclay & P. Juajibioy 5878* (AMES!, MO!, UGDA-DLSz!—drawing) (Fig. 28).

*Notes*: This species can be distinguished from *C. rotundifolia* by having spathulate (vs. ligulate) petals, 3-veined (vs. 2-veined), attenuate toward the apex lip. It is similar to *C. pennellii*, but has variegated leaf, with flat margins and lip without any undulation. From *C. badia* the new species differs in 1-veined dorsal sepal (vs. 3- or 5-veined), ligulate (vs. almost filiform) petals, 2-veined lateral sepals (vs. 3-veined) and subsessile (vs. unguiculate) lip.

***Cranichis neglecta*** Szlach. & Kolan., ***sp. nov***. TYPE: Colombia. *Pennell & Killip 6470* (holotype, AMES!, UGDA-DLSz!—drawing).
*Species distinguished from C. silvicola by having 1-veined dorsal sepal*.

**Figure 32:**
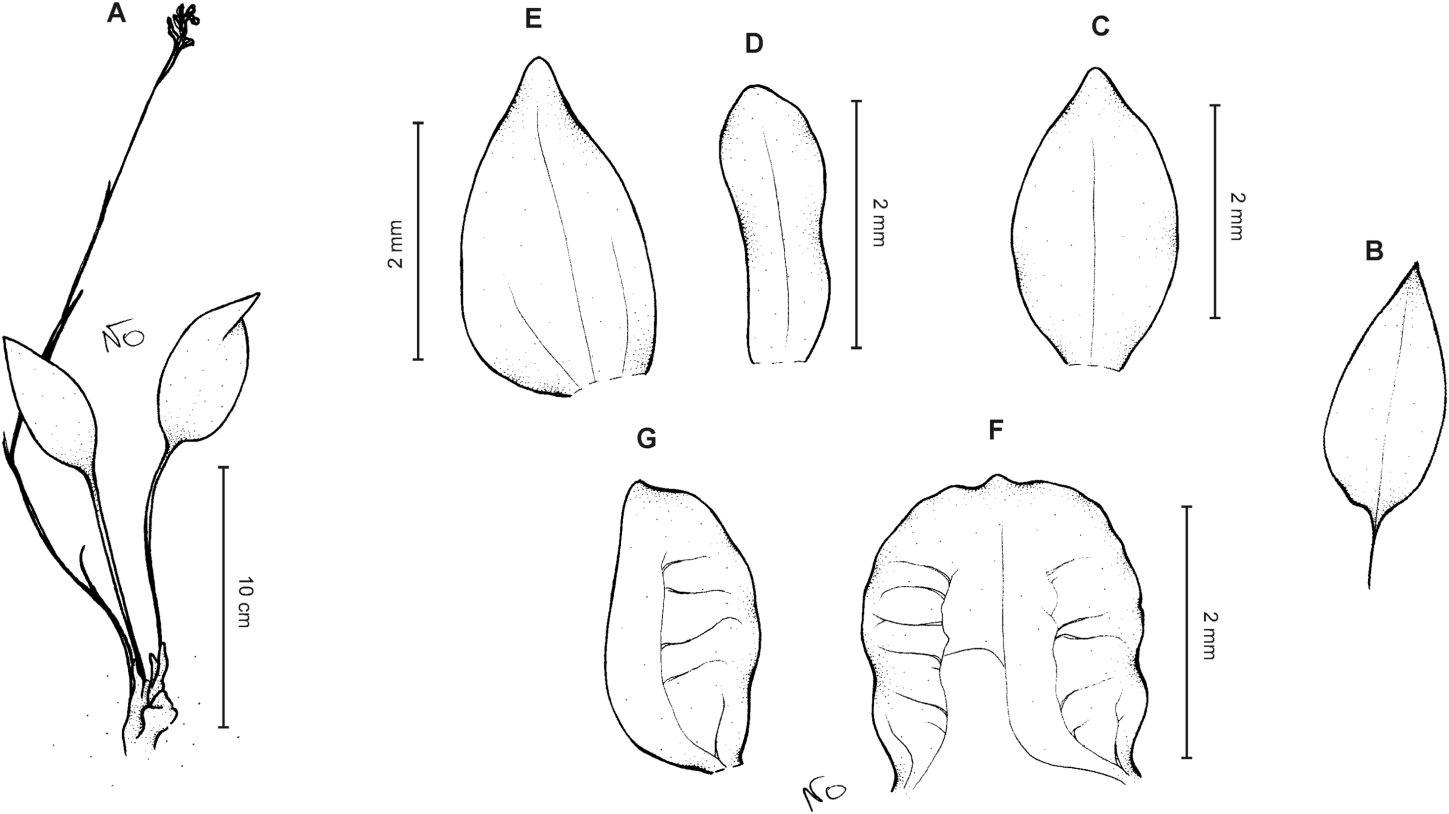
*Cranichis neglecta* Szlach. & Kolan. (A) Habit; (B) leaf; (C) dorsal sepal; (D) petal; (E) lateral sepal; (F) lip (front view); (G) lip (side view). Drawn by N. Olędrzyńska from *Pennell 2367* (NY). Scale bar for (F–G) = 2 mm.

Plants 23–48 cm tall. Leaves 1–3, gathered in the basal rosette, petiolate; petiole 6.5–13 cm long, narrow, canaliculate; blade 5–10.5 cm long, 2.2–4 cm wide, ovate-lanceolate or elliptic-lanceolate, acute acuminate, cuneate at the base. Scape erect, enclosed in about five to six sheaths. Inflorescence up to nine cm long, cylindrical, rather densely many-flowered. Floral bracts four to seven mm long, lanceolate, acuminate, glabrous. Pedicellate ovary 4.5–10 mm long, glabrous. Dorsal sepal 2.6–4.5 mm long, 1.2–1.8 mm wide, narrowly elliptic-ovate to elliptic-lanceolate, subacute to subobtuse, 1-veined. Petals 2.3–4.3 mm long, 0.5–1 mm wide, oblong-ligulate, falcate, obtuse, margins glabrous, 1-veined. Lateral sepals 2.8–4.5 mm long, 1.5–2.5 mm wide, obliquely ovate to ovate-ligulate, obtuse, obscurely 2- or 3-veined. Lip about 2.2–4 mm long, 2.2–3 mm wide, cochleate, subsessile, from the cuneate base suborbicular to obovate, almost flat, rounded to subacute at the apex, margins somewhat undulate; disc with three branching veins, without any nodules. Gynostemium 1.4–2 mm long (Fig. 32).

*Etymology*: The specific epithet “neglecta” has been chosen to reflect that this species, despite having been previously collected and studied, has been long neglected.

*Ecology*: Terrestrial in montane forest and stream banks at an altitude of 2,200–3,000 m. Flowering occurs in June.

*Distribution*: Colombia.

*Representative specimens*: COLOMBIA. **Cauca**: San Isidro, Puracé. Cordillera Central, 2,200–2,500 m, June 10–11, 1922, *F. Pennell & E. Killip 6470* (AMES!, UGDA-DLSz!—drawing). **Cundinamarca**: Buenavista, northeast of Bogotá, 2,900–3,000 m, *F. Pennell 2367* (NY!, UGDA-DLSz!—drawing); Ricaurte. *F.C. Lehmann BT71* (NY!, UGDA-DLSz!—drawing) (Fig. 28).

*Notes*: This species can be distinguished by suborbicular to obovate lip with cuneate base, 1-veined dorsal sepal and obscurely 2-veined lateral sepals. *C. neglecta* can be easily distinguished from *C. diphylla* by having glabrous ovary (vs. more or less glandular), 1-veined dorsal sepal (vs. 3-veined), and suborbicular to obovate lip from the cuneate base, almost flat, margins somewhat undulate, with disc devoid of nodules (vs. lip subcordate at base, ovate to broadly elliptic in outline, margins flat, disc obcordately papillose-thickened with three branching, often glandular veins from the base to the middle of the lip).

The other specimen from the type collection of *C. neglecta* represents *C. diphylla*.

***Cranichis pennellii*** Szlach. & Kolan., ***sp. nov***. TYPE: Colombia. *Pennell 7469* (holotype, US AMES!; isotypes, US NY!, US!; UGDA-DLSz!—drawing).
*Species distinguished from C. juajibioyi in having undulate leaf and lip margins, obscurely 3-veined lateral sepals, shortly unguiculate lip with three veins, the lateral ones branching*.

**Figure 33:**
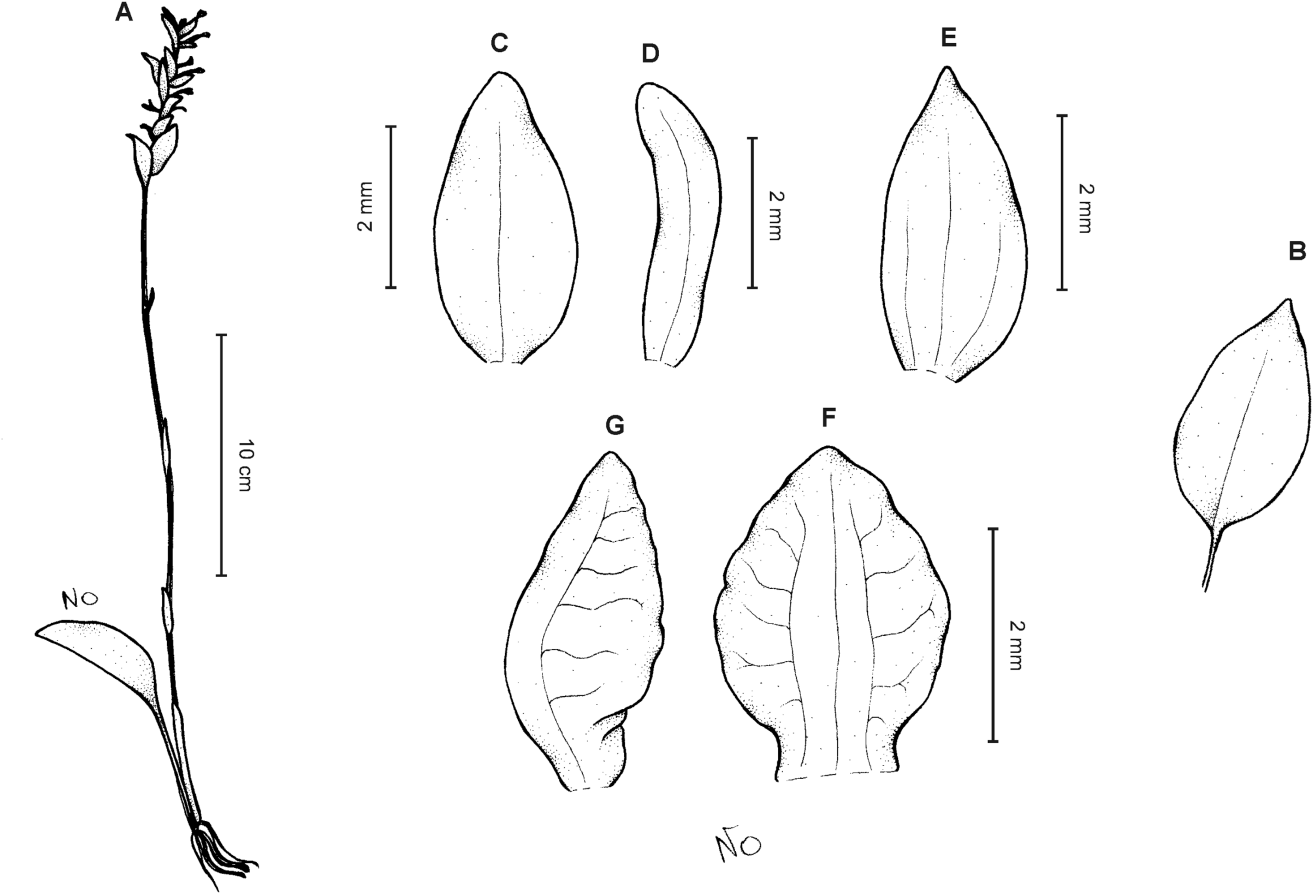
*Cranichis pennellii* Szlach. & Kolan. (A) Habit; (B) leaf; (C) dorsal sepal; (D) petal; (E) lateral sepal; (F) lip (front view); (G) lip (side view). Drawn by N. Olędrzyńska from *Pennell 7469* (NY). Scale bars for (F–G) = 2 mm.

Plant 34 cm tall. Leaf single, petiolate; petiole five cm long, narrow, canaliculate; blade four cm long, 1.8 cm wide, ovate, subobtuse, margin undulate. Scape delicate, sparsely glandular in the upper part, enclosed in three to four sheaths. Inflorescence 4.5–5.5 cm long, cylindrical, subdensely many-flowered. Flowers greenish-yellow. Floral bracts five mm long, glabrous. Pedicellate ovary six mm long, glabrous. Dorsal sepal 3.8 mm long, 1.6 mm wide, ovate, obtuse, 1-veined. Petals 3.2 mm long, 0.6 mm wide, ligulate, obtuse, falcate, margins glabrous, 1-veined. Lateral sepals 3.6 mm long, 1.6 mm wide, obliquely ovate, obtuse, obscurely 3-veined. Lip 3.1 mm long, 2.3 mm wide, somewhat cochleate, subsessile, ovate in outline above short, narrow part, apex obtuse, margin undulate; disc with three veins, lateral ones branching. Gynostemium 1.8 mm long (Fig. 33).

*Etymology*: Named in honor of the collector of the type specimen.

*Ecology*: Terrestrial plant growing in shrub zone (“paramillo) at an altitude of 2,700–3,000 m. Flowering occurs in June.

*Distribution*: Colombia.

*Representative specimen*: COLOMBIA. **Cauca**: Mt Santa Ana. Cordillera Occidental, 2,700–3,000 m, June 29–30, 1922, *F.W. Pennell 7469* (AMES!, NY!, US!, UGDA-DLSz!—drawing) (Fig. 28).

*Notes*: This species is similar to *C. juajibioyi*, but can be distinguished from the latter by having undulate leaf and lip margins, obscurely 3-veined lateral sepals (vs. 2-veined), shortly unguiculate lip (vs. subsessile) with three veins, the lateral ones branching (vs. three anastomozing veins). The new species clearly differs from *C. rotundifolia* by its 3-veined lip.

***Cranichis rotundifolia*** Szlach. & Kolan., Polish Bot. J. 58(2): 620. 2013. TYPE: Colombia. *Benavides 2699* (holotype, PSO!, UGDA-DLSz!—drawing).

**Figure 34:**
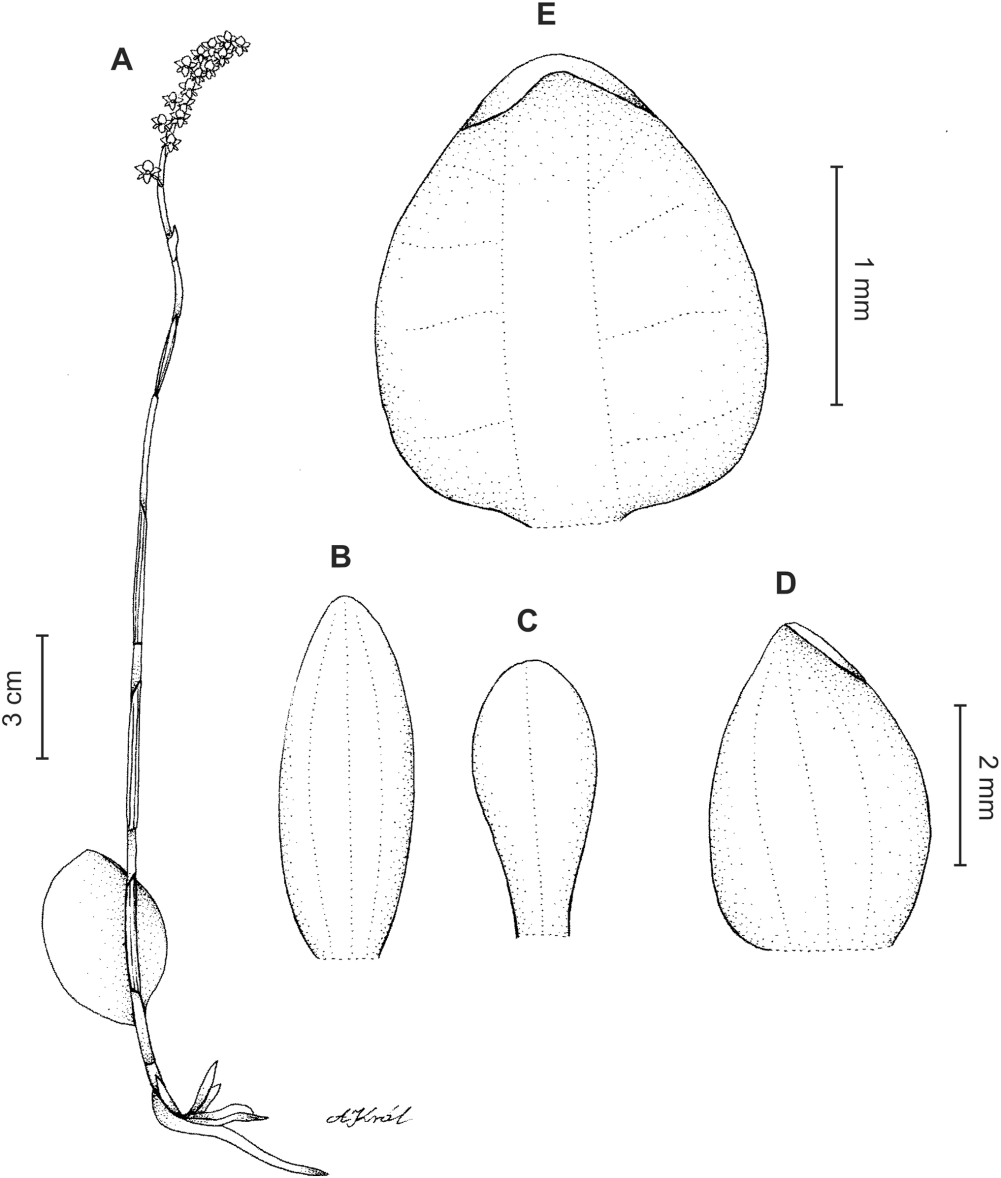
*Cranichis rotundifolia* Szlach. & Kolan. (A) Habit; (B) dorsal sepal; (C) petal; (D) lateral sepal; (E) lip (front view). Scale bar for (B–D) = 2 mm. Drawn by A. Król from *Benavides 2699* (PSO).

Plant up to about 30 cm tall. Leaf 1, basal, petiolate; petiole about three cm long, narrow; blade about five cm long and 3.5 cm wide, suborbicular, subacute, subcordate at the base. Scape about 25 cm long, slender, erect, remotely few-sheathed. Inflorescence about six cm long, cylindrical, densely many-flowered. Flowers translucent white, glabrous. Floral bracts three to four mm long, lanceolate, acute, glabrous. Pedicellate ovary five to six mm long, very sparsely glandular-ciliate. Dorsal sepal 3.1 mm long, one mm wide, narrowly elliptic, obtuse, 3-veined. Petals 2.5 mm long, one mm wide, spathulate, apex rounded, margins glabrous, 1-veined. Lateral sepals 2.9 mm long, 1.5 mm wide, obliquely ovate, subobtuse, 3-veined. Lip about two mm long, 1.6 mm wide, concave, sessile, broadly ovate, widest just above base, obtuse; disc with two anastomosing veins. Gynostemium 1.1 mm long (Fig. 34).

*Ecology*: In Colombia this species grows terrestrially on humid soil in high-montane humid forest at an altitude of 2,700 m. Flowering occurs in November.

*Distribution*: Colombia.

*Representative specimens*: COLOMBIA. **Nariño**: Pasto, Correg. de El Encano. Isla La Corota, 2,700 m, November 14, 1980, *O.S. Benavides 2699* (PSO!, UGDA!—drawing) (Fig. 28).

*Notes*: Species similar to *C. diphylla* but distinguished by its suborbicular leaf blade (vs. ovate to ovate-lanceolate), the spathulate, apically rounded petals (vs. linear-oblanceolate, acute to obtuse) and the lip broadly ovate and obtuse, with two anastomosing veins (vs. lip ovate to broadly elliptic, disc obcordately papillose-thickened with three branching, often glandular veins). Vegetatively the new species seems to resemble *C. tenuis*, from which it differs by the form of its perianth segments as well as by the shorter floral bracts and ovaries. In the floral parts *C. rotundifolia* resembles *C. fendleri*, especially in having spathulate petals and a broadly ovate lip. The two species are easily distinguished by a series of characters. In *C. fendleri* there are three to four oblong-elliptic to ovate-lanceolate, acute leaves, an oblong-lanceolate dorsal sepal, and a lip with three anastomosing veins.

***Cranichis silvicola*** Renz *ex* Kolan. & Szlach., Nordic J. Bot. 32(3): 296. 2014. TYPE: Venezuela. *Renz 6139* (holotype, RENZ!).

**Figure 35:**
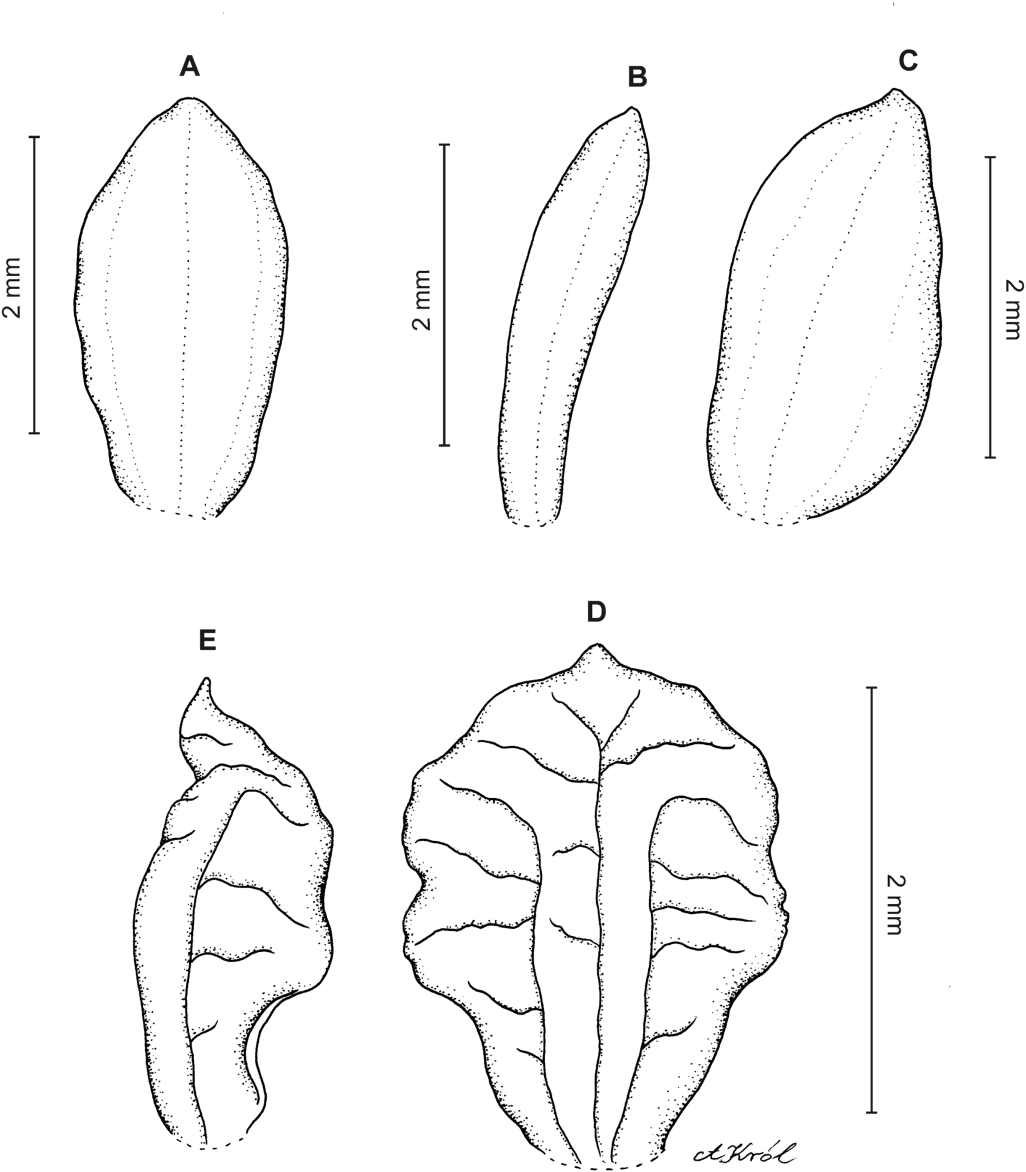
*Cranichis silvicola* Renz *ex* Kolan. & Szlach. (A) Dorsal sepal; (B) petal; (C) lateral sepal; (D) lip (front view); (E) lip (side view). Drawn by A. Król from *Lehmann 8505* (K).

Plants up to 50 cm tall. Leaves 1–2, gathered in the basal rosette, petiolate; petiole 12–15 cm long, narrow, canaliculate; blade 6.8–10.5 cm long, 3.4–4.5 cm wide, ovate-lanceolate or elliptic, acute or shortly acuminate, cuneate at the base. Scape up to 40 cm long, erect, enclosed in about five sheaths. Inflorescence up to eight cm long, cylindrical, rather laxly many-flowered. Floral bracts five to eight mm long, lanceolate, acuminate, glabrous. Pedicellate ovary five to seven mm long, glabrous. Dorsal sepal 2.8–4 mm long, one to two mm wide, narrowly elliptic-obovate to elliptic, obtuse to subobtuse, 3-veined. Petals 2.8–4 mm long, 0.4–0.9 mm wide, oblong-ligulate, obtuse, margins glabrous, 1-veined. Lateral sepals three to four mm long, 1.3–2 mm wide, obliquely oblong-ovate, obtuse, 2- or 3-veined. Lip about 3–3.2 mm long, 2.4–3 mm wide, cochleate, subsessile, from the cuneate base suborbicular to obovate, almost flat, rounded to subacute at the apex, margins somewhat undulate; disc with three branching veins. Gynostemium 1.5–2.1 mm long (Fig. 35).

*Ecology*: Terrestrial plant growing on weedy roadsides at altitudes of 1,750–3,300 m. Flowering occurs in November and December.

*Distribution*: Colombia, Venezuela.

*Representative specimens*: COLOMBIA. **Boyacá**: Mun. Sotaquira, 5°43′55.9″ 73°10′30.8″, 2,620 m, December 13, 2003, *M. Cordoba et al. 3143* (COL!, UGDA-DLSz!—drawing). **Cauca**: 1,750 m. *F.C. Lehmann 8505* (K!, RENZ!—p.p., UGDA-DLSz!—drawing). **Nariño**: On weedy roadsides. Three km NE of Pasto, 3,300 m, July 21, 1974, *C. Sheviak et al. CS825* (AMES!, UGDA-DLSz!—drawing) (Fig. 28).

*Other materials examined*: VENEZUELA. Mérida, 1,500 m, November 18, 1949, *O. Renz 6141* (RENZ!).

*Notes*: This species can be confused with *C. badia*, but has 2-veined lateral sepals.

Plant similar to *C. ciliata* as well, from which it differs, however, by the narrowly elliptic, obtuse dorsal sepal (vs. dorsal sepal ovate, apiculate), narrower lateral sepals, lip cuneate at the base, and floral bracts subequal to the ovary (vs. floral bracts distinctly shorter than ovary).

### *Polyantha* group

Lip deeply cochleate, helmet-like, suborbicular to elliptic when spread, veins usually not thickened, dendritic branching, without any nodules. Petals usually glabrous along both margins.

Four Colombian species belong to this group.

## Key to the Species

1. Petals 3-veined*C. brevirostris*
1* Petals 1-veined2
2. Leaf petiole up to 18 cm long*C. longipetiolata*
2* Leaf petiole much shorter, up to eight cm3
3. Ovary glandular, petal margins entire*C. polyantha*
3* Ovary glabrous, petal margins erose*C. calva*

***Cranichis brevirostris*** Renz *ex* Kolan. & Szlach., Nordic J. Bot. 32(3): 292. 2014. TYPE: Venezuela. *Renz 5960* (holotype, RENZ!; isotypes, RENZ!).

**Figure 36:**
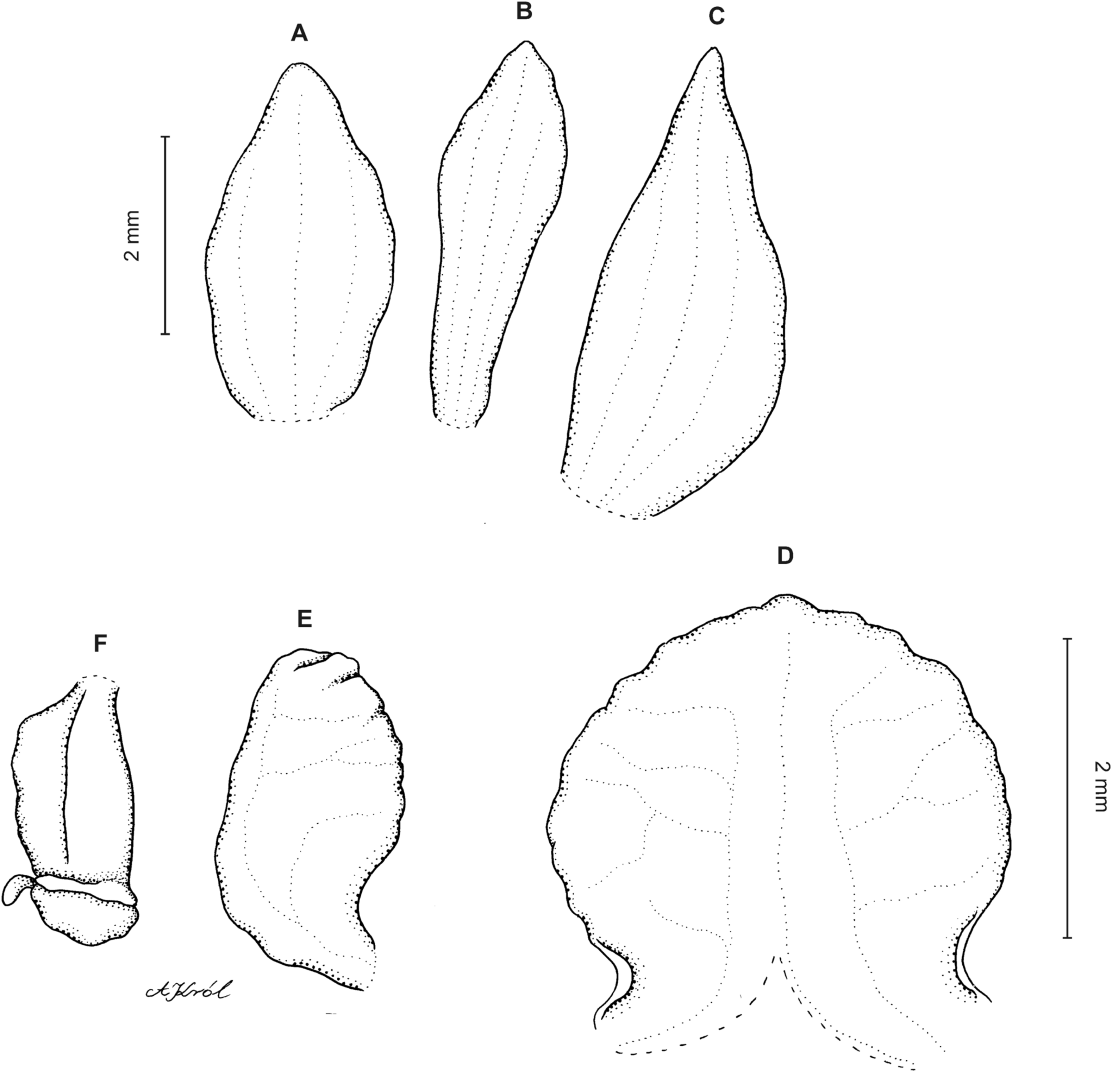
*Cranichis brevirostris* Renz *ex* Kolan. & Szlach. (A) Dorsal sepal; (B) petal; (C) lateral sepal; (D) lip (front view); (E–F) lip (side view). Drawn by A. Król from *Lehmann 5885* (K). Scale bar for (A–C) = 2 mm.

Plants up to 35 cm tall, erect. Leaves 1–2, gathered in a basal rosette, petiolate; petiole 3.75–12 cm long, narrow, canaliculate; blade 2.6–11 cm long and 3.25–4.75 cm wide, elliptic to ovate, acute or shorty acuminate, cuneate at the base, margins crenate. Scape up to 28 cm long, remotely few-sheathed. Inflorescence 2.5–5.75 cm long, conical, subdensely many-flowered. Floral bracts seven to eight mm long, lanceolate, acuminate, almost glabrous. Pedicellate ovary five to seven mm long, glabrous. Dorsal sepal 2.7–3.6 mm long, 1.8–2.1 mm wide, oblong-ovate to elliptic, obtuse, 3-veined. Petals 2.8–3.6 mm long, 0.8–1.1 mm wide, oblong-oblanceolate, obtuse, margins entire, glabrous, 3-veined. Lateral sepals 2.7–4.2 mm long, 2–2.3 mm wide, obliquely ovate, attenuate toward an obtuse apex, 3-veined. Lip up to 2.2–3 mm long, two to three mm wide, cochleate, gibbose at base, subsessile, suborbicular, subacute to obtuse at the apex; disc 3-veined, lateral veins branching, without any nodules. Gynostemium 0.9–1.1 mm long (Fig. 36).

*Ecology*: No data on habitat. In Colombia it grows at an altitude of 3,000–3,500 m. Flowering occurs in March and August.

*Distribution*: Colombia, Venezuela.

*Representative specimens*: COLOMBIA. **Cauca**: 3,000–3,500 m, March 1886, *F.C. Lehmann 5885* (K!, RENZ!, UGDA-DLSz!—drawing). **Cundinamarca**: Plants with unopened flowers collected on July 20, 1941, which flowered in Bogotá in mid-August 1941, 3,000 m, *O. Renz 4160* (RENZ!).

*Other materials examined*: VENEZUELA. Mérida, 2,880 m, November 20, 1949, *O. Renz 6156* (RENZ!).

*Notes*: Renz noted that this is possibly only a variation of *C. monophylla*; however, it is easily distinguished from this species by the crenate leaf margins, the suborbicular lip lacking any callosities (vs. lip ovate-oblong with numerous fleshy projections on the inner surface), subdensely-flowered inflorescence (vs. rachis laxly flowered) and floral bracts longer than ovary (vs. floral bracts shorter than ovary). Crenate leaf margins and entire margins of petals distinguish this species from the morphologically similar *C. picta* Rchb.f.

***Cranichis calva*** (Kraenzl.) Schltr., Repert. Spec. Nov. Regni Veg. Beih. 9: 128. 1921.
*Ponthieva calva* Kraenzl., Bot. Jahrb. Syst. 54(117): 20. 1916. TYPE: Peru. *A. Weberbauer 6314* (B†; AMES!—photo).

**Figure 37:**
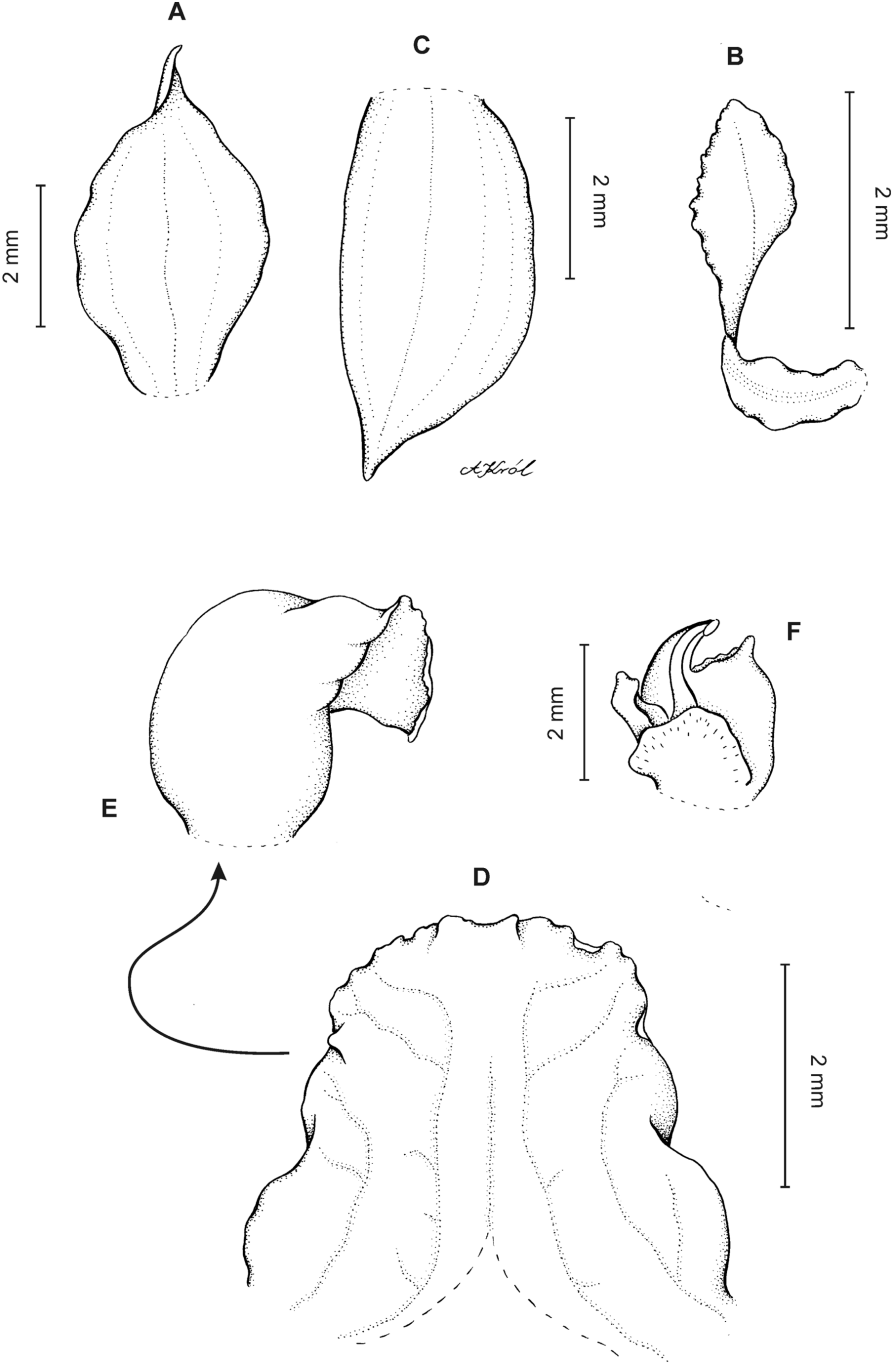
*Cranichis calva* (Kraenzl.) Schltr. (A) Dorsal sepal; (B) petal; (C) lateral sepal; (D) lip (front view); (E) lip (side view); (F) gynostemium. Drawn by A. Król from *Lehmann 475* (W-R).

Plant about 14–27 cm or more tall. Leaves 1 or 2, basal, long-petiolate; petiole up to eight cm or more long; blade up to 18 cm long, 3.2–4.5 cm wide, oblong-elliptic or oblong-lanceolate, short-acuminate, cuneate below. Scape slightly surpassing the leaf, with one to two remote, foliaceous sheaths, glabrous below raceme. Inflorescence about five to nine cm long, cylindric, loosely to subdensely many-flowered. Flowers white, the lip veined with purple or maroon, glabrous. Floral bracts 6–10 mm long, lanceolate, acute or acuminate, glabrous. Pedicellate ovary about seven mm long, slender, glabrous. Dorsal sepal 4.5–5.8 mm long, 2–2.5 mm wide, oblong-elliptic to elliptic, obtuse to acuminate, 3-veined. Petals 3.5–3.7 mm long, one mm wide, cuneate-spatulate, obtuse, margins erose, 1-veined. Lateral sepals 5–5.5 mm long, 2.7–3 mm wide, obliquely ovate, subacute, 3-veined. Lip 3.7–4.5 mm long, 3.5–4.2 mm wide, deeply concave, basally gibbose, sessile, semiovate and concave-conduplicate when viewed from the side, with a slightly recurved, broadly obtuse apex, very broadly ovate if expanded; disc with thickened, branching veins, without nodules. Gynostemium 2–2.8 mm long (Fig. 37).

*Ecology*: Terrestrial plant growing in Colombia at an altitude of 2,800–3,000 m. Flowering occurs in February and May.

*Distribution*: Peru (Schweinfurth, 1958), Colombia.

**Figure 38:**
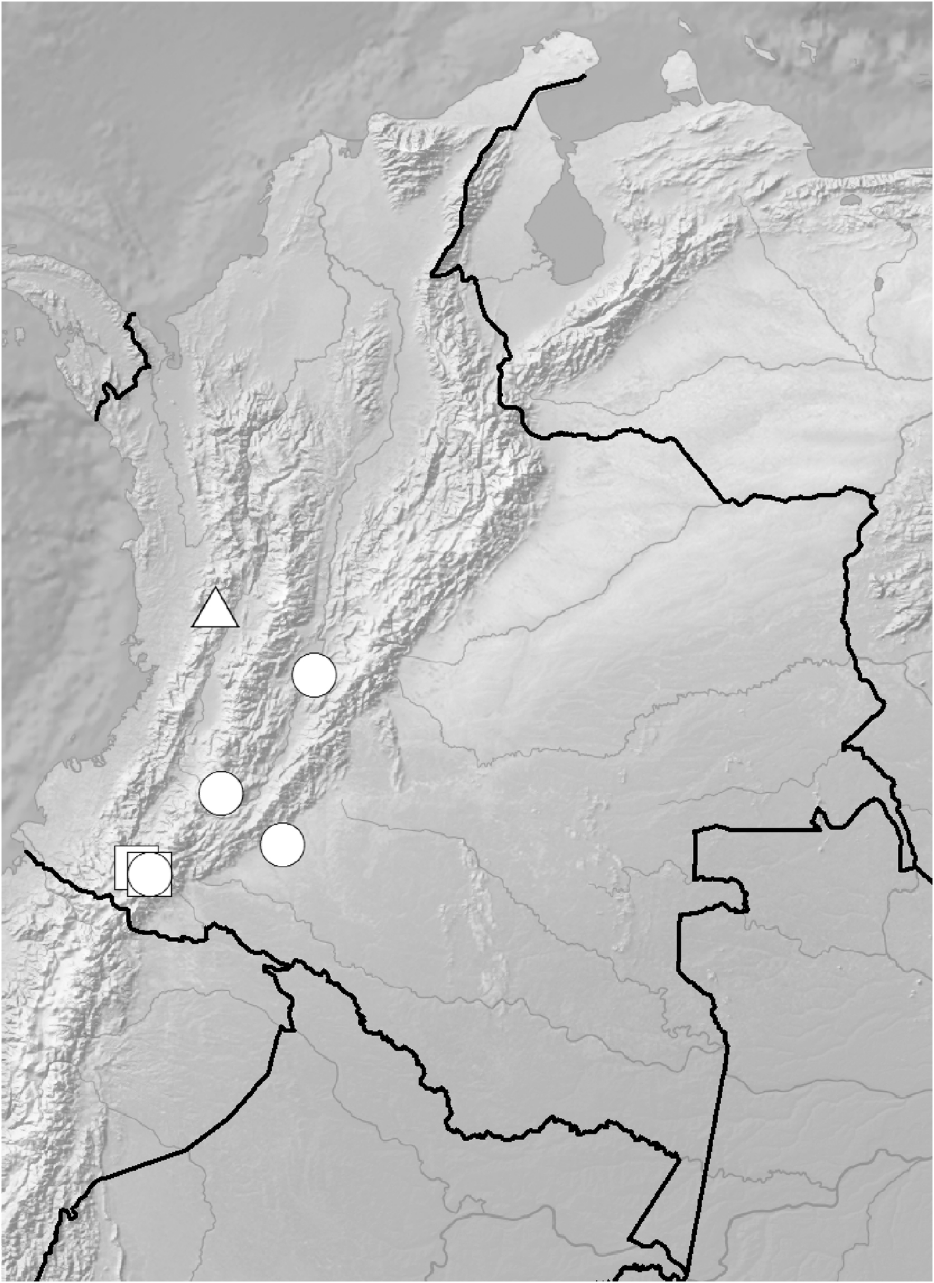
Distribution of members of the *Cranichis Polyantha* group. *C. calva* (square), *C. longipetiolata* (triangle), *C. polyantha* (circle).

*Notes*: This species can be distinguished from both taxa characterized above by having 1-veined petals with erose margins.

*Representative specimens*: COLOMBIA. **Cauca**: Volcán de Pasto, 3,000 m, February 14, 1880, *F.C. Lehmann 476* (W!, UGDA-DLSz!—drawing). **Nariño**: Pasto, corregimiento del Encano, Isla La Corota, 2,800 m, May 6, 1988, *O. de Benavides 9814* (COL, PSO—Dueñas Gómez & Fernández Alonso, 2009). *Sine loc. J. Ordonez et al. 1735* (COL!, UGDA-DLSz!—drawing) (Fig. 38).

This species appears to be closely allied to *C. multiflora* (Poepp. & Endl.) Cogn., and may prove to be conspecific (Schweinfurth, 1958).

***Cranichis longipetiolata*** C. Schweinf., Amer. Orchid Soc. Bull. 21: 268. 1952. TYPE: Peru. *Ferreyra 3120* (holotype, AMES!; UGDA-DLSz!—drawing).

**Figure 39:**
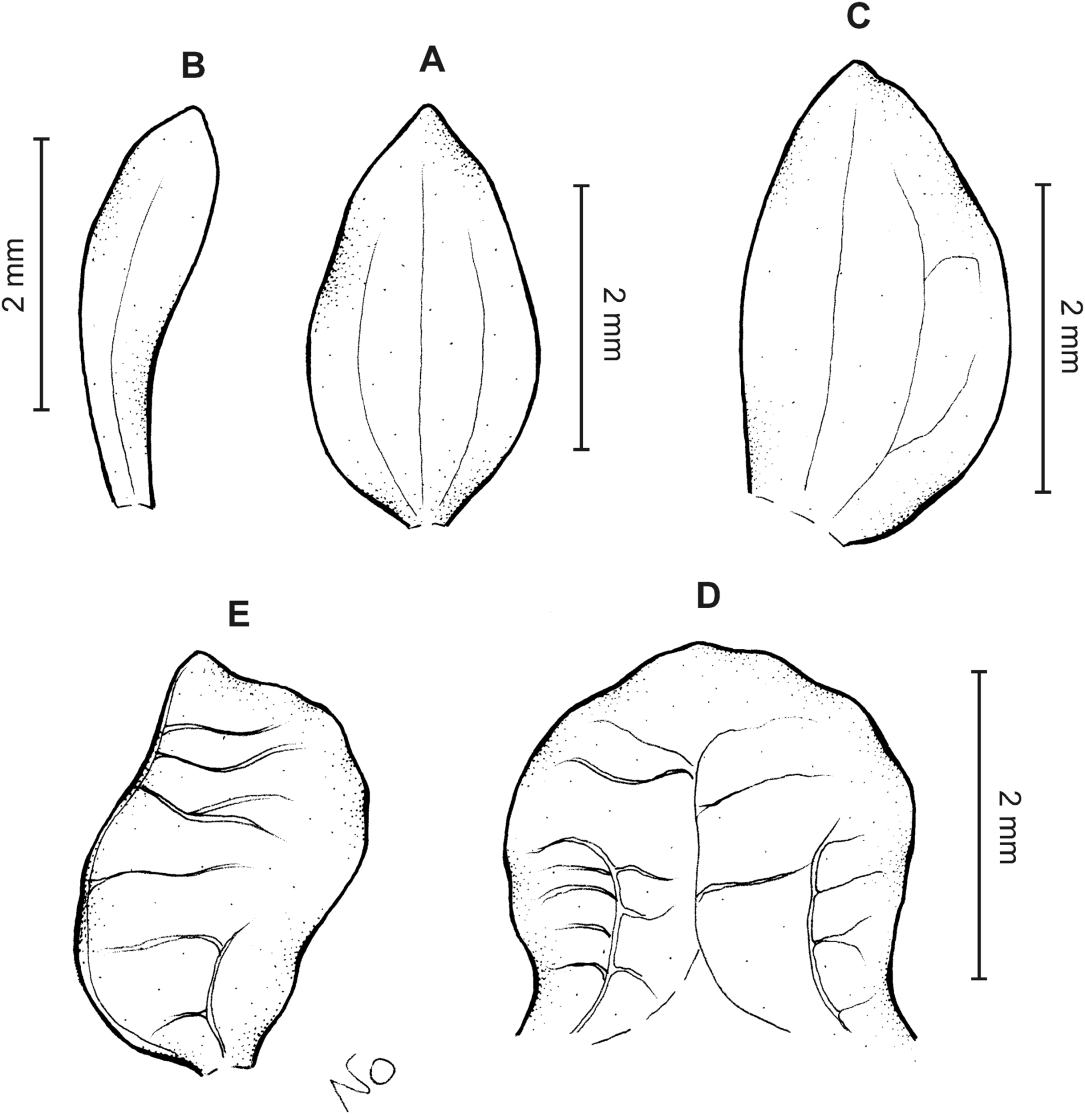
*Cranichis longipetiolata* C. Schweinf. (A) Dorsal sepal; (B) petal; (C) lateral sepal; (D) lip (front view); (E) lip (side view). Drawn by N. Olędrzyńska from *Ferreyra 3120* (AMES) Scale bar for (E–D) = 2 mm.

**Figure 40:**
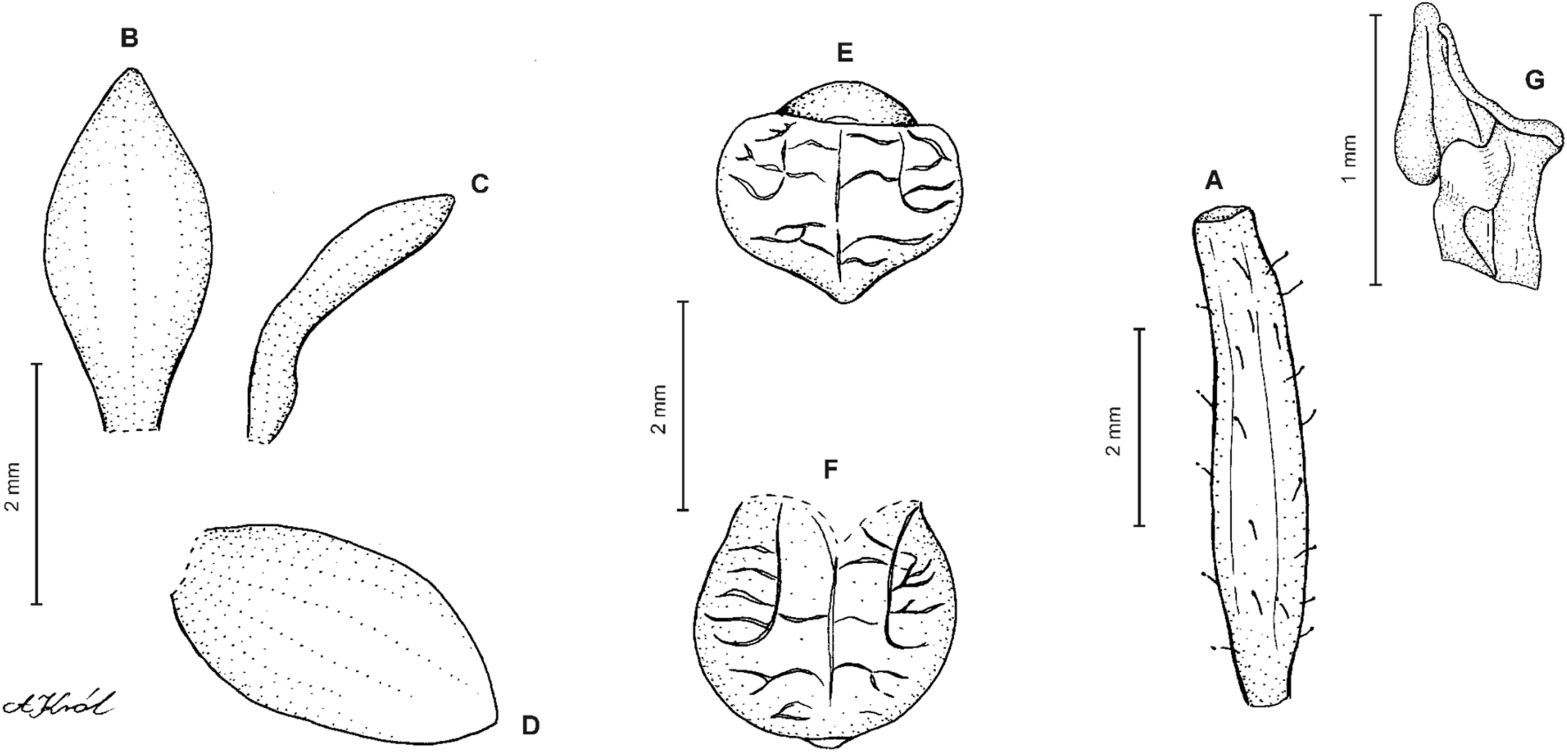
*Cranichis longipetiolata* C. Schweinf. (A) Ovary; (B) dorsal sepal; (C) petal; (D) lateral sepal; (E–F) lip (front view); (G) gynostemium. Redrawn by A. Król from Garay’s illustration of a specimen collected by *Camp E3074* (AMES).

Plants up to 40 cm tall. Leaves 1–3, basal, petiolate; petiole up to 18 cm long; blade 8–16 cm long, up to 4.3 cm wide, elliptic or oblong-elliptic, oblique, acute, cuneate at the base. Scape more or less surpassing the leaves, glabrous below, finely pubescent above, with three to five inconspicuous, remote sheaths. Inflorescence 5.5–9 cm long, conical, densely many-flowered. Flowers very small, white, with spreading segments. Floral bracts seven mm long, lanceolate, glabrous. Pedicellate ovary five to nine mm long, glandular. Dorsal sepal 3.2–4 mm long, 1.5–1.9 mm wide, ovate-elliptic, acute, concave, 3-veined. Petals three mm long, 0.7 mm wide, oblanceolate-linear, obtuse, more or less oblique or curved, 1-veined. Lateral sepals 3.2–4.1 mm long, 2–2.3 mm wide, obliquely ovate, acute or subacute, 2- to 4-veined, the outer vein sometimes branching. Lip 2.6–3 mm long, 2.6–4 mm wide, deeply concave, basally gibbose, shortly unguiculate, suborbicular, apex rounded, base more or less cuneate; disc with three transversely anastomosing veins. Gynostemium two mm long (Figs. 39 and 40).

*Ecology*: Terrestrial plant growing in humid montane forest at an altitude of 2,150 m. Flowering occurs in December.

*Distribution*: Peru, Ecuador, Colombia.

*Representative specimens*: COLOMBIA. **Valle del Cauca**: Mun. El Cairo. Corregimiento El Boquerón, vereda El Brillante, sector La Pradera, reserva de la Sociedad Civil Cerro El Inglés. Bosque húmedo de montaña, 04°44′–04°45′N, 76°16′–76°17′W, 2,150 m, December 28, 2007, *R. Arevalo et al. 787* (COL!, UGDA!—drawing) (Fig. 38).

*Other materials examined*: PERU. Piura. Huancabamba. Canchaque, 1,400–1,600 m, March 22, 1948, *R. Ferreyra 3120* (AMES!; UGDA-DLSz!). ECUADOR. **Carchi**: El Carmelo, E of Tulcán, 2,200 m. June 1985. *A. Hirtz 2651* (RPSC!, UGDA-DLSz!—drawing).

*Notes*: This species can be misidentified with *C. calva*, but unlike the latter it has petals with entire margins.

***Cranichis polyantha*** Schltr., Repert. Spec. Nov. Regni Veg., Beih. 7: 61. 1920. TYPE: Colombia. *Madero 22* (B†, lectotype, designated by Garay (1978: 203) AMES!—drawing).

**Figure 41:**
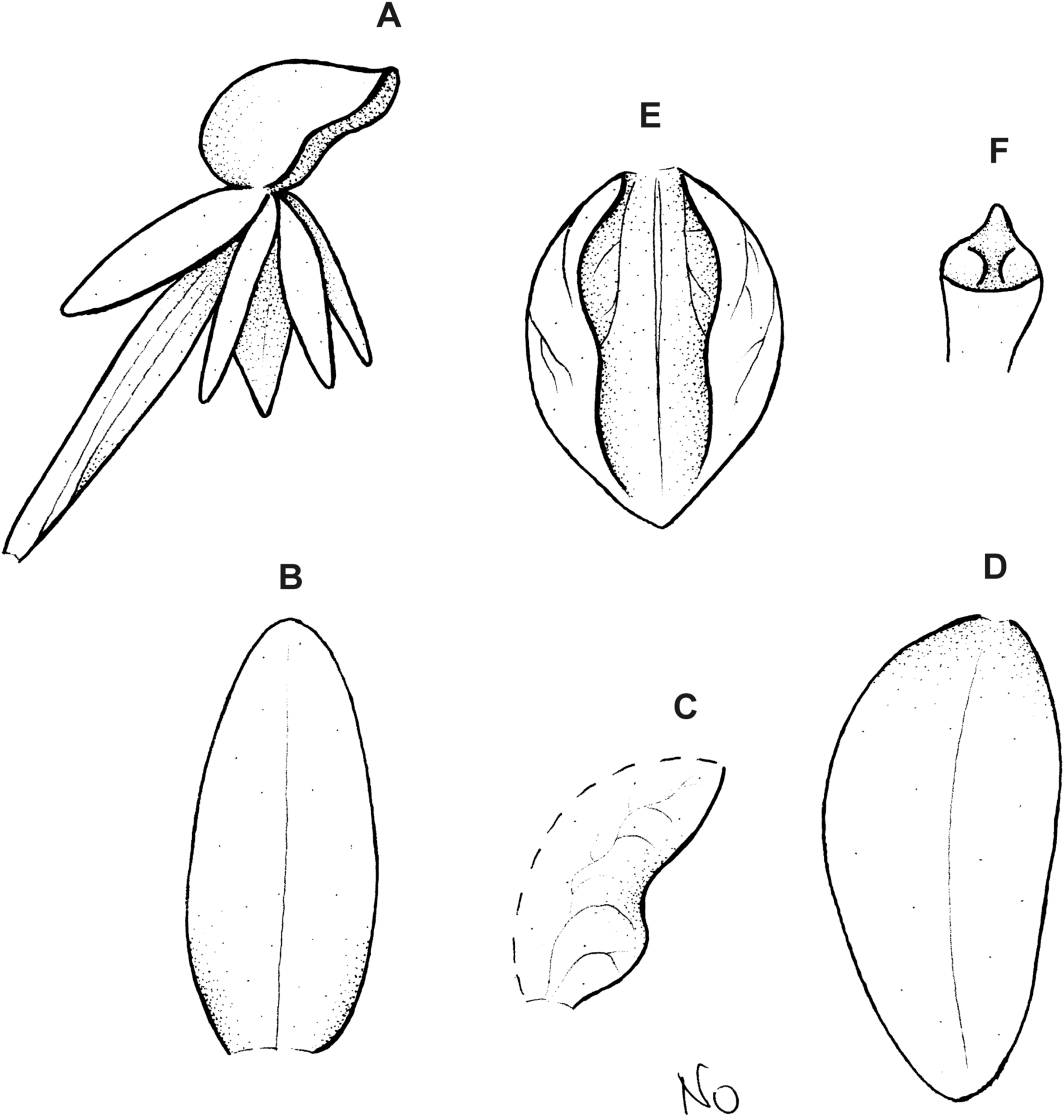
*Cranichis polyantha* Schltr. (A) Flower; (B) dorsal sepal; (C) petal; (D) lateral sepal; (E) lip (front view); (F) gynostemium. Redrawn by N. Olędrzyńska according to the original illustration from Schlechter.

**Figure 42:**
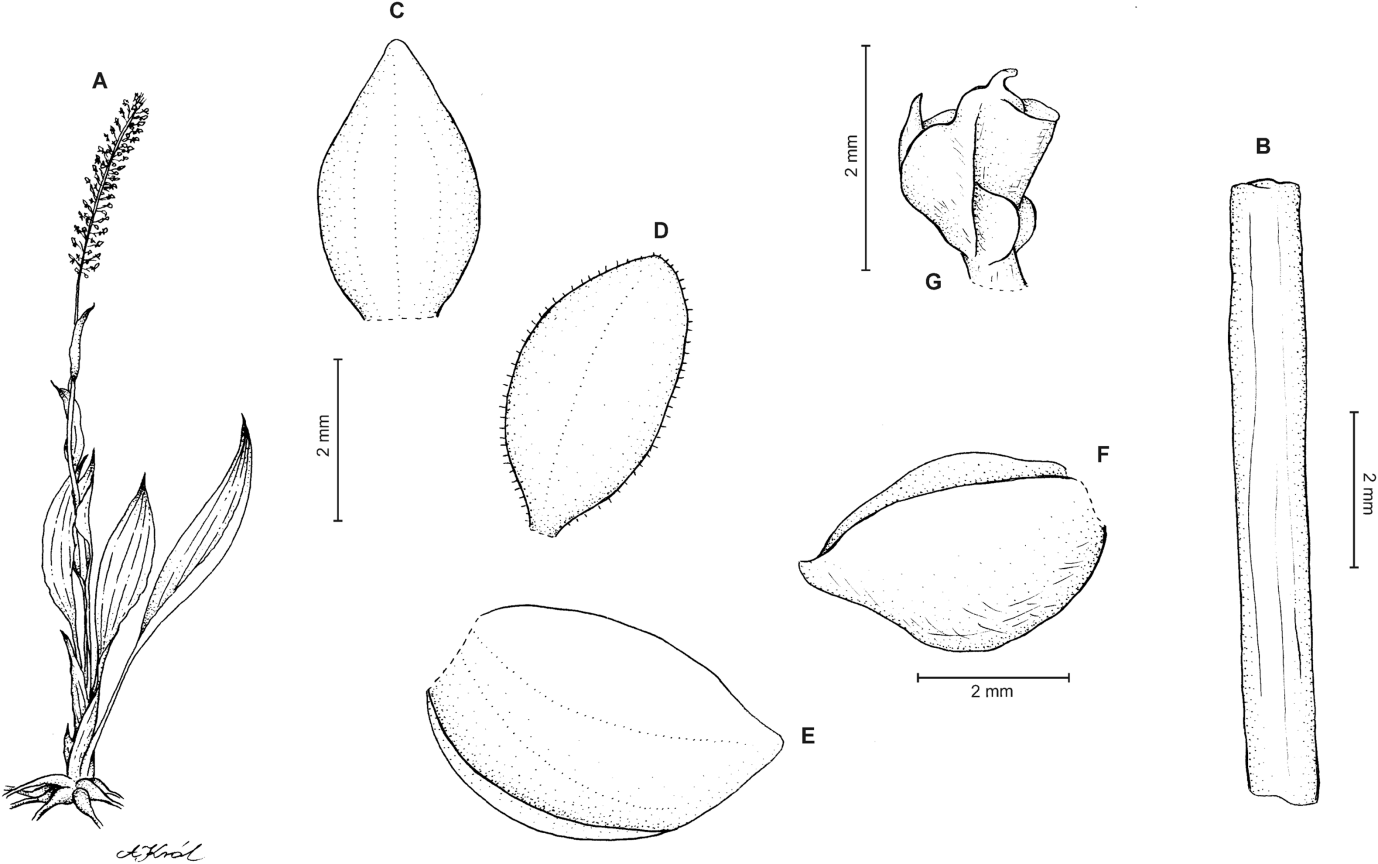
*Cranichis polyantha* Schltr. (A) Habit; (B) ovary; (C) dorsal sepal; (D) petal; (E) lateral sepal; (F) lip; (G) gynostemium. Redrawn by A. Król from Garay’s illustration of a specimen collected by *Lehmann 6540* (K).

Plants 18–40 cm tall, erect. Leaves 1–3, basal, petiolate; petiole 5.5–7.5 cm long, channeled, thick, fleshy; blade 8.5–28 cm long, 2.2–4 cm wide, lanceolate, acute. Scape erect, glabrous, remotely few-sheathed. Inflorescence 6.5–51 cm long, cylindrical, subdensely many-flowered. Flowers small, the dorsal sepal crystalline green, the lateral sepals with superior border pale green, interior border pale greenish white, the petals bright green with white borders, the lip white, inner surface of sack with small dark-green minutely spiculate pustules, the gynostemium crystalline pale green. Floral bracts seven mm long, lanceolate, acuminate, spreading. Pedicellate ovary 6–11 mm long, glandular. Dorsal sepal 3–5.2 mm long, one to two mm wide, elliptic-lanceolate to oblong-elliptic, obtuse, concave, 1- or 3-veined. Petals three to five mm long, 0.9–1.4 mm wide, broadly oblanceolate, elliptic or ligulate from a short claw, abruptly acuminate, obtuse, subfalcate, 1-veined. Lateral sepals 3–5.5 mm long, 1.3–2.7 mm wide, very strongly oblique, elliptic-ovate, obtuse to subacute, 1-, 2-, or 3-veined. Lip two to five mm long, 1.9–5 mm wide, deeply concave, subsessile, gibbose, lateral margins slightly revolute, apex more or less undulate; disc with 3, strongly branching veins. Gynostemium 1.5–3.2 mm long (Figs. 41 and 42).

*Ecology*: Terrestrial in wet montane forest and cloud forest at altitudes of 2,000–3,200 m. Flowering throughout the year.

*Distribution*: Peru, Ecuador, Colombia.

*Representative specimens*: COLOMBIA. **Caqueta**: Entre San Vicente y Campo Alegre, 2,000 m, March 1939, Plants with unopened flowers collected at the beginning of March 1939, which flowered in Bogotá at the beginning of June 1939, *O. Renz 4126* (AMES—drawing K, RENZ!). **Cauca**: *Sine loc*., 2,000 m. *M. Madero s.n*. (B†); *Sine loc., M. Madero 22* (B†, AMES!—drawing); Santa Leticia, 2,100–2,300 m, September 6, 1961, *A. Fernandez P. 5882* (COL!, UGDA-DLSz!—drawing). **Nariño**: Mpio. de Pasto. Corregimineto del Encano. Isla la Corota, 2,800 m, May 6, 1988, *O. de Benavides 9814* (COL!). **Tolima**: Mpio. del Libano. Murillo, 3,100–3,200 m, January 8, 1952, *M. Schneider 615* (COL!—sterile) (Fig. 38).

*Other materials examined*: PERU. **Amazonas**: Bongara. Quebrada Chido, 2,700 m, April 1, 1944, *W. Hodge 6137* (AMES!, UGDA-DLSz!—drawing). ECUADOR. **Imbabura**: Selva Alegre, 2,650 m, December 6, 1984, *A. Hirtz 2156* (RPSC!). **Loja**: Road from Loja to Zamora, km 13, 2,800 m, December 5, 1957, *C. Dodson 221* (RPSC!, UGDA-DLSz!—drawing). **Zamora-Chinchipe**: E side of pass on road from Yangana to Valladolid, March 24, 1985, *A. Hirtz et al. 2343* (RPSC!). *Sine loc*,. April 6, 1985, *A. Hirtz 2421* (RPSC!).

*Notes*: This species appears to be similar to *C. calva*, from which it can be distinguished by having glabrous ovary (vs. glandular) and petals with erose margins (vs. entire margins).

It is similar to *C. ciliata* Kunth., but easily distinguishable from the latter by leaf shape, much longer inflorescence, and shape of petals. Garay (1978) cited as the lectotype of this species *Madero 22*, which is not mentioned in the protologue by Schlechter (1920).

In 1948, based on plants collected at the Colombian department of Caqueta, Renz described a new variety within *C. polyantha*, however, it seems that var. *caquetaensis* Renz falls within the morphological variation of *C. polyantha* and there is no reason to recognize this taxon.

### *Polyblephara* group

Lip deeply cochleate, gibbose at base, suborbicular to elliptic when spread, veins either thickened or not, dendritic branching, without any nodules. Petals variously ciliate along both margins.

Three Colombian species belong to this group.

## Key to the Species

1. Petal margins ciliolate2
1* Petal margins long-ciliate or pubescent*C. popayanensis*
2. Petals sparsely ciliate along margins, ovary sparsely glandular*C. brachyblephara*
2* Petals densely ciliate, ovary glabrous*C. polyblephara*

***Cranichis brachyblephara*** Schltr., Repert. Spec. Nov. Regni Veg., Beih. **7**: 58. 1920. TYPE: Colombia. *Madero s.n*. (B†).

**Figure 43:**
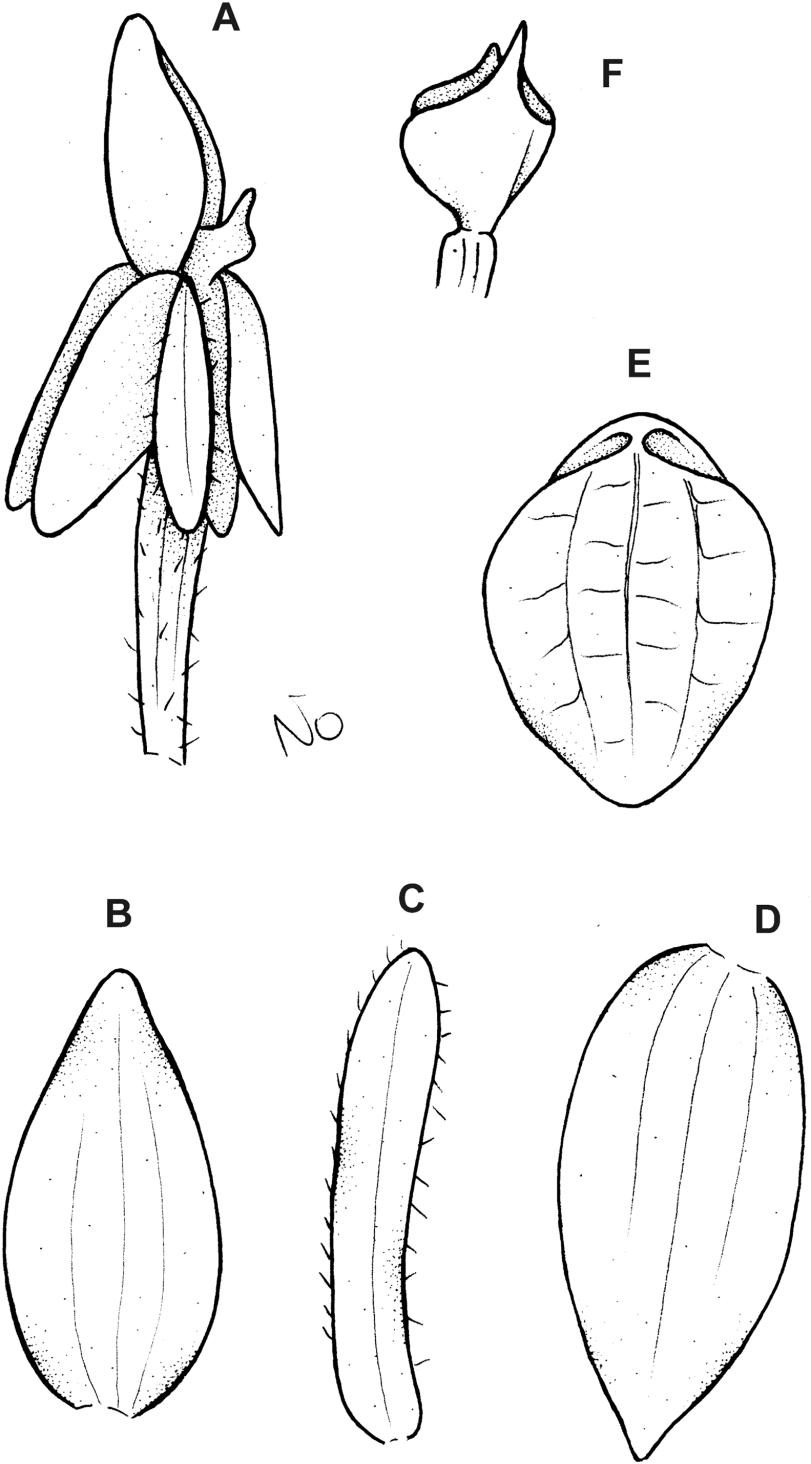
*Cranichis brachyblephara* Schltr. (A) Flower; (B) dorsal sepal; (C) petal; (D) lateral sepal; (E) lip (front view); (F) gynostemium. Redrawn by N. Olędrzyńska according to the original illustration from Schlechter.

Plants 40 cm tall. Leaf 1, basal, petiolate; petiole six to seven cm long, narrow, canaliculate; blade not seen. Scape densely glandular toward the apex. Inflorescence six to seven cm long, densely many-flowered. Flowers small, sepals glabrous. Floral bracts subequal in length to the ovary, lanceolate, acuminate. Pedicellate ovary seven mm long, sparsely glandular. Dorsal sepal 3.5 mm long, 1.4 mm wide, ovate-lanceolate to broadly ovate, subobtuse, 3-veined. Petals 3.5 mm long, 0.7 mm wide, linear to ligulate-oblong, obtuse, subfalcate, sparsely ciliolate on margins, 1-veined. Lateral sepals 3.5 mm long, 1.6 mm wide, obliquely ligulate-ovate to elliptic-ovate, slightly concave at the base, subacuminate, subobtuse, obscurely 3-veined. Lip 4.5 mm long, 3.75 mm wide, concave, basally shallowly saccate, sessile, broadly ovate, obtuse; disc with three branching veins, main veins thickened, branches and anastomoses thin, without any nodules. Gynostemium 1.5 mm long (Fig. 43).

*Ecology*: Terrestrial plant growing at an altitude of about 3,000 m. No data on flowering time.

*Distribution*: Colombia.

*Representative specimens*: COLOMBIA. **Cauca**: *Sine loc*., 3,000 m, *M. Madero s.n*. (B†).

*Notes*: It can be confused with *C. polyblephara* but has sparsely ciliate petal margins and ovary sparsely glandular.

According to Schlechter (1920) floral bracts are longer, flowers larger, petals narrower and shorter, sparsely hairy, and lip more saccate than in its close ally, *C. antioquiensis*.

***Cranichis polyblephara*** Schltr., Repert. Spec. Nov. Regni Veg., Beih. **7**: 61. 1920. TYPE: Colombia. *Lehmann 2812* (B†, BM).

**Figure 44:**
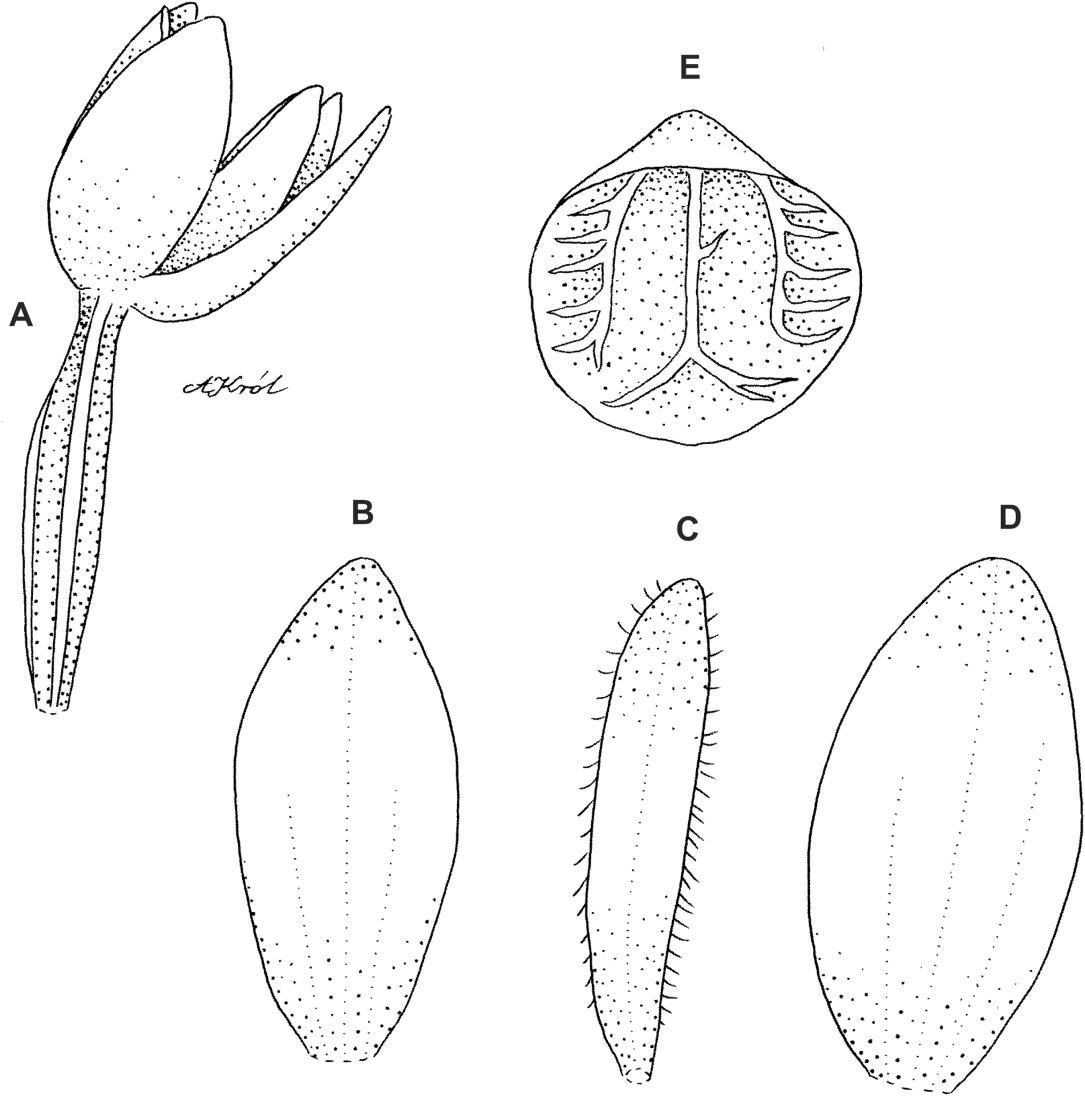
*Cranichis polyblephara* Schltr. (A) Flower; (B) dorsal sepal; (C) petal; (D) lateral sepal; (E) lip (front view). Redrawn by A. Król according to the original illustration from Schlechter.

Plants 12–25 cm tall. Leaf 1, basal, petiolate; petiole 2.5–3 cm long; blade up to 3.5 cm long, 2.8 cm wide; ovate, acute. Scape glandular toward the apex, enclosed in three to four sheaths. Inflorescence two to six cm long, conical to cylindrical, densely few- to many-flowered. Flowers small, glabrous. Floral bracts up to ca. seven mm long, ovate-lanceolate, acuminate, glabrous. Pedicellate ovary 5.5 mm long, glabrous. Dorsal sepal three mm long, 1.2 mm wide, elliptic-obovate to oblong-obovate, obtuse, obscurely 3-veined. Petals three mm long, 0.6 mm wide, linear-oblanceolate to ligulate-oblanceolate, obtuse, subfalcate, shortly and densely ciliolate on both margins, 1-veined. Lateral sepals three mm long, 1.4 mm wide, elliptic to oblong elliptic-ovate, somewhat oblique, slightly concave at the base, obtuse, obscurely 3-veined. Lip 2.75 mm long and wide, concave, gibbose to shallowly saccate at base, sessile, suborbicular, cucullate, obtuse; disc with three thickened, dendritic branching veins without any nodules. Gynostemium ca. one mm long (Fig. 44).

*Ecology*: Terrestrial plant growing at an altitude of 1,600–1,900 m. Flowering occurs in May.

*Distribution*: Colombia.

**Figure 45:**
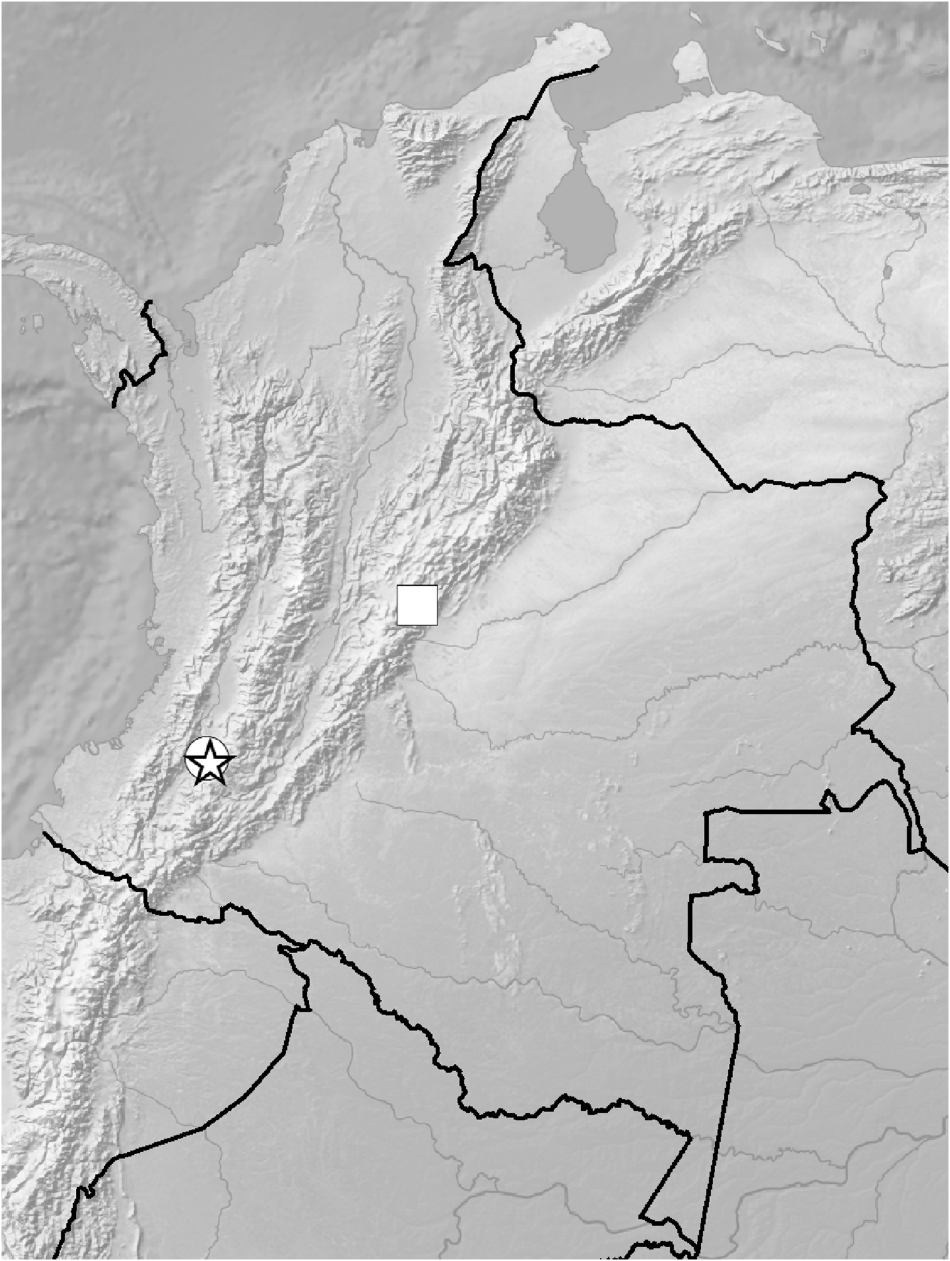
Distribution of members of the *Cranichis Polyblephara* group and *Cristalensis* group. *C. polyblephara* (circle), *C. popayánensis* (star), and *C. cristalinensis* (square).

*Notes*: This species is morphologically similar to *C. brachyblephara* but petal margins are densely ciliate and ovary is glabrous.

*Representative specimen*: COLOMBIA. **Cauca**: Bei Popayán, 1,600–1,900 m, May 1883, *F.C. Lehmann 2812* (B†, BM) (Fig. 45).

Describing this species Schlechter (1920) compared it with *C. ciliata* and stated that it differs from the former by smaller leaf, smaller flowers, shorter ovary, and form of petals.

***Cranichis popayanensis*** Szlach. & Kolan., ***sp. nov***. TYPE: Colombia. *de Escobar et al. 4257* (holotype, MO!, UGDA-DLSz!—drawing).
*Species distinguished from C. polyblephara by the anterior margin of petals being pubescent and posterior margin sparsely ciliate*.

**Figure 46:**
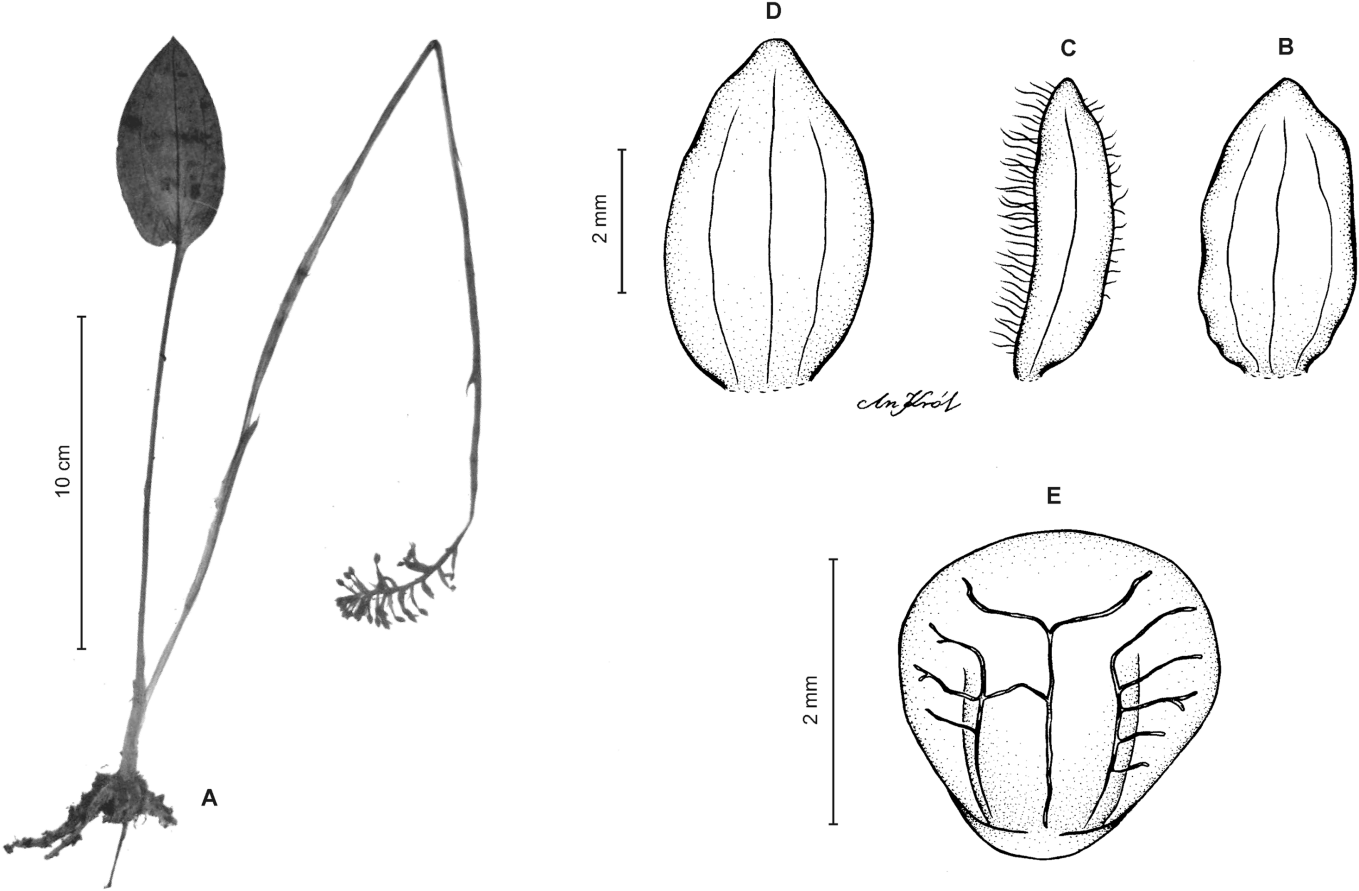
*Cranichis popayánensis* Szlach. & Kolan. (A) Habit; (B) dorsal sepal; (C) petal; (D) lateral sepal; (E) lip (front view). Scale bar for (B–D) = 2 mm. Drawn by A. Król from *de Escobar et al. 4257* (MO).

Plants about 43 cm tall. Leaf 1, basal, erect-patent, petiolate; petiole 15 cm long, narrow, canaliculate; blade six cm, three cm wide, ovate, shortly acuminate, base rounded. Scape delicate, terete, apically densely glandular, enclosed in seven sheaths. Inflorescence about four cm long, laxly many-flowered. Flowers small. Floral bracts six mm long, lanceolate, acuminate, glabrous. Pedicellate ovary eight mm long, cylindrical, glabrous. Dorsal sepal 3.3 mm long, 1.5 mm wide, elliptic-oblong, obtuse, glabrous, 3-veined. Petals three mm long, 0.9 mm wide, obliquely oblong-ligulate, obtuse, anterior margin pubescent, posterior margin sparsely ciliate, 1-veined. Lateral sepals four mm long, two mm wide, obliquely elliptic-ovate, obtuse, glabrous, 3-veined. Lip 2.5 mm long, 2.8 mm wide, basally concave, gibbose, subsessile, transversely elliptic-orbicular, truncate at the apex; disc with three branching veins, without any nodules. Gynostemium 2.5 mm long (Fig. 46).

*Etymology*: In reference to the place of collection of the type specimen.

*Ecology*: Terrestrial plant growing at an altitude of 2,300–2,500 m. Flowering occurs in May.

*Distribution*: Colombia.

*Representative specimens*: COLOMBIA. **Cauca**: Camino Real entre Popayán y Coconuco cerca al Alto de Pesares, 2,300–2,500 m, May 3, 1984, *L. de Escobar et al. 4257* (MO!, UGDA-DLSz!—drawing) (Fig. 45).

*Notes*: This species can be easily distinguished from other species classified in this group by having pubescent anterior margin of petals and sparsely ciliate posterior one.

### *Cristalinensis* group

Lip subglobose, very thick in the center, margins thinner, erose-dentate. Petals with glabrous margins.

***Cranichis cristalinensis*** Szlach. & Kolan., Polish Bot. J. 58(2): 618. 2013. TYPE: Colombia. *Mendoza et al. 15473* (holotype, COL!; isotype, FMB!, UGDA-DLSz!—drawing).

**Figure 47:**
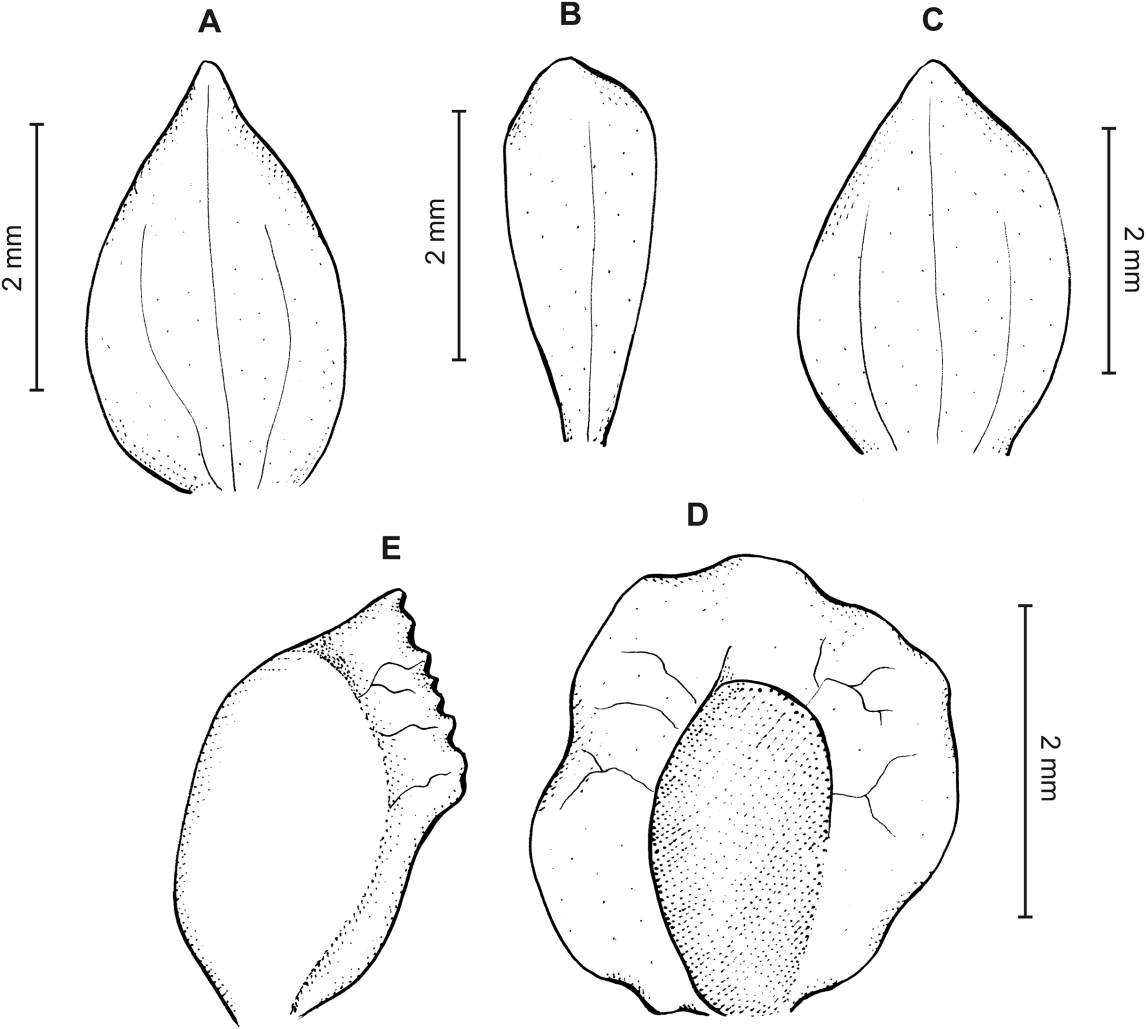
*Cranichis cristalinensis* Szlach. & Kolan. (A) Dorsal sepal; (B) petal; (C) lateral sepal; (D) lip (front view); (E) lip (side view). Drawn by S. Nowak from *Mendoza et al. 15473* (COL).

Plants up to 40 cm tall. Leaves 2, basal, petiolate; petiole up to 6.5 cm long, narrow; blade up to 5.3 cm long and 2.2 cm wide, oblong- or ovate-lanceolate, acute, rounded or truncate at base. Scape erect, delicate, glandular in the upper third, enclosed in four sheaths. Inflorescence up to 5.5 cm long, subdensely many-flowered. Flowers small, inconspicuous, glabrous. Floral bracts 4.5 mm long, lanceolate, acuminate, glabrous or sparsely glandular. Pedicellate ovary up to 7.5 mm long, papillate. Sepals glabrous. Dorsal sepal 3.5 mm long, 2.1 mm wide, ovate, subobtuse, somewhat cochleate in the center, 3-veined. Petals 3.1 mm long, 1.3 mm wide, oblong-spathulate, rounded at apex, subfalcate, glabrous along margins, 1-veined. Lateral sepals 3.2 mm long, 2.1 mm wide, ovate to elliptic-ovate, acute to shortly acuminate, somewhat oblique, slightly concave, 3-veined. Lip concave, sessile, three mm long and wide, suborbicular in outline, apical margins somewhat wavy; disc greatly thickened in the center. Gynostemium 1.8 mm long (Fig. 47).

*Ecology*: Terrestrial plant growing in high-montane forest with *Drimys granadensis* (Winteraceae) at an altitude of 3,200 m. Flowering occurs in November.

*Distribution*: Colombia.

*Representative specimen*: COLOMBIA. **Cundinamarca**: Fomeca. Vda. La Cristalina. Parque Nacional Natural Chingaza. Laguna de Chingaza. Sector La Playa. Bosque de *Drymis granatensis*, planta terrestre, flores blancas. 4°31′50″, 73°45′32″, 3,200 m, November 17, 2003, *H. Mendoza et al. 15473* (COL!, FMB!) (Fig. 45).

*Notes*: *Cranichis cristalinensis* appears to be somewhat related to *C. sylvatica* and *C. werffii*, but is easily distinguishable from both species mentioned by the petals and lip shape. Petals of this entity are widest at the apex, lip is sessile, suborbicular, much thickened in the center.

### *Diphylla* group

Lip suborbicular to elliptic when spread, thin, rarely somewhat thickened in the lower half, veins with small globose projections, dendritic branching. Petals glabrous along both margins to ciliate.

A total of 13 Colombian species belong to this group.

## Key to the Species

1. Lip basally truncate, subcordate, or auriculated2
1* Lip cuneate or truncate at the base, but never auriculate3
2. Ovary glandular, lateral sepals 2-veined*C. diphylla*
2* Ovary usual glabrous, lateral sepals 1-veined*C. fendleri*
3. Petals ciliate along margins4
3* Petals glabrous along margins5
4. Lip elliptic-suborbicular, almost flat with recurved acute apex*C. queremalensis*
4* Lip ovate to suborbicular-ovate, concave, shortly apiculate to acute*C. muscosa*
5. Ovary glandular6
5* Ovary glabrous or almost glabrous10
6. Dorsal and lateral sepals 3-veined*C. schlimii*
6* Dorsal sepal 1-veined, lateral sepals 1-veined, or obscurely 2-veined7
7. Lateral sepals 2-veined*C. stictophylla*
7* Lateral sepals 1-veined8
8. Sepals papillate on the outer surface*C. ovatilabia*
8* Sepals with smooth outer surface9
9. Lip ovate, widest at the base, subacute to subobtuse*C. nigrescens*
9* Lip elliptic, widest near the middle or above, rounded*C. monophylla*
10. Lateral sepals 3-veined*C. parvula*
10* Lateral sepals 2-veined11
11. Dorsal sepal 3-veined*C. pseudomuscosa*
11* Dorsal sepal 1-veined12
12. Inflorescence 2.5–10.5 cm long, conical*C. lehmannii*
12* Inflorescence 11–15 cm long, cylindrical*C. cylindrostachys*

***Cranichis cylindrostachys*** Schltr., Repert. Spec. Nov. Regni Veg., Beih. 7: 59. 1920. TYPE: Colombia. *Madero s.n*. (B†).

**Figure 48:**
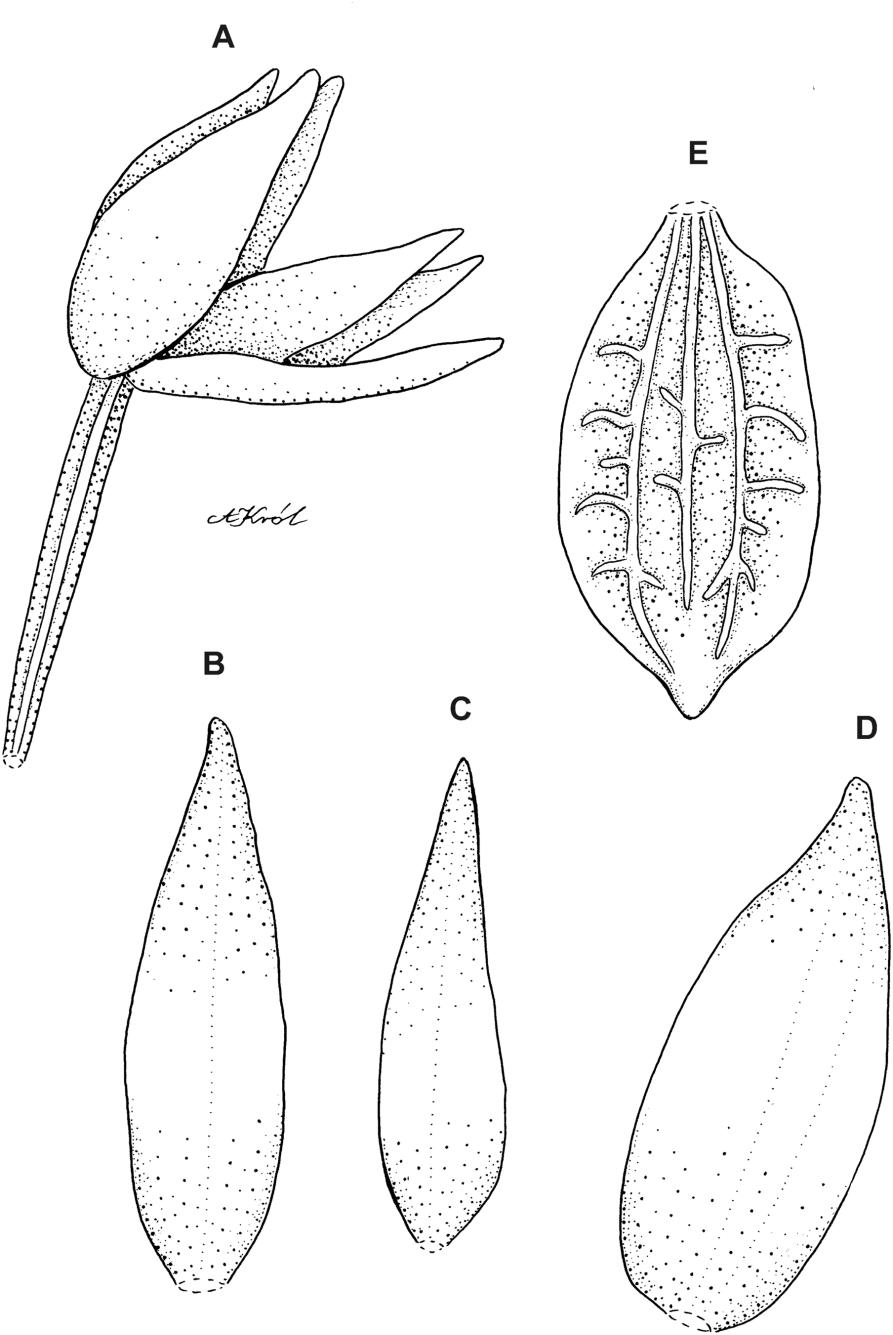
*Cranichis cylindrostachys* Schltr. (A) Flower; (B) dorsal sepal; (C) petal; (D) lateral sepal; (E) lip (front view). Redrawn by A. Król according to the original illustration from Schlechter.

Plants 38–45 cm tall. Leaves 3–4, basal, petiolate; petiole four to nine cm long, narrow, canaliculate; blade six to nine cm long, 2.7–5.5 cm wide, ovate, subacuminate, base obliquely cordate to cuneate. Scape glabrous, remotely few-sheathed. Inflorescence 11–15 cm long, cylindrical, subdensely many-flowered. Flowers small, glabrous. Floral bracts seven mm long, elliptic-lanceolate, acuminate, glabrous. Pedicellate ovary seven to nine mm long, glabrous. Dorsal sepal 3.2–4 mm long, one mm wide, lanceolate, subobtuse to subacute, 1-veined. Petals 3.2–4 mm long, 0.5–0.8 mm wide, obliquely lanceolate to oblong-lanceolate, subacute or subobtuse, glabrous on margins, 1-veined. Lateral sepals four mm long, 1.3–1.5 mm wide, elliptic to elliptic-ovate, somewhat oblique, slightly concave at the base, subacuminate, subobtuse, obscurely 2-veined. Lip 3–3.5 mm long, two mm wide, somewhat concave, subsessile, elliptic to oblong-elliptic, shortly acuminate to blunt; disc with 3 thickened, dendritic branching veins with prominent nodules. Gynostemium 1.5 mm long (Fig. 48).

*Ecology*: Terrestrial plants growing at an altitude of about 1,500 m.

*Distribution*: Colombia, Ecuador.

*Representative specimen*: COLOMBIA. **Cauca**: *Sine loc*., 1,500 m, *M. Madero s.n*. (B†).

*Other materials examined*: ECUADOR. **El Oro**: 18.5 km W of Piñas, 620 m. July 16, 1971, *B. MacBryde 579* (AMES!, UGDA-DLSz!—drawing).

*Notes*: It differs from *C. lehmannii* by the length and shape of inflorescence, therefore we suppose they can be conspecific.

Garay (1978) designated as lectotype of *C. cylindrostachys* drawing made under Schlechter’s supervision of *Madero 14* collection from Antioquia, 1,500 m a.s.l. Schlechter (1920), however, mentioned in the protologue *Madero s.n*.

***Cranichis diphylla*** Sw., Nov. Gen. Sp. Prodr.: 120. 1788. TYPE (Garay, 1978: 192): Jamaica. *Swartz s.n*. (lectotype, BM!; isolectotype, W!; AMES!—drawing).

**Figure 49:**
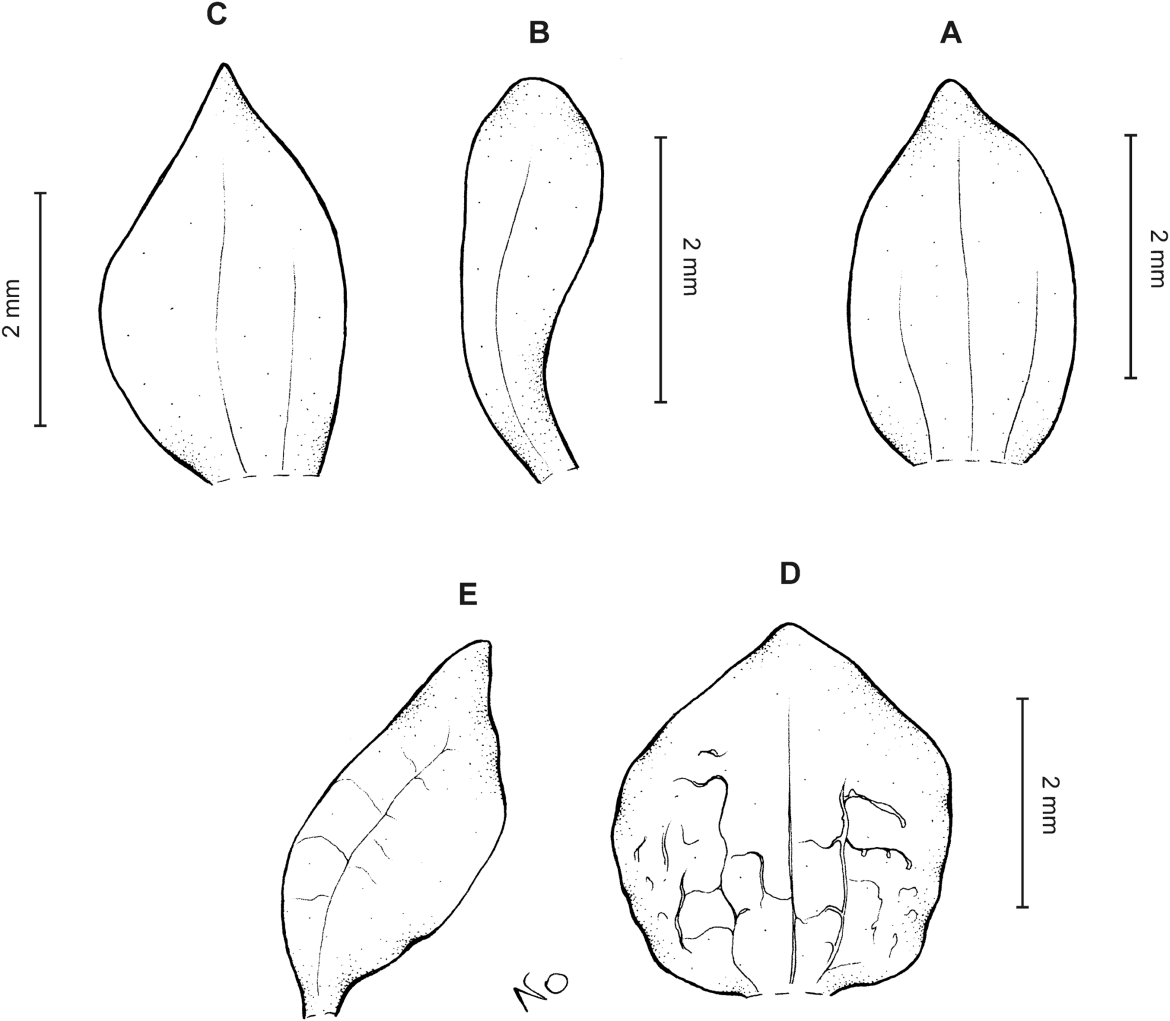
*Cranichis diphylla* Sw. (A) Dorsal sepal; (B) petal; (C) lateral sepal; (D) lip (front view); (E) lip (side view). Drawn by N. Olędrzyńska from *Killip & Smith 15946* (AMES).

Plants up to 40 cm tall. Leaves 1–3, basal, often variegated, petiolate; petiole rather variable in size, up to three cm long; blade up to nine cm long and four cm wide, ovate to ovate-lanceolate, acute to subacuminate, subcordate at the base. Scape slender, erect, remotely few-sheathed, glabrous below, glandular-pubescent above. Inflorescence up to 6.5 cm long, cylindric, loosely to subdensely many-flowered. Flowers white with green veins. Floral bracts four mm long, ovate-lanceolate, acuminate, sparsely glandular. Pedicellate ovary up to six mm long, cylindrical, more or less glandular. Dorsal sepal up to 3.5 mm long and 1.6 mm wide, erect, elliptic, subacute to subobtuse, 3-veined, occasionally sparsely pubescent dorsally. Petals up to 3.1 mm long and one mm wide, near apex linear-oblanceolate, acute to obtuse, 1-veined, glabrous along margins. Lateral sepals up to four mm long and 1.6 mm wide, spreading, obliquely ovate to ovate-elliptic, acute to obtuse, 2-veined, occasionally sparsely pubescent dorsally. Lip up to 3.6 mm long and 3.2 mm wide, concave, subcordate at base, ovate to broadly elliptic in outline, subacute to subobtuse; disc obcordately papillose-thickened with three branching, often glandular veins from base to middle of lip. Gynostemium 1.3–2 mm long (Fig. 49).

*Ecology*: Terrestrial in premontane and montane forest edge at the altitude of 1,300–3,000 m. Flowering occurs in August, November, and December.

*Distribution*: Mexico (Williams, 1951), Nicaragua (Hamer, 1985), Costa Rica, The Greater Antilles (Jamaica, Hispaniola; Cogniaux, 1911), Peru (Schweinfurth, 1970), Ecuador (Dodson & Luer, 2005), Colombia, Venezuela, Suriname, French Guiana.

**Figure 50:**
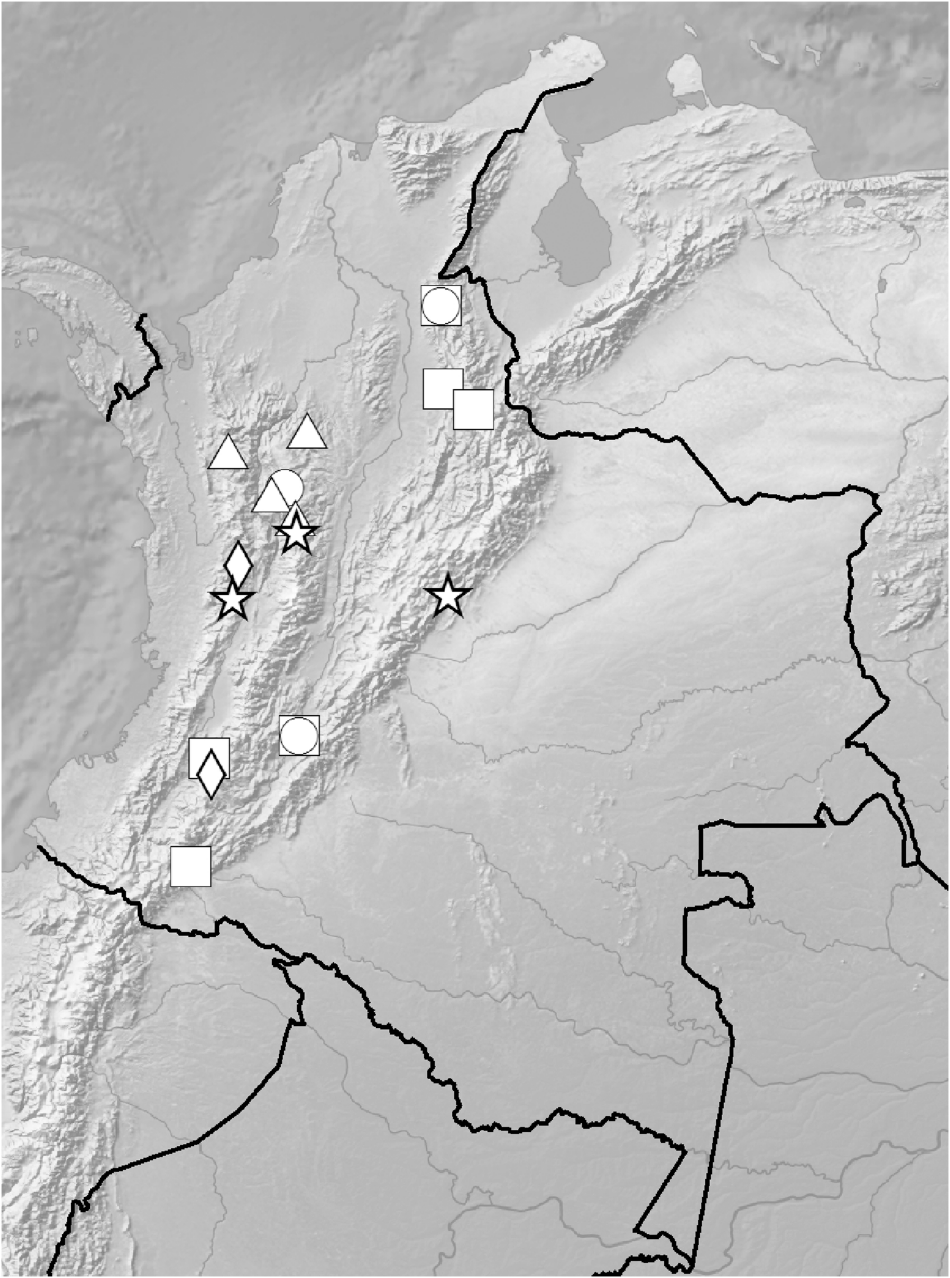
Distribution of members of the *Cranichis Diphylla* group. *C. diphylla* (circle), *C. fendleri* (square), *C. lehmannii* (triangle), *C. monophylla* (diamond), *C. muscosa* (star).

*Representative specimens*: COLOMBIA. **Antioquia**: Mpios. Medellín/Guarne. Parque Ecologico Piedras Blancas, sector Lajas, 6°18′N 75°29′W, 2,350 m, November 19, 1994, *R. Fonnegra et al. 5266* (MO!). **Huila**: Cordillera Oriental. Forest E of Neiva, 1,300–1,800 m, August 1–8, 1917, *H. Rusby & F. Pennell 944* (AMES!). **Santander**: Vicinity of Las Vegas, 2,600–3,000 m, December 21–23, 1926, *E. Killip & A. Smith 15946* (AMES!, UGDA-DLSz!—drawing); The same loc., *E. Killip & A. Smith 16060* (AMES!) (Fig. 50).

*Other materials examined*: FRENCH GUIANA. Inselberg au NW des Monts de la Trinite, August 4, 1981, *K. Cremers 7408* (CAY!, P!) SURINAME. Sipaliwani. Lisa Kreek Savanah. June 26, 1998, *J. Hawkins 1812* (MO!). VENEZUELA. Cerca de la cumbre de El Ávila, 1,900–2,000 m, December 1938, *L. Williams & A. Alston 268* (BM!). COSTA RICA. Bei San Isidro, 1,500 m, December 29, 1881, *F.C. Lehmann 1772* (BM!). JAMAICA. Morse’s path, 1,600 m, December 19, 1892, *H. Harris 7555* (BM!).

*Notes*: This species has very wide geographical range extending from Mexico through the Greater Antilles, French Guiana, to Peru. It is distinguishable from *C. fendleri* by having glandular ovary and 2-veined lateral sepals.

Garay (1978) synonymized under this name the following species: *C. monophylla, C. guatemalensis*, *C. nigrescens*, *C. pittieri, C. ovatilabia*, *C. stictophylla*, and *C. alfredi*. In *C. monophylla*, however, the leaf base is rounded, scape is densely glandular, sepals are 1-veined, and lip disc is glabrous. The scape of *C. nigrescens* is densely glandular-hispid in the upper part, pedicellate ovary is glandular-hispid, sepals are 1-veined, and lip is glabrous inside. *C. ovatilabia* can be distinguished from *C. diphylla* by having leaves with rounded base, scape glandular toward the apex, 1-veined sepals papillate on the outside and lip with broadly cuneate base with glabrous disc. *C. stictophylla* can be recognized by relatively long-petiolate leaves (up to 3 cm vs. 5–9.5 cm), leaf base being subrounded-cuneate (vs. subcordate), 1-veined dorsal sepal (vs. 3-veined) and lip disc with five dendritic veins with prominent nodules (vs. three veins, often glandular). We did not examine any material of *C. guatemalensis*, *C. pittieri*, and *C. alfredii* and we do not want to speculate on their current taxonomic status.

***Cranichis fendleri*** Schltr., Repert. Spec. Nov. Regni Veg., Beih. 6: 30. 1919. TYPE (here designated): Venezuela. *Fendler 2135* (B†; lectotype: AMES!; isolectotypes: G; AMES!—drawing, photo).

**Figure 51:**
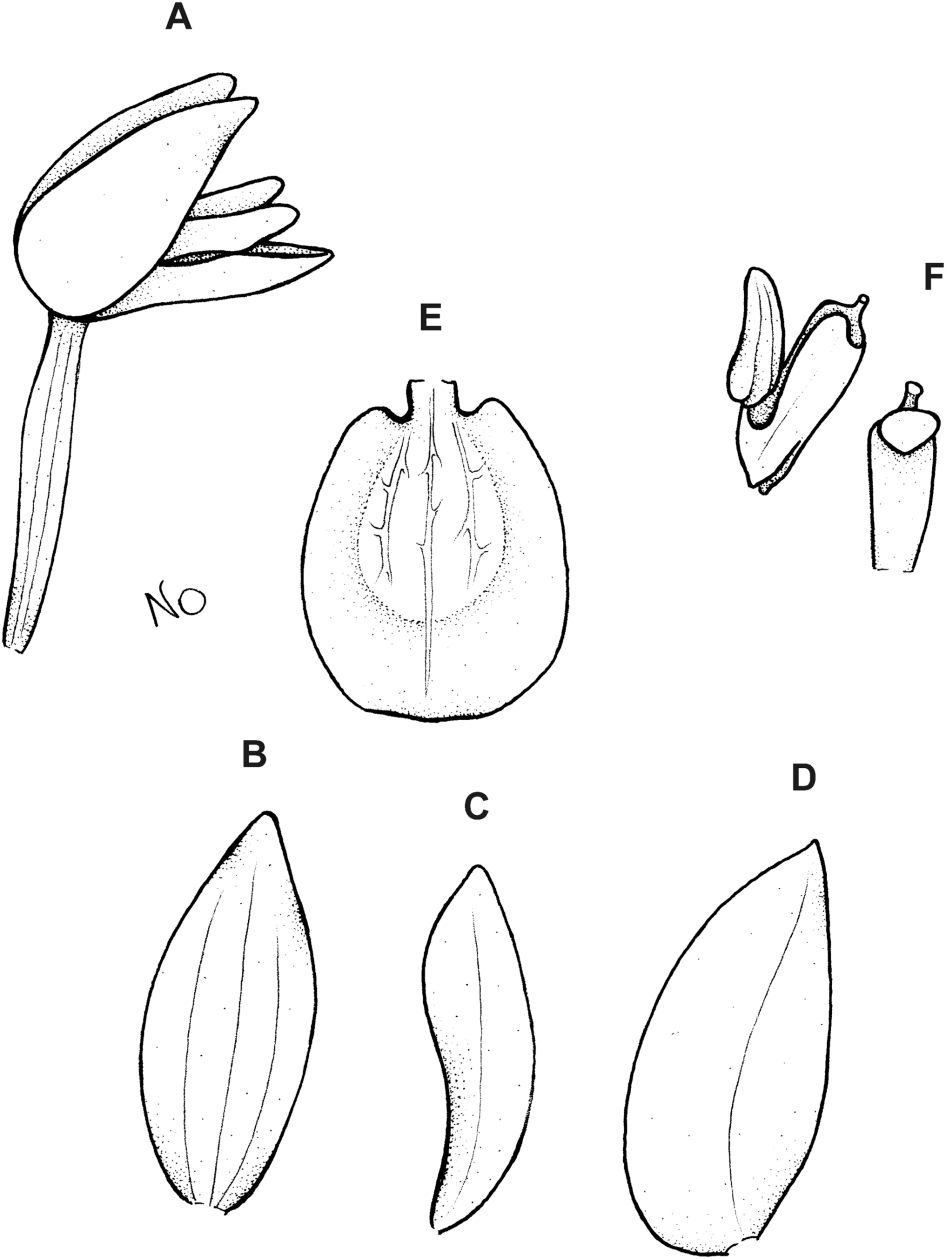
*Cranichis fendleri* Schltr. (A) Flower; (B) dorsal sepal; (C) petal; (D) lateral sepal; (E) lip (front view); (F) gynostemium. Redrawn by N. Olędrzyńska according to the original illustration from Schlechter.

**Figure 52:**
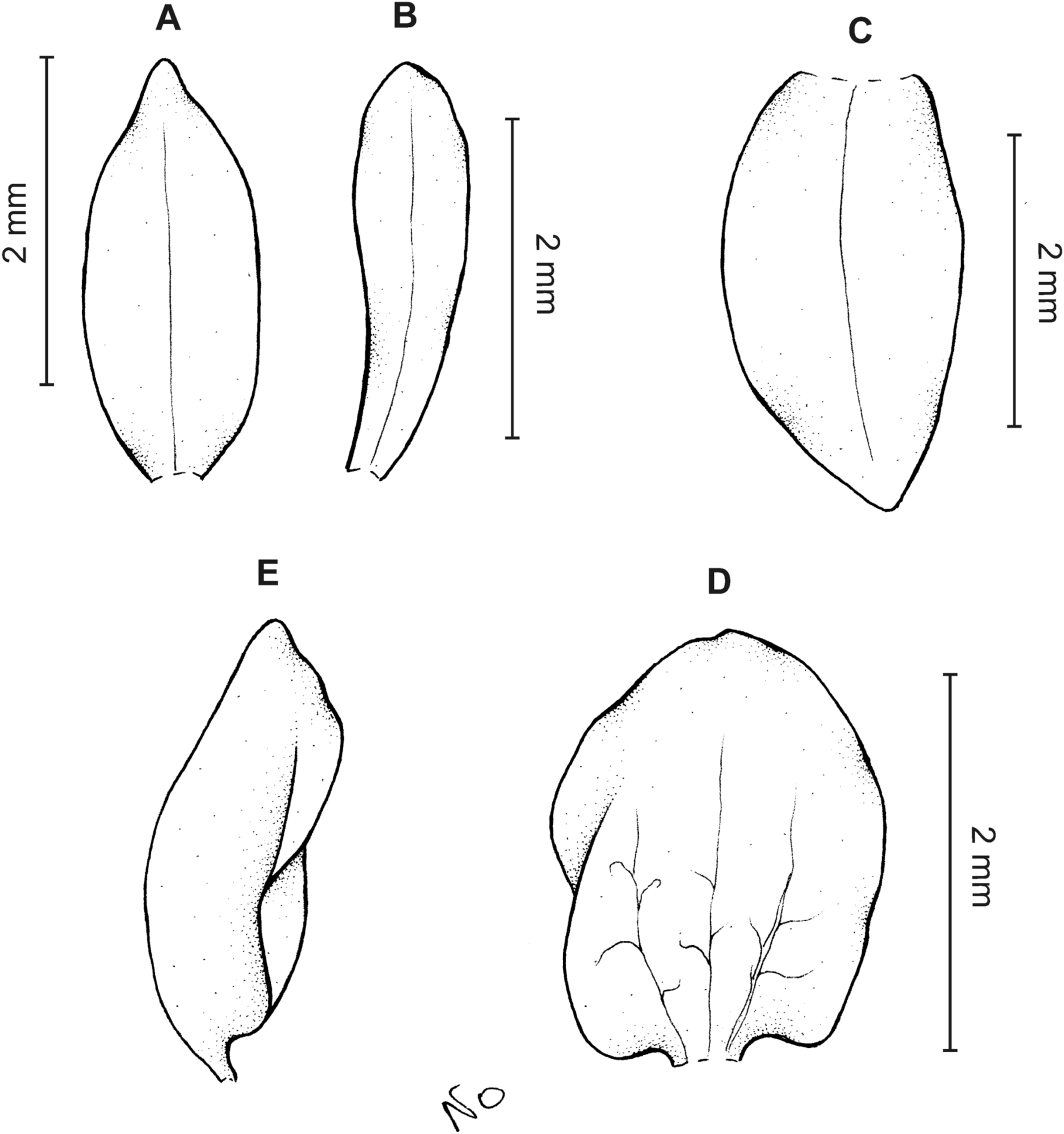
*Cranichis fendleri* Schltr. (A) Dorsal sepal; (B) petal; (C) lateral sepal; (D) lip (front view); (E) lip (side view). Drawn by N. Olędrzyńska from *Pittier 11281* (AMES).

Plants 17–35 cm tall. Leaves 1–4, basal, erect-patent, petiolate; petiole 0.8–7 cm long; blade 2.8–10.5 cm long, 1.8–5 cm wide, elliptic, shortly acuminate, base truncate-subcordate. Scape delicate, straight or almost straight, terete, apically glandular-puberulent, enclosed in five acuminate sheaths. Inflorescence 3–10 cm long, cylindrical, densely many-flowered. Flowers whitish, glabrous. Floral bracts three to four mm long, ovate-lanceolate, acuminate, glabrous to sparsely glandular. Pedicellate ovary about 5.5–6 mm long, cylindrical, glabrous or sparsely glandular. Dorsal sepal two to three mm long, 1.1 mm wide, ovate, obtuse, 1- or 3-veined. Petals 2–2.7 mm long, 0.6–0.7 mm wide, oblanceolate, falcate, obtuse, 1-veined. Lateral sepals 2.5–3 mm long, 1.5–1.8 mm wide, obliquely ovate, subobtuse, 2- or 1-veined. Lip 2–3 mm long, 1.7 mm wide, concave, shortly unguiculate, basally cordate, broadly ovate to elliptic, obtuse, margins recurved; disc with three parallel, branching veins with more or less prominent nodules. Gynostemium 1.1–1.6 mm long (Figs. 51 and 52).

*Ecology*: Terrestrial plant growing in wet forest and along roadsides at altitudes of 1,300–3,300 m. Flowering throughout the year.

*Distribution*: Peru, Colombia, Venezuela.

*Representative specimens*: COLOMBIA. **Cauca**: Wet glen in forest, San José. San Antonio. Cordillea Occidental, 2,400–2,700 m, June 28, 1922, *F. Pennell & E. Killip 7330* (AMES!, UGDA-DLSz!—drawing); Forest, San José, San Antonio. Cordillera Occidental, 2,400–2,780 m, July 1, 1922, *F. Pennell 7597a* (AMES!). **Huila**: Cordillera Oriental. E of Neiva, 1,800–2,300 m, August 1–8, 1917, *H. Rusby & F. Pennell 584* (AMES!); Cordillera Oriental. E of Neiva, 1,300–1,800 m, August 1–8, 1917, *H. Rusby & F. Pennell 944* (AMES!). **Norte de Santander**: Hacienda La Esperanza. 10 km NW. Cordillera Oriental, 2,160 m, December 23, 1943, *F. Hermann 10924* (AMES!). **Putumayo**: Along roadside between Sibundoy and Pepino, 3,300 m, July 26, 1960, *L. Garay 58* (AMES!). **Santander**: Vicinity of La Baja, 3,100 m, January 14–31, 1927, *E. Killip & A. Smith 18141* (AMES!); Vicinity of Las Vegas, 2,600–3,000 m, December 21–23, 1926, *E. Killip & A. Smith 16149* (AMES!) (Fig. 50).

*Other materials examined*: PERU. **Huanuco**: 46 km from Huanuco along road to Tingo María (the pass Carpich ca. 45 km from N), 2,550 m, *K. Rahn 00306* (C!, UGDA-DLSz!—drawing). VENEZUELA. Prope coloniam Tovar. 1856–1857. *A. Fendler 2135* (G, AMES!). **Miranda**: Quebrada de Turumo, near Guarenas, December 2, 1923, *H. Pittier 11281* (AMES!, UGDA-DLSz!—drawing).

*Notes*: It differs from *C. diphylla* by having 1-veined lateral sepals and usually glabrous ovary. In flower shape this species resembles somewhat *C. ciliata* (Schlechter, 1919), however, from this species *C. fendleri* can be easily distinguished by lip and petal shape. Lip of *C. fendleri* is basally (sub)cordate, often with prominent small auricles (vs. cuneate base of the lip of *C. ciliata*). The petals of this species are oblanceolate with glabrous margins, whereas petals of *C. ciliata* are oblong-lanceolate to narrowly-ligulate with margins unevenly pilose.

***Cranichis lehmannii*** Rchb.f., Otia Bot. Hamb. 1: 4. 1878. TYPE (Garay, 1978: 199): Ecuador. *Lehmann 77* (lectotype, W!; AMES!—drawing, UGDA-DLSz!—drawing).

**Figure 53:**
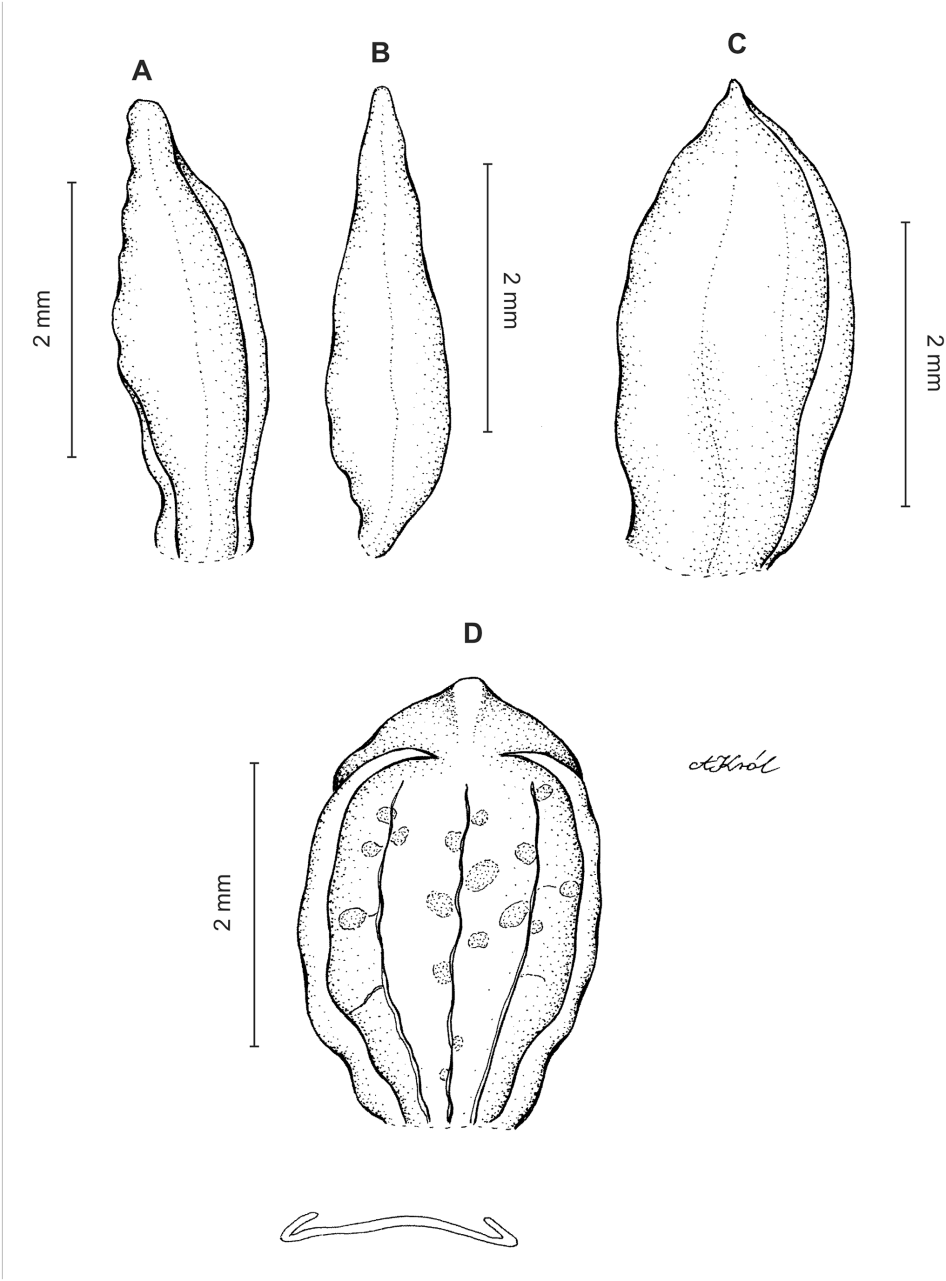
*Cranichis lehmannii* Rchb.f. (A) Dorsal sepal; (B) petal; (C) lateral sepal; (D) lip (front view). Drawn by A. Król from *Lehmann 77* (W-R).

**Figure 54:**
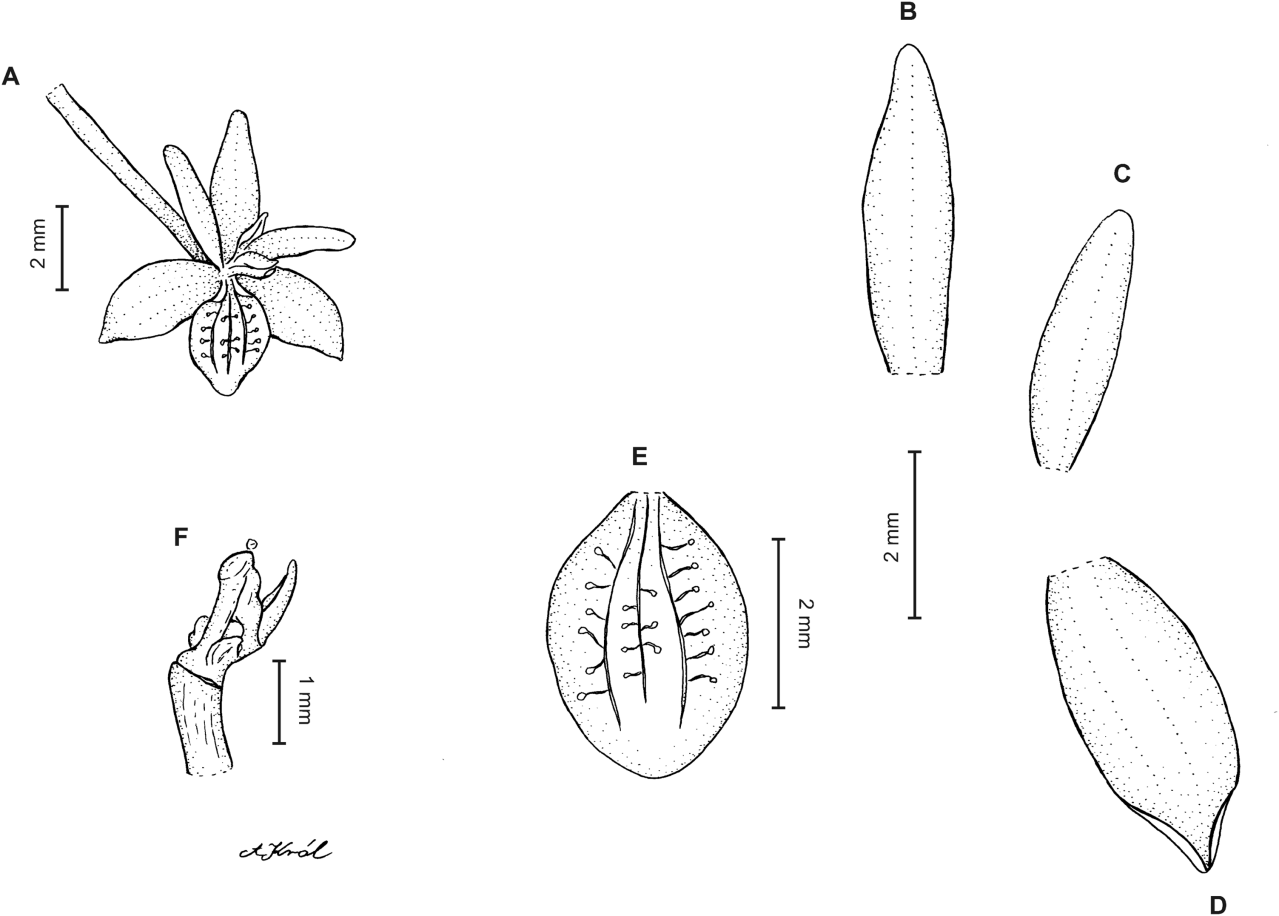
*Cranichis lehmannii* Rchb.f. (A) Flower; (B) dorsal sepal; (C) petal; (D) lateral sepal; (E) lip; (F) gynostemium. Redrawn by A. Król from Garay’s illustration of specimen collected by *Lehmann 77* (W).

Plants 26–60 cm tall. Leaves 1–3, basal, petiolate; petiole 3–4(8) cm long, narrow, canaliculate; blade 6.5–11 cm long, 2.8–5 cm wide, ovate, acute. Scape erect, enclosed in about six to nine sheaths. Inflorescence 2.5–10.5 cm long, conical, sublaxly many-flowered. Flowers small, glabrous. Floral bracts 4.5–8 mm long, lanceolate, acute. Pedicellate ovary six to nine mm long, almost glabrous. Dorsal sepal three to four mm long, 1–1.1 mm wide, oblong-lanceolate to oblong ovate, acuminate, obtuse, concave, 1-veined. Petals 2.5–3.5 mm long, 0.5–1.2 mm wide, lanceolate, somewhat oblique at base, subobtuse, 1-veined. Lateral sepals 3.5–4 mm long, 1.5–1.7 mm wide, obliquely elliptic-ovate to elliptic-lanceolate, subacute to subapiculate, concave in the center, obscurely 2-veined. Lip 3–3.3 mm long, 1.6–2.3 mm wide, concave, subsessile, elliptic to oblong-elliptic in outline, obtuse at apex, lateral margins reflexed; disc with numerous, irregularly subglobose thickenings on the inner surface, veins thickened dendritic branching. Gynostemium 1.2–1.5 mm long (Figs. 53 and 54).

*Ecology*: Terrestrial plants growing in forest and disturbed roadside vegetation at altitudes of 1,000–2,360 m. Flowering occurs in February, October, November.

*Distribution*: Peru (Garay, 1978), Ecuador, Colombia.

*Representative specimens*: COLOMBIA. **Antioquia**: Mpio. Frontino, Corregimiento de Nutibara, zona de Murrí, Alto de Cuevas, 1,000–1,850 m, February 14, 1991, *R. Callejas et al. 9899* (NY!—in fruit, HUA—Dueñas Gómez & Fernández Alonso, 2009); Mpio. Anori. Sitios El Río y Bramadero, km 1–9 sobre la vía de Anori-Dos Bocas, NE del Pueblo de Anori, 7°5′N 75°10′W, 12,901,510 m, November 16, 1989, *R. Callejas et al. 8674* (NY!); Mpio. Heliconia. 16 km NW de San Antonio de Prado, en la vía Medellín-Heliconia, bosque a orilla de Canada, 6°13′N 75°39′W, 1,710 m, October 19, 1990, *R. Callejas et al. 9541* (NY!, UGDA-DLSz!—drawing); Mpio. La Unión. Km 16 of road la Unión-Sonson (40 km before Sonson), disturbed roadside vegetation, 5°54′N 75°20′W, 2,360 m, November 9, 1988, *J.L. Zarucchi & F. Roldan 7262* (MO!, UGDA-DLSz!—drawing) (Fig. 50).

*Other materials examined*: ECUADOR. Tungurahua, January 1877, *F.C. Lehmann 77* (W!).

*Notes*: This species can be conspecific with *C. cylindrostachys* (cf. Garay, 1978). As we noted, both can be distinguished only by the length and shape of the inflorescence, which is shorter in *C. lehmannii* (2.5–10.5 cm long, conical vs. 11–15 cm long, cylindrical).

***Cranichis monophylla*** Lindl., Orchid. Linden.: 27. 1846. TYPE: Venezuela. *Linden s.n*. (lectotype, designated by Garay (1978: 192) K!).

*Sauroglossum monophyllum* (Lindl.) Griseb., Cat. Pl. Cub.: 269. 1866.
*Spiranthes monophylla* (Lindl.) Cogn. in I.Urban, Symb. Antill. 6: 339. 1909.
*Cyclopogon monophyllus* (Lindl.) Schltr., Beih. Bot. Centralbl. 37(2): 391. 1920.

**Figure 55:**
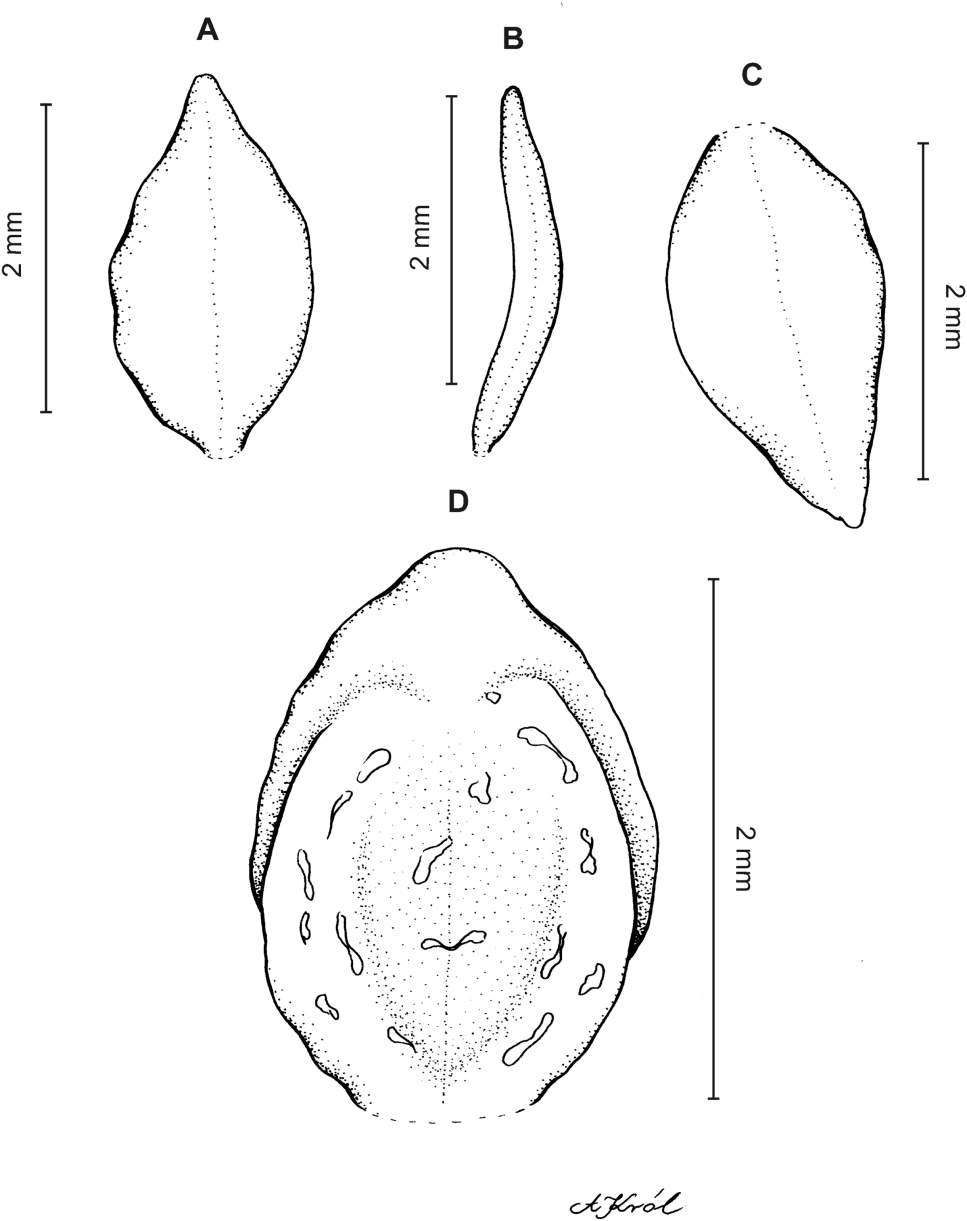
*Cranichis monophylla* Lindl. (A) Dorsal sepal; (B) petal; (C) lateral sepal; (D) lip (front view). Drawn by A. Król from *Moritz 1107* (W-R).

Plant 30 cm tall. Leaves 2, basal, erect-patent, petiolate; petiole 1.2 cm long, narrow, canaliculate; blade four cm long, 2.3 cm wide, ovate to oblong ovate, base rounded, acute to shortly acuminate. Scape erect or slightly flexuose, with five to eight sheaths, apically densely glandular. Inflorescence 6.5 cm long, cylindrical, subdensely many-flowered. Flowers small, inconspicuous. Floral bracts three to four mm long, lanceolate, acuminate, glandular. Pedicellate ovary five to seven mm long, cylindric, glandular. Dorsal sepal 2.5–2.6 mm long, 1.1–1.3 mm wide, oblong ovate, obtuse, glabrous, 1-veined. Petals 2.5 mm long, 0.4–0.9 mm wide, linear, subfalcate, obtuse, 1-veined. Lateral sepals 2.5–3.2 mm long, 1.3–1.5 mm wide, obliquely oblong ovate, subacute, glabrous, 1-veined. Lip 2.2–2.5 mm long, 1.5–1.8 mm wide, concave, elliptic, widest near the middle or above, rounded; disc 3-veined, branching, with small nodules. Gynostemium 1.2–1.8 mm long (Fig. 55).

*Ecology*: Terrestrial plants growing in montane forest at an altitude of 2,200–2,500 m. Flowering occurs in June and September.

*Distribution*: Colombia, Venezuela.

*Representative specimens*: COLOMBIA. **Cauca**: Forest between S[…] and Río Vinagre, 2,200–2,500 m, June 10–11, 1922, *F. Pennell & E. Killip 6470b* (AMES!, UGDA-DLSz!—drawing). **Risaralda**: Mun. de Pueblo Rico. Cerro Montezuma, 5°14′59″N, 76°06′35″W, ca. 2,500 m, September 30, 2006, *R. Arévalo et al. 657* (COL) (Fig. 50).

*Other materials examined*: VENEZUELA. **Mérida**: *J. Linden s.n*. (K!), 1844–1845, *J. Moritz 1107* (W!, UGDA-DLSz!—drawing).

*Notes*: *C. monophylla*, *C. stictophylla*, and *C. ovatilabia* are morphologically similar species and rather difficult to determine. As far as we could detect, they can be distinguished by means of the sepal surface, which is papillate on the outside in *C. ovatilabia*, and smooth in both *C. monophylla* and *C. stictophylla*. Further study should reveal if this character provides proper determination.

According to Garay (1978) it is conspecific with *C. diphylla*. In our opinion, however, both species show some differences that can be of taxonomic value. In *C. diphylla* the leaf base is subcordate (vs. rounded in *C. monophylla*), scape is glandular-pubescent in the upper part (vs. densely glandular), dorsal sepal is 3- and lateral sepals are 2-veined (vs. sepals 1-veined in *C. monophlla*), and lip disc is papillose-thickened (vs. glabrous). Further studies should reveal whether these characters warrant separate status for *C. monophylla*.

***Cranichis muscosa*** Sw., Prodr.: 120. 1788. TYPE: Jamaica. *Swartz s.n*. (lectotype, designated by Garay (1978: 202) BM!; isolectotype, W!).
*Cranichis ovata* Wickstr., Kongl. Vetensk. Acad. Handl. 73. 1920.

**Figure 56:**
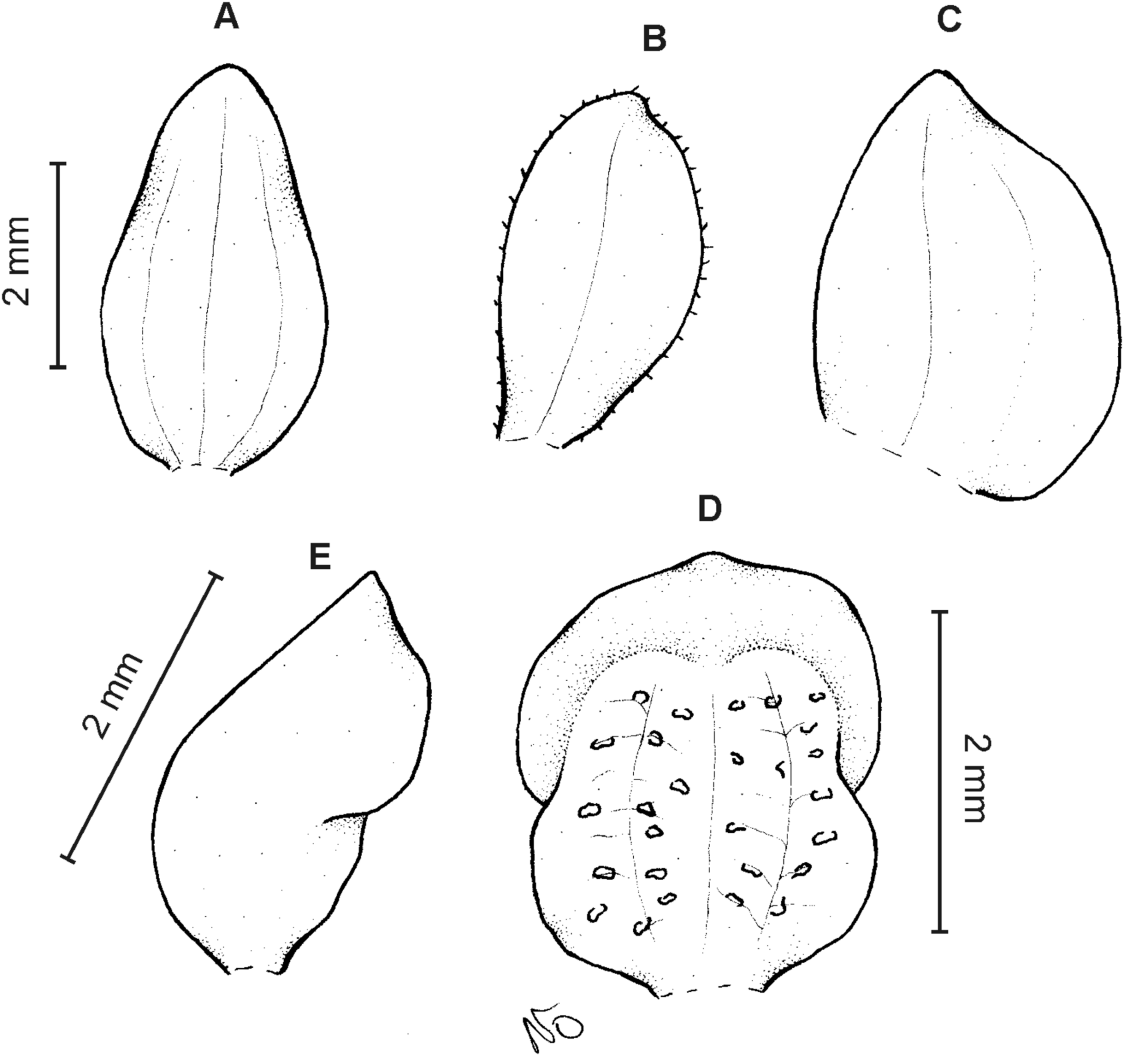
*Cranichis muscosa* Sw. (A) Dorsal sepal; (B) petal; (C) lateral sepal; (D) lip (front view); (E) lip (side view). Drawn by N. Olędrzyńska from *Garay & Sweet 1057* (AMES).

Plants up to 25 cm tall, erect or flexuose. Leaves 3–5, basal, rosulate, petiolate; petiole two to three cm long; blade 2.5–3 cm long, up to two cm wide, ovate, elliptic-ovate to oblong, acute to subobtuse. Scape slender, enclosed in five sheaths. Inflorescence up to 7.5 cm long, cylindric, subdensely many-flowered. Flowers small, white. Floral bracts four to five mm long, lanceolate to ovate-lanceolate, acuminate. Pedicellate ovary five to six mm long, glabrous. Dorsal sepal 2.2 mm long, one mm wide, oblong-lanceolate to oblong-ovate, acute, 3-veined. Petals two mm long, 0.6 mm wide, linear-ligulate to narrowly oblanceolate, obtuse, subfalcate, margins ciliate, 1-veined. Lateral sepals three mm long, 1.8 mm wide, obliquely oblong-ovate to elliptic-ovate, acuminate, acute, obscurely 2-veined. Lip 2.2 mm long, 1.87 mm wide, concave, subsessile, ovate to suborbicular-ovate, shortly apiculate to acute; disc with irregular knob-like projections in the center. Gynostemium two mm long (Fig. 56).

*Ecology*: Terrestrial in wet forest at altitudes of 800–2,800 m. Flowering in January, February, and October.

*Distribution*: USA (Florida; Ames & Correll, 1952), Mexico, Belize (Ames & Correll, 1985), Guatemala (Ames & Correll, 1952), Costa Rica (Pupulin, 2005), Panama (Correa, Galdames & Stapf, 2004), Cuba (Llamacho & Larramendi, 2005), Jamaica, Martinique, Peru (Schweinfurth, 1958), Ecuador (Garay, 1978), Colombia, Venezuela (Foldats, 1969), Trinidad (Schultes, 1960).

*Representative specimens*: COLOMBIA. **Antioquia**: Vicinity of Sonsón, 2,400–2,800 m, January 23, 1970, *L. Garay & H. Sweet 1057* (AMES!, UGDA-DLSz!—drawing). **Boyacá**: Santa María, 800 m, October 8, 2000, *J. Betancur 8748* (COL!, UGDA-DLSz!—drawing, Dueñas Gómez & Fernández Alonso, 2009). **Chocó**: Carretera Ansermanuevo-San José del Palmar, 8.4 km del Alto del Galápago, 1,600 m, February 19, 1977, *E. Forero et al. 3016* (COL—Dueñas Gómez & Fernández Alonso, 2009) (Fig. 50).

*Other materials examined*: CUBA. In Cuba Orientali, 1860, *C. Wright 620 & s.n*. (W!); Cahobas*, in sylvis obscures ad rupes* muscosas, October 1823, *E. Poeppig s.n*. (W!). MARTINIQUE. Morre Coco, January 1868, *L. Hahn 90* (W!). JAMAICA. *Sine loc., W. Morris s.n*. (W!).

*Notes*: Distinguished by the foliaceous scape, the glabrous ovary, the lip with membranaceous margin, the disc of the lip 3-veined, papillose-verrucose, the 1-veined, linear-oblong petals that are minutely ciliolate on the margin, the dorsal sepal 3-veined, and lateral sepals 2-veined.

According to Garay (1978), *C. ovata* Wickstr. is conspecific with *C. muscosa*, but we did not have access to adequate material of the former species to confirm this concept.

***Cranichis nigrescens*** Schltr., Repert. Spec. Nov. Regni Veg. 10(263–265): 482. 1912. TYPE: Costa Rica. *Tonduz s.n*. (B†; lectotype, designated by Garay (1978: 192) AMES!—drawing).

**Figure 57:**
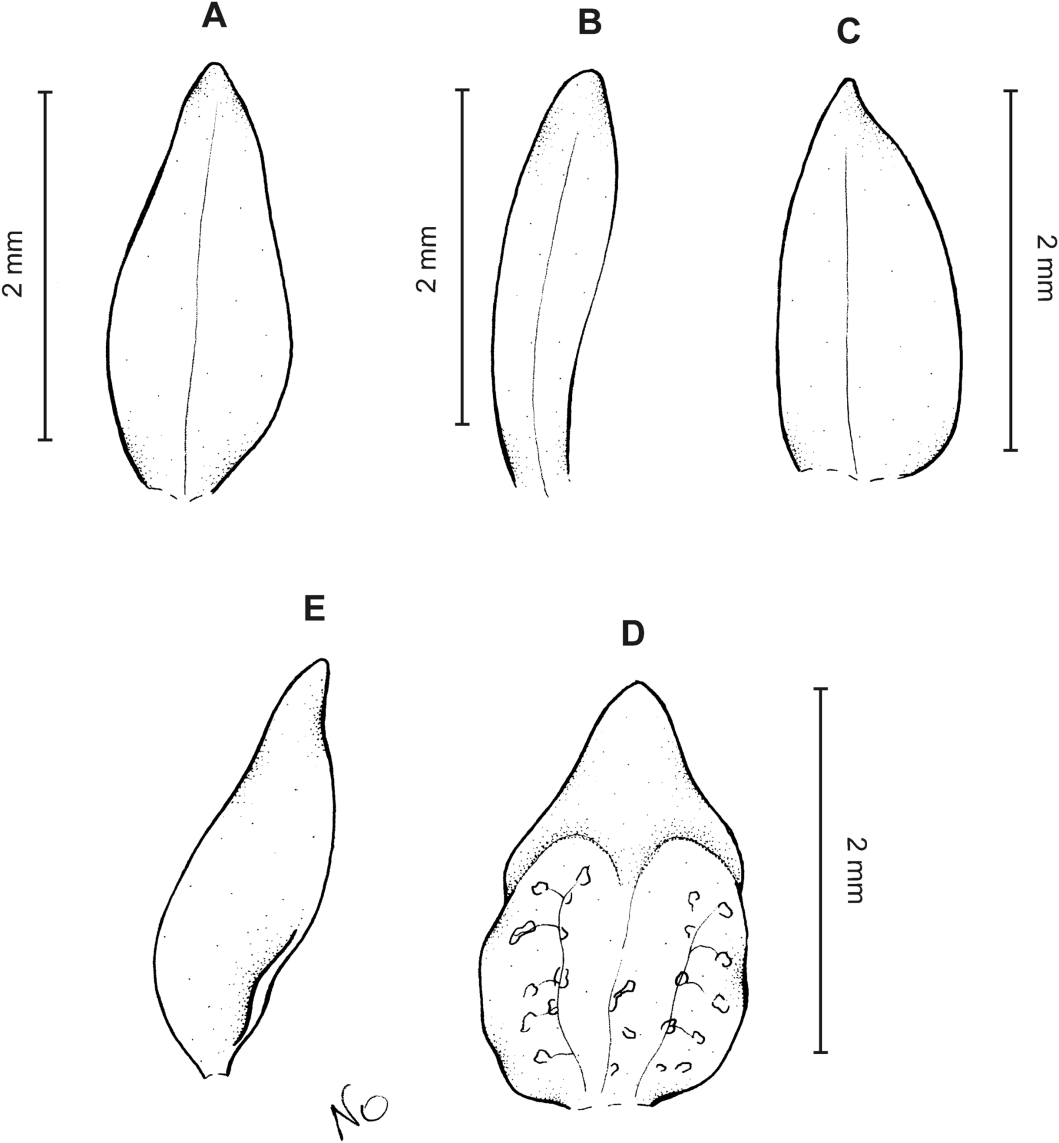
*Cranichis nigrescens* Schltr. (A) Dorsal sepal; (B) petal; (C) lateral sepal; (D) lip (front view); (E) lip (side view). Drawn by N. Olędrzyńska from *Ospina H. 60* (AMES).

Plant 12–30 cm tall. Leaf 1, rarely 2, basal, erect-patent, petiolate; petiole 1–6(10) cm long, narrow, canaliculate; blade 2.2–6 cm long, 1.2–3 cm wide, ovate to oblong, base rounded to subcordate, acute to shortly acuminate. Scape erect or slightly flexuose, with five to eight sheaths, apically densely glandular-hispid. Inflorescence 2.5–4 cm long, cylindrical, subdensely many-flowered. Flowers small, inconspicuous. Floral bracts four to seven mm long, lanceolate, acuminate, sparsely glandular or glabrous. Pedicellate ovary 3.25–6 mm long, cylindric, glandular-hispid. Dorsal sepal 2.5–3.1 mm long, 0.9–1.1 mm wide, ovate-lanceolate, subacute, glabrous, 1-veined. Petals 2.5–3 mm long, 0.6–0.7 mm wide, linear, subfalcate, obtuse, 1-veined. Lateral sepals 2.3–4 mm long, 1.1–1.4 mm wide, obliquely ovate-lanceolate, subacute, glabrous, 1-veined. Lip 2.25–3 mm long, 1.25–2.1 mm wide, concave, ovate, widest at the base, subacute to subobtuse; disc 3-veined, branching, with several thickened nodules. Gynostemium 1.3–1.8 mm long (Fig. 57).

*Ecology*: Lithophytic or terrestrial plant growing in forest and shrubland at altitudes of 2,500–3,300 m. Flowering occurs in June, November, and December.

*Distribution*: Costa Rica, Colombia.

**Figure 58:**
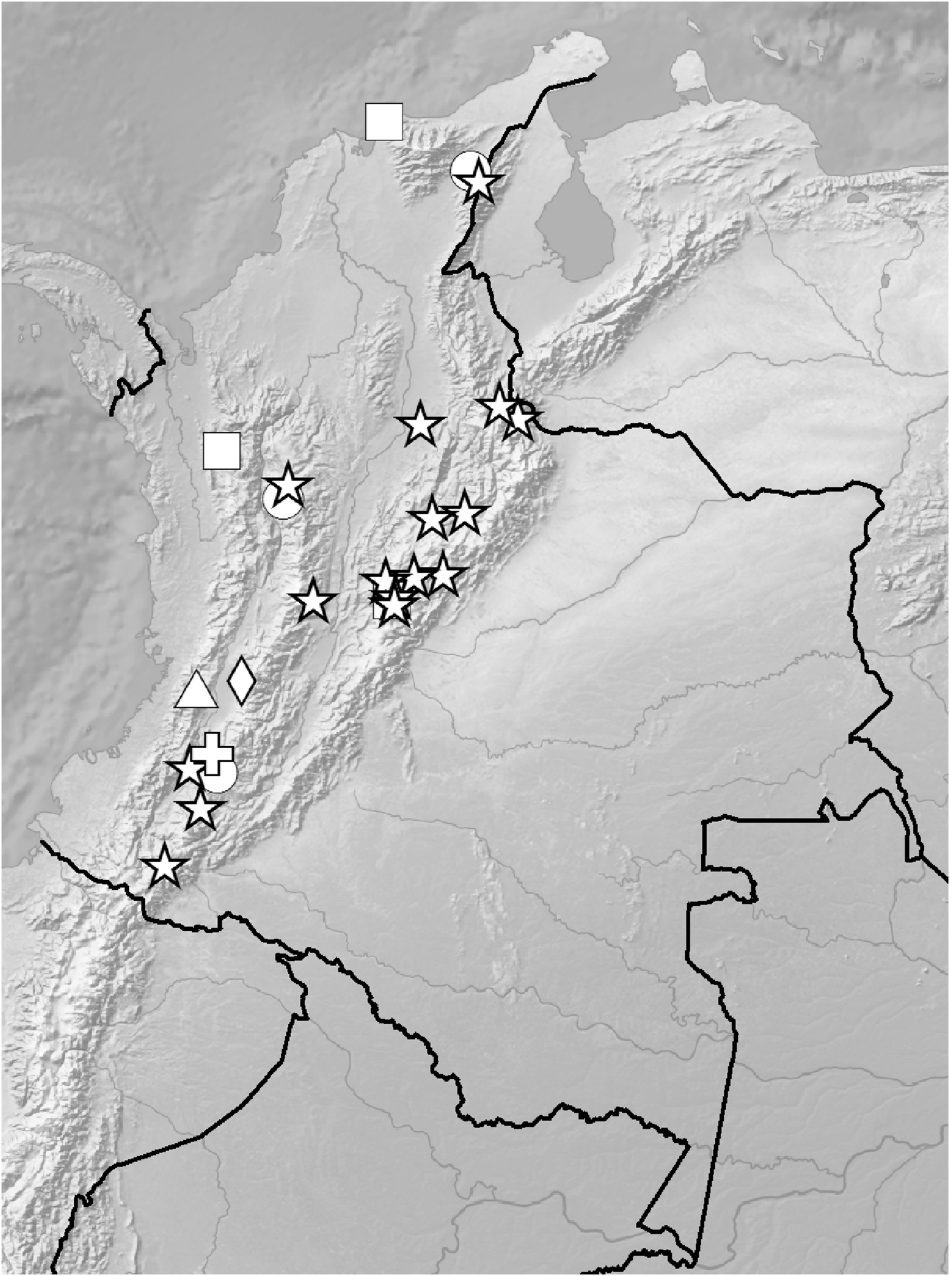
Distribution of members of the *Cranichis Diphylla* group. *C. nigrescens* (triangle), *C. parvula* (square), *C. pseudomuscosa* (star), *C. nigrescens* (circle), *C. queremalensis C. schlimii* (diamond), *C. stictophylla* (cross).

*Representative specimens*: COLOMBIA. **Antioquia**: Mpio. El Retiro. Hacienda Normandía, 2,500 m, December 2–3, 1956, *M. Ospina H. 60* (AMES!, UGDA-DLSz!—drawing). **Cauca**: Cannan. Mt. Puracé, 3,100–3,300 m, June 11–13, 1922, *F.W. Pennell & E.P. Killip 6595* (AMES!, NY!, UGDA-DLSz!—drawing). **Magdalena**?[**Guajíra**]. Sierra de Perija, E of Manaure, Quebrada de Florida blanca. Andean forest and bushes, 2,700–2,800 m, November 10, 1959, *J. Cuatrecasas & R. Romero-Castaneda 25188* (US!, UGDA-DLSz!—drawing) (Fig. 58).

*Other materials examined*: COSTA RICA. Ohne nähere Standortsangabe (without specification of location), *A. Tonduz s.n*. (AMES!).

*Notes*: It differs from *C. schlimii* by having 1-veined sepals (vs. dorsal sepal 3-veined and lateral sepals either 3- or 2-veined) and form of the lip, which is ovate, widest at the base, subacute to subobtuse at apex (vs. subrectangular to oblong-elliptic in outline, obtuse to slightly notched at the apex).

This species is often reduced to the synonymy of *C. diphylla*. Both species, however, can be distinguished by a series of characters: scape of *C. nigrescens* is densely glandular-hispid in the upper part (vs. glandular-pubescent in *C. diphylla*), pedicellate ovary is glandular-hispid (vs. more or less glandular), sepals are 1-veined (vs. dorsal sepal 3-veined, and lateral sepals 2-veined), and lip is glabrous inside (vs. papillose-thickened).

Dried plants become black.

***Cranichis ovatilabia*** Schltr., Repert. Spec. Nov. Regni Veg., Beih. 7: 59. 1920. TYPE: Colombia. *Madero s.n.* (B†).

**Figure 59:**
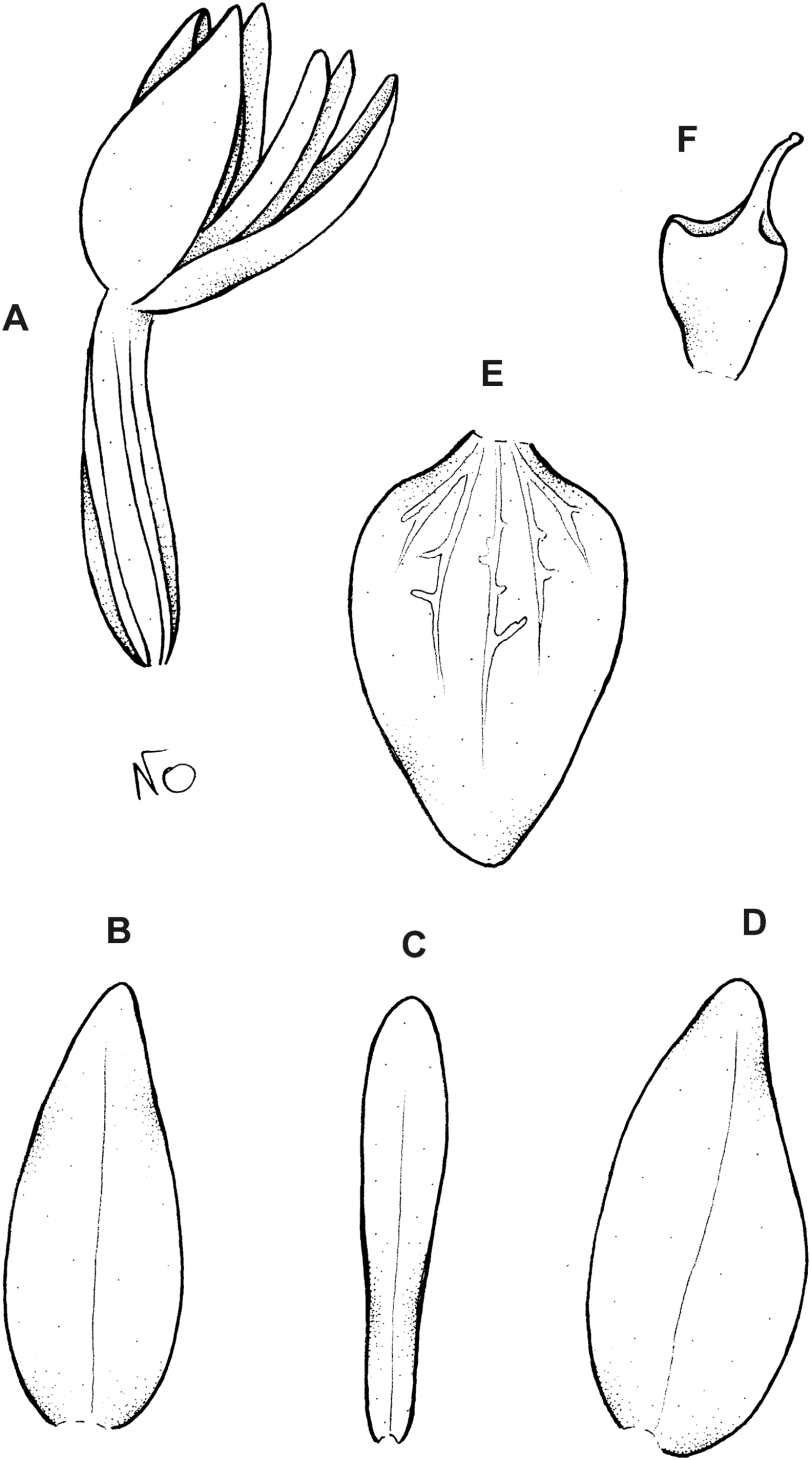
*Cranichis ovatilabia* Schltr. (A) Flower; (B) dorsal sepal; (C) petal; (D) lateral sepal; (E) lip (front view); (F) gynostemium. Redrawn by N. Olędrzyńska according to the original illustration from Schlechter.

Plants 20–25 cm tall, erect. Leaf single, basal, petiolate; petiole 2.3–2.5 cm long; blade four cm long, 1.8 cm wide, ovate, acuminate, with rounded base. Scape glandular toward the apex, enclosed in about five sheaths. Inflorescence four to five cm long, cylindric, sublaxly 10–15-flowered. Flowers tiny, sepals sparsely glandular. Floral bracts up to ca. six mm long, elliptic-lanceolate, acuminate. Pedicellate ovary five mm long, densely glandular. Sepals papillate on the outer surface. Dorsal sepal three mm long, one mm wide, lanceolate to oblong ovate, obtuse, papillate on the outside, 1-veined. Petals three mm long, 0.5 mm wide, linear-oblanceolate to narrowly ligulate-oblanceolate, obtuse, glabrous on margins, 1-veined. Lateral sepals three mm long, 1.2 mm wide, obliquely elliptic-ovate, slightly concave at the base, subacuminate, subobtuse, papillate on the outside, 1-veined. Lip 2.75 mm long, 1.75 mm wide, concave, subsessile, ovate, obtuse; disc with five thickened, dendritic, branching veins disappearing near the middle of the lip. Gynostemium ca one mm long (Fig. 59).

*Ecology*: Terrestrial plant growing at an altitude of 2,500 m. No data on flowering time available.

*Distribution*: Colombia.

*Representative specimen*: COLOMBIA. **Antioquia**: *Sine loc*., 2,500 m, *M. Madero s.n*. (B†; AMES!—drawing).

*Notes*: According to Schlechter (1920) this species is similar to *C. stictophylla*, but is a smaller plant, with ovate leaves, shorter inflorescence, broadly ovate lip and different shape of sepals and rostellum. Unlike *C. stictophylla*, sepals of this species are papillate on the outside. We know *C. ovatilabia* only from an original drawing kept at AMES.

According to Garay (1978) this species is conspecific with *C. diphylla*, but it can be distinguished from the latter by having leaves with rounded base (vs. subcordate base), scape glandular toward apex (vs. glandular-pubescent), 1-veined sepals papillate on the outside (vs. sepals either 3- or 2-veined, occasionally sparsely pubescent on the outer surface) and lip with broadly cuneate base (vs. subcordate base), and glabrous disc (vs. papillose-thickened).

Garay (1978) designated this as lectotype of the *C. stictophylla* drawing made under Schlechter’s superivision of the *Madero 162*. Schlechter (1920), however, mentioned in the protologue *Madero s.n*.

***Cranichis parvula*** Renz, Candollea 11: 259, fig. 8B. 1948. TYPE: Colombia. *Renz 4124* (lectotype, designated by Garay (1978: 20) RENZ).

**Figure 60:**
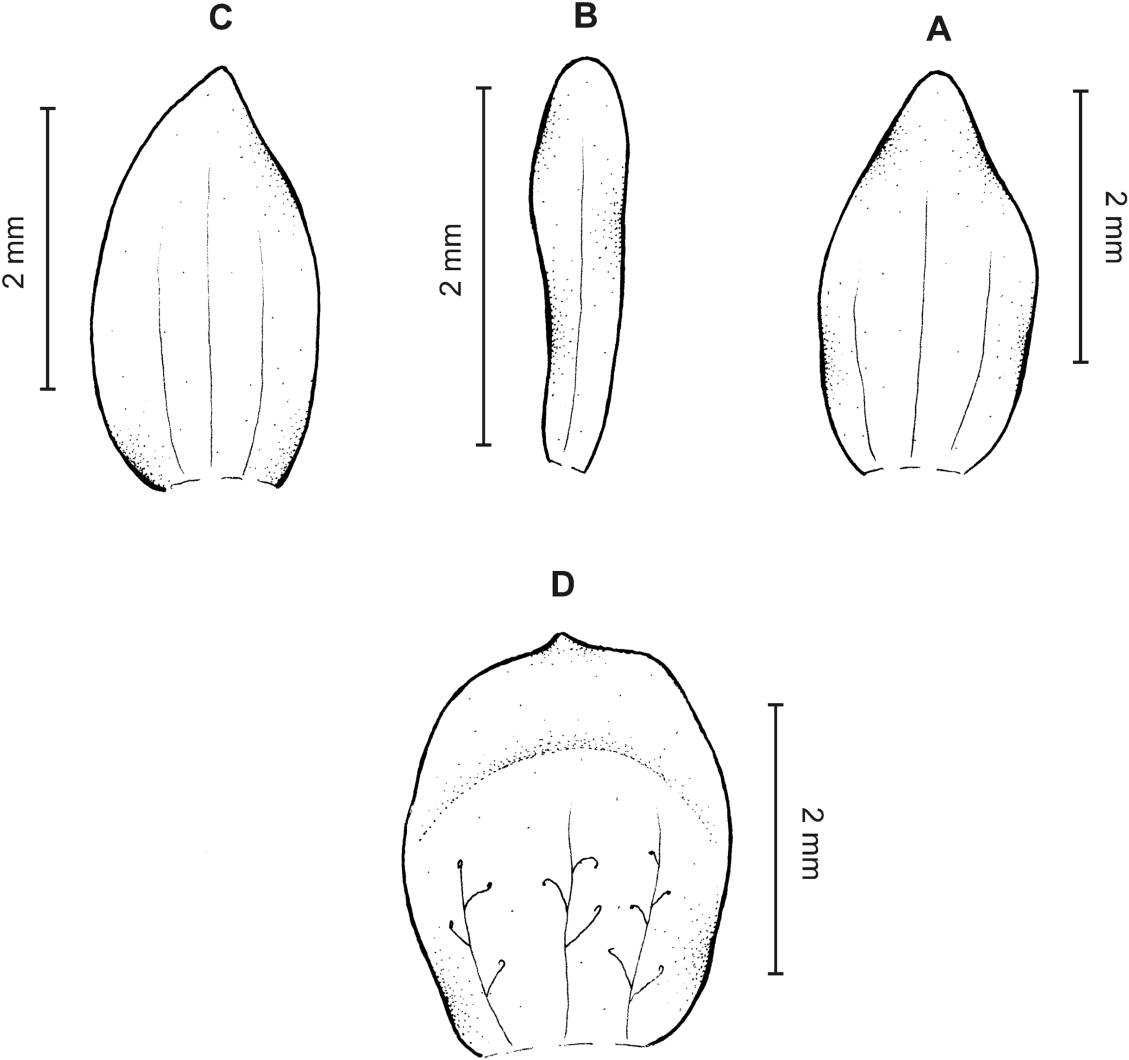
*Cranichis parvula* Renz. (A) Dorsal sepal; (B) petal; (C) lateral sepal; (D) lip (front view). Drawn by N. Olędrzyńska from *Smith 2553* (US).

**Figure 61:**
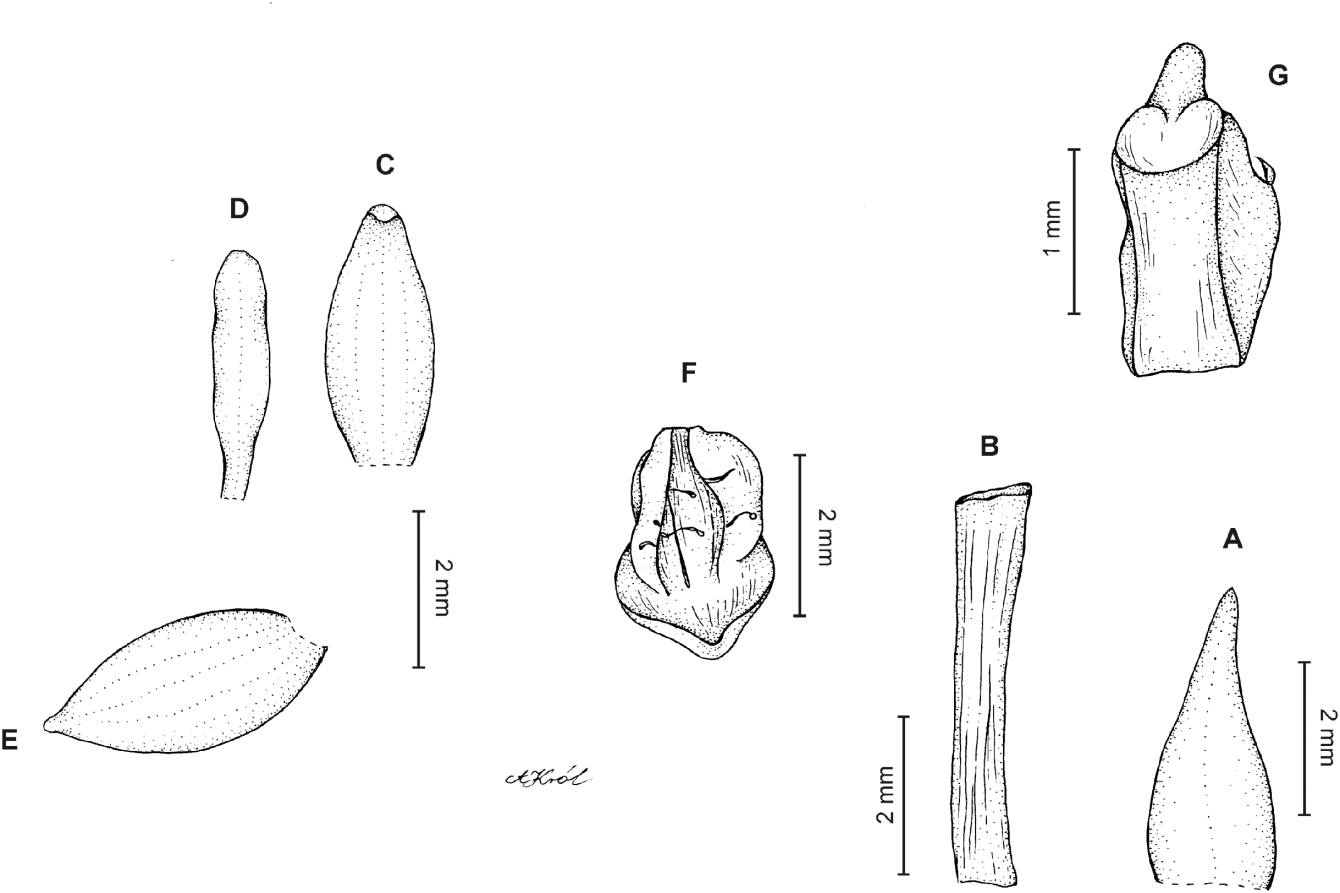
*Cranichis parvula* Renz. (A) Floral bract; (B) ovary; (C) dorsal sepal; (D) petal; (E) lateral sepal; (F) lip; (G) gynostemium. Redrawn by A. Król from Garay’s illustration of a specimen collected by *Renz 4159* (RENZ).

Plants up to 32(40) cm tall, slender. Leaves 4–7, basal, petiolate; petiole 2–6.5 cm long, narrow, canaliculated; blade four to eight cm long, 2–3.7 cm wide, ovate to ovate-elliptic, acute, rounded at base. Scape erect, remotely several-sheathed; sheaths foliaceous. Inflorescence 2.5–6 cm long, cylindrical, loosely many-flowered. Flowers greenish white, glabrous. Floral bracts four to five mm long, ovate-lanceolate, acuminate, glabrous. Pedicellate ovary six to eight mm long, cylindrical, glabrous. Dorsal sepal 2.6–3.8 mm long, 1–1.8 mm wide, elliptic-ovate, acute to obtuse, 3-veined. Petals 2.2–4 mm long, 0.6–0.9 mm wide, ligulate to oblong-ligulate, somewhat falcate, acute, l-veined. Lateral sepals 3–4.3 mm long, 2–2.2 mm wide, obliquely ovate, acute, prominently 3-veined. Lip 3–4.2 mm long, 2.3–3.2 mm wide, concave, subsessile, elliptic-ovate, obtuse; disc with three veins, lateral ones branching, from base to middle more or less cushionlike, ballooned out. Gynostemium 1.8 mm long (Figs. 60 and 61).

*Ecology*: Terrestrial plants growing at the forest edge at altitudes of 500–2,000 m. Flowering occurs in October and December.

*Distribution*: Ecuador (Garay, 1978), Colombia, Venezuela.

*Representative specimens*: COLOMBIA. **Antioquia**: Mpio. Frontino. Region of Murri. Ca. 13 road-km from Nutibara. Forets edge E of road, 6°40′N 76°20′W, 2,000 m, December 9, 1988, *G. McPherson 13411* (MO!, UGDA-DLSz!—drawing). **Cundinamarca**: Bogotá, *J. Ordóñez et al. 1782* (COL!, UGDA-DLSz!—drawing). **Magdalena**: Santa Marta, 1898–1901, *H.H. Smith 2553* (US!, UGDA-DLSz!—drawing). Meta: Int. Meta, 500–700 m, October 10, 1939, *O. Renz 4124* (Renz, 1948) (Fig. 58).

*Other materials examined*: VENEZUELA. **Mérida**, ca. 900 m, November 2, 1949, *O. Renz 6027* (RENZ); 500 m, October 29, 1949, *O. Renz 6017* (RENZ). **Sucre**. Dtto. Marino/Dtto. Arismendi. Península de Paria. Along rocky Río Tacarigua, 560 m, February 23, 1980, *J. Steyermark 121599* (MO!, UGDA-DLSz!—drawing).

*Notes*: Distinguished by a foliaceous scape, glabrous ovary, 3-veined dorsal sepal, lip with a membranaceous margin, disc of the lip 3-veined, papillose-verrucose, 1-veined, linear-oblong petals with entire margins, short inflorescence, broadly elliptic, loosely few-flowered, ovate lip, disc from base to middle cushion-like ballooned out.

***Cranichis pseudomuscosa*** Szlach. & Kolan., ***sp. nov***. TYPE: Colombia. *Schneider 250/2* (holotype, COL!; UGDA-DLSz!—drawing).
*Species distinguished from C. parvula by unifoliate stem, obtuse petals and suborbicular lip ornamented with five veins*.

**Figure 62:**
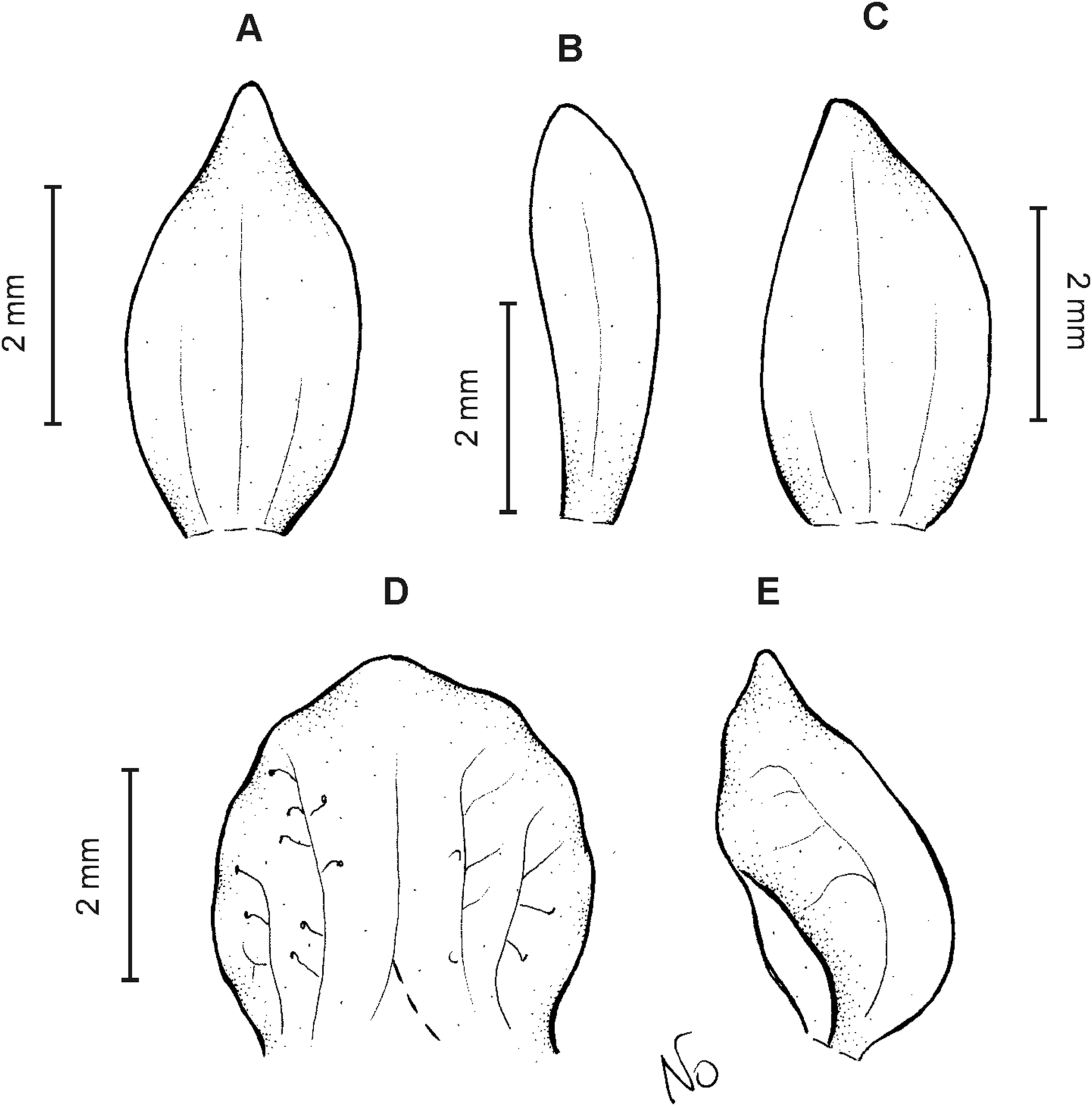
*Cranichis pseudomuscosa* Szlach. & Kolan. (A) Dorsal sepal; (B) petal; (C) lateral sepal; (D) lip (front view); (E) lip (side view). Drawn by N. Olędrzyńska from *Ceron & Alarcon 3530* (RPSC).

Plants 40–52 cm tall, slender. Leaf 1, basal, petiolate; petiole 7–10 cm long, narrow, canaliculated; blade 5.5–10 cm long, 3.2–4 cm wide, ovate to ovate-elliptic, acute, rounded at base. Scape erect, remotely several-sheathed; sheaths foliaceous. Inflorescence six to eight cm long, cylindrical, loosely to subdensely many-flowered. Flowers glabrous. Floral bracts five to six mm long, ovate-lanceolate, acuminate, glabrous to sparsely glandular. Pedicellate ovary 5.5–7 mm long, cylindrical, glabrous or sparsely glandular. Dorsal sepal 2.8–3.8 mm long, 1–1.9 mm wide, elliptic-ovate, subobtuse, 3-veined. Petals 2.5–4 mm long, 0.5–1 mm wide, ligulate-oblanceolate, somewhat falcate, obtuse, l-veined. Lateral sepals three to four mm long, 1.3–2 mm wide, obliquely ovate, acute, obscurely 2- or 3-veined. Lip 2.5–3.8 mm long, 1.8–3.8 mm wide, concave, subsessile, suborbicular, obtuse; disc with five veins, lateral ones branching, with numerous nodules. Gynostemium 1.5–2 mm long (Fig. 62).

*Etymology*: In reference to the superficial similarity to *C. muscosa*.

*Ecology*: Terrestrial in very humid montane forest, paramo, also very wet areas with grasses, shrubs, as well as in forest with *Prumnopytis* and *Podocarpus*. Growing at altitudes of 2,200–3,150 m. Flowering occurs in the second half of the year.

*Distribution*: Ecuador, Colombia, Venezuela.

*Representative specimens*: COLOMBIA. **Antioquia**: Mpio. de Medellín y Guarne. Parque Ecologico Piedras Blancas, sector Lajas, 6°18′N, 75°29′W, 2,350 m, December 10, 1994, *R. Fonnegra et al. 5334* (COL!, MO!). **Boyacá**: Mpio. de Duitama. Trayecto entre la vereda El Carmen (3,150 m) y Virolin (1,900 m). Páramo la Rusia. Bosques de robles en la mayor parte del trayecto, November 21, 1994, *J.L. Fernandez Alonso et al. 12084* (COL!); Mpio. de Chinavita. Cerro Mamapacha. Vereda Mundo Nuevo, 3,000 m, July 27, 2001, *H. Dueñas et al. s.n*. (COL!); Mpio. de Moniquirá. Vereda Colorado, sector alto. Río Pomeca. Bosque subandino, 2,200–2,300 m, July 9, 2001, *H. Dueñas 3088* (COL!). **Cauca**: Macizo Colombiano. Valle de Las Papas, alrededores de Valencia. In very wet area, with grasses, shrubs, etc. Los Andes, 3,140 m, September 27, 1958, *H.G. Barclay & P. Juajibioy 5818* (COL!); Macizo Colombiano. Valle de Las Papas, alrededores de Valencia. Los Andes, 2,910 m, September–October 1958, *J.M. Idrobo et al. 3963* (COL!); Parque Nacional Natural Munchique. El Tambo. Vereda La Romelia, camino al observatorio, 2,600 m, July 23, 1993, *F. Gonzalez et al. et al. 2928* (COL!). Cesar. Mpio. de Manaure. Serrania del Perija. Casa de Vidrio. 32 km SE de Manaure. Arriba de la Laguna, en bosque de *Prumnopytis* y *Podocarpus*, 10°25′N, 72°53′W, 2,950 m, November 5, 1993, *O. Rangel et al. 11078* (COL!). **Cundinamarca**: Mpio. de Sesquilé, 5°03′01.7″N, 73°46′22″W, 3,000 m, *M.P. Cordoba et al. 3006* (COL!); Mpio. de Subachoque. Finca El Cerro (Vereda El Tobal). En un bosque andino con presencia de *Weinmannia, Verbesina* y *Axinaea*, 2,920 m, October 24, 1998, *M. Hernandez Schmidt 357* (COL!); The same loc., December 5, 1998, *M. Hernandez Schmidt 374* (COL!); Mpio. de Subachoque. Vereda Tobal. Finca El Cerro. En un bosque de porte bajo con presencia *Weinmannia, Myrsine, Ilex, Ageratina* y *Cavendishia*. En sitio relativamente bien iluminado, suelo con musgo y hojarasca, 2,950 m, November 11, 2002, *M. Hernandez Schmidt 1032* (COL!); Usaquén, 2,800 m, November 24, 1946, *M. Schneider 250/2* (COL!, UGDA-DLSz!—drawing); Páramo de Usaquén, 3,000 m, October 12, 1951, *M. Schneider 250/3* (COL!); Usaquén, 2,700 m, November 11, 1950, *M. Schneider 250/4* (COL!); Macizo de Bogotá. Quebrada de Chicó. Matorroles musgosos, 2,800 m, October 10, 1943, *M. Schneider 250/1* (COL!). **Nariño**: Mpio. de Pasto. Corregimiento del Encano. Isla la Corota, 2,800 m, October 13, 1984, *O. de Benavides & B.R. Ramirez 4976* (COL!). **Norte de Santander**: Mpio. de Toledo. 26.4 km de San Bernardo de Bata en la vía a Saravena. Cuchilla Santa Ines, 2,200 m, November 1, 1994, *J.L. Fernandez Alonso et al. 11774* (COL!); Al oriente de Pamplona. Páramo de Fontibon, 2,800 m, November 8–9, 1969, *M.T. Murillo & R. Jaramillo M. 1297* (COL!). **Santander**: Mpio. de Charales. Vereda El Taladro. Km 50–55, carretera Duitama-Virolin, 2,250–2,300 m, December 6, 1978, *S. Díaz P. 1641* (COL!). **Tolima**: Mpio. de Sta. Isabel. Vereda de La Yuca. Cerca de Las Bodegas. Finca Buenavista. Alto La Esperanza. Cordillera Central, vertiente oriental, 2,700 m, July 31, 1980, *J.M. Idrobo et al. 10315* (COL!) (Fig. 58).

*Other materials examined*: ECUADOR. **Chimborazo/Cañar**: Between Sta Rosa and Joyagshi, near Tipococha, 3,300–3,800 m, July 6–9, 1945, *W. Camp E-4065* (AMES!, UGDA-DLSz!—drawing). **Pichincha**: Canton Ruminiahui. Parroquia Amaguaña bosque protector Pasochoa, 0°27′S 78°28′W, 2,800–3,500 m, February 7, 1988, *C. Ceron & R. Alarcon 3530* (RPSC!, UGDA-DLSz!—drawing). VENEZUELA. **Mérida**: Sierra Nevada. La Laguna Coromoto. La Mucuy: valley of Río Loro, 3,100 m, *H. Barclay & P. Juajibioy 9933* (AMES!).

*Notes*: This species appears to be similar to *C. parvula* characterized above, but it has unifoliate stem (vs. 4–7-leaved), obtuse petals (vs. acute), and suborbicular (vs. elliptic-ovate) lip ornamented with five veins (vs. disc 3-veined).

***Cranichis queremalensis*** Szlach. & Kolan., Polish Bot. J. 58(2): 618–619, f. 2A–D. 2013. TYPE: Colombia. *Gonzalez et al. 168* (holotype, COL!, UGDA-DLSz!—drawing).

**Figure 63:**
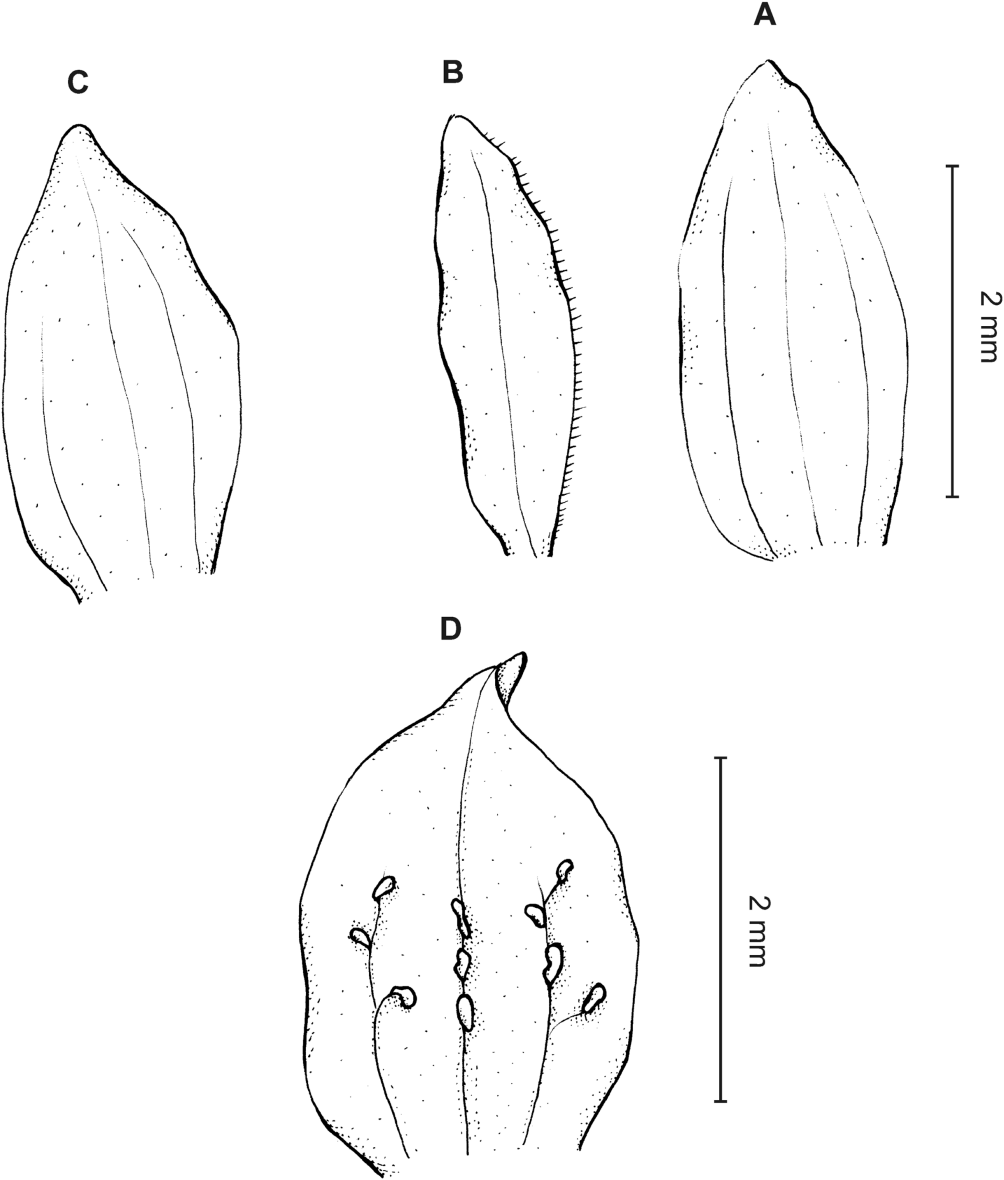
*Cranichis queremalensis* Szlach. & Kolan. (A) Dorsal sepal; (B) petal; (C) lateral sepal; (D) lip (front view). Drawn by S. Nowak from *Gonzalez et al. 168* (COL).

Plants up to 20 cm tall. Leaves 3, basal, petiolate; petiole up to 4.5 cm long, narrow; blade up to 4.5 cm long and two cm wide, elliptic-suborbicular, base subcordate, obtuse to subobtuse at apex. Scape erect, delicate, glabrous, enclosed distantly in four sheaths, of which the lowers are leafy. Inflorescence up to four cm long, conical, subdensely many-flowered. Flowers white. Floral bracts four mm long, lanceolate, acuminate, glabrous. Pedicellate ovary up to eight mm long, glabrous. Sepals glabrous. Dorsal sepal three mm long, 1.1 mm wide, oblong-elliptic-ovate, subobtuse, somewhat cochleate in the center, 3-veined. Petals three mm long, 0.6 mm wide, oblong-lanceolate, subacute to subobtuse, subfalcate, densely and shortly ciliate along the outer margin except base and apex, 1-veined. Lateral sepals three mm long, 1.2 mm wide, ovate to elliptic-ovate, subobtuse to subacute, somewhat oblique, slightly concave, 3-veined. Lip three mm long, two mm wide, almost flat, sessile, elliptic-suborbicular in outline, widest near the middle, apex with short, triangular, recurved, acute apiculus; disc with three obscure veins, with one or two branches and several knob-like swellings on veins. Gynostemium 1.1 mm long (Fig. 63).

*Ecology*: Terrestrial on the roadside in the area covered with premontane forest at the altitude of 1,430–1,550 m. Flowering occurs in December.

*Distribution*: Colombia.

*Representative specimen*: COLOMBIA. **Valle del Cauca**: Mpio. Dagua. Corregimiento El Queremal, 0–6 km carretera El Queremal-Buenaventura, 3°31.42–31.81′N, 76°43.62–44.92′W, Relictos bosque al lado de la carretera, 1,430–1,550 m, December 30, 2009, *M.F. Gonzalez et al. 168* (COL!, UGDA-DLSz!—drawing) (Fig. 58).

*Notes*: *C. queremalensis* appears to be related to its Colombian-Ecuadorian congener *C. parvula*, originally described from the Colombian department of Meta. *C. queremalensis* is characterized by petals attenuate toward base and apex with only outer margin shortly and densely ciliate, lip elliptic-suborbicular, very thin, and almost flat with recurved apex. Petals of *C. parvula* are oblong-ligulate, rounded at apex, glabrous on margins, lip is similar in shape to *C. queremalensis*, but cochleate in the center, with numerous knob-like projections, and straight apex.

***Cranichis schlimii*** Rchb.f., Linnaea 41: 19. 1877. TYPE: Venezuela. *Funck & Schlim 1238* (lectotype, designated by Garay (1978: 206) W!; isolectotype, G; AMES!—drawing).

**Figure 64:**
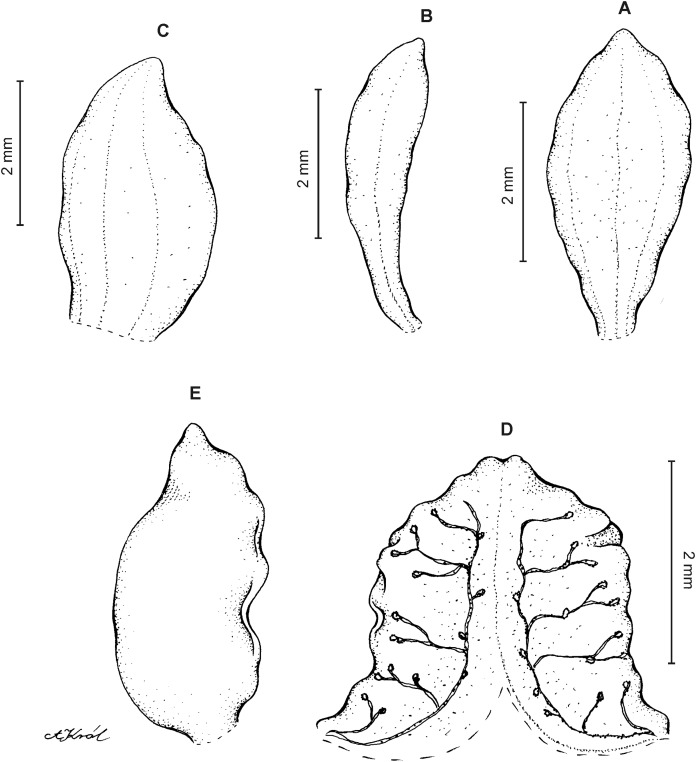
*Cranichis schlimii* Rchb.f. (A) Dorsal sepal; (B) petal; (C) lateral sepal; (D) lip (front view); (E) lip (side view). Drawn by A. Król from *Funck & Schlim 1238* (W-R).

**Figure 65:**
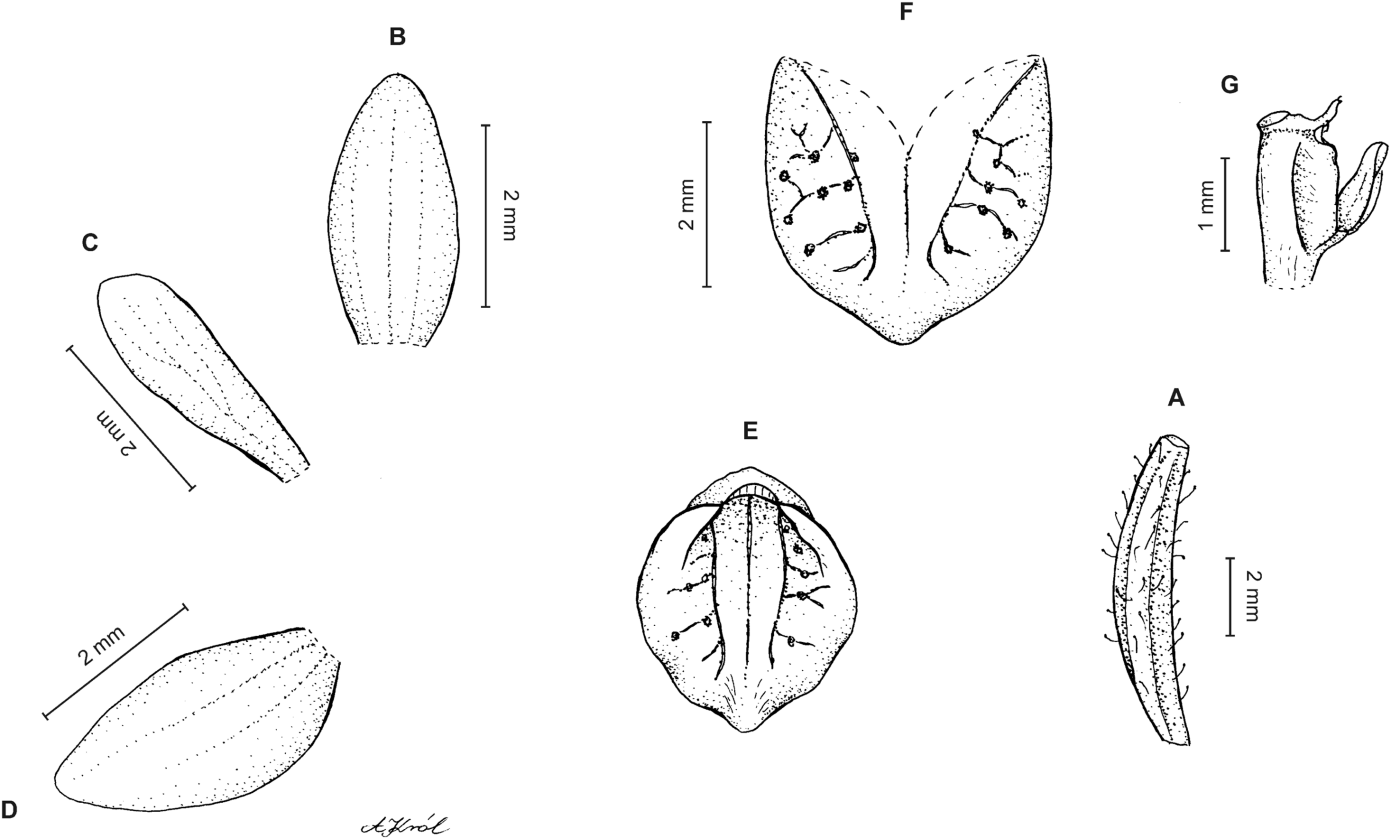
*Cranichis schlimii* Rchb.f. (A) Ovary; (B) dorsal sepal; (C) petal; (D) lateral sepal; (E–F) lip; (G) gynostemium. Redrawn by A. Król from Garay’s illustration of a specimen collected by *Barcley 9933* (AMES).

Plants 32 cm tall. Leaf single, basal, petiolate; petiole seven cm long; blade seven cm long, 3.5 cm wide, ovate, acute, base rounded. Scape remotely few-sheathed. Inflorescence 4.5 cm long, cylindric, subdensely many-flowered. Flowers small, sepals glandular outside. Floral bracts six mm long, lanceolate, glandular outside. Pedicellate ovary eight mm long, densely glandular. Dorsal sepal four mm long, 1.8 mm wide, oblong-obovate to obovate-oblanceolate, subobtuse, almost flat, 3-veined. Petals four mm long, one mm wide, linear-oblanceolate, falcate, subobtuse to subacute, 1-veined. Lateral sepals four mm long, two mm wide, oblong-elliptic to elliptic-ovate, oblique to somewhat sigmoid, acute to subobtuse, concave, 3- or 2-veined. Lip three mm long, 2.5 mm wide, deeply concave, sessile, subrectangular to oblong-elliptic in outline, obtuse to slightly notched at the apex; disc covered by irregular, small knob-like projections, veins three or five, thickened, dendritic branching. Gynostemium 1.5–2 mm (Figs. 64 and 65).

*Ecology*: Terrestrial in montane forest at an altitude of 2,800 m. Flowering occurs in May.

*Distribution*: Ecuador (Garay, 1978), Colombia, Venezuela.

*Representative specimen*: COLOMBIA. **Valle del Cauca**: Mpio. El Cerrito. I.P. Auji, hacienda La Pajosa, 2,800 m, May 22, 1990, *S. Sarria et al. 1422* (CUVC!) (Fig. 58).

*Other materials examined*: VENEZUELA. **Mérida**: 2,000 m, August 1846, *D. Funck & L.J. Schlim 1238* (W!).

*Notes*: Distinguished by the nonfoliaceous scape, bracts sparsely glandular-pubescent, green leaves, linear-oblanceolate, petals glabrous on the margins, ovary glandular-pubescent, lip obovate-cochleate, and disc with the same texture throughout.

This species could be related to *C. nigrescens*, from which it is easily distinguishable by lip shape (subrectangular to oblong-elliptic in outline, obtuse to slightly notched at the apex vs. ovate, widest at the base, subacute to subobtuse), and constantly 3-veined sepals (vs. 1-veined).

***Cranichis stictophylla*** Schltr., Repert. Spec. Nov. Regni Veg., Beih. 7: 62. 1920. TYPE: Colombia. *Madero s.n*. (B†).

**Figure 66:**
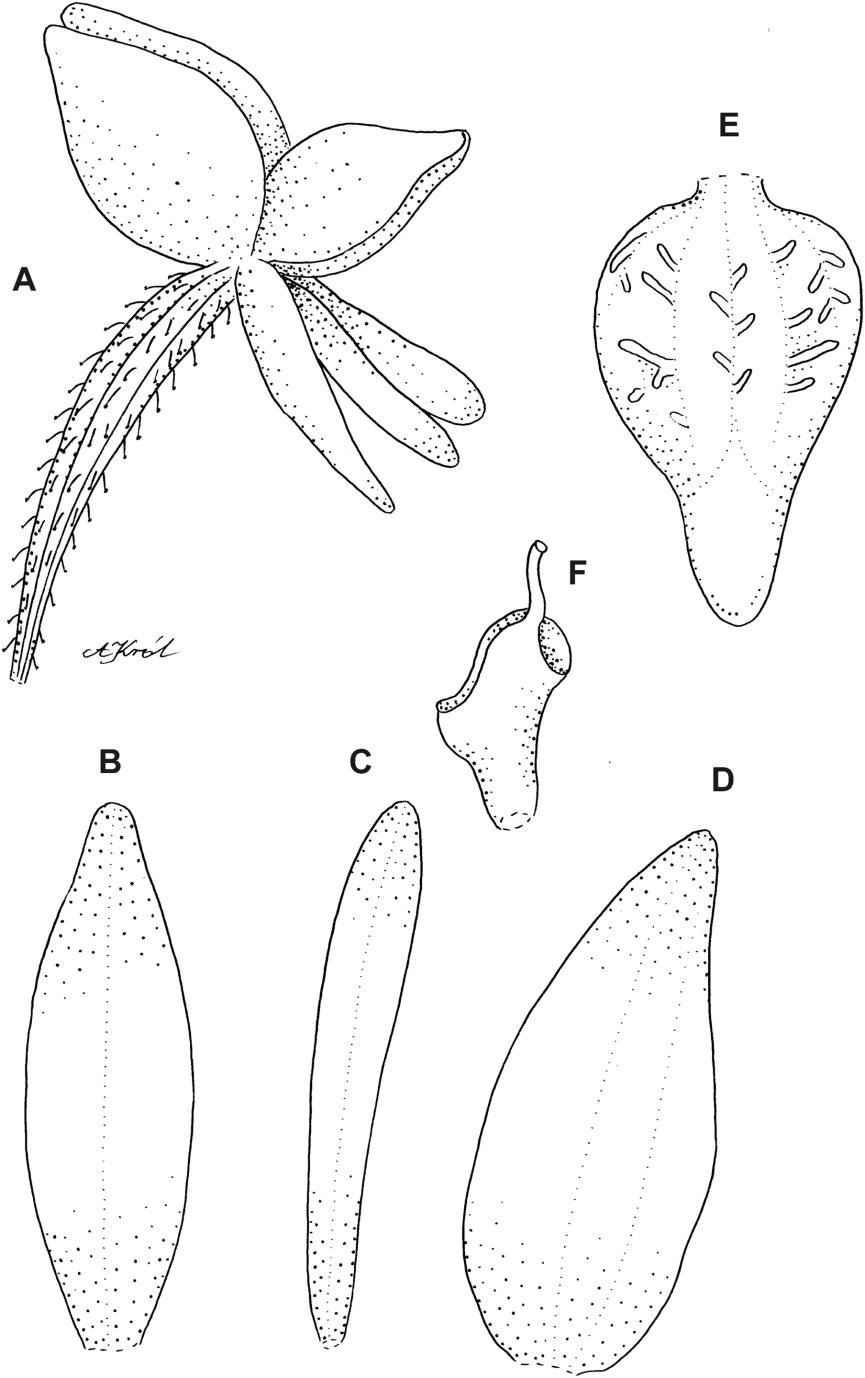
*Cranichis stictophylla* Schltr. (A) Flower; (B) dorsal sepal; (C) petal; (D) lateral sepal; (E) lip (front view); (F) gynostemium. Redrawn by A. Król according to the original illustration from Schlechter.

**Figure 67:**
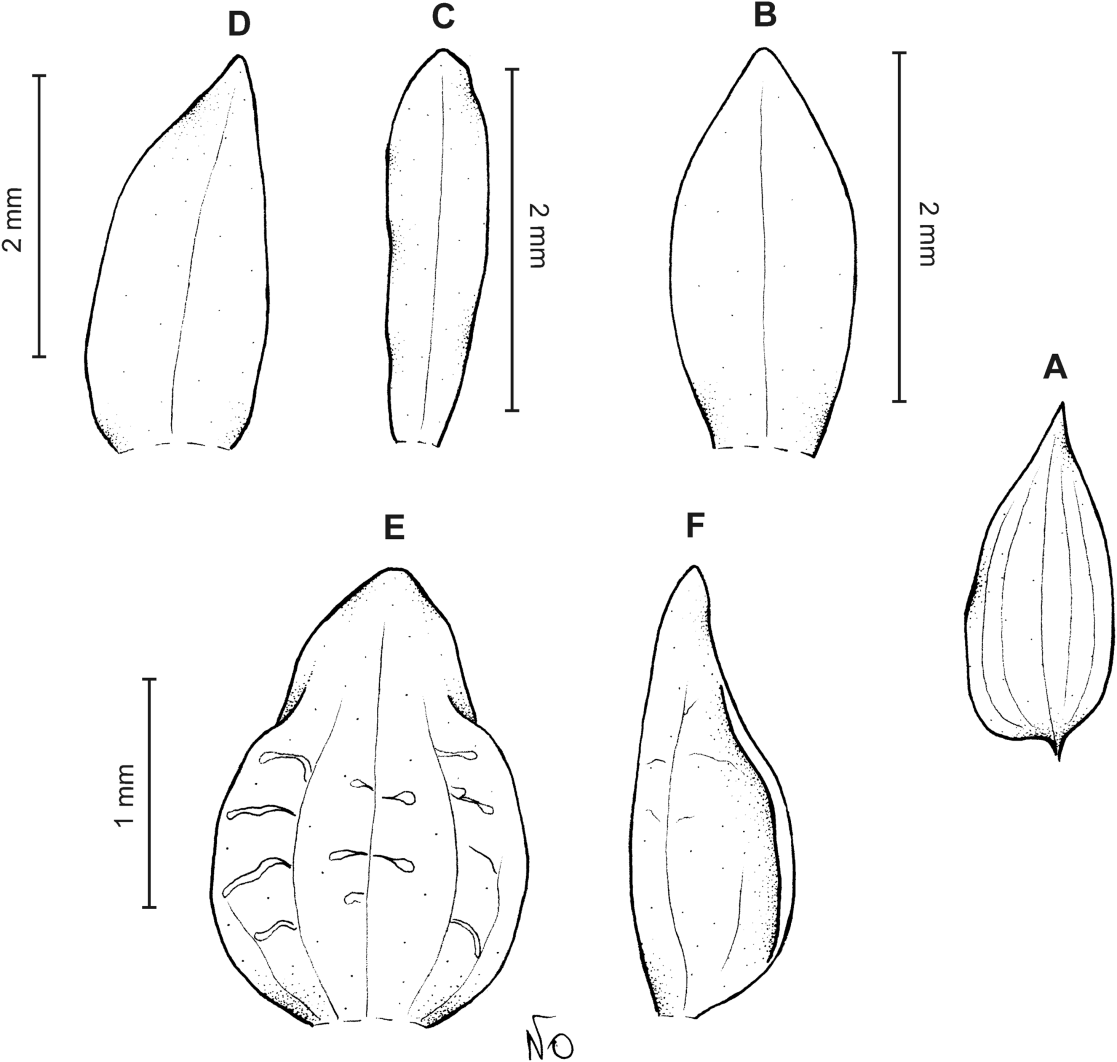
*Cranichis stictophylla* Schltr. (A) Leaf; (B) dorsal sepal; (C) petal; (D) lateral sepal; (E) lip (front view); (F) lip (side view). Drawn by N. Olędrzyńska from *Pennell 7597* (NY).

Plants 18–42 cm tall. Leaf 1, basal, petiolate; petiole 5–9.5 cm long; blade 3.5–8 cm long, 1.6–4.5 cm wide, elliptic-lanceolate to ovate, acuminate, base subrounded-cuneate, white or yellowish spotted. Scape glandulose-pilose toward the apex, enclosed in five to six sheaths. Inflorescence two to four cm long, cylindrical, sublaxly to subdensely 15-flowered. Flowers small, glabrous. Floral bracts three to six mm long, lanceolate, acuminate, sparsely glandular at the base. Pedicellate ovary six to seven mm long, densely glandular. Dorsal sepal 2.8–3.6 mm long, 1–1.2 mm wide, oblong elliptic, obtuse, 1-veined. Petals 2.3–3.1 mm long, 0.5–0.9 mm wide, obliquely linear-oblanceolate to linear-ligulate, obtuse, glabrous on margins, 1-veined. Lateral sepals 2.8–3.3 mm long, 1.2–1.6 mm wide, obliquely oblong-ovate to elliptic-ovate, slightly concave at the base, subacuminate, subobtuse, obscurely 2-veined. Lip two to three mm long, 1.5–1.9 mm wide, slightly concave, sessile, ovate to oblong ovate, obtuse; disc with 5, dendritic branching veins, branches thickened, with prominent nodules. Gynostemium 1.5 mm long (Figs. 66 and 67).

*Ecology*: Terrestrial in forests at an altitude of 2,000–2,700 m. Flowering occurs in July.

*Distribution*: Colombia, Peru.

*Representative specimens*: COLOMBIA. **Cauca**: *Sine loc*., 2,000 m, *M. Madero s.n*. (B†); San José. San Antonio. Cordillera Occidental, 2,400–2,700 m, July 1, 1922, *F.W. Pennell 7597* (AMES!, NY!, UGDA-DLSz!—drawing) (Fig. 58).

*Other materials examined*: PERU. **Amazonas**: Chachapoyas. Cerros Calla Calla, W side, 45 km above Balsas, 3,100 m, June 21, 1964, *P. Hutchison & J. Wright 5792* (AMES!, UGDA-DLSz!—drawing).

*Notes*: According to Schlechter (1920) this species is similar to *C. wageneri*, from which it differs in leaf shape and hair covering stem and ovary.

It appears to be very similar to *C. ovatilabia* from which it can be distinguished by their smooth sepals. Additionally, it has obscurely 2-veined lateral sepals, unlike *C. ovatilabia* and *C. monophylla*. Garay (1978) reduced this species to a synonym of *C. diphylla. C. stictophylla*, however, can be easily distinguished from the latter by the relatively long-petiolate leaves (5–9.5 cm vs. up to three cm), leaf base being subrounded-cuneate (vs. subcordate), 1-veined dorsal sepal (vs. 3-veined), and lip disc with five dendritic veins with prominent nodules (vs. three veins, often glandular).

Garay (1978) designated this as a lectotype of a *C. stictophylla* drawing made under Schlechter’s superivision of the *Madero 89* collection from Antioquia. Schlechter (1920), however, mentioned in the protologue *Madero s.n*.

### *Wageneri* group

Lip more or less oblong, elongate and curved down apically, concave below, with few branching veins. Petals with pubescent margins.

Three Colombian species belong to this group.

## Key to the Species

1. Lip base cuneate2
1* Lip base truncate or auriculate*C. schlechteri*
2. Lip apex hooked*C. viereckii*
2* Lip apex erect*C. wageneri*

***Cranichis schlechteri*** Szlach. & Kolan., Polish Bot. J. 58(2): 619. 2013. TYPE: Colombia. *Díaz P. et al. 745* (holotype, COL!, UGDA-DLSz!—drawing).

**Figure 68:**
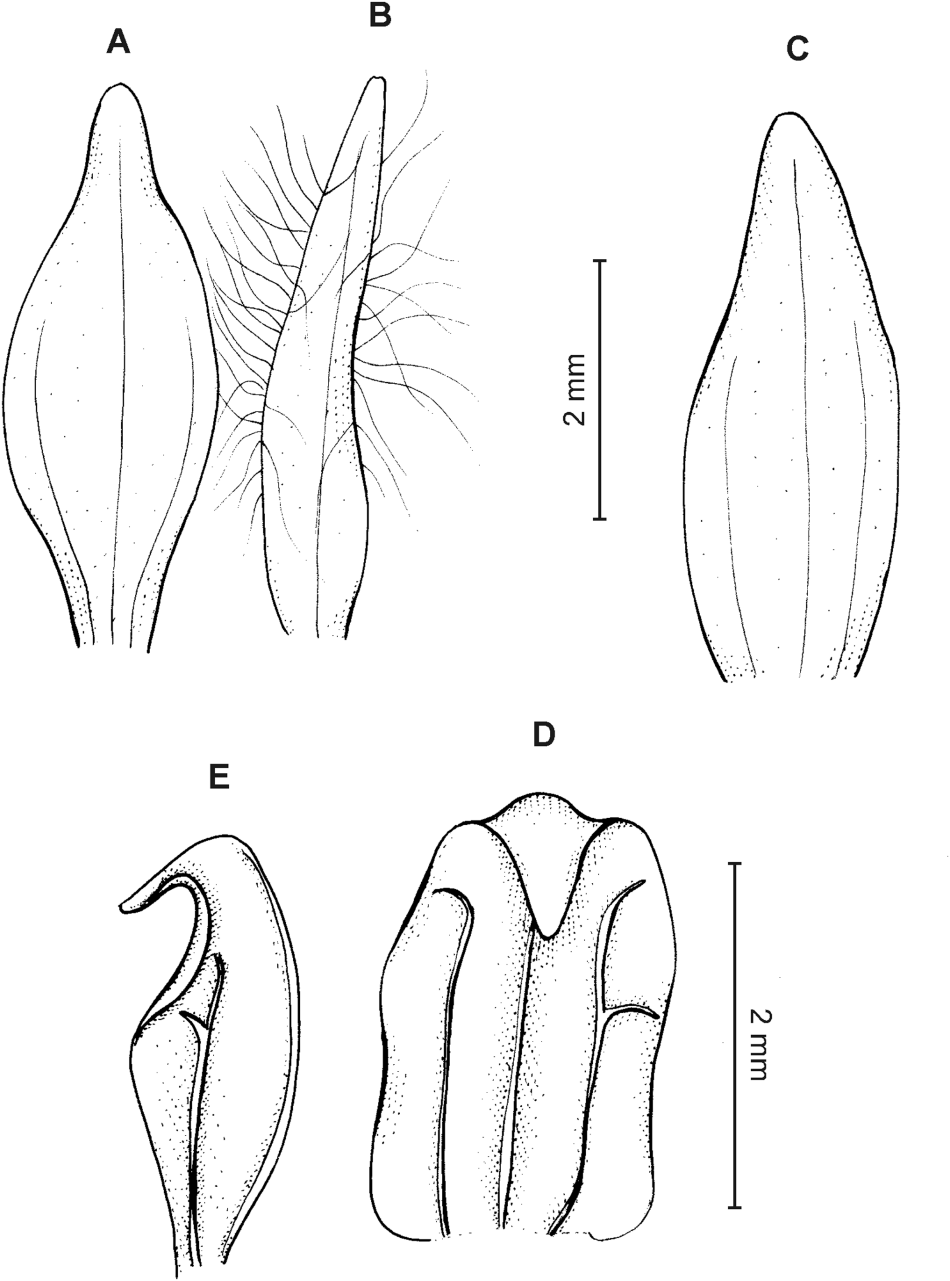
*Cranichis schlechteri* Szlach. & Kolan. (A) Dorsal sepal; (B) petal; (C) lateral sepal; (D) lip (front view); (E) lip (side view). Drawn by S. Nowak from the holotype Scale bar for (A–C) and for (D–E) = 2 mm.

Plants to 40 cm tall. Leaf single, basal, petiolate; petiole to 3.5 cm long; blade up to six cm long and 3.7 cm wide, ovate, acute, subcordate at base. Scape erect, delicate, glabrous in the lower half, otherwise glandular, enclosed in five sheaths. Inflorescence about three cm long, conical, subdensely many-flowered. Flowers small, inconspicuous, glabrous. Floral bracts five mm long, lanceolate, acuminate. Pedicellate ovary up to 10 mm long. Dorsal sepal 4.5 mm long, 1.5 mm wide, oblong-ovate above narrow base and attenuate toward obtuse apex, somewhat cochleate in the center, obscurely 3-veined. Petals four mm long, 0.6 mm wide, oblong-lanceolate to linear-lanceolate, acute at apex, subfalcate, margins covered by long and soft ciliae except base and apex, 1-veined. Lateral sepals 4.5 mm long, 1.8 mm wide, ovate to elliptic-ovate, subobtuse, somewhat oblique, slightly concave, obscurely 3-veined. Lip concave, sessile, 2.5 mm long, 1.6 mm wide, oblong-ovate in outline, basally auriculated, apex elongate, subobtuse, incurved; veins 3, protruding, sparsely branching. Gynostemium 1.5 mm long (Fig. 68).

*Ecology*: Terrestrial plant growing at an altitude of 1,200–1,300 m. Flowering occurs in July. This species is most probably autogamous.

*Distribution*: Colombia.

**Figure 69:**
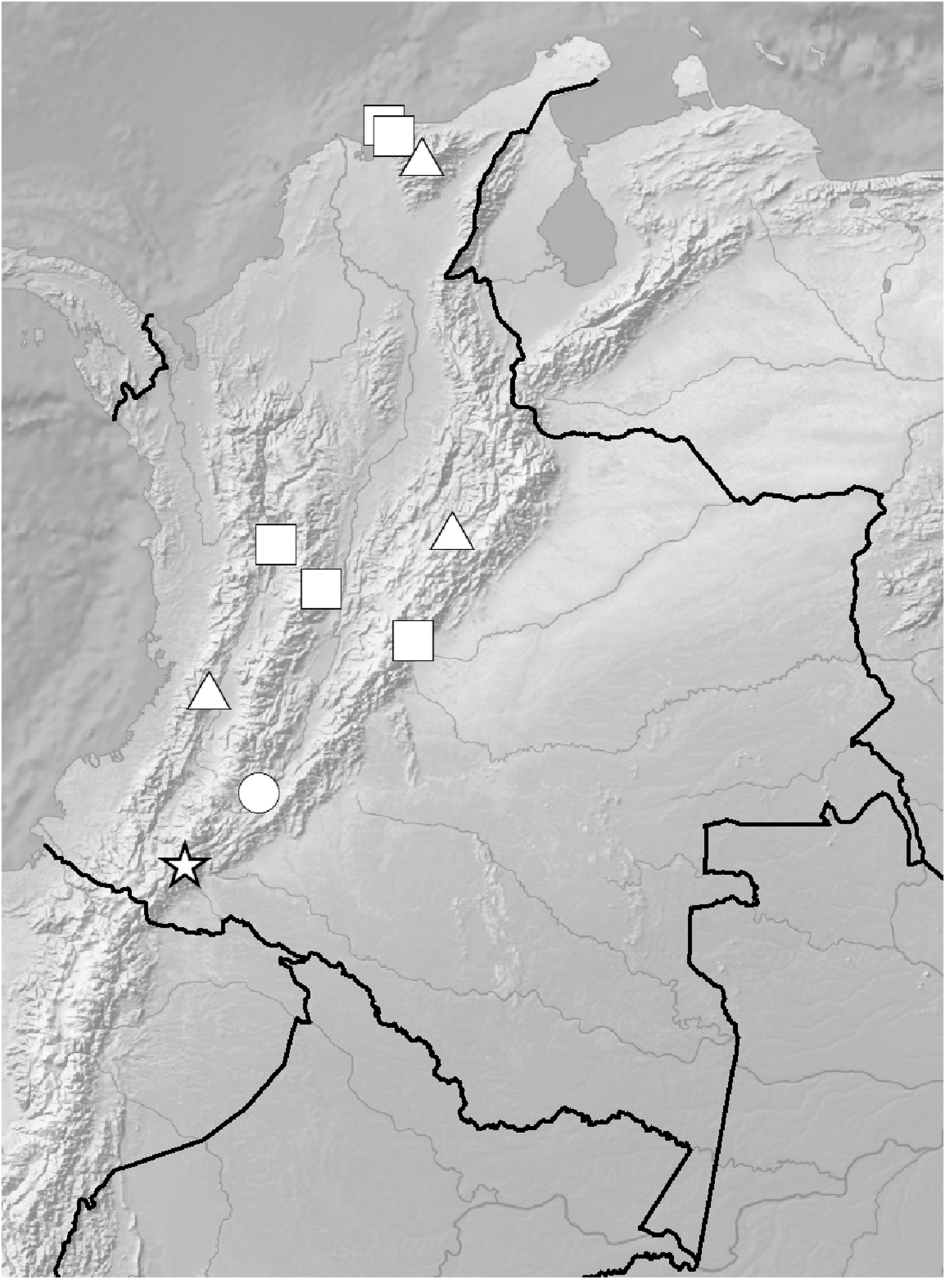
Distribution of members of the *Cranichis Wageneri* group and *Pulvinifera* group. *Cranichis schlechteri* (circle), *C. viereckii* (square), *C. wageneri* (triangle), *C. pulvinifera* (star).

*Notes*: This species appears to be similar to its Ecuadorian congener *C. macroblepharis* Rchb.f., but is easily distinguishable from the latter by having an auriculate base of the lip and long ciliate petal margins.

*Representative specimen*: COLOMBIA. **Huila**: Mpio. La Plata. Vereda Agua Bonita. Finca Merenburg, 1,200–1,300 m, July 20, 1975, *S. Díaz P. et al. 745* (COL!, UGDA-DLSz!—drawing) (Fig. 69).

***Cranichis viereckii*** Ames, Sched. Orch. 7: 1. 1924. TYPE (here designated): Colombia. *Viereck s.n*. (lectotype, AMES!; isolectotype, US!, UGDA-DLSz!—drawing).

**Figure 70:**
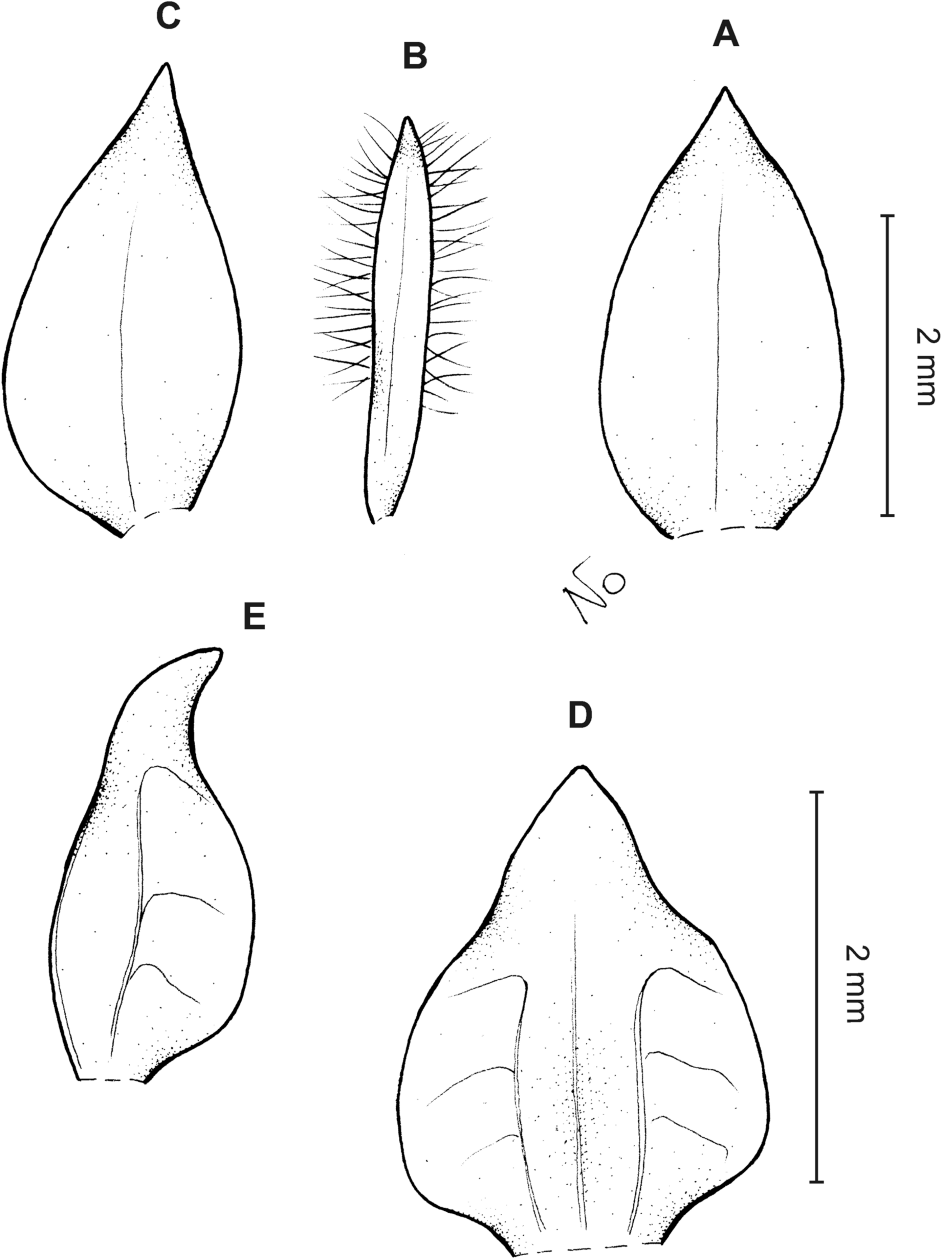
*Cranichis viereckii* Ames. (A) Dorsal sepal; (B) petal; (C) lateral sepal; (D) lip (front view); (E) lip (side view). Scale bar for (A–C) = 2 mm. Drawn by N. Olędrzyńska from *Viereck s.n*. (US).

**Figure 71:**
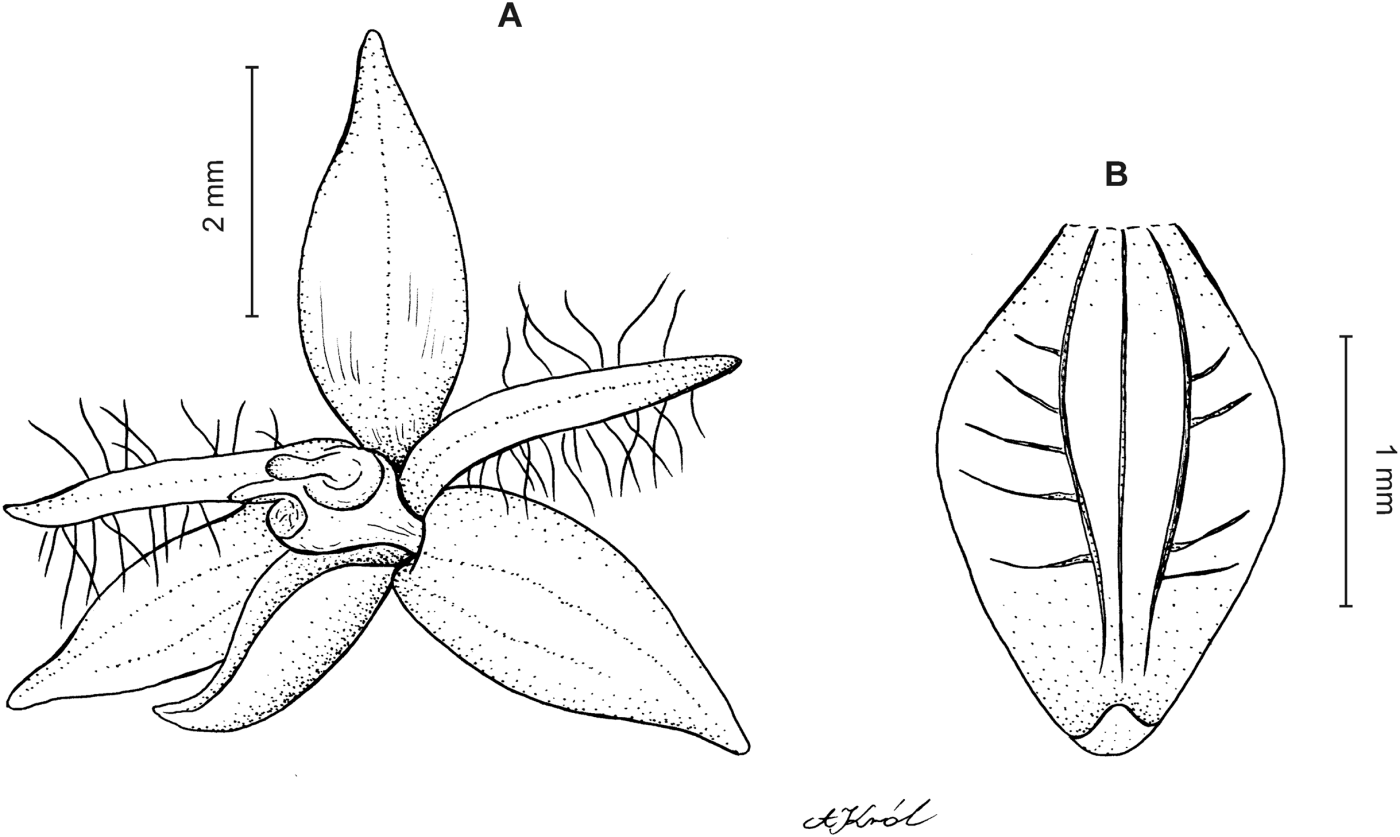
*Cranichis viereckii* Ames. (A) Flower; (B) lip. Redrawn by A. Król from Garay’s illustration of a specimen collected by *Viereck s.n. (*AMES).

**Figure 72:**
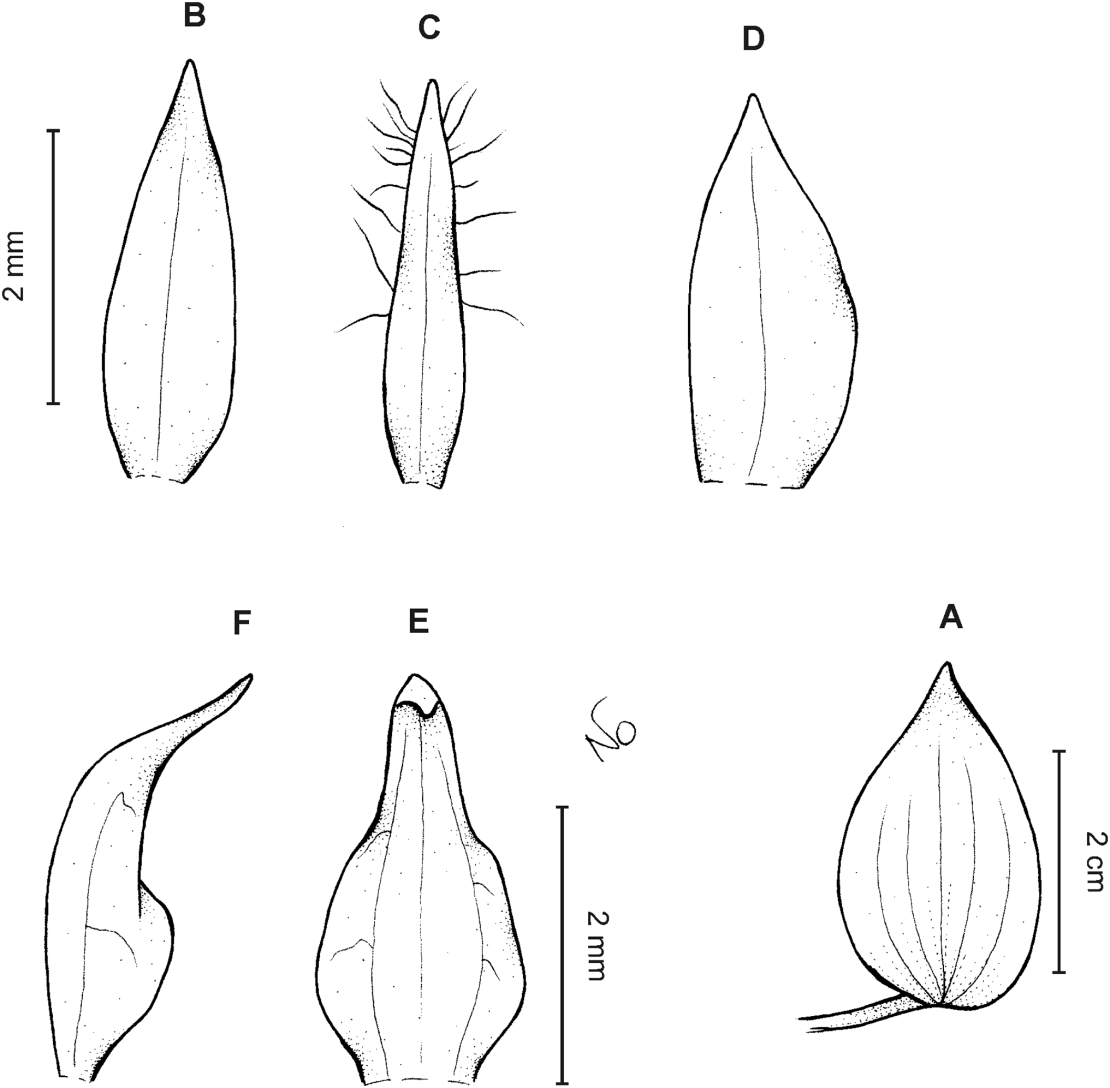
*Cranichis viereckii* Ames. (A) Leaf; (B) dorsal sepal; (C) petal; (D) lateral sepal; (E) lip (front view); (F) lip (side view). Drawn by N. Olędrzyńska from *Pennell 3300* (NY). Scale bar for (B–D) and (E–F) = 2 mm.

Plant 13–30 cm tall. Leaves 1–2, basal, very dissimilar in size, petiolate; petiole one to six cm long; smaller blade 1.5–4 cm long, 0.7–1.6 cm wide, narrowly ovate-lanceolate, acuminate, acute; larger blade 6.5–8 cm long, 3.5–4.5 cm wide, ovate or elliptical, acuminate, acute, membranaceous, reticulate-veined. Scape including the raceme up to 28.5 cm long, slender or rather stout, shortly glandular-pubescent above, enclosed in about six closely appressed acute tubular sheaths. Inflorescence 1.5–5 cm long, conical, rather laxly many-flowered, glabrous. Floral bracts 5–7.5 mm long, lanceolate, acute, glabrous. Pedicellate ovary 7–10 mm long, glabrous. Dorsal sepal 3.1–4.2 mm long, 0.9–1.75 mm wide, elliptic- or ovate-lanceolate, acute, 1- or occasionally 3-veined. Petals 2.6–4 mm long, 0.3–0.8 mm wide, linear-oblong, obtuse, somewhat curved at the base, mid-vein conspicuous, margin provided with numerous elongated flattish whitish hairs which are often 1.5–2 mm long. Lateral sepals 3–4.5 mm long, 1.1–1.5 mm wide, elliptic-lanceolate, acute, conspicuously 1-veined through the middle, with an indistinct shorter vein on each side, glabrous. Lip 2.5–3 mm long, 1.1–2.1 mm wide, concave, minutely unguiculate, broadly ovate, narrowed above the middle into a subacute hooked apex; disc conspicuously 3-veined, with each lateral vein giving off at right angles four to five supplementary veins. Gynostemium one to two mm long (Figs. 70–72).

*Ecology*: Terrestrial in montane and cloud forests, epiphytic in wet forest. Growing at altitudes of 1,200–2,420 m. Flowering occurs in October and December.

*Distribution*: Ecuador, Colombia.

*Representative specimens*: COLOMBIA. **Antioquia**: Mpio. Caramanta. Vereda Hojas Anchas. Cerro Viringo, 9.8 km de Caramanta hacia Supia, Cordillera Occidental, bosque primario poco intervenido y nublado, 5°31.8′N 75°40.69′W, 2,140–2,420 m, October 16, 1988, *J. Betancur et al. 1096* (MO!, UGDA-DLSz!—drawing). **Cundinamarca**: Guayabetal, 1,550 m, *M. Schneider 418* (AMES!). **Magdalena**: Santa Marta. Forest near Manzanaces, 1,200 m, December 8, 1898, *H.H. Smith 2845* (NY!); Vista Nieve. Santa María, December 1922, *L. Viereck s.n*. (AMES!, US!, UGDA-DLSz!—drawing). **Tolima**: La Virginia. Libano, 1,200–1,500 m, December 22, 1917, *F.W. Pennell 3300* (NY!, UGDA-DLSz!—drawing) (Fig. 69).

*Other materials examined*: ECUADOR. **Carchi**: Vicinity of Maldonado, 1,600 m, April 13, 1977, *M.T. Madison 3847* (RPSC!, UGDA-DLSz!—drawing).

*Notes*: *C. viereckii* is similar to *C. schlechteri*, from which it differs by cuneate lip base (vs. auriculate), with lateral veins giving off at right angles four to five supplementary veins (vs. sparsely branching) and 1-veined sepals (vs. obscurely 3-veined).

***Cranichis wageneri*** Rchb.f., Linnaea 41: 19. 1877. TYPE (here designated): Venezuela. *Wagener s.n*. (lectotype, W!; isolectotype, AMES!; AMES!—drawing, UGDA-DLSz!—drawing).

**Figure 73:**
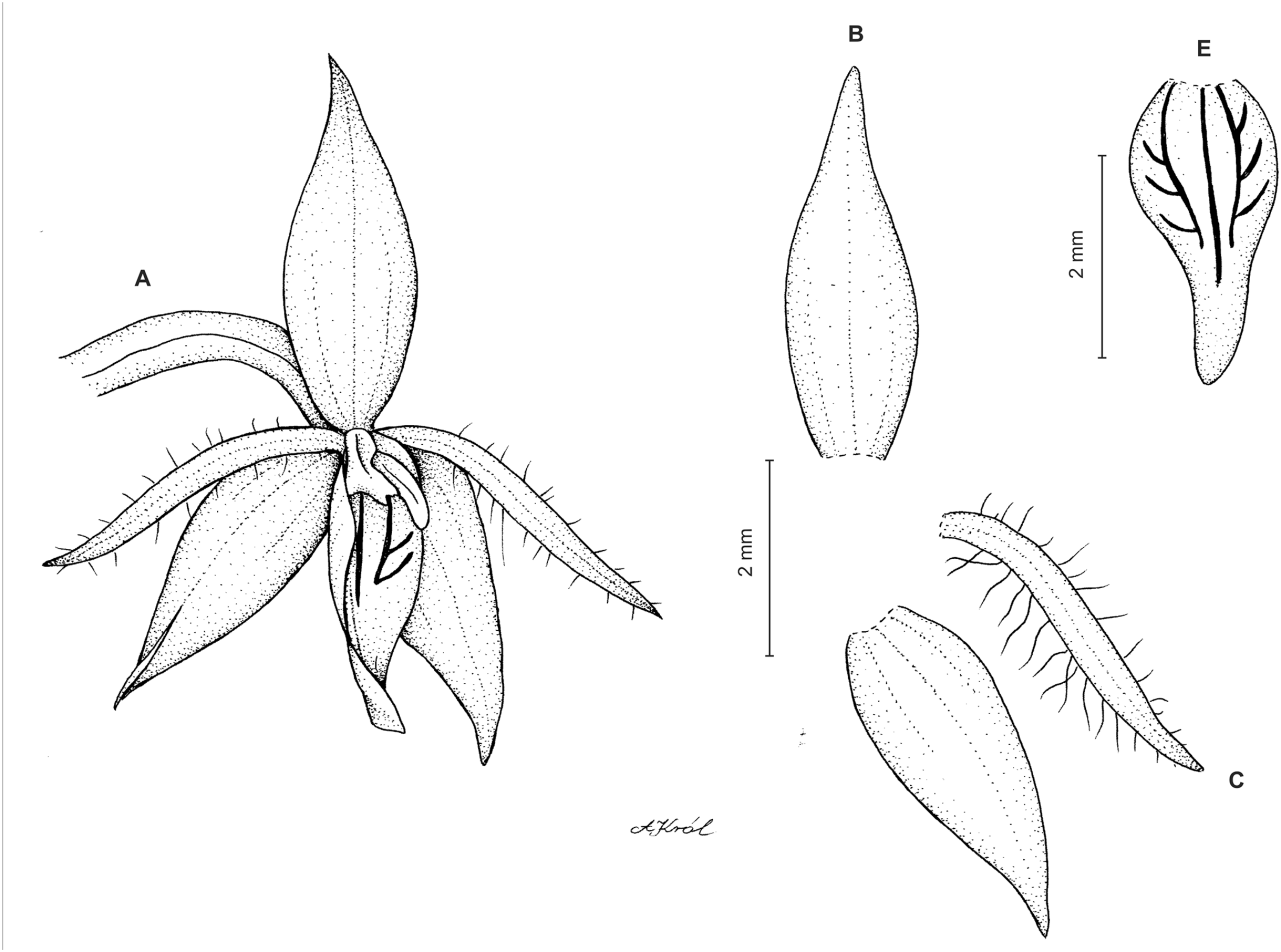
*Cranichis wageneri* Rchb.f. (A) Flower; (B) dorsal sepal; (C) petal; (D) lateral sepal; (E) lip (front view). Redrawn by A. Król from Garay’s illustration of specimen collected by *Wagener s.n. (*AMES).

Plants 20–30 cm tall. Leaves 1–2, basal, petiolate; petiole up to two cm long; blade up to 7.5 cm long and 3.5 cm wide, ovate, acute, base rounded. Scape enclosed in about five to eight tubular sheaths. Inflorescence up to eight cm long, conical to subglobose, laxly few- to many-flowered. Flowers small, glabrous. Floral bracts five mm long, lanceolate, acute. Pedicellate ovary eight mm long. Dorsal sepal 4–4.5 mm long, 1.1–2 mm wide, lanceolate to oblong-lanceolate, acute to acuminate, 1- or 3-veined. Petals 3.5–4 mm long, 0.3–0.4 mm wide, linear-lanceolate, somewhat falcate, acute, with irregular, long hairs along margins, 1-veined. Lateral sepals 4–4.2 mm long, 1.5 mm wide, obliquely lanceolate-ovate to oblong-ovate, acute to acuminate, somewhat concave, 1- or 3-veined. Lip 2.8–3 mm long, 1.6–2 mm wide, almost flat, subsessile, ovate-sagittate in outline, acute; disc with 3, thick and branching veins, each lateral vein giving off at right angles four to five supplementary veins. Gynostemium 1.5 mm long (Fig. 73).

*Ecology*: Terrestrial in humid montane forest at altitudes of 2,050–2,810 m. Flowering occurs in April, October, and December.

*Distribution*: Mexico (Hágsater et al., 2005), Guatemala (Ames & Correll, 1952), El Salvador (Hamer, 1974), Honduras (Ames & Correll, 1985), Nicaragua (Hamer, 1982), Costa Rica (Dressler, 2003), Peru (Schweinfurth, 1958), Ecuador?, Colombia, Venezuela (Foldats, 1969).

*Representative specimens*: COLOMBIA. **Boyacá**: Mpio. de Sotaquira. Monte Vargas. 5°43′53.3″N, 73°17′05.0″W, 2,810 m, *M.P. Cordoba et al. 3122* (COL!); Mpio. de Sotaquira, 5°43′55.9″N, 73°10′30.8″W, 2,620 m, December 13, 2003, *M.P. Cordoba et al. 3143* (COL!). **Magdalena**: Sierra Nevada de Santa Marta, SE slopes. Hoya del Río Donachui, Cancurua, fields and forest, 2,400–2,650 m, October 10–11, 1959, *J. Cuatrecasas & R. Romero Castaneda 24743* (COL!, US!). **Valle del Cauca**: Mpio. Yumbo. Dapa, Jorge Negret farm, 35 min from el Rodadero station, 3°32′54.9″N 76°35′15.4″W, 2,074 m, April 2012, *E. Parra s.n*. (VALLE!) (Fig. 69).

*Other materials examined*: VENEZUELA. Caracas. *M. Wagener s.n*. (AMES!, W!, AMES!—drawing, UGDA-DLSz!—drawing).

*Notes*: This is the only species of the group with erect, not hooked apex of the lip.

### *Pulvinifera* group

Lip suborbicular in outline, basally concave, more or less 3-lobed at the apex, laterally thickened, lateral veins branching with numerous globose projections. Petals with glabrous margins.

***Cranichis pulvinifera*** Garay, Fl. Ecuador 9: 204, fig. 61 A. 1978. TYPE: Colombia. *Bristol 1227* (holotype, AMES!).

**Figure 74:**
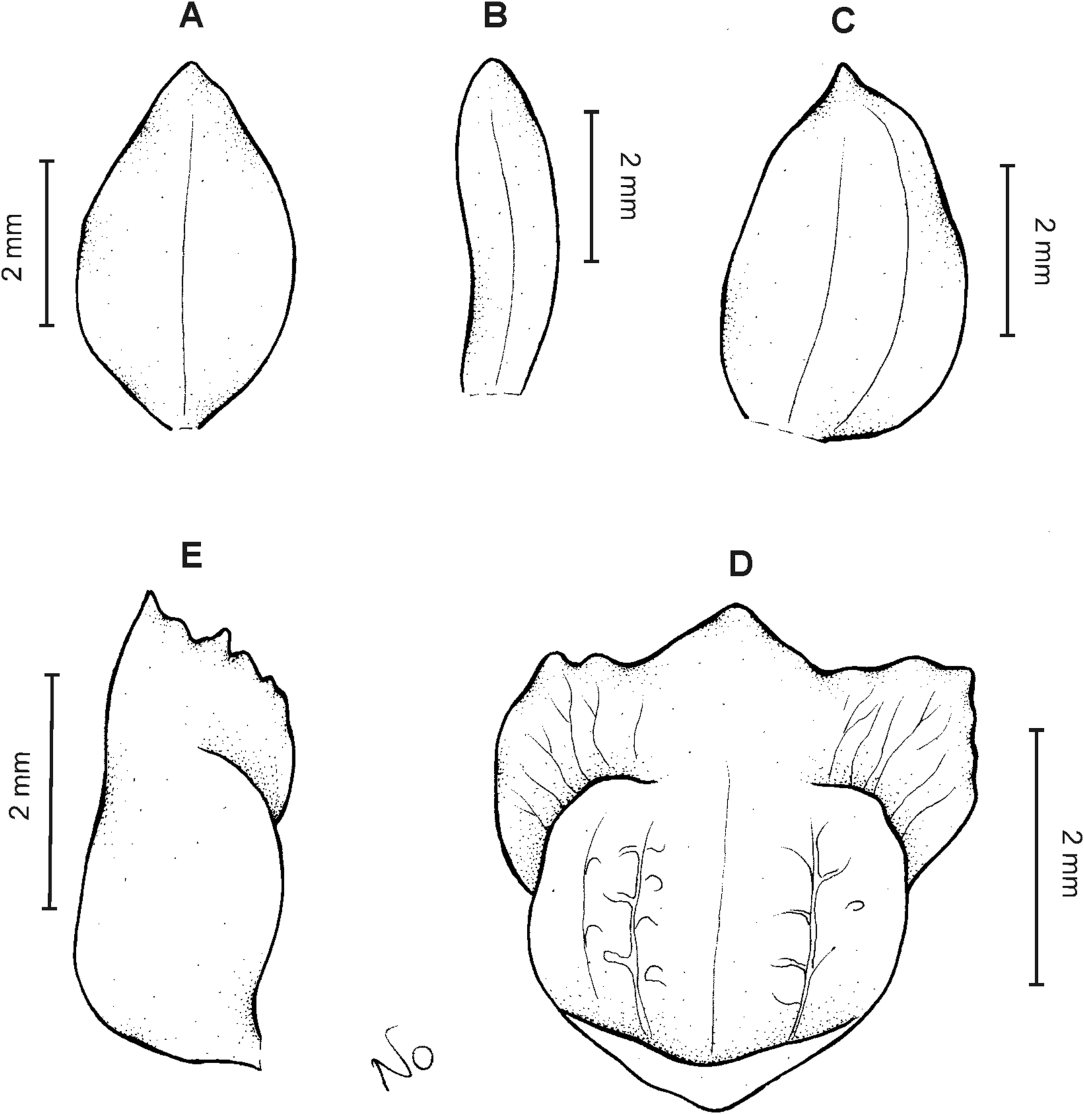
*Cranichis pulvinifera* Garay; (A) Dorsal sepal; (B) petal; (C) lateral sepal; (D) lip (front view); (E) lip (side view). Drawn by N. Olędrzyńska from *Bristol 1227* (AMES).

**Figure 75:**
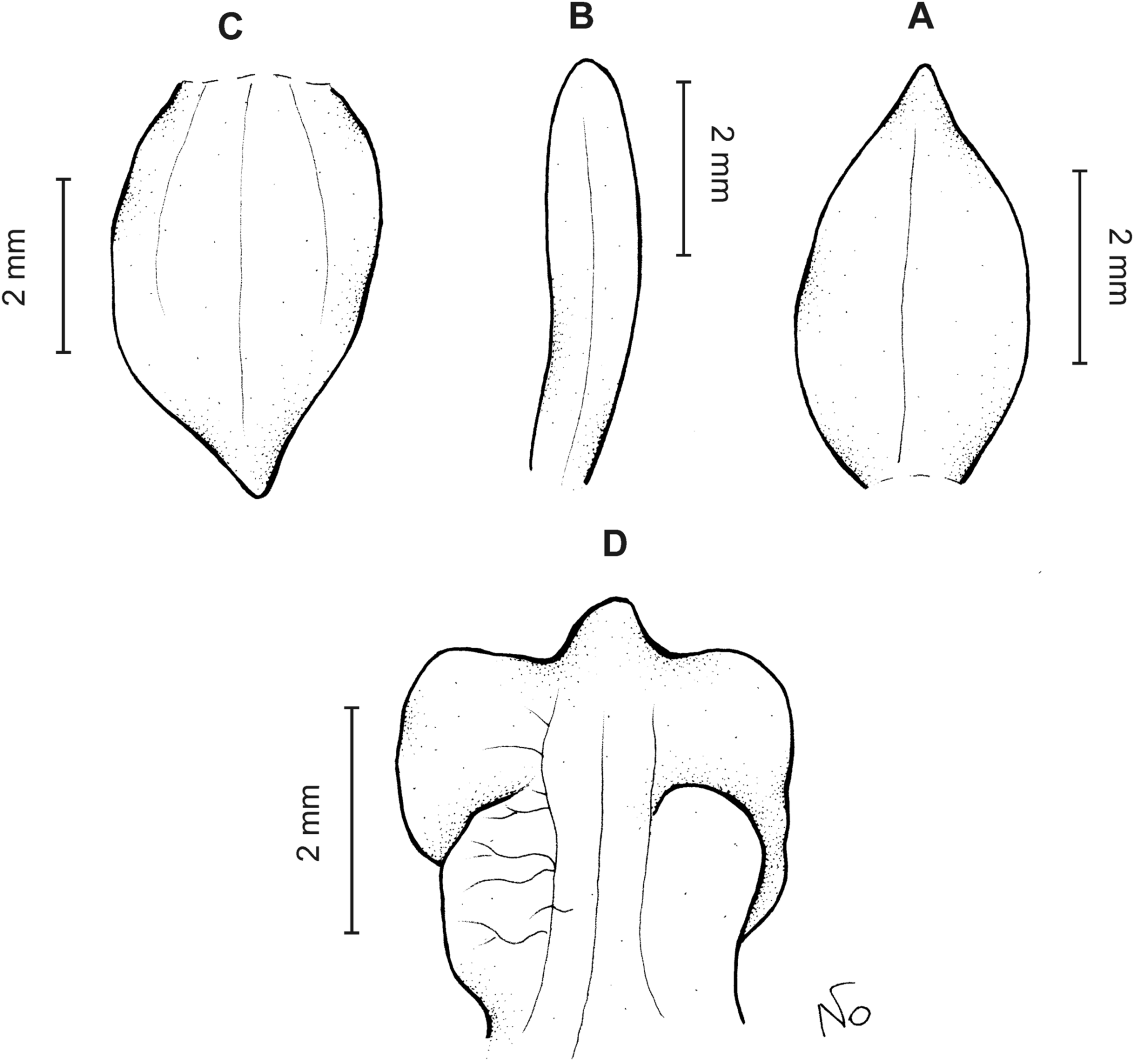
*Cranichis pulvinifera* Garay. (A) Dorsal sepal; (B) petal; (C) lateral sepal; (D) lip (front view). Drawn by N. Olędrzyńska from *Luer et al. 498* (RPSC).

Plants up to 32 cm tall. Leaves several, basal, petiolate; petiole up to nine cm long, narrow; blade up to eight cm long and four cm wide, ovate, acute, with rounded base. Scape erect, slender, few-sheathed, the lowermost foliaceous. Inflorescence seven to nine cm long, cylindrical, loosely few-flowered. Flowers glabrous, greenish. Floral bracts up to eight mm long, lanceolate, acuminate, glabrous. Pedicellate ovary 9–11 mm long, glabrous. Dorsal sepal up to five mm long and 2.3 mm wide, narrowly elliptic, obtuse, l-veined. Petals up to 4.5 mm long and 1.3 mm wide, linear-ligulate, obtuse, l-veined. Lateral sepals up to 5.3 mm long and three mm wide, obliquely ovate-elliptic, obtuse, obscurely 2 or rarely 3-veined. Lip up to four mm long and wide, navicular or subsaccate, sessile, triangular-obovate, truncately 3-lobed in front with the middle lobe itself being triangular, obtuse; disc ballooned out like inflated cushions which are sparsely covered with large papillae. Gynostemium 2.3–3 mm long (Figs. 74 and 75).

*Ecology*: Terrestrial in montane forest at an altitude of about 2,200 m. Flowering occurs in July.

*Distribution*: Ecuador, Colombia.

*Representative specimen*: COLOMBIA. **Putumayo**: Valle de Sibundoy. 2.5 km SE Colón. Alt. 2,200 m. July 16, 1963. *M. Bristol 1227* (AMES!, UGDA-DLSz!—drawing) (Fig. 69).

*Other materials examined*: ECUADOR. **Loja**: San Pedro de Vilcabamba entering from along the road to Loja and walking 2 h up the path, 2,400 m, April 23, 1986, *D. Dalessandro 623* (RPSC!, UGDA-DLSz!—drawing). **Tungurahua**: Valle de Cahupe above Baños, 2,200 m, July 25, 1975, *C. Luer et al. 498* (RPSC!—para, UGDA-DLSz!—drawing).

*Notes*: Distinguished by the foliaceous scape, the glabrous ovary, the 1-veined dorsal sepal, the triangular-obovate lip with a membranaceous margin, the disc of the lip 3-veined, papillose-verrucose, the 1-veined, linear-oblong petals with entire margins, the short, broadly cylindric, loosely few-flowered inflorescence, the ovate lip, and the disc from base to middle cushion-like ballooned out.

### Unconfirmed species

***Cranichis picta*** Rchb.f., Linnaea 41: 52. 1877. TYPE (Garay, 1978: 203): Ecuador. *Jameson s.n*. (W!; lectotype, designated by Garay (1978: 203) AMES!—drawing, RENZ—drawing).

Plants up to 35 cm long, erect, rather delicate. Leaves basal, usually 1, rarely 2–3, or deciduous at flowering, up to 20 cm long including the petiole and 3.5 cm wide, elliptic, lanceolate-elliptic, oblanceolate to narrowly oblanceolate, acute to acuminate, gradually tapering into a distinct petiole. Scape 10 cm long, glabrous, remotely few-sheathed. Inflorescence about eight cm long, cylindric, densely many-flowered. Flowers white, lip white with brown veins, fleshy. Floral bracts three to seven mm long, ovate-lanceolate, acute to acuminate, oblique. Pedicellate ovary 6–11 mm long. Dorsal sepal five mm long, 2.5–3 mm wide, elliptic-ovate, concave, obtuse, acute to shortly acuminate, 3-veined. Petals 3.5–5 mm long, 1.3–1.6 mm wide, obliquely oblong-oblanceolate, acute to acuminate, margins erose, 3-veined. Lateral sepals 4–5.5 mm long, 2.5–3.5 mm wide, obliquely elliptic-ovate to suborbicular-ovate, acute to shortly acuminate, principally 3-veined. Lip very fleshy with thin margins, cochleate, sessile, three to five mm long and wide, subglobose, margins recurved, irregularly dentate; disc 3-veined, lateral veines with several branches. Gynostemium two mm long.

*Ecology*: Terrestrial in high-montane forest.

*Distribution*: Ecuador, Colombia (Garay, 1978).

*Specimen examined*: ECUADOR. Pichincha. *W. Jameson s.n*. (W!).

*Notes*: Distinguished by the foliaceous scape, ovary glabrous, lip with a distinctly membranaceous margin, disc 5-veined, not verrucose, petals more or less erose-undulate, 1–3-veined, often branching. Due to the discrepancy between the illustration of this species deposited in RENZ and the description provided by Garay (1978), the actual morphological characteristics of this species require further studies.

It can be confused with *C. brevirostris*, but unlike the latter it has petals with erose margins and non-crenate, flat leaf margins.

***Cranichis tenuiflora*** Griseb., Cat. Pl. Cub.: 268. 1866. TYPE: Cuba. *Wright 3292* (AMES!, P!; AMES!—drawing).

Plants up to 30 cm tall. Leaves 2, basal, petiolate; petiole up to about 5.6 cm long; blade up to 5.6 cm long, 3.1 cm wide, ovate-elliptic to ovate-suborbicular, subcordate, acuminate. Scape slender, enclosed in few lanceolate, acute sheaths. Inflorescence up to four cm long, subdensely several-flowered. Flowers yellow, lip with purple spots. Floral bracts lanceolate, acute, about 2.5 mm long, about half shorter than the ovary. Pedicellate ovary subsessile, glabrous, about six mm long. Sepals similar, glabrous, up to 3.5 mm long, 2.5 mm wide, broadly ovate, obtuse; lateral sepals somewhat oblique. Petals oblong-elliptic, 3.5 mm long, 1.2 mm wide. Lip membranaceous, concave-cucullate, sessile, about 2.6 mm long, less than one mm wide, elliptic to suborbicular, obtuse, shortly apiculate; disc 3-veined, lateral veins branching. Gynostemium about one mm long.

*Distribution*: Cuba.

*Notes*: The occurrence of this species in Colombia was reported by Dueñas Gómez & Fernández Alonso (2009). The authors’ reference material was a collection from Antioquia (*Ortiz 655*, HPUJ), but we did not have access to this specimen.

This species is often placed on the list of synonyms of *C. diphylla*, but in our opinion both taxa deserve to be treated separately. The flowers of *C. tenuiflora* are yellow, with lip adorned with purple spots (vs. flowers white with green veins), ovary is glabrous (vs. more or less glandular), lip is over twice as long as wide (vs. ca. as long as wide), with broadly cuneate base (vs. subcordate base) and thin (vs. papillose-thickened center).

***Cranichis tenuis*** Rchb.f., Flora 48: 274. 1865. TYPE (here designated): Cuba. *Wright 1478* (lectotype, W!; isolectotypes, AMES!, P!).

Plants 8–35 cm tall. Leaves several, basal, petiolate; petiole 0.4 cm long; blade 0.9–6 cm long, 0.6–2 cm wide, elliptic to orbicular, base cuneate to acute, apex rounded to acute. Scape slender with a few distant scarious, sheathing bracts, glandular pubescent above. Inflorescence 2–12 cm long, conical, rather laxly many-flowered. Flowers white. Floral bracts seven mm long, obovate, acute. Pedicellate ovary 8–14 mm long, pubescent. Perianth glabrous. Dorsal sepal 2–3.5 mm long, 2–2.5 mm wide, broadly elliptic to broadly obovate, rounded-obtuse. Lateral sepals 3.5 mm long, 2.5 mm wide, ovate-elliptic to broadly ovate, obtuse to subacute. Petals 3.5 mm long, 1.3–1.5 mm wide, entire, oblong elliptic to oblong oblanceolate, blunt. Lip concave, subsessile, 2–2.5 mm long, 1.5 mm wide, rectangular-elliptic in outline, rounded at the apex, fleshy, deeply concave, laterally compressed, hoodlike and surrounding the column.

*Ecology*: Terrestrial in serpentine soils of moist scrub forest at middle to high elevations, restricted, but locally common.

*Distribution*: The Greater Antilles (Cuba, Hispaniola, Puerto Rico).

*Materials examined*: CUBA. *Sine loc., C. Wright 1478* (AMES!, P!, W!).

*Notes*: The occurrence of this species in Colombia was reported by Ortiz Valdivieso & Uribe Vélez (2007) without providing a reference specimen.

## Taxonomic Transfers

The Neotropical genus *Ocampoa* A. Rich. & Galeotti was described by Richard and Galeotti in the mid-19th century (Richard & Galeotti, 1845) based on a plant collected in Mexico. The morphology of flower segments is a discriminative character which allows this genus to be easily discerned from *Cranichis*. The lip of *Ocampoa* is free from the gynostemium, its base is unguiculate, more or less concave to even subsaccate as caused by a C or S curvation of the claw. The lip of *Cranichis* is usually sessile or subsessile, flat or basally gibbose, but never C or S curved. Lateral sepals of the former are more or less ovate-triangular, acute and oblique, and usually oblong ovate to ovate-ellitpic, subobtuse in *Cranichis* (cf. Kolanowska & Szlachetko, 2015; Szlachetko & Kolanowska, 2017b). *Ocampoa* species are characterized by the presence of some cauline leaves, which are linear to lanceolate, and the general habit of these species reminds of *Habenaria*. All species of *Cranichis* have basal, narrowly petiolate leaves with wide blade.

Until 2013, only one species of *Ocampoa* was known: *O. mexicana* A. Rich. & Galeotti, restricted to Guatemala and Mexico. The second representative of the genus was described based on Colombian material (Kolanowska & Szlachetko, 2013), and soon after that, additional transfers of two South American *Cranichis* species were proposed: *C. carlos-parrae* Szlach. & Kolan. and *C. crumenifera* Garay to *Ocampoa* (Kolanowska & Szlachetko, 2015). Recent studies on Andean orchids have revealed the existence of six additional *Cranichis* species which morphologically correspond to *Ocampoa* (Szlachetko & Kolanowska, 2017b). During the course of this study two additional transfers were deemed to be necessary, as both of these *Cranichis* species share all features of *Ocampoa*:

***Ocampoa asplundii*** (Garay) Szlach. & Kolan., ***comb. nov***.
Basionym: *Cranichis asplundii* Garay, Canad. J. Bot 34: 248. 1956. TYPE: Ecuador. *E. Asplund 9553* (Holotype: S; Isotype: AMES!). *Solenocentrum asplundii* (Garay) Garay, Opera Bot., B 9(225: 1): 229. 1978.

***Ocampoa stenopetala*** (Dressler) Szlach. & Kolan., ***comb. nov***.
Basionym: *Baskervilla stenopetala* Dressler, Bol. Inst. Bot. (Univ. Guadalajara) 5(1–3): 70, f. 1. 1998. TYPE: Panama. *J. Folsom s.n*. (Holotype: MO!).

## Conclusions

The compiled list of Colombian representatives of the genus totals 42 species, including 10 new species described in this paper. We were not able to confim the occurence of the two *Cranichis* species previously reported from the country, being *C. picta* Rchb.f. and *C. tenuiflora* Griseb. Lectotypes were selected for six species: *C. ciliata*, *C. fendleri*, *C. mandonii*, *C. tenuis*, *C. viereckii*, and *C. wageneri*.

Most national representatives of *Cranichis* are restricted in their distribution to the Andean region; however, five species have also been reported from Central America. In Colombia, species of *Cranichis* are found within an altitudinal range of 500–3,200 m with the greatest altitudinal amplitude observed in *C. ciliata* (2,250 m range). The highest number of *Cranichis* species occur in the Cauca and Magdalena river valleys. Plants grow in disturbed forests, along roadsides, as well as in wet premontane, montane, and cloud forests as well as in paramo. *Cranichis mandonii* was found in a *Cupressus* plantation.

## Supplemental Information

10.7717/peerj.7385/supp-1 Supplemental Information 1 Altitudinal range of Colombian *Cranichis* (Raw data for Figure 1).

10.7717/peerj.7385/supp-2 Supplemental Information 2 Ecoregional species richness of Colombian *Cranichis* (Raw data for Figure 2).

10.7717/peerj.7385/supp-3 Supplemental Information 3 List of collections examined.

## Additional Information and Declarations

### New Species Registration

The following information was supplied regarding the registration of a newly described species: Cranichis callejasii: 77199259-1; Cranichis roldanii: 77199260-1; Cranichis schultesii: 77199261-1; Cranichis barkleyi: 77199262-1; Cranichis killipii: 77199263-1; Cranichis juajibioyi: 77199264-1; Cranichis neglecta: 77199265-1; Cranichis pennellii: 77199268-1; Cranichis popayánensis: 77199271-1; Cranichis pseudomuscosa: 77199273-1; Ocampoa asplundii: 77199274-1; Ocampoa stenopetala: 77199275-1.

### Data Availability

The following information was supplied regarding data availability: The dataset of altitudinal range of each Colombian Cranichis species as well as database of ecoregions in which they occur are available as Files S1 and S2. All data were obtained from the examined herbarium specimens labels. The specimens are identified by collector number and they are deposited in herbaria of Aarhus University (AAU), Harvard University (AMES), Natural History Museum (BM), University of Copenhagen (C), Institut de Recherche pour le Developpement (CAY), Universidad del Valle (CUVC), Universidad Nacional de Colombia (COL), Instituto de Investigación de Recursos Biológicos Alexander von Humboldt (FMB), Royal Botanic Gardens (K), Missouri Botanical Garden (MO), New York Botanical Garden (NY), Muséum National d’Histoire Naturelle (P), Universidad de Nariño (PSO), University of Basel (RENZ), Río Palenque Science Center (RPSC), Smithsonian Institution (US), Universidad Nacional de Colombia sede Palmira (VALLE), and Naturhistorisches Museum Wien (W). The illustrations deposited in Harvard University (AMES), Gdansk University (UGDA), and University of Basel (RENZ) were examined. The complete list of 245 collections studied (including the collector name and number as well as location of each specimen) is available in Dataset S3.

## References

[ref-1] Acuña J (1939). Catalogo descriptivo de las Orquideas Cubanas. Boletín, Estaçión Experimental Agronómica de Santiago de las Vegas.

[ref-2] Álvarez-Molina A, Cameron KM (2009). Molecular phylogenetics of Prescottiinae s.l. and their close allies (Orchidaceae, Cranichideae) inferred from plastid and nuclear ribosomal DNA sequences. American Journal of Botany.

[ref-3] Ames O, Correll DS (1952). Orchids of Guatemala. Fieldiana, Botany.

[ref-4] Ames O, Correll DS (1985). Orchids of Guatemala and Belize.

[ref-5] Bernal R, Gradstein SR, Celis M (2016). Catálogo de plantas y líquenes de Colombia.

[ref-6] Bonilla Morales MM, Mosquera Hernández JJ, Petini-Benelli A (2017). A new species of *Catasetum* (Orchidaceae: Catasetinae) from Casanare, Colombia. Lankesteriana.

[ref-7] Breedlove DE (1986). Flora de Chiapas. Listados Florísticos De México.

[ref-8] Chase MW, Cameron KM, Freudenstein JV, Pridgeon AM, Salazar G, Van Den Berg C, Schuiteman A (2015). An updated classification of Orchidaceae. Botanical Journal of the Linnean Society.

[ref-9] Christenson EA (1991). Mesoamerican orchid studies I: orchids of Panama. Lindleyana.

[ref-10] Christenson EA (2004). Deux nouvelles orchidées terrestres d’Équateur. Richardiana.

[ref-11] Cogniaux A, Urban I (1911). Cranichis. Orchidaceae. Symbolae Antillanae, seu, Fundamenta florae Indiae Occidentalis.

[ref-12] Correa MN (1972). Una nueva especie y dos nuevas citas de Orchidaceae para la flora de Jujuy. Bulletin of the Botanical Society of Argentina.

[ref-13] Correa A, Galdames C, Stapf M (2004). Catálogo de las plantas vasculares de Panamá.

[ref-14] Dodson CH (1996). New Orchid species and combinations from Ecuador – 4. Orquideologia.

[ref-15] Dodson CH, Luer C, Harling G, Andersson L (2005). Orchidaceae. Genera *Aa-Cyrtidiorchis*. Flora of Ecuadorz.

[ref-58] Dodson CH, Vásquez R (1989). Orchids of Bolivia. Icones plantarum tropicarum.

[ref-16] Dressler RL (1993). Phylogeny and classification of the orchid family.

[ref-17] Dressler RL, Hammel BE, Grayum MH, Herrera C, Zamora N (2003). Orchidaceae. Manual de Plantas de Costa Rica.

[ref-18] Dueñas Gómez HC, Fernández Alonso JL (2009). Sinopsis de la subfamilia Spiranthoideae (Orchidaceae) en Colombia, parte II. Revista de la Academia Colombiana de Ciencias Exactas, Físicas y Naturales.

[ref-19] Dunsterville GCK, Garay LA (1966). Venezuelan orchids illustrated.

[ref-22] Foldats E, Lasser T (1969). Orchidaceae. Flora de Venezuela.

[ref-23] Garay LA, Harling G, Sparre B (1978). 225(1) Orchidaceae, Cypripedioideae, Orchidoideae, Neottioideae. *Ponthieva*. Flora of Ecuador.

[ref-24] Garay LA, Romero González G (1999). Schedulae Orchidium II. Harvard Papers in Botany.

[ref-25] Hágsater E, Soto MA, Salazar GA, Jiménez R, López MA, Dressler RL (2005). Orchids of Mexico.

[ref-26] Hamer F (1974). Las Orquideas de El Salvador.

[ref-27] Hamer F (1982). Orchids of Nicaragua. Icones plantarum tropicarum.

[ref-28] Hamer F (1985). Orchids of Nicaragua. Icones plantarum tropicarum.

[ref-29] Hamer F (1988). Orchids of Central America. Selbyana.

[ref-62] Kolanowska M, Szlachetko DL (2013). Recognition of Ponthieva micromystax and a new Ocampoa species (Orchidaceae). Annales Botanici Fennici.

[ref-30] Kolanowska M, Szlachetko DL (2014). *Cranichis badia*, *C. brevirostris* and *C. silvicola* spp. nov. (Cranichidinae) from Colombia and Venezuela. Nordic Journal of Botany.

[ref-61] Kolanowska M, Szlachetko DL (2015). Overview of Cranichis (Orchidaceae, Cranichidinae) and allied genera with notes on their Colombian representatives. Plant Systematics and Evolution.

[ref-31] Llamacho JA, Larramendi JA (2005). The orchids of Cuba/Las Orquídeas de Cuba.

[ref-32] Moreno JS, Vieira-Uribe S, Karremans AP (2017). A new species of *Lepanthes* (Orchidaceae: Pleurothallidinae) from Colombia with a large and protruding column. Lankesteriana.

[ref-33] Olson DM, Dinerstein E, Wikramanayake ED, Burgess ND, Powell GVN, Underwood EC, D’Amico JA, Itoua I, Strand HE, Morrison JC, Loucks CJ, Allnutt TF, Ricketts TH, Kura Y, Lamoreux JF, Wettengel WW, Hedao P, Kassem KR (2001). Terrestrial ecoregions of the world: a new map of life on earth. BioScience.

[ref-34] Ortiz Valdivieso P, Uribe Vélez C (2007). Gallery of Colombian orchids.

[ref-35] Pfitzer E (1887). Entwurf einer natürlichen Anordnung der Orchideen.

[ref-36] Pridgeon AM, Cribb PJ, Chase MW, Rasmussen FN (2003). Genera Orchidacearum.

[ref-37] Pupulin F (2005). Vanishing beauty. Native Costa Rican orchids.

[ref-59] Renz J (1948). Beitráge zur Kenntnis der süd- und zentralamerikanischen Orchideen. l. Orchidaceae-Cranichidinae. Candollea.

[ref-60] Richard A, Galeotti H (1845). Orchidographie mexicaine, d'après les échantillons, notes et dessins de MM. Galeotti, Linden, Funck, Ghiesbreght. Annales des Sciences Naturelles, Botanique.

[ref-38] Schlechter R (1917). Orchidaceae novae et criticae, Decas XLIX–L. Additamenta ad Orchideologiam ecuadorensem III. Feddes Repertorium.

[ref-39] Schlechter R (1919). Orchideenfloren der Suedamerikanischen Kordillerenstaaten, I. Venezuela. Feddes Repertorium, Beihefte.

[ref-40] Schlechter R (1920). Orchideenfloren der Suedamerikanischen Kordillerenstaaten, II. Colombia. Feddes Repertorium, Beihefte.

[ref-41] Schlechter R (1921). Orchideenfloren der Suedamerikanischen Kordillerenstaaten, IV. Peru. Feddes Repertorium, Beihefte.

[ref-42] Schlechter R (1923). Beitraege zur Orchideenkunde von Zentralamerika, II. Additamenta ad Orchideologiam Costaricensem, II. Orchidaceae Bradeanae Costaricenses. Feddes Repertorium, Beihefte.

[ref-43] Schneider M (1953). El Género *Cranichis* (Orchidaceae) en Colombia. Caldasia.

[ref-44] Schultes RE (1960). Native orchids of Trinidad and Tobago.

[ref-45] Schweinfurth C (1958). Orchidaceae, orchids of Peru. Fieldiana, Botany.

[ref-46] Schweinfurth C (1970). First supplement to the Orchids of Peru. Fieldiana, Botany.

[ref-47] Swartz O (1788). Nova Genera Species Plantarum seu Prodromus descriptionum Vegetabilium maximam partem incognitorum quae sub itinere Indiam Occidentalem annis 1783 – 1787.

[ref-48] Szlachetko DL (1995). Systema Orchidalinum. Fragmenta Floristica et Geobotanica Polonica, Supplementum.

[ref-49] Szlachetko DL, Kolanowska M (2013a). Four new species of *Cranichis* (Spiranthoideae, Cranichidinae) from Colombia. Polish Botanical Journal.

[ref-50] Szlachetko DL, Kolanowska M (2013b). New species of the genus *Cranichis* (Cranichidinae) from Colombia. Plant Systematics and Evolution.

[ref-51] Szlachetko DL, Kolanowska M (2017a). Materials to the orchid flora of Colombia.

[ref-52] Szlachetko DL, Kolanowska M (2017b). Synopsis of the genus *Ocampoa* (Orchidaceae). Annals of the Missouri Botanical Garden.

[ref-53] Thiers B (2018). Index Herbariorum: a global directory of public herbaria and associated staff.

[ref-54] Turland NJ, Wiersema JH, Barrie FR, Greuter W, Hawksworth DL, Herendeen PS, Knapp S, Kusber W-H, Li D-Z, Marhold K, May TW, McNeill J, Monro AM, Prado J, Price MJ, Smith GF (2018). International Code of Nomenclature for algae, fungi, and plants (Shenzhen Code). 19th International Botanical Congress Shenzhen, China, July 2017. Regnum Vegetabile 159.

[ref-55] Williams LO (1951). Orchidaceae of Mexico. Ceiba.

[ref-56] Williams LO (1972). Tropical American plants XII (Orchidaceae). Fieldiana, Botany.

[ref-57] Williams LO, Allen PH, Dressler RL (1980). Orchids of Panama: a facsimile reprint of “The Orchidaceae”.

